# Enantioselective synthesis of α-alkenyl α-amino acids *via* N–H insertion reactions†
†Electronic supplementary information (ESI) available: Experimental procedures; spectral data for all new compounds; HPLC or SFC charts for all insertion products. See DOI: 10.1039/c5sc03558a


**DOI:** 10.1039/c5sc03558a

**Published:** 2015-10-28

**Authors:** Jun-Xia Guo, Ting Zhou, Bin Xu, Shou-Fei Zhu, Qi-Lin Zhou

**Affiliations:** a State Key Laboratory and Institute of Elemento-Organic Chemistry , Nankai University , Tianjin 300071 , China; b Collaborative Innovation Center of Chemical Science and Engineering (Tianjin) , Nankai University , Tianjin 300071 , China . Email: sfzhu@nankai.edu.cn ; Fax: +86-22-2350-4087

## Abstract

A new highly enantioselective route to α-alkenyl α-amino acid derivatives, which are important naturally occurring compounds with attractive bioactivity and synthetic utility, was developed using a N–H insertion reaction of vinyldiazoacetates and *tert*-butyl carbamate cooperatively catalyzed by achiral dirhodium(ii) carboxylates and chiral spiro phosphoric acids under mild, neutral conditions. This reaction has a broad substrate scope, a fast reaction rate (turnover frequency > 6000 h^–1^), a high yield (61–99%), and excellent enantioselectivity (83–98% ee). The chiral spiro phosphoric acid, which is proposed to realize the enantioselectivity of the insertion reaction by promoting the proton transfer of a ylide intermediate by acting as a chiral proton shuttle catalyst, can suppress several usual side reactions of vinyldiazoacetates and broaden the applications of these versatile carbene precursors in organic synthesis. To our knowledge, it is the first highly enantioselective carbene insertion reaction of vinyldiazoacetates with heteroatom–hydrogen bonds in which the heteroatom has lone-pair electrons.

## Abstract 2

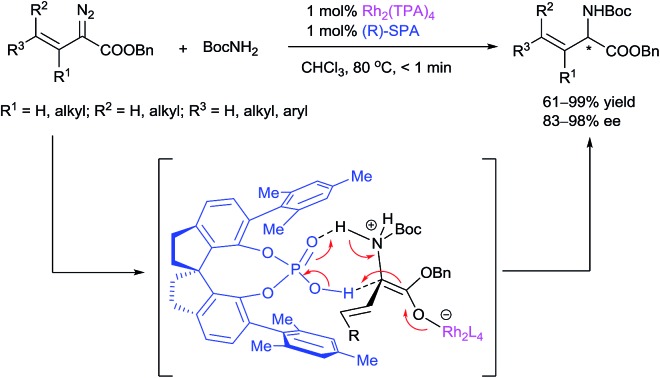
A new highly enantioselective route to α-alkenyl α-amino acid derivatives using a N–H insertion reaction of vinyldiazoacetates and *tert*-butyl carbamate cooperatively catalyzed by achiral dirhodium(ii) carboxylates and chiral spiro phosphoric acids was developed.

α-Amino acids are vital building blocks of peptides, proteins, and many other bioactive compounds, and the development of highly efficient and enantioselective methods for the synthesis of diverse α-amino acids has been a long-standing goal of synthetic chemists. Over the past several decades, many catalytic methods have been established for the synthesis of α-alkyl and α-aryl substituted α-amino acids.^1^ However, even though chiral α-alkenyl α-amino acids are important naturally occurring compounds^2^ with attractive bioactivity^3^ and synthetic utility (Fig. 1 and Scheme 1),^4^ few enantioselective catalytic methods for their synthesis have been reported.^5^ Moreover, only two types of chiral α-alkenyl α-amino acids (γ-mono-substituted vinylglycines5a,b and β-carbonyl vinylglycines5c,d) can be prepared *via* these reported methods. Therefore, general enantioselective catalytic methods for preparing optically active α-alkenyl α-amino acids and their derivatives are highly desired. The challenge in the enantioselective synthesis of chiral α-alkenyl α-amino acids lies in the lability of the products toward racemization and undesirable isomerization of the double bond.

**Fig. 1:**
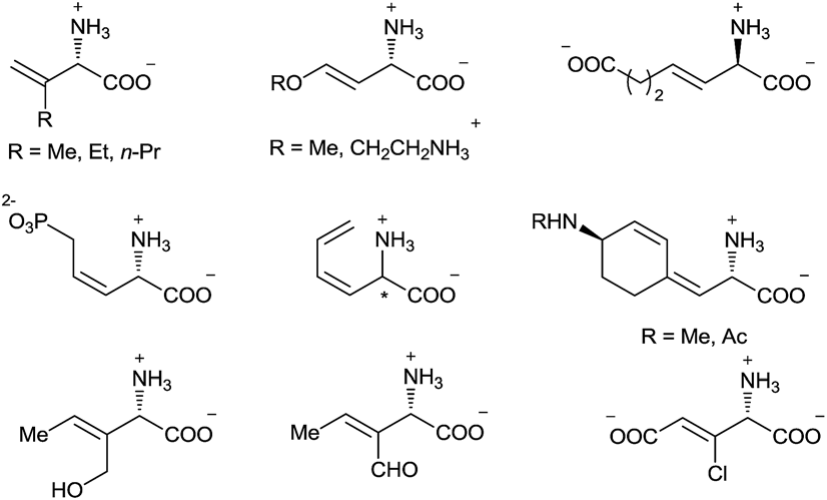
Selected naturally occurring α-alkenyl α-amino acids.

**Scheme 1:**
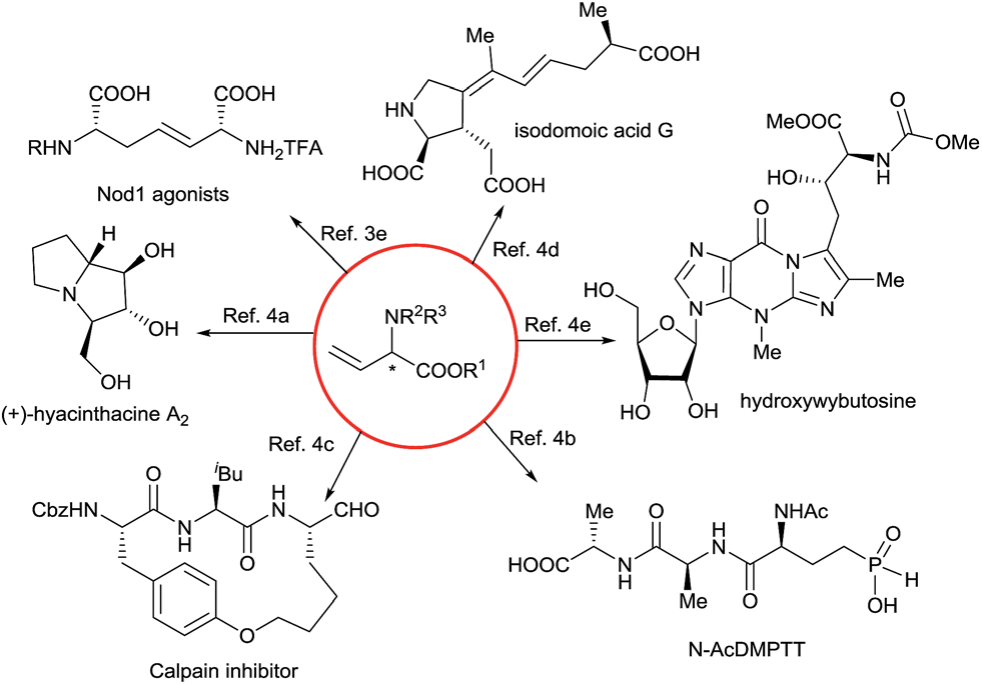
Synthetic utilities of vinylglycines.

Transition-metal-catalyzed carbenoid insertion into N–H bonds is one of the most efficient methods for constructing C–N bonds, and remarkable progress in asymmetric N–H insertion reactions has been achieved in recent years.^6^ However, asymmetric N–H insertion reactions of vinyldiazoacetates, which could be used to produce chiral α-alkenyl α-amino acid derivatives, remain an unresolved problem.^7^ Such reactions can be expected to be challenging because the highly reactive olefin moiety of the vinyldiazoacetates might undergo migration, rearrangement, or cyclopropanation in the presence of traditional metal-complex catalysts.^8^ For instance, Doyle and co-workers^9^ studied the reaction of 3-(trialkylsiloxy)-2-diazo-3-butenoate with aldehyde-derived hydrazones using chiral dirhodium catalysts but found that the reaction occurred at the vinyl terminus (referred to as vinylogous N–H insertion), instead of at the α position, to generate α,β-unsaturated γ-amino acid derivatives. Fu and co-workers6*f* described an asymmetric N–H insertion of 2-diazo-4-phenylbut-3-enoate with good enantioselectivity (87% ee) but very low yield (25%) in a footnote (experimental data not given). Herein we report that vinyldiazoacetates and *tert*-butyl carbamate undergo a highly enantioselective N–H insertion reaction cooperatively catalyzed by achiral dirhodium(ii) carboxylates and chiral spiro phosphoric acids (SPAs) under mild, neutral conditions. This reaction, which constitutes a new route to α-alkenyl α-amino acid derivatives, has a broad substrate scope, a fast reaction rate (turnover frequency > 6000 h^–1^), a high yield (61–99% yields), and excellent enantioselectivity (83–98% ee). The SPA is proposed to promote the proton transfer of a ylide intermediate by acting as a chiral proton shuttle catalyst, consequently achieving the enantioselectivity of the insertion reaction. Moreover, the SPA suppresses several usual side reactions of vinyldiazoacetates and broadens the applications of these versatile carbene precursors in organic synthesis. To our knowledge, this is the first highly enantioselective carbene insertion reaction of vinyldiazoacetates with heteroatom–hydrogen bonds in which the heteroatom has lone-pair electrons.^10^

To evaluate various chiral catalysts, we carried out the insertion reaction of (*E*)-benzyl 2-diazopent-3-enoate (**1a**) with *tert*-butyl carbamate in CHCl_3_ at 25 °C (Table 1). The traditional chiral metal-complex catalysts for carbene insertion reactions, including copper and palladium complexes with chiral spirobisoxazoline ligand **3**,^11^ Rh_2_(*R*-DOSP)_4_,^12^ and Rh_2_(*S*-PTAD)_4_,^13^ exhibited only modest yields and low enantioselectivities (entries 1–4). We next turned to cooperative catalysts composed of achiral dirhodium complexes and SPAs **4**, which may accelerate the proton shift step of the insertion reaction by acting as chiral proton shuttles (entries 5–13).^14^ The use of the SPAs significantly increased the yields of the desired N–H insertion products and suppressed double bond rearrangement and carbene dimerization. The SPA (*R*)-**4g**, which bears 6,6′-di[2,4,6-(Me)_3_C_6_H_2_] substituents, exhibited the best performance (78% yield, 61% ee; entry 11). The investigation of various achiral dirhodium complexes revealed that the steric characteristics of the complexes strongly affected the enantioselectivity of the N–H insertion reaction (entries 14–16). With dirhodium complex Rh_2_(TPA)_4_, which has bulky carboxylate ligands, the reaction was complete in <1 min and afforded a high yield (74%) of the desired product with excellent enantioselectivity (96% ee) (entry 16). Considering their significant effects on the enantioselectivity of the reaction, the rhodium catalysts are most likely involved in the proton transfer step.6*i* Chlorinated solvents dichloromethane and dichloroethane were suitable for the N–H insertion reaction, whereas the use of THF or toluene dramatically lowered the enantioselectivity (to 3% ee and 6% ee, respectively; Table S1, ESI†). Increasing the reaction temperature increased the yield of the reaction (entries 17–19). The unexpected high enantioselectivity at 80 °C (entry 19) implies that the SPAs can effectively promote the proton shift of the unstable ylide intermediate in this insertion reaction even under harsh conditions.6i,14h

**Table 1:** Asymmetric N–H insertion reactions of (*E*)-benzyl 2-diazopent-3-enoate (**1a**) with BocNH_2_^*a*^

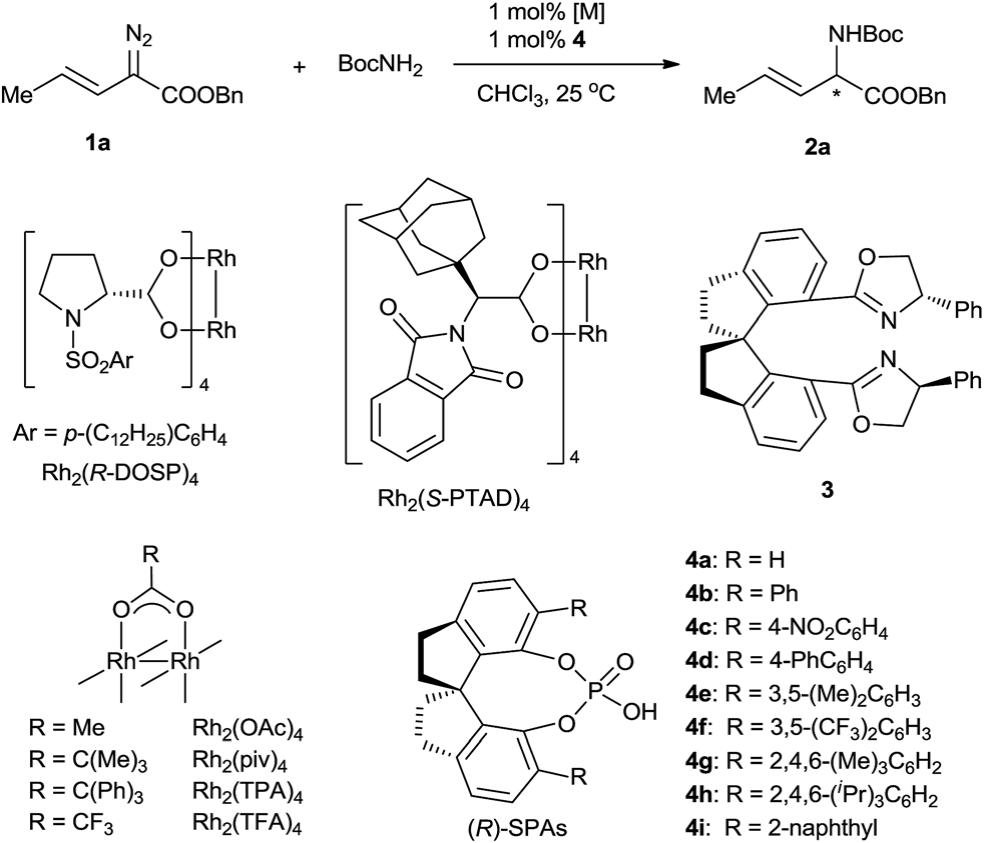					
Entry	[M]	SPA	Time	Yield^*b*^ (%)	ee^*c*^ (%)
1^*d*^	Pd(PhCN)_2_Cl_2_ and **3**	None	12 h	<5	—
2^*d*^	Cu(MeCN)_4_PF_6_ and **3**	None	12 h	37	11
3	Rh_2_(*R*-DOSP)_4_	None	3 min	23	12
4	Rh_2_(*S*-PTAD)_4_	None	1 min	41	12
5	Rh_2_(OAc)_4_	(*R*)-**4a**	2 min	66	4
6	Rh_2_(OAc)_4_	(*S*)-**4b**	3 min	66	–37
7	Rh_2_(OAc)_4_	(*R*)-**4c**	4 min	64	11
8	Rh_2_(OAc)_4_	(*R*)-**4d**	2 min	60	57
9	Rh_2_(OAc)_4_	(*R*)-**4e**	4 min	76	19
10	Rh_2_(OAc)_4_	(*R*)-**4f**	2 min	53	31
11	Rh_2_(OAc)_4_	(*R*)-**4g**	2 min	78	61
12	Rh_2_(OAc)_4_	(*S*)-**4h**	3 min	58	–2
13	Rh_2_(OAc)_4_	(*R*)-**4i**	3 min	64	34
14	Rh_2_(piv)_4_	(*R*)-**4g**	<1 min	37	71
15	Rh_2_(TFA)_4_	(*R*)-**4g**	3 h	35	59
16	Rh_2_(TPA)_4_	(*R*)-**4g**	<1 min	74	96
17^*e*^	Rh_2_(TPA)_4_	(*R*)-**4g**	<1 min	66	95
18^*f*^	Rh_2_(TPA)_4_	(*R*)-**4g**	<1 min	86	97
19^*g*^	Rh_2_(TPA)_4_	(*R*)-**4g**	<1 min	93	96

^*a*^Reaction conditions: [Rh]/**4**/**1a**/BocNH_2_ = 0.002 : 0.002 : 0.2 : 0.2 (mmol) in 3 mL CHCl_3_, 25 °C.

^*b*^Isolated yield.

^*c*^Determined using HPLC using a Chiralcel OD-H column.

^*d*^Using 5 mol% catalyst.

^*e*^Performed at 0 °C.

^*f*^Performed at 60 °C.

^*g*^Performed at 80 °C.

Using the optimized reaction conditions, we carried out reactions of vinyldiazoacetate substrates **1** bearing various substituents on the C=C bond (Table 2).^15^ Impressively, all the reactions were complete within 1 min (turnover frequency > 6000 h^–1^). The length of the γ-alkyl group (R^3^) had a negligible effect on the yield and enantioselectivity of the reaction (**1a–1d**). The reaction of γ-isopropyl-substituted substrate **1e** proceeded in a slightly lower yield, and this result may be attributable in part to increased steric hindrance due to the branched alkyl group. (*E*)-Benzyl 2-diazo-4-phenylbut-3-enoate (**1f**), which has a γ-phenyl group, also underwent the insertion reaction, but the enantioselectivity was lower than that for vinyldiazoacetates with a γ-alkyl group. The N–H insertion reactions of γ,γ-disubstituted vinyldiazoesters **1g–1n** also exhibited excellent enantioselectivities. Substrates with substituted phenyl groups (**1h–1j**), fused rings (**1k** and **1l**), and a thiophenyl moiety (**1m**) were tolerated. The reaction of benzyl 2-diazo-4-methylpent-3-enoate (**1n**), which has γ,γ-dimethyl groups, afforded excellent enantioselectivity (98% ee) albeit with a relatively low yield (66%). Benzyl 2-diazobut-3-enoate (**1o**), which bears a terminal olefin, was also a suitable substrate for the N–H insertion reaction, which afforded the ester form of vinylglycine in high yield with excellent enantioselectivity. A vinyldiazoacetate with a cyclic olefin moiety (**1p**) also afforded the desired product (61% yield, 94% ee). The benzyloxy functional group in the side chain of vinyldiazoacetate **1q** was also tolerated. The size of the ester moiety slightly affected the enantioselectivity; bulkier esters gave higher enantioselectivities (compare substrates **1g**, **1r**, and **1s**). The cooperative catalytic system is highly active. The catalyst loading could be reduced to 0.5 mol% or even 0.3 mol% catalyst without significantly affecting the outcome of the N–H insertion reaction of diazoester **1s**. The further reduction in the catalyst loading to 0.2 mol% and 0.1 mol%, leads to lower enantioselectivity (83% ee and 77% ee, respectively). The reaction could be easily performed at a gram scale (Scheme 2), which demonstrates its potential for practical applications.

**Table 2:** Asymmetric N–H insertion reactions of vinyldiazoacetates with BocNH_2_^*a*^

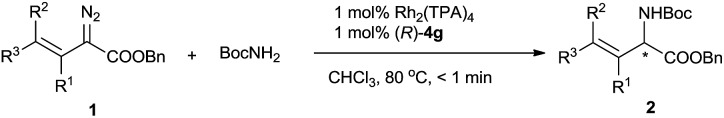
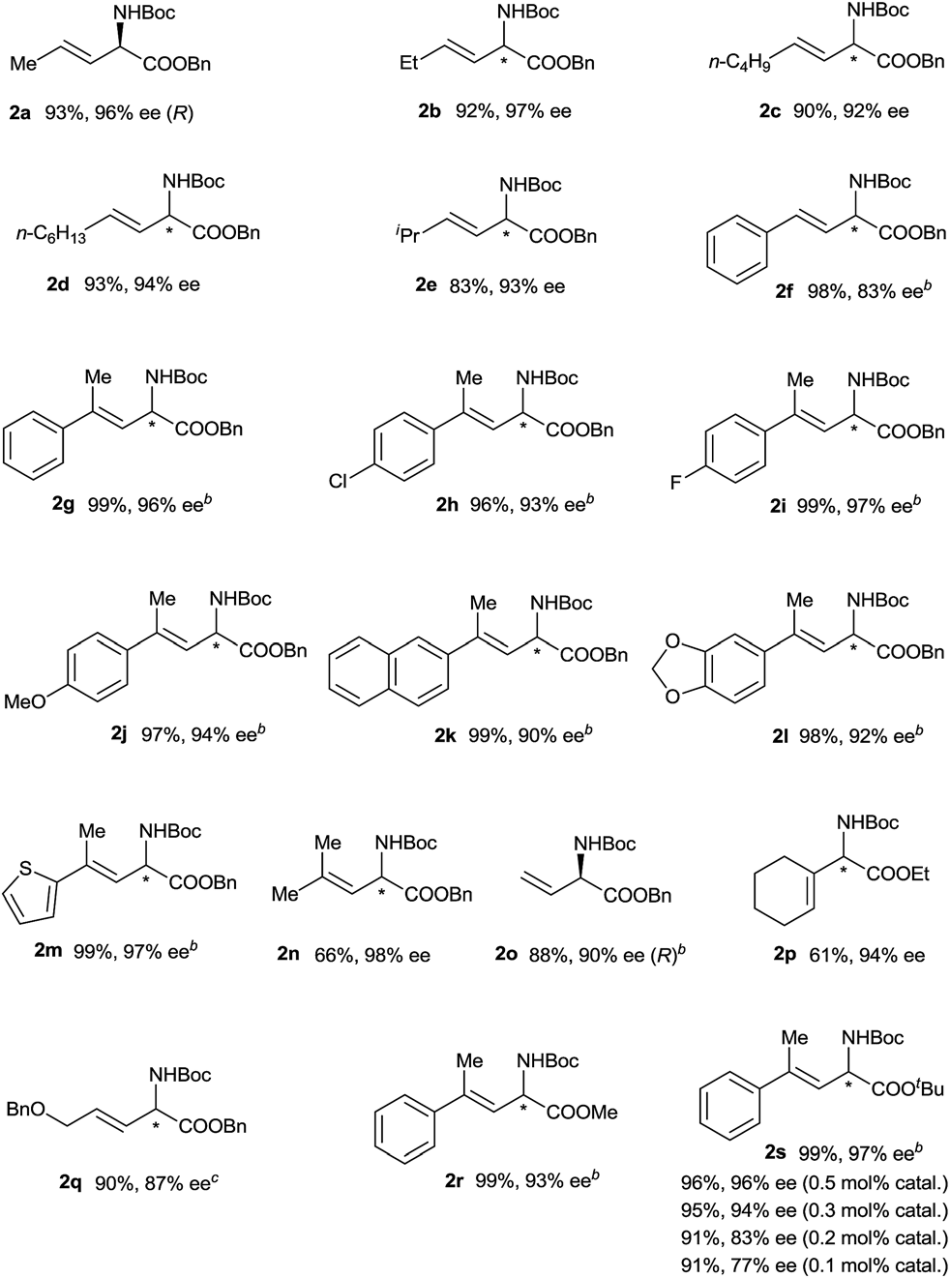

^*a*^The reaction conditions and analysis methods were the same as those described in Table 1.

^*b*^Using 1.5 equiv. of **1**.

^*c*^Using (*S*)-**4g**.

**Scheme 2:**
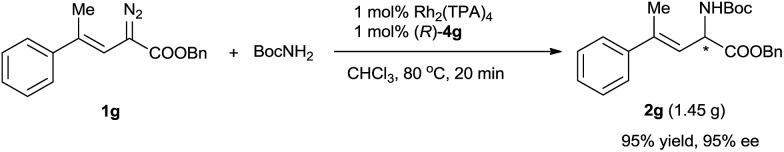
The gram-scale experiment.

This N–H insertion reaction of vinyldiazoacetates provides a new route to chiral α-amino acids. For example, l-vinylglycine was easily prepared in good yield by the hydrolysis of insertion product (*S*)-**2o** (Scheme 3, eqn (1)).^16^ Hydrogenation of (*R*)-**2b** over a Pd/C catalyst and subsequent acidic hydrolysis gave α-alkyl-α-amino acid **6** (94% yield, eqn (2)).^16^ The olefin moieties in the products can be expected to undergo transformations such as dihydroxylation, cyclopropanation, and epoxidation,^4^,^16^ making this reaction potentially useful for synthesizing various optically active α-amino acid derivatives.

**Scheme 3:**
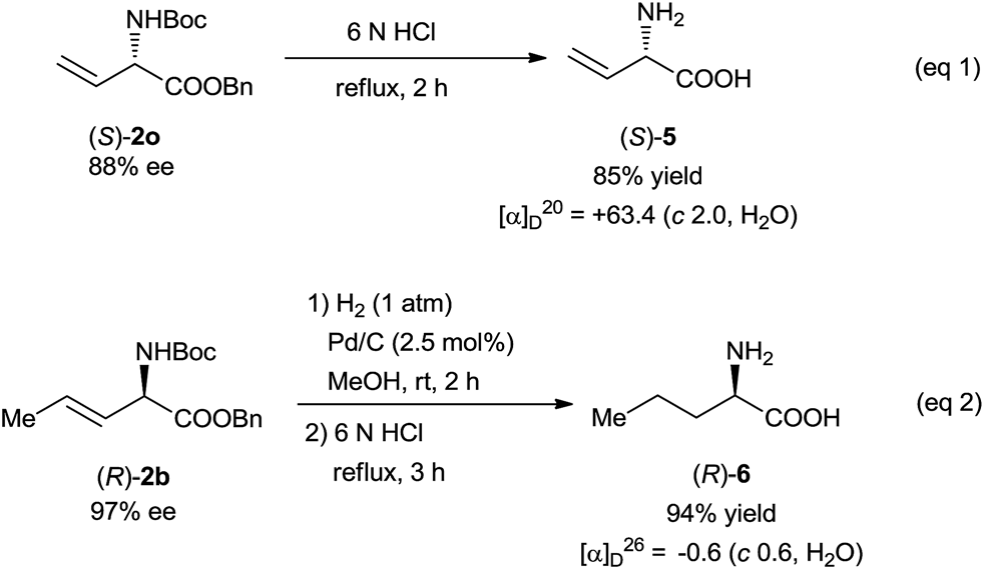
Synthesis of optically active α-amino acids.

In summary, a highly efficient asymmetric synthesis of α-alkenyl α-amino acids was realized by means of the N–H insertion reaction of vinyldiazoacetates with *tert*-butyl carbamate cooperatively catalyzed by achiral dirhodium(ii) carboxylates and chiral SPAs. The wide substrate scope, good yield, high enantioselectivity, fast reaction rate, and mild, neutral conditions make this N–H insertion reaction widely applicable for the preparation of chiral α-amino acid derivatives. The combination of SPAs and dirhodium(ii) carboxylates exhibits a special advantage in the transformation of highly functionalized vinyldiazoacetates by minimizing the side-reactions, and has potential applications in other enantioselective transformations involving vinyldiazoacetates.

## Supplementary Material

Supplementary information
