# Supplementary material for: Enantioselective synthesis of α-alkenyl α-amino acids via N–H insertion reactions
Source: Chem Sci. 2015 Oct 28;7(2):1104–8. doi: 10.1039/c5sc03558a (PMC5975786; doi:10.1039/c5sc03558a)
Supplement: Supplementary file 1 [file SC-007-C5SC03558A-s001.pdf]

Electronic Supplementary Material (ESI) for Chemical Science.
This journal is © The Royal Society of Chemistry 2015

Electronic Supplementary Information for:

Enantioselective synthesis of α-alkenyl α-amino acids via N−H insertion reactions

Jun-Xia Guo,[a] Ting Zhou,[a] Bin Xu,[a] Shou-Fei Zhu*[a,b] and Qi-Lin Zhou[a,b]

[a] *The State Key Laboratory and Institute of Elemento-Organic Chemistry,* [b] *Collaborative Innovation Center of Chemical Science and Engineering (Tianjin), Nankai University, Tianjin 300071, China*

Email: sfzhu@nankai.edu.cn

Contents:

1. General Information……………………………………………S2

2. Synthesis of (*R*)-4g……………………………………………S2

3. Analytical Data of New Vinyldiazoacetates……………………S4

4. Procedures of N−H Insertions……………………………………S9

5. Analytical Data of N−H Insertion Products……………………S10

6. Transformations of N−H Insertion Products ……………………S16

7. NMR Spectra of New Compounds………………………………S18

8. HPLC Charts of N−H Insertion Products…………………………S58

9. References…………………………………………………………S77

1. General Information

All solvents were purified and dried using standard procedures.[1] Dirhodium (II) (Sigma-Aldrich) and other commercially available reagents were used without further purification. The chiral spiro phosphoric acids (SPAs) **4a**-**4f**, **4h**, **4i** were synthesized according to the reported procedures.[2] Vinyldiazoacetates **1a**-**1s** were prepared according to the literature procedures.[3] All reactions were performed in an argon-filled glovebox (MBRAUN Labstar or Mikrouna super 1220/750) or using standard Schlenk techniques. Melting points were measured on a RY–I apparatus and uncorrected. NMR spectra were recorded with a Bruker AV 400 spectrometer at 400 MHz (^{1}H NMR) and 100 MHz (^{13}C NMR). Chemical shifts (*δ* values) were reported in ppm down field from internal Me_4Si (^{1}H and ^{13}C NMR). High Resolution Mass Spectra (HRMS) were recorded on an IonSpec FT-ICR mass spectrometer with Electron Spray Ionization (ESI) resource. Enantiomeric excesses (ee) of the N–H insertion products were determined by High Performance Liquid Chromatography (HPLC). HPLC analyses were performed on a Hewlett Packard Model HP 1100 Series chromatography.

2. Synthesis of (*R*)-4g

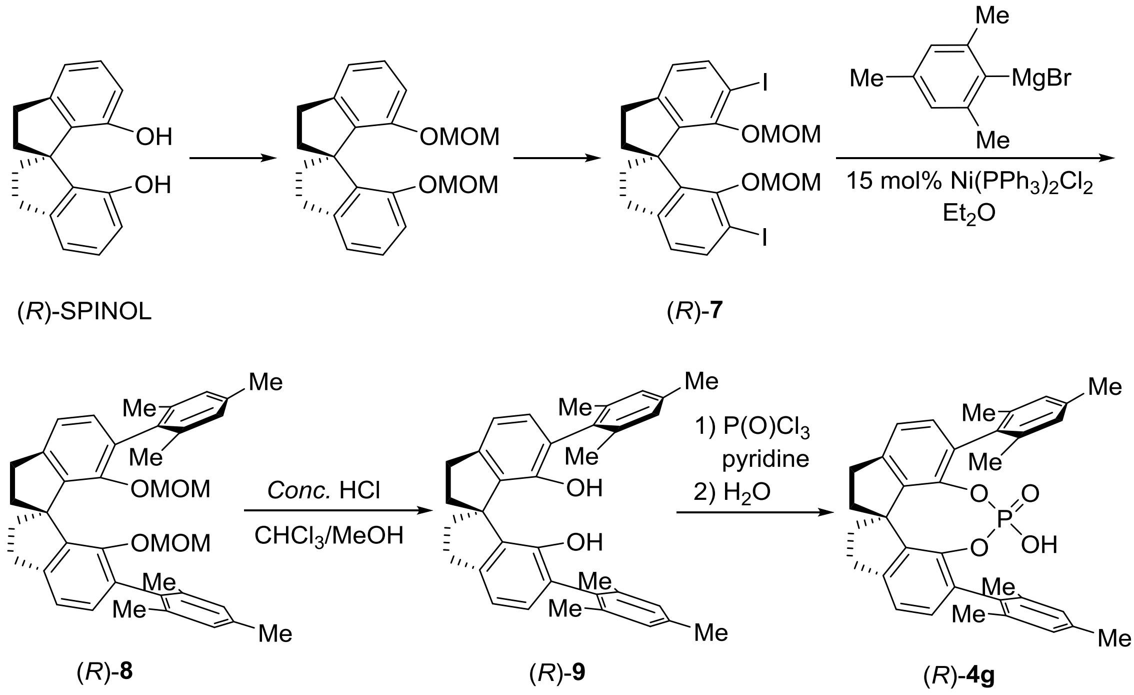

The (*R*)-**7** were prepared from optically pure (*R*)-1,1'-spirobiindane-7,7'-diols (SPINOL) according to our previously reported procedures.[4] A typical procedure for preparation of (*R*)-**4g** is described as following.

In a flame dried 100 mL three neck round botom flask Mg (330 mg, 13.7 mmol) was layered with a minimum amount of anhydrous Et_2O. After addition of 2-bromo-1,3,5-trimethylbenzene (0.1 mL) and a few drops of 1,2-dibromoethane the Grignard reaction was initiated by local heating. After initiation the remaining Et_2O (25 mL in total) and 2-

bromo-1,3,5-trimethylbenzene (1.55 g, 7.8 mmol in total) were added alternately to keep the reaction refluxing without the need for external heating. After complete addition the mixture was refluxed for 16 h. After cooling to ambient temperature this Grignard solution was added dropwise to a mixture of (*R*)-**7** (464 mg, 0.78 mmol) and $Ni(PPh_3)_2Cl_2$ (76 mg, 0.12 mmol) in anhydrous Et_2O (20 mL) upon which the mixture turned dark. The reaction mixture was refluxed for 24 h, cooled to ambient temperature, carefully quenched by slow addition of H_2O and saturated NH_4Cl solution and diluted with CH_2Cl_2. The layers were dried over by $MgSO_4$ and the solvent removed under reduced pressure. The residue was purified by column chromatography (PE/CH_2Cl_2 = 4:1 v/v; PE = petroleum ether) to give (*R*)-**8** as a light yellow solid (206 mg, 46% yield). TLC R_f = 0.40 (PE/CH_2Cl_2 = 4:1 v/v). Mp: 170–171 ^oC. $[\alpha]_D^{26}$ = +191 (*c* 0.93, $CHCl_3$). ^1H NMR (400 MHz, $CDCl_3$) δ 6.90 (d, *J* = 7.6 Hz, 2H, Ar-H), 6.80 (s, 4H, Ar-H), 6.75 (d, *J* = 7.6 Hz, 2H, Ar-H), 4.11 (d, *J* = 5.6 Hz, 2H, Ar-H), 3.86 (d, *J* = 5.2 Hz, 2H, Ar-H), 3.05–2.90 (m, 4H, $2CH_2$), 2.53 (s, 6H, $2OCH_3$), 2.50–2.42 (m, 2H, $2CH_2$), 2.40–2.22 (m, 2H, $2CH_2$), 2.20 (s, 6H, $2ArCH_3$), 2.01 (s, 6H, $2CH_3$), 1.94 (s, 6H, $2CH_3$). ^{13}C NMR (100 MHz, $CDCl_3$) δ 151.9 (2C, Ar-C), 144.6 (2C, Ar-C), 142.3 (2C, Ar-C), 137.1 (2C, Ar-C), 136.5 (2C, Ar-C), 136.4 (2C, Ar-C), 136.1 (2C, Ar-C), 131.1 (2C, Ar-C), 130.3 (2C, Ar-C), 128.0 (4C, Ar-C), 119.9 (2C, Ar-C), 97.5 (2C, $2OCH_2O$), 60.0 (1C, C), 55.9 (2C, $2OCH_3$), 39.8 (2C, $2CH_2$), 31.2 (2C, $2CH_2$), 21.0 (2C, $2CH_3$), 20.9 (2C, $2CH_3$), 20.4 (2C, $2CH_3$). HRMS (ESI) Calcd for $[C_{39}H_{44}O_4Na, M + Na]^+$: 599.3132, Found: 599.3131.

To a solution of (*R*)-**8** (859 mg, 1.49 mmol) in 8 mL $CHCl_3$ and 12 mL MeOH, conc. HCl (8.0 mL) was added and the mixture was heated at reflux for 3 h. After cooled to ambient temperature, the mixture was poured into water, extracted by CH_2Cl_2 and the combined organic phase was washed with saturated $NaHCO_3$ and brine, dried over by $MgSO_4$. The solvent was removed, and the residue was purified by chromatography (PE/EA = 10:1 v/v; EA = ethyl acetate) to give (*R*)-**9** (727 mg, 100% yield) as a white solid. TLC R_f = 0.38 (PE/EA = 10:1 v/v). Mp: 223–224 ^oC. $[\alpha]_D^{25}$ = +271 (*c* 1.0, $CHCl_3$). ^1H NMR (400 MHz, $CDCl_3$) δ 6.99 (d, *J* = 7.2 Hz, 4H, Ar-H), 6.95–6.87 (m, 4H, Ar-H), 4.60 (s, 2H, 2OH), 3.35–3.15 (m, 4H, $2CH_2$), 2.55–2.40 (m, 4H, $2CH_2$), 2.37 (s, 6H, $2CH_3$), 2.10 (s, 6H, $2CH_3$), 1.98 (s, 6H, $2CH_3$). ^{13}C NMR (100 MHz, $CDCl_3$) δ 149.2 (2C, Ar-C), 144.5 (2C, Ar-C), 137.6 (2C, Ar-C), 137.4 (2C, Ar-C), 137.2 (2C, Ar-C), 132.7 (2C, Ar-C), 129.5 (2C, Ar-C), 128.3 (2C, Ar-C), 128.2 (2C, Ar-C), 125.1 (2C, Ar-C), 116.9 (2C, Ar-C), 58.5 (1C, C), 38.2 (2C, $2CH_2$), 31.3 (2C, $2CH_2$), 21.0 (2C, $2CH_3$), 20.4 (2C, $2CH_3$), 20.1 (2C, $2CH_3$). HRMS (ESI) Calcd for $[C_{35}H_{36}O_2Na, M + Na]^+$: 511.2608, Found: 511.2608.

To a 50 mL oven-dried Schlenk flask containing (*R*)-**9** (122 mg, 0.25 mmol) was added 4 mL anhydrous pyridine and freshly distilled $P(O)Cl_3$ (249 mg, 1.63 mmol) under a nitrogen atmosphere. The mixture was stirred for 12 h under 90 ^oC. After cooling to room temperature, 4 mL of H_2O was added. The mixture was stirred for another 12 h under 90 ^oC, then cooled by an ice-bath, followed by slow addition of 35 mL 3N HCl. After stirring for 1 h, the mixture was extracted by $CHCl_3$ (20 mL × 4). The combined organic layer was concentrated and purified by chromatography on silica gel (first PE/EA = 1:2, then MeOH/CH_2Cl_2 = 1:10) to give 744 mg white solid. The white solid was dissolved with 100 mL $CHCl_3$, washed with 3N HCl (50 mL × 3), distilled water (50 mL × 3), and dried

under vacuum to give (*R*)-**4g** as a white solid (136 mg, 98% yield). TLC R_f = 0.35 (PE/EA = 2:1 v/v). Mp: 302–304 °C. $[\alpha]_D^{24}$ = +217 (*c* 0.6, $CHCl_3$). ^{1}H NMR (400 MHz, $CDCl_3$) δ 7.13 (d, J = 7.2 Hz, 2H, Ar-H), 6.99 (d, J = 7.2 Hz, 2H, Ar-H), 6.74 (s, 2H, Ar-H), 6.70 (s, 2H, Ar-H), 3.15–3.04 (m, 2H, CH_2), 2.92–2.82 (m, 2H, CH_2), 2.32–2.23 (m, 2H, CH_2), 2.17 (s, 6H, $2CH_3$), 2.10–2.00 (m, 2H, CH_2), 2.03 (s, 6H, $2CH_3$), 1.96 (s, 6H, $2CH_3$). ^{13}C NMR (100 MHz, $CDCl_3$) δ 144.6 (2C, Ar-C), 143.1 (2C, Ar-C), 139.9 (2C, Ar-C), 136.8 (2C, Ar-C), 136.4 (2C, Ar-C), 136.2 (2C, Ar-C), 133.4 (2C, Ar-C), 132.6 (2C, Ar-C), 131.5 (2C, Ar-C), 128.6 (2C, Ar-C), 127.4 (2C, Ar-C), 121.9 (2C, Ar-C), 59.9 (1C, C), 38.5 (2C, $2CH_2$), 30.1 (2C, $2CH_2$), 21.3 (2C, $2CH_3$), 21.1 (2C, $2CH_3$), 20.4 (2C, $2CH_3$). ^{31}P NMR (162 MHz, $CDCl_3$) δ -9.3 (s). HRMS (ESI) calcd for $[C_{35}H_{34}O_4P^-$, M - H$]^-$: 549.2200, Found: 549.2205.

3. Analytical Data of New Vinyldiazoacetates

(*E*)-Benzyl 2-diazopent-3-enoate (1a)

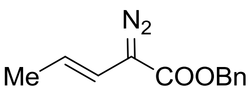

Red oil, TLC R_f = 0.25 (PE/EA = 25:1 v/v), 80% yield. ^{1}H NMR (400 MHz, $CDCl_3$) δ 7.35–7.29 (m, 5H, Ar-H), 5.75 (dd, J_1 = 16.0 Hz, J_2 = 1.6 Hz, 1H, CH=CH), 5.35–5.26 (m, 1H, CH=CH), 5.22 (s, 2H, CH_2Ph), 1.81 (dd, J_1 = 6.8 Hz, J_2 =1.6 Hz, 3H, CH_3). ^{13}C NMR (100 MHz, $CDCl_3$) δ 165.3 (1C, C=O), 135.8 (1C, CH=CH), 128.4 (2C, Ar-C), 128.2 (1C, Ar-C), 128.1 (1C, Ar-C), 128.0 (1C, Ar-C), 120.3 (1C, Ar-C), 112.5 (1C, CH=CH), 66.4 (1C, CH_2Ph), 18.1 (1C, CH_3). IR (neat): 3030w, 2962w, 2881w, 2080s (C=N), 1702s, 1252s, 735w, 697w cm^{-1}. HRMS (ESI) Calcd for $[C_{12}H_{12}N_2O_2Na$, M + Na$]^+$: 239.0791, Found: 239.0793.

(*E*)-Benzyl 2-diazohex-3-enoate (1b)

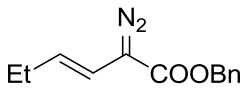

Red oil, TLC R_f = 0.20 (PE/EA = 25:1 v/v), 74% yield. ^{1}H NMR (400 MHz, $CDCl_3$) δ 7.36–7.31 (m, 5H, Ar-H), 5.74 (dt, J_1 = 16.0 Hz, J_2 = 1.6 Hz, 1H, CH=CH), 5.37–5.33 (m, 1H, CH=CH), 5.24 (s, 2H, CH_2Ph), 2.21–2.14 (m, 2H, CH_2), 1.02 (t, J = 7.2 Hz, 3H, CH_3). ^{13}C NMR (100 MHz, $CDCl_3$) δ 165.4 (1C, C=O), 135.8 (1C, CH=CH), 128.5 (1C, Ar-C), 128.2 (2C, Ar-C), 128.1 (1C, Ar-C), 127.2 (2C, Ar-C), 110.7 (1C, CH=CH), 66.5 (1C, CH_2Ph), 25.8 (1C, CH_2), 13.7 (1C, CH_3). IR (neat): 2964w, 2080s (C=N), 1702s, 1455m, 1307m, 1135m, 951m, 735w, 697w cm^{-1}. HRMS (ESI) Calcd for $[C_{13}H_{14}N_2O_2Na$, M + Na$]^+$: 253.0947, Found: 253.0952.

(*E*)-Benzyl 2-diazooct-3-enoate (1c)

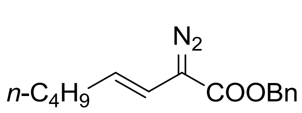

Red oil, TLC R_f = 0.24 (PE/EA = 25:1 v/v), 63% yield. ^{1}H NMR (400 MHz, $CDCl_3$) δ 7.37–7.34 (m, 5H, Ar-H), 5.76–5.71 (m, 1H, CH=CH), 5.34–5.24 (m, 3H, CH=CH and CH_2Ph), 2.19–2.14 (m, 2H, CH_2), 1.38–1.34 (m, 4H, $(CH_2)_2$), 0.90 (t, J = 3.6 Hz, 3H, CH_3). ^{13}C NMR (100 MHz, $CDCl_3$) δ 165.3 (1C, C=O), 135.8 (1C, CH=CH), 128.4 (1C, Ar-C), 128.1 (2C, Ar-C), 128.0 (1C, Ar-C), 125.8 (2C, Ar-C), 111.4 (1C, CH=CH), 66.4 (1C, CH_2Ph), 32.4 (1C, CH_2), 31.5 (1C, CH_2), 22.0 (1C, CH_2), 13.8 (1C, CH_3). IR (neat): 2957m, 2928m, 2080s (C=N), 1703s, 1456m, 1311m, 1257m, 1134m, 1097s, 950m, 735w, 697w cm^{-1}.

HRMS (ESI) Calcd for [$C_{15}H_{18}N_2O_2Na$, M + Na]$^+$: 281.1260, Found: 281.1259.

(*E*)-Benzyl 2-diazodec-3-enoate (1d)

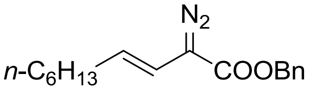

Red oil, TLC R_f = 0.23 (PE/EA = 20:1 v/v), 75% yield. ^{1}H NMR (300 MHz, $CDCl_3$) δ 7.36–7.32 (m, 5H, Ar-H), 5.70 (d, J = 21.2 Hz, 1H, CH=CH), 5.36–5.24 (m, 3H, CH=CH and CH_2Ph), 2.15 (q, J = 7.1 Hz, 2H, CH_2), 1.38–1.27 (m, 8H, $(CH_2)_4$), 0.88 (t, J = 6.3 Hz, 3H, CH_3). ^{13}C NMR (100 MHz, $CDCl_3$) δ 165.5 (1C, C=O), 135.9 (1C, CH=CH), 128.5 (1C, Ar-C), 128.3 (2C, Ar-C), 128.1 (1C, Ar-C), 126.0 (2C, Ar-C), 111.4 (1C, CH=CH), 66.5 (1C, CH_2Ph), 32.8 (1C, CH_2), 31.7 (1C, CH_2), 29.5 (1C, CH_2), 28.7 (1C, CH_2), 22.6 (1C, CH_2), 14.1 (1C, CH_3). IR (neat): 2926m, 2079s (C=N), 1703s, 1243s, 1133s, 735m, 697m cm^{-1}. HRMS (ESI) Calcd for [$C_{17}H_{22}N_2O_2Na$, M + Na]$^+$: 309.1573, Found: 309.1574.

(*E*)-Benzyl 2-diazo-5-methylhex-3-enoate (1e)

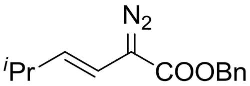

Red oil, TLC R_f = 0.30 (PE/EA = 20:1 v/v), 75% yield. ^{1}H NMR (400 MHz, $CDCl_3$) δ 7.37–7.34 (m, 5H, Ar-H), 5.71 (dd, J_1 = 15.6 Hz, J_2 = 1.2 Hz, 1H, CH=CH), 5.31–5.24 (m, 3H, CH=CH and CH_2Ph), 2.46-2.41 (m, 1H, CH), 1.02 (d, J = 6.8 Hz, 6H, $2CH_3$). ^{13}C NMR (100 MHz, $CDCl_3$) δ 165.5 (1C, C=O), 135.9 (1C, CH=CH), 132.6 (1C, Ar-C), 128.5 (2C, Ar-C), 128.2 (1C, Ar-C), 128.1 (2C, Ar-C), 109.1 (1C, CH=CH), 66.5 (1C, CH_2Ph), 31.4 (1C, CH), 22.5 (2C, CH_3). IR (neat): 3090w, 3034w, 2960m, 2079s (C=N), 1702s cm^{-1}. HRMS (ESI) Calcd for [$C_{14}H_{16}N_2O_2Na$, M + Na]$^+$: 267.1104, Found: 267.1108.

(*E*)-Benzyl 2-diazo-4-phenylbut-3-enoate (1f)

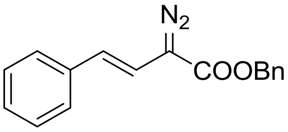

Red solid, TLC R_f = 0.40 (PE/EA = 10:1 v/v), 73% yield. Mp: 52–54 ^{o}C. ^{1}H NMR (400 MHz, $CDCl_3$) δ 7.40–7.29 (m, 9H, Ar-H), 7.22–7.18 (m, 1H, Ar-H), 6.49 (d, J = 16.4 Hz, 1H, CH=CH), 6.20 (d, J = 16.4 Hz, 1H, CH), 5.31 (s, 2H, CH_2Ph). ^{13}C NMR (100 MHz, $CDCl_3$) δ 165.0 (1C, C=O), 136.7 (1C, CH=CH), 135.6 (1C, Ar-C), 128.7 (1C, Ar-C), 128.6 (2C, Ar-C), 128.4 (2C, Ar-C), 128.2 (2C, Ar-C), 127.1 (2C, Ar-C), 125.8 (1C, Ar-C), 123.1 (1C, Ar-C), 111.2 (1C, CH=CH), 66.9 (1C, CH_2Ph). IR (neat): 3059w, 3030w, 2959w, 2079s (C=N), 1701s, 1238s, 734m, 693m cm^{-1}. HRMS (ESI) Calcd for [$C_{17}H_{14}N_2O_2Na$, M + Na]$^+$: 301.0947, Found: 301.0952.

(*E*)-Benzyl 2-diazo-4-phenylpent-3-enoate (1g)

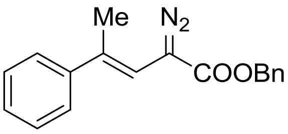

Red oil, TLC R_f = 0.35 (PE/EA = 10:1 v/v), 85% yield. ^{1}H NMR (400 MHz, $CDCl_3$) δ 7.35–7.10 (m, 10H, Ar-H), 5.90 (q, J = 1.1 Hz, 1H, CH=), 5.18 (s, 2H, CH_2Ph), 1.99 (d, J = 1.1 Hz, 3H, CH_3). ^{13}C NMR (100 MHz, $CDCl_3$) δ 166.3 (1C, C=O), 142.4 (1C, C), 135.8 (1C, Ar-C), 134.7 (1C, Ar-C), 128.5 (2C, Ar-C), 128.3 (2C, Ar-C), 128.2 (4C, Ar-C), 127.32 (1C, Ar-C), 125.7 (1C, Ar-C), 108.9 (1C, CH), 66.8 (1C, CH_2Ph), 16.9 (1C, CH_3). IR (neat): 3059w, 3032w, 2955w, 2075s (C=N), 1701s, 1235s, 1108m, 756m, 736m, 696m cm^{-1}. HRMS (ESI) Calcd for [$C_{18}H_{16}O_2Na$, M + Na – N_2]$^+$: 287.1043, Found: 287.1036.

(*E*)-Benzyl 4-(4-chlorophenyl)-2-diazopent-3-enoate (1h)

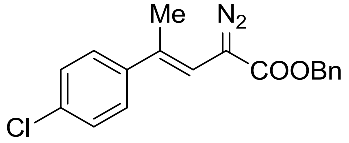

Red solid, TLC R_f = 0.30 (PE/EA = 10:1 v/v), 72% yield. Mp: 42–44 °C. ^{1}H NMR (400 MHz, $CDCl_3$) δ 7.40–7.25 (m, 9H, Ar-H), 5.98 (q, J = 1.2 Hz, 1H, CH=), 5.27 (s, 2H, CH_2Ph), 2.05 (d, J = 0.8 Hz, 3H, CH_3). ^{13}C NMR (100 MHz, $CDCl_3$) δ 166.2 (1C, C=O), 140.9 (1C, C), 135.7 (1C, Ar-C), 133.2 (1C, Ar-C), 133.1 (2C, Ar-C), 128.6 (2C, Ar-C), 128.4 (1C, Ar-C), 128.3 (2C, Ar-C), 128.2 (2C, Ar-C), 127.0 (1C, Ar-C), 109.6 (1C, CH), 66.9 (1C, CH_2Ph), 16.9 (1C, CH_3). IR (neat): 3089w, 3065w, 2953w, 2932w, 2077s (C=N), 1700s, 1439m, 1403m, 1323m, 1295m, 1236s, 1096m, 1010m, 979m, 822m, 751m, 736m, 697m cm^{-1}. HRMS (ESI) Calcd for [$C_{18}H_{15}ClO_2Na$, M + Na – N_2]$^+$: 321.0653, Found: 321.0655.

(*E*)-Benzyl 2-diazo-4-(4-fluorophenyl)pent-3-enoate (1i)

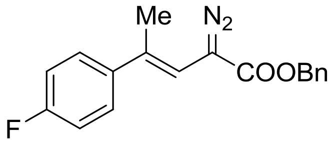

Red solid, TLC R_f = 0.28 (PE/EA = 10:1 v/v), 80% yield. Mp: 36–39 °C.^{1}H NMR (400 MHz, $CDCl_3$) δ 7.30–7.22 (m, 7H, Ar-H), 6.92 (t, J = 8.8 Hz, 2H, Ar-H), 5.84 (q, J = 0.4 Hz, 1H, CH=), 5.18 (s, 2H, CH_2Ph), 1.96 (d, J = 1.2 Hz, 3H, CH_3). ^{13}C NMR (100 MHz, $CDCl_3$) δ 166.3 (1C, C=O), 162.0 (d, J = 245.3 Hz, 1C, Ar-C), 138.5 (d, J = 3.1 Hz, 1C, Ar-C), 135.7 (1C, Ar-C), 133.7 (1C, Ar-C), 128.5 (2C, Ar-C), 128.3 (1C, Ar-C), 128.2 (2C, Ar-C), 127.4 (d, J = 7.8 Hz, 2C, Ar-C), 115.2 (d, J = 21.2 Hz, 2C, Ar-C), 108.9 (1C, CH), 66.8 (1C, CH_2Ph), 17.1 (1C, CH_3). IR (neat): 3065w, 3035w, 2932w, 2855w, 2077s (C=N), 1700s, 1509s, 1235s, 1108m, 827m, 736m, 697m cm^{-1}. HRMS (ESI) Calcd for [$C_{18}H_{15}FO_2Na$, M + Na – N_2]$^+$: 305.0948, Found: 305.0952.

(*E*)-Benzyl 2-diazo-4-(4-methoxyphenyl)pent-3-enoate (1j)

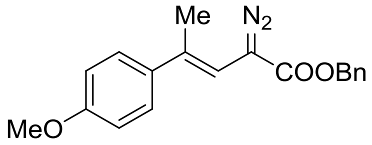

Red solid, TLC R_f = 0.25 (PE/EA = 10:1 v/v), 70% yield. Mp: 82–84 °C.^{1}H NMR (400 MHz, $CDCl_3$) δ 7.38–7.33 (m, 7H, Ar-H), 6.88–6.85 (m, 2H, Ar-H), 5.90 (q, J = 1.2 Hz, 1H, CH), 5.27 (s, 2H, CH_2Ph), 3.81 (s, 3H, OCH_3), 2.05 (d, J = 0.8 Hz, 3H, CH_3). ^{13}C NMR (100 MHz, $CDCl_3$) δ 159.1 (1C, C=O), 135.8 (1C, C), 134.9 (1C, Ar-C), 134.6 (1C, Ar-C), 128.6 (2C, Ar-C), 128.3 (2C, Ar-C), 128.2 (2C, Ar-C), 127.0 (2C, Ar-C), 113.7 (2C, Ar-C), 107.1 (1C, CH), 66.7 (1C, CH_2Ph), 55.3 (1C, OCH_3), 17.1 (1C, CH_3). IR (neat): 3031w, 2982w, 2955w, 2896w, 2839w, 2080s (C=N), 1687s, 1255s, 1103m, 820m, 756m, 735m, 697m cm^{-1}. HRMS (ESI) Calcd for [$C_{19}H_{18}O_3Na$, M + Na – N_2]$^+$: 317.1148, Found: 317.1145.

(*E*)-Benzyl 2-diazo-4-(naphthalen-2-yl)pent-3-enoate (1k)

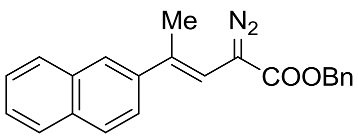

Red solid, TLC R_f = 0.31 (PE/EA = 10:1 v/v), 65% yield. Mp: 88–90 °C.^{1}H NMR (400 MHz, $CDCl_3$) δ 7.82–7.59 (m, 3H, Ar-H), 7.46–7.26 (m, 9H, Ar-H), 6.16 (q, J = 1.1 Hz, 1H, CH), 5.30 (s, 2H, CH_2Ph), 2.20 (d, J = 1.1 Hz, 3H, CH_3). ^{13}C NMR (100 MHz, $CDCl_3$) δ 166.3 (1C, C=O), 139.6 (1C, C), 135.8 (1C, Ar-H), 134.3 (1C, Ar-C), 133.3 (1C, Ar-C), 132.6 (1C, Ar-C), 128.6 (2C, C), 128.3 (1C, Ar-C), 128.2 (2C, Ar-C), 128.1 (1C, Ar-C), 127.9 (1C, Ar-C), 127.5 (1C, Ar-C), 126.2 (1C, Ar-C), 125.9 (1C, Ar-C), 124.4 (1C, Ar-C), 124.0 (1C, Ar-C), 109.4 (1C, CH), 66.8 (1C,

CH$_2$Ph), 17.0 (1C, CH$_3$). IR (neat): 3052w, 3035w, 2897w, 2084s (C=N), 1696s, 1382m, 1318m, 1249m, 1216m, 1117m, 820m, 740m, 698m, 494m, 476m cm^{-1}. HRMS (ESI) Calcd for [$C_{22}H_{18}O_2Na$, M + Na – N$_2$]$^+$: 337.1199, Found: 337.1198.

(*E*)-Benzyl 4-(benzo[d][1,3]dioxol-5-yl)-2-diazopent-3-enoate (1l)

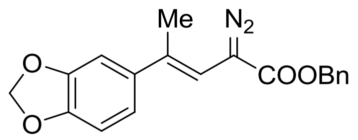

Red oil, TLC R_f = 0.27 (PE/EA = 10:1 v/v), 74% yield. ^{1}H NMR (400 MHz, CDCl$_3$) δ 7.39–7.34 (m, 5H, Ar-H), 6.91 (d, J = 1.7 Hz, 1H, Ar-H), 6.88 (dd, J_1 = 8.0 Hz, J_2 = 1.8 Hz, 1H, Ar-H), 6.77 (d, J = 8.0 Hz, 1H, Ar-H), 5.95 (s, 2H, CH$_2$), 5.88 (d, J = 0.8 Hz, 1H, CH), 5.27 (s, 2H, CH$_2$Ph), 2.03 (d, J = 0.8 Hz, 3H, CH$_3$). ^{13}C NMR (100 MHz, CDCl$_3$) δ 166.4 (1C, C=O), 147.7 (1C, C), 147.0 (1C, Ar-C), 136.4 (1C, Ar-C), 135.8 (1C, Ar-C), 134.6 (1C, Ar-C), 128.6 (2C, Ar-C), 128.3 (1C, Ar-C), 128.2 (1C, Ar-C), 119.4 (2C, Ar-C), 108.0 (1C, Ar-C), 107.8 (1C, Ar-C), 106.4 (1C, CH), 101.1 (1C, CH$_2$), 66.8 (1C, CH$_2$Ph), 17.3 (1C, CH$_3$). IR (neat): 3065w, 3033w, 2955w, 2894w, 2077s (C=N), 1670s, 1503m, 1488m, 1219s, 1101m, 1038m, 804m, 736m, 697m cm^{-1}. HRMS (ESI) Calcd for [$C_{19}H_{16}O_4Na$, M + Na - N$_2$]$^+$: 331.0941, Found: 331.0947.

(*E*)-Benzyl 2-diazo-4-(thiophen-2-yl)pent-3-enoate (1m)

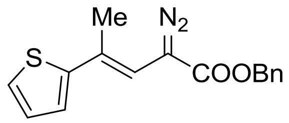

Red oil, TLC R_f = 0.29 (PE/EA = 10:1 v/v), 71% yield. ^{1}H NMR (400 MHz, CDCl$_3$) δ 7.38–7.34 (m, 5H, Ar-H), 7.18 (dd, J_1 = 5.1 Hz, J_2 = 0.9 Hz, 1H, Ar-H), 7.04 (dd, J_1 = 3.6 Hz, J_2 = 1.0 Hz, 1H, Ar-H), 6.98 (dd, J_1 = 5.1 Hz, J_2 = 3.7 Hz, 1H, Ar-H), 6.10 (q, J = 1.0 Hz, 1H, CH), 5.28 (s, 2H, CH$_2$Ph), 2.09 (d, J = 1.0 Hz, 3H, CH$_3$). ^{13}C NMR (100 MHz, CDCl$_3$) δ 166.1 (1C, C=O), 146.4 (1C, C), 135.7 (1C, Ar-C), 128.6 (1C, Ar-C), 128.3 (2C, Ar-C), 128.2 (1C, Ar-C), 128.1 (1C, Ar-C), 127.5 (2C, Ar-C), 124.5 (1C, Ar-C), 122.9 (1C, Ar-C), 107.9 (1C, CH), 66.9 (1C, CH$_2$Ph), 17.1 (1C, CH$_3$). IR (neat): 3107w, 3089w, 3067w, 3033w, 2955w, 2075s (C=N), 1700s, 1497s, 1315m, 1284m, 1209m, 849m, 735m cm^{-1}. HRMS (ESI) Calcd for [$C_{16}H_{14}SO_2Na$, M + Na - N$_2$]$^+$: 293.0607, Found: 293.0605.

Benzyl 2-diazo-4-methylpent-3-enoate (1n)

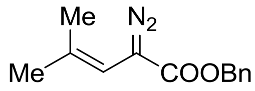

Red oil, TLC R_f = 0.30 (PE/EA = 10:1 v/v), 59% yield. ^{1}H NMR (400 MHz, CDCl$_3$) δ 7.37–7.33 (m, 5H, Ar-H), 5.43 (s, 1H, CH), 5.24 (s, 2H, CH$_2$) 1.86 (s, 3H, CH$_3$), 1.68 (s, 3H, CH$_3$). ^{13}C NMR (100 MHz, CDCl$_3$) δ 166.8 (1C, C=O), 156.1 (1C, C), 136.1 (1C, Ar-C), 128.5 (2C, Ar-C), 128.2 (1C, Ar-C), 128.1 (1C, Ar-C), 118.3 (1C, Ar-C), 106.3 (1C, CH), 66.0 (1C, CH$_2$Ph), 31.0 (1C, CH$_3$), 21.2 (1C, CH$_3$). IR (neat): 3033w, 2075s (C=N), 1700s, 1279s, 1191m, 1048m, 735w, 697w cm^{-1}. HRMS (ESI) Calcd for [$C_{13}H_{14}N_2O_2Na$, M + Na]$^+$: 253.0947, Found: 253.0950.

Benzyl 2-diazobut-3-enoate (1o)

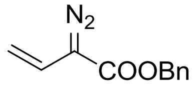

Red oil, TLC R_f = 0.30 (PE/EA = 10:1 v/v), 55% yield. ^{1}H NMR (400 MHz, CDCl$_3$) δ 7.39–7.34 (m, 5H, Ar-H), 6.18 (dd, J_1 = 17.4 Hz, J_2 = 11.0 Hz, 1H, CH), 5.24 (s, 2H, CH$_2$Ph), 5.11 (d, J = 11.0 Hz, 1H, CH$_2$), 4.86 (d, J = 17.4 Hz, 1H, CH$_2$). ^{13}C NMR (100 MHz, CDCl$_3$) δ 164.6 (1C, C=O), 135.7

(1C, Ar-C), 128.6 (2C, Ar-C), 128.3 (1C, Ar-C), 128.1 (2C, Ar-C), 120.3 (1C, CH_2), 107.5 (1C, CH), 66.6 (1C, CH_2Ph). IR (neat): 3033w, 2085s (C=N), 1704s, 1615m, 1379m, 1305s, 1266s, 1133s, 1100m, 743m, 697m cm^{-1}. HRMS (ESI) Calcd for [$C_{11}H_{10}N_2O_2Na$, M + Na]$^+$: 225.0634, Found: 225.0638.

(*E*)-Benzyl 5-(benzyloxy)-2-diazopent-3-enoate (1q)

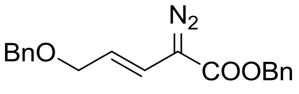

Red oil, TLC R_f = 0.30 (PE/EA = 20:1 v/v), 45% yield. ^{1}H NMR (300 MHz, $CDCl_3$) δ 7.74–7.25 (m, 10H, Ar-H), 6.11 (d, J = 15.9 Hz, 1H, CH=CH), 5.48 (dt, J_1 = 15.9 Hz, J_2 = 1.8 Hz, 1H, CH=CH), 5.25 (s, 2H, CH_2), 4.52 (s, 2H, CH_2), 4.11 (dd, J_1 = 6.3 Hz, J_2 = 1.1 Hz, 2H, CH_2). ^{13}C NMR (100 MHz, $CDCl_3$) δ 164.7 (1C, C=O), 138.0 (1C, Ar-C), 135.6 (1C, Ar-C), 128.5 (1C, CH), 128.3 (2C, Ar-C), 128.1 (2C, Ar-C), 127.7 (2C, Ar-C), 120.1 (2C, Ar-C), 116.4 (2C, CH), 72.0 (1C, CH_2), 70.1 (1C, CH_2), 66.7 (1C, CH_2). HRMS (ESI) Calcd for [$C_{19}H_{19}N_2O_3$, M + H]$^+$: 323.1390, Found: 323.1385.

(*E*)-Methyl 2-diazo-4-phenylpent-3-enoate (1r)

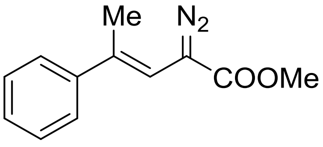

Red oil, TLC R_f = 0.31 (PE/EA = 20:1 v/v), 89% yield. ^{1}H NMR (400 MHz, $CDCl_3$) δ 7.41 (dd, J_1 = 5.5 Hz, J_2 = 3.6 Hz, 2H, Ar-H), 7.35–7.29 (m, 2H, Ar-H), 7.25 (ddd, J_1 = 7.2 Hz, J_2 = 3.7 Hz, J_3 = 1.3 Hz, 1H, Ar-H), 5.98 (d, J = 1.1 Hz, 1H, CH=), 3.82 (s, 3H, OCH_3), 2.08 (d, J = 1.1 Hz, 3H, CH_3). ^{13}C NMR (100 MHz, $CDCl_3$) δ 166.86 (1C, C=O), 142.39 (1C, C), 134.54 (1C, Ar-C), 128.26 (2C, Ar-C), 127.25 (1C, Ar-C), 125.68 (2C, Ar-C), 108.92 (1C, CH), 52.22 (1C, OCH_3), 16.86 (1C, CH_3). IR (neat): 3080w, 3057w, 2998w, 2078s (C=N), 1706s, 1242s, 1115s, 758s, 739s, 698s cm^{-1}. HRMS (ESI) Calcd for [$C_{12}H_{12}O_2Na$, M + Na – N_2]$^+$: 211.0730, Found: 211.0702.

(*E*)-*tert*-Butyl 2-diazo-4-phenylpent-3-enoate (1s)

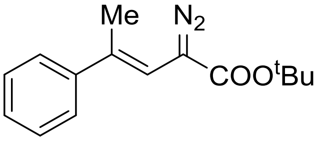

Red oil, TLC R_f = 0.49 (PE/EA = 20:1 v/v), 80% yield. ^{1}H NMR (400 MHz, $CDCl_3$) δ 7.44–7.38 (m, 2H, Ar-H), 7.32 (t, J = 7.5 Hz, 2H, Ar-H), 7.27–7.24 (m, 1H, Ar-H), 5.96 (d, J = 1.0 Hz, 1H, CH=), 2.08 (d, J = 1.1 Hz, 3H, CH_3), 1.52 (s, 9H, $C(CH_3)_3$). ^{13}C NMR (100 MHz, $CDCl_3$) δ 165.78 (1C, C=O), 142.71 (1C, C), 133.64 (1C, Ar-C), 128.29 (2C, Ar-C), 127.12 (1C, Ar-C), 125.72 (2C, Ar-C), 109.62 (1C, Ar-C), 81.99 (1C, O-C), 28.30 (3C, $(CH_3)_3$), 16.88 (1C, CH_3). IR (neat): 3057w, 3027w, 2979w, 2073s (C=N), 1699s, 1247s, 1164s, 1114s, 791s, 739s, 696s cm^{-1}. HRMS (ESI) Calcd for [$C_{15}H_{18}O_2Na$, M + Na – N_2]$^+$: 253.1199, Found: 253.1165.

4. Procedures of N−H Insertions

4.1 The typical procedure

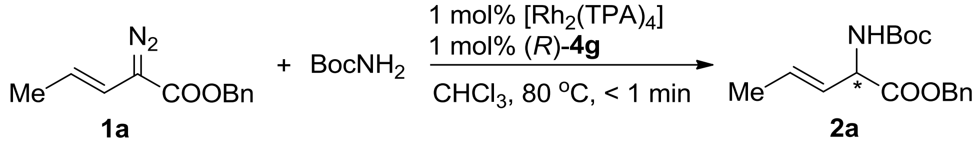

The $Rh_2(TPA)_4$ (2.7 mg, 0.002 mmol, 1 mol%) and (*R*)-**4g** (1.1 mg, 0.002 mmol, 1 mol%) were introduced into an oven-dried Schlenk tube in an argon-filled glovebox. After 2 mL $CHCl_3$ was injected into the Schlenk tube, the mixture was stirred at 80 °C. The mixed solution of (*E*)-benzyl 2-diazopent-3-enoate **1a** (43.2 mg, 0.2 mmol) and *tert*-butyl carbamate (23.4 mg, 0.2 mmol) in 1 mL $CHCl_3$ was added by a syringe in one portion. The reaction finished in 1 min after the addition (TLC monitoring). Then the reaction mixture was concentrated and purified by a flash chromatography on silica gel (PE/EA = 10:1 v/v) to give (*E*)-benzyl 2-(*tert*-butoxycarbonylamino)pent-3-enoate **2a** as a colorless oil. The analysis data for N−H insertion products was listed below.

Table S1: Solvent evaluation [a]

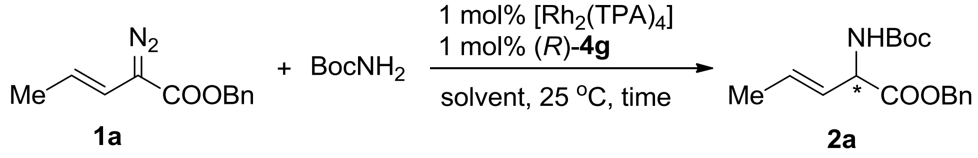

Entry	Solvent	Time	Yield (%)[b]	*ee* (%)[c]
1	$CHCl_3$	< 1 min	74	96
2	DCM	< 1 min	70	92
3	DCE	< 1 min	45	89
4	*c*-hexane	2 h	39	67
5	THF	0.5 h	56	3
6	toluene	2 h	53	6

[a] Reaction conditions: $[Rh_2(TPA)_4]$/(*R*)-**4g**/**1a**/$BocNH_2$ = 0.002:0.002:0.2:0.2 (mmol) in 3 mL solvent at 25 °C. [b] Isolated yield. [c] Determined by HPLC using Chiralcel OD-H column.

4.2 The gram-scale experiment

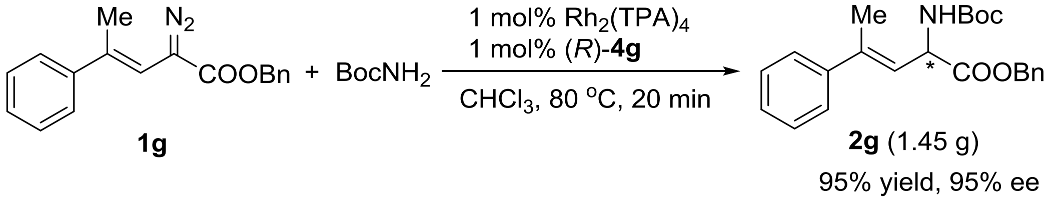

The $Rh_2(TPA)_4$ (36.6 mg, 0.027 mmol, 1 mol%), (*R*)-**4g** (14.8 mg, 0.027 mmol, 1 mol%), and *tert*-butyl carbamate (320 mg, 2.7 mmol) were introduced into an oven-dried three-necked round bottom flask in an argon-filled glovebox. After 13 mL $CHCl_3$ was

injected into the flask, the mixture was stirred at 80 °C. The solution of (*E*)-benzyl 2-diazo-4-phenylpent-3-enoate **1g** (1.2 g, 4.1 mmol) in 27 mL $CHCl_3$ was added to the flask through constant pressure funnel within 2 h. The reaction finished immediately after the addition (TLC monitoring). Then the reaction mixture was concentrated and purified by a flash chromatography on silica gel (PE/EA = 10:1 v/v) to give (*E*)-benzyl 2-(*tert*-butoxycarbonylamino)-4-phenylpent-3-enoate **2g** (1.45 g, 95% yield, 95% ee) as a colorless oil, which slowly solidified with standing.

5. Analytical Data of N−H Insertion Products

(*E*)-Benzyl 2-(*tert*-butoxycarbonylamino)pent-3-enoate (2a)

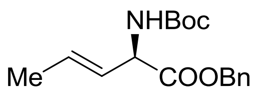

Colorless oil, TLC R_f = 0.3 (PE/EA = 10:1 v/v). 93% yield, 96% ee. [HPLC condition: Chiralcel OD-H column, *n*-hexane/2-propanol = 95:5, flow rate = 1.0 mL/min, wavelength = 220 nm, t_R = 9.74 min for major isomer, t_R = 12.53 min for minor isomer], $[\alpha]_D^{25}$ = –16.6 (*c* 1.0, $CHCl_3$). ^{1}H NMR (400 MHz, $CDCl_3$) δ 7.38–7.31 (m, 5H, Ar-H), 5.80–5.73 (m, 1H, CH=CH), 5.48 (dd, J_1 = 15.2 Hz, J_2 = 5.2 Hz, 1H, CH=CH), 5.22–5.15 (m, 3H, CH and CH_2Ph), 4.82 (brs, 1H, NH), 1.69 (d, *J* = 8.0 Hz, 3H, CH_3), 1.43 (s, 9H, $C(CH_3)_3$). ^{13}C NMR (100 MHz, $CDCl_3$) δ 171.2 (1C, C=O), 154.9 (1C, C=O), 135.3 (1C, Ar-C), 129.6 (1C, CH), 128.5 (1C, Ar-C), 128.2 (1C, Ar-C), 128.0 (1C, Ar-C), 125.3 (1C, CH), 79.9 (1C, C-O), 67.0 (1C, CH_2Ph), 55.4 (1C, CH), 28.2 (3C, $C(CH_3)_3$), 17.6 (1C, CH_3). HRMS (ESI) Calcd for $[C_{17}H_{23}NO_4Na, M + Na]^+$: 328.1519, Found: 328.1521.

(*E*)-Benzyl 2-(*tert*-butoxycarbonylamino)hex-3-enoate (2b)

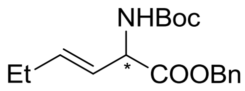

Colorless oil, TLC R_f = 0.25 (PE/EA = 10:1 v/v). 92% yield, 97% ee. [HPLC condition: Chiralcel OD-H column, *n*-hexane/2-propanol = 99:1, flow rate = 1.0 mL/min, wavelength = 210 nm, t_R = 8.92 min for major isomer, t_R = 11.05 min for minor isomer], $[\alpha]_D^{25}$ = –22.0 (*c* 1.0, $CHCl_3$). ^{1}H NMR (400 MHz, $CDCl_3$) δ 7.36–7.26 (m, 5H, Ar-H), 5.83–5.76 (m, 1H, CH=CH), 5.45 (dd, J_1 = 15.6 Hz, J_2 = 5.6 Hz, 1H, CH=CH), 5.24–5.13 (m, 3H, CH and CH_2Ph), 4.83 (brs, 1H, NH), 2.08–2.01 (m, 2H, CH_2), 1.44 (s, 9H, $C(CH_3)_3$), 0.96 (t, *J* = 7.2 Hz, 3H, CH_3). ^{13}C NMR (100 MHz, $CDCl_3$) δ 171.3 (1C, C=O), 154.9 (1C, C=O), 136.3 (1C, Ar-C), 135.4 (1C, CH), 128.5 (2C, Ar-C), 128.3 (1C, Ar-C), 128.1 (2C, Ar-C), 123.1 (1C, CH), 79.9 (1C, C-O), 67.0 (1C, CH_2Ph), 55.3 (1C, CH), 28.3 (3C, $C(CH_3)_3$), 25.2 (1C, CH_2), 13.0 (C, CH_3). HRMS (ESI) Calcd for $[C_{18}H_{25}NO_4Na, M + Na]^+$: 342.1676, Found: 342.1678.

(*E*)-Benzyl 2-(*tert*-butoxycarbonylamino)oct-3-enoate (2c)

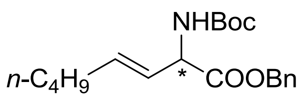

Colorless oil, TLC R_f = 0.3 (PE/EA = 10:1 v/v). 90% yield, 92% ee. [HPLC condition: Chiralcel OD-H column, *n*-hexane/2-propanol = 99:1, flow rate = 1.0 mL/min, wavelength = 210 nm, t_R = 7.57 min for major isomer, t_R = 9.68 min for minor isomer], $[\alpha]_D^{25}$ = –22.8 (*c* 1.0, $CHCl_3$). ^{1}H NMR (400 MHz, $CDCl_3$) δ 7.35–7.32 (m, 5H, Ar-H), 5.78–5.70 (m, 1H, CH=CH), 5.45 (dd, J_1 = 12.0 Hz, J_2 = 8.0 Hz, 1H, CH=CH), 5.24–5.13 (m, 3H, CH and

CH_2Ph), 4.84 (brs, 1H, NH), 2.04–2.03 (m, 2H, CH_2), 1.44 (s, 9H, $C(CH_3)_3$), 1.29–1.26 (m, 4H, $(CH_2)_2$), 0.87 (t, J = 4.0 Hz, 3H, CH_3). ^{13}C NMR (100 MHz, $CDCl_3$) δ 171.2 (1C, C=O), 154.9 (1C, C=O), 135.4 (1C, Ar-C), 135.0 (1C, CH), 128.5 (1C, CH), 128.3 (2C, Ar-C), 128.1 (2C, Ar-C), 124.0 (1C, Ar-C), 79.9 (1C, C-O), 67.0 (1C, CH_2Ph), 55.4 (1C, CH), 31.8 (1C, CH_2), 30.9 (1C, CH_2), 28.2 (3C, $C(CH_3)_3$), 22.0 (1C, CH_2), 13.8 (C, CH_3). HRMS (ESI) Calcd for $[C_{20}H_{29}NO_4Na, M + Na]^+$: 370.1989, Found: 370.1995.

(*E*)-Benzyl 2-(*tert*-butoxycarbonylamino)dec-3-enoate (2d)

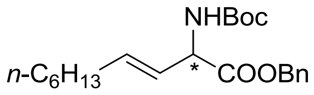

Colorless oil, TLC R_f = 0.3 (PE/EA = 10:1 v/v). 93% yield, 94% ee. [HPLC condition: Chiralcel OD-H column, *n*-hexane/2-propanol = 99:1, flow rate = 1.0 mL/min, wavelength = 210 nm, t_R = 7.76 min for major isomer, t_R = 9.57 min for minor isomer], $[\alpha]_D^{25}$ = –22.4 (*c* 1.0, $CHCl_3$). ^{1}H NMR (400 MHz, $CDCl_3$) δ 7.40–7.28 (m, 5H, Ar-H), 5.80–5.73 (m, 1H, CH=CH), 5.48 (dd, J_1 = 15.2 Hz, J_2 = 5.8 Hz, 1H, CH=CH), 5.23–5.13 (m, 3H, CH and CH_2Ph), 4.85 (brs, 1H, NH), 2.03 (q, J = 7.2 Hz, 2H, CH_2), 1.46 (s, 9H, $C(CH_3)_3$), 1.36–1.24 (m, 8H, $(CH_2)_4$), 0.88 (t, J = 6.8 Hz, 3H, CH_3). ^{13}C NMR (100 MHz, $CDCl_3$) δ 171.2 (1C, C=O), 154.9 (1C, C=O), 135.4 (1C, Ar-C), 135.0 (1C, CH), 128.5 (2C, Ar-C), 128.2 (1C, Ar-C), 128.0 (2C, Ar-C), 123.9 (1C, CH), 79.8 (1C, C-O), 67.0 (1C, CH_2Ph), 55.4 (1C, CH), 32.1 (1C, CH_2), 31.6 (1C, CH_2), 28.7 (2C, CH_2), 28.23 (3C, $C(CH_3)_3$), 22.5 (1C, CH_2), 14.1 (1C, CH_3). HRMS (ESI) Calcd for $[C_{22}H_{33}NO_4Na, M + Na]^+$: 398.2302, Found: 398.2302.

(*E*)-Benzyl 2-(*tert*-butoxycarbonylamino)-5-methylhex-3-enoate (2e)

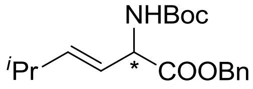

Colorless oil, TLC R_f = 0.35 (PE/EA = 10:1 v/v). 83% yield, 93% ee. [HPLC condition: Chiralcel AD-H column, *n*-hexane/2-propanol = 95:5, flow rate = 1.0 mL/min, wavelength = 210 nm, t_R = 10.85 min for major isomer, t_R = 11.82 min for minor isomer], $[\alpha]_D^{25}$ = –12.4 (*c* 1.0, $CHCl_3$). ^{1}H NMR (400 MHz, $CDCl_3$) δ 7.38–7.26 (m, 5H, Ar-H), 5.74–5.67 (m, 1H, CH=CH), 5.42 (dd, J_1 = 15.2 Hz, J_2 = 5.8 Hz, 1H, CH=CH), 5.24–5.11 (m, 3H, CH and CH_2Ph), 4.82 (brs, 1H, NH), 2.30–2.25 (m, 1H, CH), 1.44 (s, 9H, $C(CH_3)_3$), 0.95 (d, J = 6.7 Hz, 6H, $(CH_3)_2$). ^{13}C NMR (100 MHz, $CDCl_3$) δ 171.4 (1C, C=O), 155.0 (1C, C=O), 141.5 (1C, Ar-C), 135.5 (1C, CH), 128.5 (2C, Ar-C), 128.3 (1C, Ar-C), 128.1 (2C, Ar-C), 121.3 (1C, CH), 79.9 (1C, C-O), 67.0 (1C, CH_2Ph), 55.3 (1C, CH), 30.8 (1C, $CH(CH_3)_2$), 28.3 (3C, $C(CH_3)_3$), 21.9 (1C, $CH(CH_3)_2$). HRMS (ESI) Calcd for $[C_{19}H_{27}NO_4Na, M + Na]^+$: 356.1832, Found: 356.1836.

(*E*)-Benzyl 2-(*tert*-butoxycarbonylamino)-4-phenylbut-3-enoate (2f)

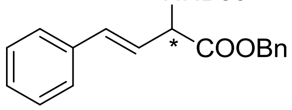

Colorless oil, TLC R_f = 0.3 (PE/EA = 8:1 v/v). 98% yield, 83% ee. [HPLC condition: Chiralcel OD-H column, *n*-hexane/2-propanol = 90:10, flow rate = 1.0 mL/min, wavelength = 254 nm, t_R = 8.33 min for major isomer, t_R = 11.13 min for minor isomer], $[\alpha]_D^{25}$ = –22.0 (*c* 1.0, $CHCl_3$). ^{1}H NMR (400 MHz, $CDCl_3$) δ 7.36–7.26 (m, 10H, Ar-H), 6.63 (d, J = 16.1 Hz, 1H, CH=CH), 6.19 (dd, J_1 = 15.9 Hz, J_2 = 6.1 Hz, 1H, CH=CH), 5.29 (d, J = 7.0 Hz, 1H, CH), 5.22 (s, 2H, CH_2Ph), 5.01 (brs, 1H, NH), 1.46 (s, 9H, $C(CH_3)_3$). ^{13}C NMR (100 MHz, $CDCl_3$) δ 170.8 (1C, C=O), 154.9 (1C, C=O), 135.9 (1C,

Ar-C), 135.2 (1C, Ar-C), 132.9 (1C, CH), 128.6 (2C, Ar-C), 128.5 (2C, Ar-C), 128.2 (2C, Ar-C), 128.1 (2C, Ar-C), 126.6 (2C, Ar-C), 123.8 (1C, CH), 80.2 (1C, C-O), 67.4 (1C, CH_2Ph), 55.5 (1C, CH), 28.3 (3C, $C(CH_3)_3$). HRMS (ESI) Calcd for [$C_{22}H_{25}NO_4Na$, M + Na]$^+$: 390.1676, Found: 390.1678.

(*E*)-Benzyl 2-(*tert*-butoxycarbonylamino)-4-phenylpent-3-enoate (2g)

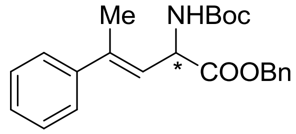

Colorless oil, TLC R_f = 0.29 (PE/EA = 8:1 v/v). 99% yield, 96% ee. [HPLC condition: Chiralcel OD-H column, *n*-hexane/2-propanol = 90:10, flow rate = 1.0 mL/min, wavelength = 254 nm, t_R = 6.54 min for major isomer, t_R = 9.72 min for minor isomer], $[\alpha]_D^{25}$ = –62.0 (*c* 1.0, $CHCl_3$). ^1H NMR (400 MHz, $CDCl_3$) δ 7.32–7.26 (m, 10H, Ar-H), 5.53–5.52 (m, 1H, CH=), 5.26–5.13 (m, 4H, CH and NH and CH_2Ph), 2.20 (s, 3H, CH_3), 1.45 (s, 9H, $C(CH_3)_3$). ^{13}C NMR (100 MHz, $CDCl_3$) δ 171.3 (1C, C=O), 154.9 (1C, C=O), 142.3 (1C, Ar-C), 141.6 (1C, C), 135.5 (1C, Ar-C), 128.5 (2C, Ar-C), 128.2 (3C, Ar-C), 128.2 (1C, Ar-C), 127.8 (1C, Ar-C), 127.6 (1C, Ar-C), 125.9 (2C, Ar-C), 121.9 (1C, CH), 80.0 (1C, C-O), 67.0 (1C, CH_2Ph), 52.9 (1C, CH), 28.2 (3C, $C(CH_3)_3$), 16.9(1C, CH_3). HRMS (ESI) Calcd for [$C_{23}H_{27}NO_4Na$, M + Na]$^+$: 404.1832, Found: 404.1836.

(*E*)-Benzyl 2-(*tert*-butoxycarbonylamino)-4-(4-chlorophenyl)pent-3-enoate (2h)

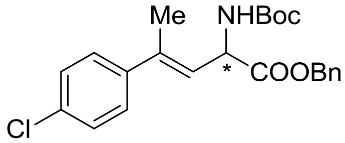

Colorless oil, TLC R_f = 0.25 (PE/EA = 8:1 v/v). 96% yield, 93% ee. [HPLC condition: Chiralcel OD-H column, *n*-hexane/2-propanol = 90:10, flow rate = 1.0 mL/min, wavelength = 254 nm, t_R = 6.92 min for major isomer, t_R = 9.82 min for minor isomer], $[\alpha]_D^{25}$ = –56.2 (*c* 1.0, $CHCl_3$). ^1H NMR (400 MHz, $CDCl_3$) δ 7.23–7.14 (m, 9H, Ar-H), 5.44 (dd, J_1 = 8.8 Hz, J_2 = 1.2 Hz, 1H, CH), 5.18–5.05 (m, 4H, CH and NH and CH_2Ph), 2.09 (s, 3H, CH_3), 1.36 (s, 9H, $C(CH_3)_3$). ^{13}C NMR (100 MHz, $CDCl_3$) δ 171.2 (1C, C=O), 154.9 (1C, C=O), 140.7 (1C, Ar-C), 140.4 (1C, Ar-C), 135.4 (1C, Ar-C), 133.4 (1C, C), 128.5 (3C, Ar-C), 128.4 (2C, Ar-C), 128.3 (2C, Ar-C), 127.9 (2C, Ar-C), 122.5 (1C, CH), 80.1 (1C, C-O), 67.1 (1C, CH_2Ph), 52.9 (1C, CH), 28.3 (3C, $C(CH_3)_3$), 16.9 (1C, CH_3). HRMS (ESI) Calcd for [$C_{23}H_{26}ClNO_4Na$, M + Na]$^+$: 438.1443, Found: 438.1442.

(*E*)-Benzyl 2-(*tert*-butoxycarbonylamino)-4-(4-fluorophenyl)pent-3-enoate (2i)

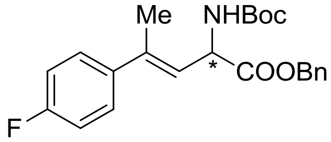

Colorless oil, TLC R_f = 0.27 (PE/EA = 10:1 v/v). 99% yield, 97% ee. [HPLC condition: Chiralcel OD-H column, *n*-hexane/2-propanol = 90:10, flow rate = 1.0 mL/min, wavelength = 254 nm, t_R = 6.21 min for major isomer, t_R = 8.33 min for minor isomer], $[\alpha]_D^{25}$ = –62.4 (*c* 1.0, $CHCl_3$). ^1H NMR (400 MHz, $CDCl_3$) δ 7.36–7.29 (m, 7H, Ar-H), 7.03 (t, *J* = 8.6 Hz, 2H, Ar-H), 5.55 (d, *J* = 9.0 Hz, 1H, CH), 5.40–5.13 (m, 4H, CH and NH and CH_2Ph), 2.22 (s, 3H, CH_3), 1.49 (s, 9H, $C(CH_3)_3$). ^{13}C NMR (100 MHz, $CDCl_3$) δ 171.2 (1C, C=O), 163.5 (1C, C=O), 161.1 (1C, Ar-C), 154.9 (1C, Ar-C), 140.5 (1C, Ar-C), 138.3 (1C, C), 135.4 (1C, Ar-C), 128.5 (2C, Ar-C), 128.2 (1C, Ar-C), 127.8 (2C, Ar-C), 127.5 (1C, Ar-C), 127.4 (1C, Ar-C), 121.9 (1C, Ar-C), 115.1 (1C, Ar-C), 114.9 (1C, C), 80.0 (1C, C-O), 67.0 (1C, CH_2Ph), 52.9 (1C, CH), 28.2 (3C, $C(CH_3)_3$), 17.0 (1C, CH_3). HRMS (ESI) Calcd for [$C_{23}H_{26}FNO_4Na$, M + Na]$^+$:

422.1738, Found: 422.1741.

(*E*)-Benzyl 2-(*tert*-butoxycarbonylamino)-4-(4-methoxyphenyl)pent-3-enoate(2j)

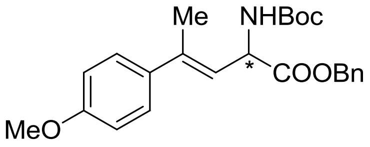

Colorless oil, TLC R_f = 0.26 (PE/EA = 8:1 v/v). 97% yield, 94% ee. [HPLC condition: Chiralcel OD-H column, *n*-hexane/2-propanol = 90:10, flow rate = 1.0 mL/min, wavelength = 254 nm, t_R = 8.96 min for major isomer, t_R = 12.15 min for minor isomer], $[\alpha]_D^{25}$ = –64.0 (*c* 1.0, $CHCl_3$). ^{1}H NMR (400 MHz, $CDCl_3$) δ 7.35–7.29 (m, 7H, Ar-H), 6.87 (d, J = 8.7 Hz, 2H, Ar-H), 5.52 (dd, J_1 = 8.8 Hz, J_2 = 1.1 Hz, 1H, CH), 5.29–5.15 (m, 4H, CH and NH and CH_2Ph), 3.83 (s, 3H, OCH_3), 2.20 (s, 3H, CH_3), 1.48 (s, 9H, $C(CH_3)_3$). ^{13}C NMR (100 MHz, $CDCl_3$) δ 171.5 (1C, C=O), 159.2 (1C, C=O), 155.0 (1C, Ar-C), 141.0 (1C, Ar-C), 135.5 (1C, Ar-C), 134.6 (1C, C), 128.5 (2C, Ar-C), 128.1 (2C, Ar-C), 127.8 (2C, Ar-C), 127.0 (2C, Ar-C), 120.2 (1C, Ar-C), 113.6 (1C, CH), 79.9 (1C, C-O), 66.9 (1C, CH_2Ph), 55.2 (1C, CH), 53.0 (1C, OCH_3), 28.3 (3C, $C(CH_3)_3$), 16.9 (1C, CH_3). HRMS (ESI) Calcd for $[C_{24}H_{29}NO_5Na, M + Na]^+$: 434.1938, Found: 434.1936.

(*E*)-Benzyl 2-(*tert*-butoxycarbonylamino)-4-(naphthalen-2-yl)pent-3-enoate (2k)

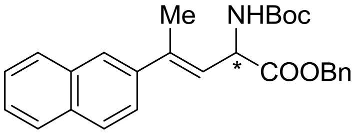

Colorless oil, TLC R_f = 0.30 (PE/EA = 10:1 v/v). 99% yield, 90% ee. [HPLC condition: Chiralcel OD-H column, *n*-hexane/2-propanol = 90:10, flow rate = 1.0 mL/min, wavelength = 210 nm, t_R = 9.35 min for major isomer, t_R = 10.68 min for minor isomer], $[\alpha]_D^{25}$ = –68.8 (*c* 1.0, $CHCl_3$). ^{1}H NMR (400 MHz, $CDCl_3$) δ 7.75–7.60 (m, 4H，Ar-H), 7.40–7.30 (m, 3H, Ar-H), 7.25–7.10 (m, 5H, Ar-H), 5.60 (d, J = 8.7 Hz, 1H, CH), 5.24–5.03 (m, 4H, CH and NH and CH_2Ph), 2.20 (s, 3H, CH_3), 1.36 (s, 9H, $C(CH_3)_3$). ^{13}C NMR (100 MHz, $CDCl_3$) δ 171.3 (1C, C=O), 155.0 (1C, C=O), 135.5 (1C, C), 133.2 (2C, Ar-C), 132.8 (1C, Ar-C), 128.5 (2C, Ar-C), 128.2 (1C, Ar-C), 128.1 (1C, Ar-C), 127.9 (2C, Ar-C), 127.8 (1C, Ar-C), 127.5 (2C, Ar-C), 126.2 (1C, Ar-C), 126.0 (1C, Ar-C), 124.8 (1C, Ar-C), 124.1 (1C, Ar-C), 122.4 (1C, CH), 80.1 (1C, C-O), 67.0 (1C, CH_2Ph), 53.0 (1C, CH), 28.3 (3C, $C(CH_3)_3$), 16.9 (1C, CH_3). HRMS (ESI) Calcd for $[C_{27}H_{29}NO_4Na, M + Na]^+$: 454.1989, Found: 454.1980.

(*E*)-Benzyl-4-(benzo[d][1,3]dioxol-5-yl)-2-(*tert*-butoxycarbonylamino)pent-3-enoate (2l)

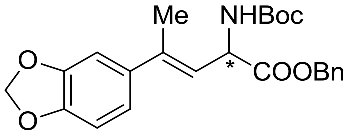

Colorless oil, TLC R_f = 0.26 (PE/EA = 8:1 v/v). 98% yield, 92% ee. [HPLC condition: Chiralcel OD-H column, *n*-hexane/2-propanol = 90:10, flow rate = 1.0 mL/min, wavelength = 220 nm, t_R = 9.05 min for major isomer, t_R = 11.40 min for minor isomer], $[\alpha]_D^{25}$ = –54.4 (*c* 1.0, $CHCl_3$). ^{1}H NMR (400 MHz, $CDCl_3$) δ 7.36–7.29 (m, 5H, Ar-H), 6.85–6.83 (m, 2H, Ar-H), 6.78 (d, J = 8.5 Hz, 1H, Ar-H), 5.98 (s, 2H, CH_2), 5.45 (d, J = 8.5 Hz, 1H, CH), 5.30–5.16 (m, 4H, CH and NH and CH_2Ph), 2.18 (s, 3H, CH_3), 1.48 (s, 9H, $C(CH_3)_3$). ^{13}C NMR (100 MHz, $CDCl_3$) δ 171.4 (1C, C=O), 155.0 (1C, C=O), 147.6 (1C, Ar-C), 147.2 (1C, Ar-C), 136.6 (1C, Ar-C), 135.5 (1C, Ar-C), 128.5 (2C, C), 128.2 (1C, Ar-C), 127.9 (2C, Ar-C), 121.0 (1C, Ar-C), 119.5 (1C, Ar-C), 108.0 (1C, Ar-C), 106.5 (1C, CH), 101.1 (1C, CH_2), 80.0 (1C, C-O), 67.0 (1C, CH_2Ph),

52.9 (1C, CH), 28.3 (3C, $C(CH_3)_3$), 17.1 (1C, CH_3). HRMS (ESI) Calcd for [$C_{24}H_{27}NO_6Na$, M + Na]$^+$: 448.1731, Found: 448.1733.

(*E*)-Benzyl 2-(*tert*-butoxycarbonylamino)-4-(thiophen-2-yl)pent-3-enoate (2m)

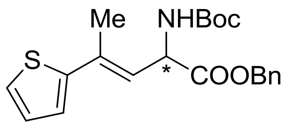

Colorless oil, TLC R_f = 0.28 (PE/EA = 10:1 v/v). 99% yield, 97% ee. [HPLC condition: Chiralcel OD-H column, *n*-hexane/2-propanol = 90:10, flow rate = 1.0 mL/min, wavelength = 273 nm, t_R = 7.11 min for major isomer, t_R = 8.75 min for minor isomer], $[\alpha]_D^{25}$ = –60.4 (*c* 1.0, $CHCl_3$). ^{1}H NMR (400 MHz, $CDCl_3$) δ 7.32–7.25 (m, 5H, Ar-H), 7.17 (d, J = 4.8 Hz, 1H, Ar-H), 7.05 (dd, J_1 = 3.6 Hz, J_2 = 0.8 Hz, 1H, Ar-H), 6.97 (dd, J_1 = 5.0 Hz, J_2 = 3.7 Hz, 1H, Ar-H), 5.70 (dd, J_1 = 8.8 Hz, J_2 = 1.1 Hz, 1H, CH), 5.23–5.17 (m, 4H, CH and NH and CH_2Ph), 2.21 (s, 3H, CH_3), 1.44 (s, 9H, $C(CH_3)_3$). ^{13}C NMR (100 MHz, $CDCl_3$) δ 171.2 (1C, C=O), 154.9 (1C, C=O), 145.8 (1C, Ar-C), 135.4 (1C, Ar-C), 135.0 (1C, C), 128.5 (1C, Ar-C), 128.2 (2C, Ar-C), 127.8 (1C, Ar-C), 127.4 (2C, Ar-C), 124.5 (1C, Ar-C), 124.0 (1C, Ar-C), 120.1 (1C, CH), 80.1 (1C, C-O), 67.1 (1C, CH_2Ph), 52.6 (1C, CH), 28.3 (3C, $C(CH_3)_3$), 16.7 (1C, CH_3). HRMS (ESI) Calcd for [$C_{21}H_{25}NSO_4Na$, M + Na]$^+$: 410.1397, Found: 410.1398.

Benzyl 2-(*tert*-butoxycarbonylamino)-4-methylpent-3-enoate (2n)

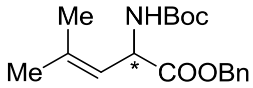

Colorless oil, TLC R_f = 0.3 (PE/EA = 10:1 v/v). 66% yield, 98% ee. [HPLC condition: Chiralcel OD-H column, *n*-hexane/2-propanol = 90:10, flow rate = 1.0 mL/min, wavelength = 210 nm, t_R = 4.84 min for major isomer, t_R = 5.92 min for minor isomer], $[\alpha]_D^{25}$ = –126.9 (*c* 1.0, $CHCl_3$). ^{1}H NMR (400 MHz, $CDCl_3$) δ 7.37–7.26 (m, 5H, Ar-H), 5.22–5.01 (m, 5H, CH and NH and CH_2Ph), 1.78 (s, 3H, CH_3), 1.73 (s, 3H, CH_3), 1.43 (s, 9H, $C(CH_3)_3$). ^{13}C NMR (100 MHz, $CDCl_3$). δ 171.9 (1C, C=O), 155.0 (1C, C=O), 139.7 (1C, Ar-C), 135.6 (1C, C), 128.5 (2C, Ar-C), 128.2 (1C, Ar-C), 127.8 (2C, Ar-C), 119.2 (1C, CH), 79.9 (1C, C-O), 66.8 (1C, CH_2Ph), 52.6 (1C, CH), 28.3 (3C, $C(CH_3)_3$), 25.7 (1C, CH_3), 18.7 (1C, CH_3). HRMS (ESI) Calcd for [$C_{18}H_{25}NO_4Na$, M + Na]$^+$: 342.1676, Found: 342.1676.

Benzyl 2-(*tert*-butoxycarbonylamino)but-3-enoate (2o)

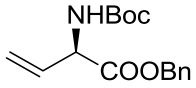

Colorless oil, TLC R_f = 0.35 (PE/EA = 10:1 v/v). 88% yield, 90% ee. [HPLC condition: Chiralpak AD-H column, *n*-hexane/2-propanol = 90:10, flow rate = 1.0 mL/min, wavelength = 210 nm, t_R = 8.74 min for major isomer, t_R = 10.40 min for minor isomer], $[\alpha]_D^{25}$ = 8.0 (*c* 1.0, $CHCl_3$). ^{1}H NMR (400 MHz, $CDCl_3$) δ 7.37–7.33 (m, 5H, Ar-H), 5.96–5.88 (m, 1H, CH), 5.37–5.15 (m, 5H, CH_2 and CH and CH_2Ph), 4.92 (brs, 1H, NH), 1.45 (s, 9H, $C(CH_3)_3$). ^{13}C NMR (100 MHz, $CDCl_3$) δ 170.6 (1C, C=O), 155.0 (1C, C=O), 135.2 (1C, Ar-C), 132.5 (1C, CH), 128.6 (2C, Ar-C), 128.4 (1C, Ar-C), 128.1 (2C, Ar-C), 117.4 (1C, CH_2), 80.1 (1C, C-O), 67.3 (1C, CH_2Ph), 55.8 (1C, CH), 28.3 (3C, $C(CH_3)_3$). HRMS (ESI) Calcd for [$C_{16}H_{21}NO_4Na$, M + Na]$^+$: 314.1363, Found: 314.1365.

Ethyl 2-(*tert*-butoxycarbonylamino)-2-cyclohexenylacetate (2p)

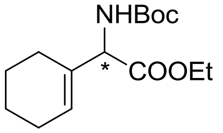

Colorless oil, TLC R_f = 0.30 (PE/EA = 8:1 v/v). 61% yield, 94% ee. [HPLC condition: Chiralcel OD-H column, *n*-hexane/2-propanol = 99:1, flow rate = 1.0 mL/min, wavelength = 210 nm, t_R = 4.94 min for major isomer, t_R = 5.34 min for minor isomer], $[\alpha]_D^{25}$ = –53.0 (*c* 1.0, $CHCl_3$). ^1H NMR (400 MHz, $CDCl_3$) δ 5.73 (s, 1H, CH), 5.22 (d, *J* = 5.8 Hz, 1H, CH), 4.63 (d, *J* = 7.6 Hz, 1H, CH), 4.24–4.17 (m, 2H, CH_2), 2.05–1.94 (m, 4H, $(CH_2)_2$), 1.65–1.54 (m, 4H, $(CH_2)_2$), 1.44 (s, 9H, $C(CH_3)_3$), 1.27 (t, *J* = 7.1 Hz, 3H, CH_3). ^{13}C NMR (100 MHz, $CDCl_3$) δ 171.4 (1C, C=O), 155.0 (1C, C=O), 133.3 (1C, C), 126.3 (1C, CH), 79.8 (1C, C-O), 61.3 (1C, CH_2), 59.3 (1C, CH), 28.3 (3C, $C(CH_3)_3$), 25.4 (1C, CH_2), 25.1 (1C, CH_2), 22.5 (1C, CH_2), 21.9 (1C, CH_2), 14.1 (1C, CH_3). HRMS (ESI) Calcd for $[C_{15}H_{25}NO_4Na, M + Na]^+$: 306.1676, Found: 306.1680.

(*E*)-Benzyl 5-(benzyloxy)-2-(*tert*-butoxycarbonylamino)pent-3-enoate (2q)

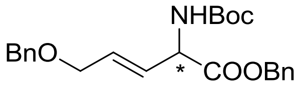

Colorless oil, TLC R_f = 0.35 (PE/EA = 10:1 v/v). 90% yield, 87% ee [using (*S*)-**4g**]. [HPLC condition: Chiralcel OD-H column, *n*-hexane/2-propanol = 90:10, flow rate = 1.0 mL/min, wavelength = 210 nm, t_R = 19.19 min for major isomer, t_R = 25.53 min for minor isomer], $[\alpha]_D^{25}$ = –11.4 (*c* 1.0, $CHCl_3$). ^1H NMR (400 MHz, $CDCl_3$) δ 7.24–7.21 (m, 10H, Ar-H), 5.77–5.74 (m, 2H, CH_2), 5.19–5.17 (m, 1H, CH), 5.09 (s, 2H, CH_2), 4.85 (brs, 1H, NH), 4.37 (s, 1H, CH), 3.95–3.80 (m, 2H, CH_2), 1.35 (s, 9H, $C(CH_3)_3$). ^{13}C NMR (100 MHz, $CDCl_3$) δ 170.6 (1C, C=O), 154.9 (1C, C=O), 137.9 (1C, Ar-C), 135.1 (1C, Ar-C), 129.9 (1C, CH), 128.5 (2C, Ar-C), 128.3 (2C, Ar-C), 128.1 (2C, Ar-C), 127.7 (2C, Ar-C), 127.6 (2C, Ar-C), 126.7 (1C, CH), 80.0 (1C, C-O), 72.0 (1C, CH_2), 69.3 (1C, CH_2), 67.2 (1C, CH_2), 54.9 (1C, CH), 28.2 (3C, $C(CH_3)_3$). IR (neat): 3088w, 2859w, 2085s (C=N), 1722s, 1496m, 1286s, 1165m, 973w, 737m, 697m cm^{-1}. HRMS (ESI) Calcd for $[C_{24}H_{33}N_2O_5, M + NH_4]^+$: 429.2384, Found: 429.2388.

(*E*)-Methyl 2-(*tert*-butoxycarbonylamino)-4-phenylpent-3-enoate (2r)

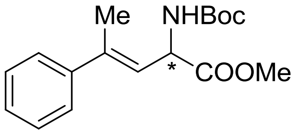

Colorless oil, TLC R_f = 0.59 (PE/EA = 5:1 v/v). 99% yield, 93% ee. [HPLC condition: Chiralcel OD-H column, *n*-hexane/2-propanol = 90:10, flow rate = 1.0 mL/min, wavelength = 254 nm, t_R = 5.37 min for major isomer, t_R = 6.09 min for minor isomer], $[\alpha]_D^{27}$ = –146.8 (*c* 1.0, $CHCl_3$). ^1H NMR (400 MHz, $CDCl_3$) δ 7.41–7.24 (m, 5H, Ar-H), 5.57 (d, *J* = 8.4 Hz, 1H, CH=), 5.28 (s, 1H, NH), 5.21–5.06 (m, 1H, CH), 3.74 (s, 3H, OMe), 2.23 (s, 3H, CH_3), 1.45 (s, 9H, $C(CH_3)_3$). ^{13}C NMR (100 MHz, $CDCl_3$) δ 172.14 (1C, C=O), 154.93 (1C, C=O), 142.14 (1C, C), 141.42 (1C, Ar-C), 128.25 (2C, Ar-C), 127.66 (1C, Ar-C), 125.86 (2C, Ar-C), 121.82 (1C, CH), 79.99 (1C, C-O), 52.66 (1C, C-O), 52.50 (1C, C-N), 28.26 (3C, $C(CH_3)_3$), 16.70 (1C, CH_3). HRMS (ESI) Calcd for $[C_{17}H_{23}NO_4Na, M + Na]^+$: 328.1519, Found: 328.1518.

(*E*)-*tert*-Butyl 2-(*tert*-butoxycarbonylamino)-4-phenylpent-3-enoate (2s)

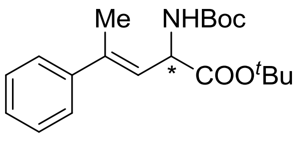

White solid, Mp: 100–102 °C. TLC R_f = 0.78 (PE/EA = 5:1 v/v). 99% yield, 97% ee. [HPLC condition: Chiralcel OD-H column, *n*-hexane/2-propanol = 90:10, flow rate = 1.0 mL/min, wavelength = 254 nm, t_R = 3.79 min for major isomer, t_R = 4.23

min for minor isomer], $[\alpha]_D^{27}$ = –126.6 (*c* 1.0, $CHCl_3$). ^{1}H NMR (400 MHz, $CDCl_3$) δ 7.31 (ddd, *J* = 25.7, 12.6, 5.4 Hz, 5H, Ar-H), 5.52 (dd, *J* = 9.0, 1.1 Hz, 1H, CH=), 5.27 (d, *J* = 6.3 Hz, 1H, NH), 5.05 (t, *J* = 7.9 Hz, 1H, CH), 2.23 (s, 3H, CH_3), 1.45 (d, *J* = 1.2 Hz, 18H, $2C(CH_3)_3$). ^{13}C NMR (100 MHz, $CDCl_3$) δ 170.58 (1C, C=O), 154.95 (1C, C=O), 142.62 (1C, Ar-C), 140.68 (1C, C), 128.25 (2C, Ar-C), 127.49 (1C, Ar-C), 125.90 (2C, Ar-C), 123.11 (1C, CH), 82.01 (1C, C-N), 79.71 (1C, C-O), 53.47 (1C, C-O), 28.30 (3C, $C(CH_3)_3$), 27.93 (3C, $C(CH_3)_3$), 16.88 (1C, CH_3). HRMS (ESI) Calcd for $[C_{20}H_{29}NO_4Na, M + Na]^+$: 370.1989, Found: 370.1983.

6. Transformations of N−H Insertion Products

6.1 Synthesis of (*S*)-vinylglycine (5)[5]

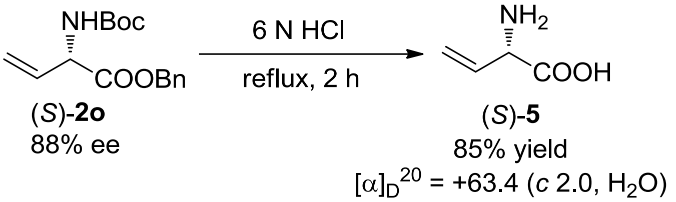

Compound (*S*)-**2o** (118 mg, 0.4 mmol) was dissolved in 8 mL 6 N HCl. The resulting solution was heated to reflux for 2 h and then evaporated under reduced pressure at 30 °C. The residue was dissolved in the minimum amount of water and applied to a column of Dowex 50W-X8 ion exchange resin. The column was washed with water and eluted with 4% aqueous pyridine. The eluate was evaporated under reduced pressure to give product (*S*)-**5** with 85% yield as a white solid. Mp: 195–197 °C (dec.); $[\alpha]_D^{26}$ = +63.4 (*c* 2.0, H_2O); ^{1}H NMR (400 MHz, D_2O) *δ* 5.99–5.95 (m, 1H, CH), 5.50–5.46 (m, 2H, CH_2), 4.27 (d, *J* = 7.6 Hz, 1H, CH). ^{13}C NMR (100 MHz, $CDCl_3$) 133.1 (1C, CH=), 119.5 (1C, CH_2=), 58.5 (1C, CH).

6.2 Synthesis of (*R*)-2-aminopentanoic acid (6)[6]

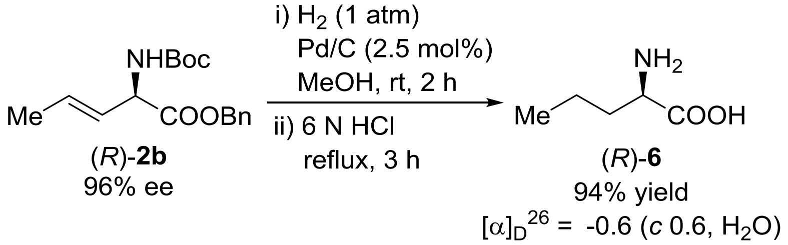

A suspension of (*R*)-**2b** (127 mg, 0.4 mmol) and Pd/C (11 mg, 2.5 mol%) in 4 mL MeOH was stired under hydrogen atmosphere for 2 h. The mixture was concentrated under vacuum and then 8 mL of 6 N HCl was added. The resulting solution was heated to reflux for 3 h and then evaporated under reduced pressure at 30 °C. The residue was dissolved in the minimum amount of water and applied to a column of Dowex 50W-X8 ion exchange resin. The column was washed with water and eluted with 4% aqueous pyridine. The eluate was evaporated under reduced pressure to give product (*R*)-**6** with 94% yield as a white solid. $[\alpha]_D^{26}$ = –0.6 (*c* 0.6, H_2O); ^{1}H NMR (400 MHz, D_2O) *δ* 3.73

(t, *J* = 6.0 Hz, 1H, CH), 1.91–1.75 (m, 2H, CH_2), 1.45–1.30 (m, 2H, CH_2), 0.93 (t, *J* = 7.6 Hz, 3H, CH_3). ^{13}C NMR (100 MHz, $CDCl_3$): 174.9 (1C, C=O), 56.0 (1C, CH), 34.4 (1C, CH_2), 19.6 (1C, CH_2), 14.2 (1C, CH_3).

7. NMR Spectra of New Compounds

(*R*)-6,6'-dimesityl-7,7'-bis(methoxymethoxy)-1,1'-spirobiindane [(*R*)-8]

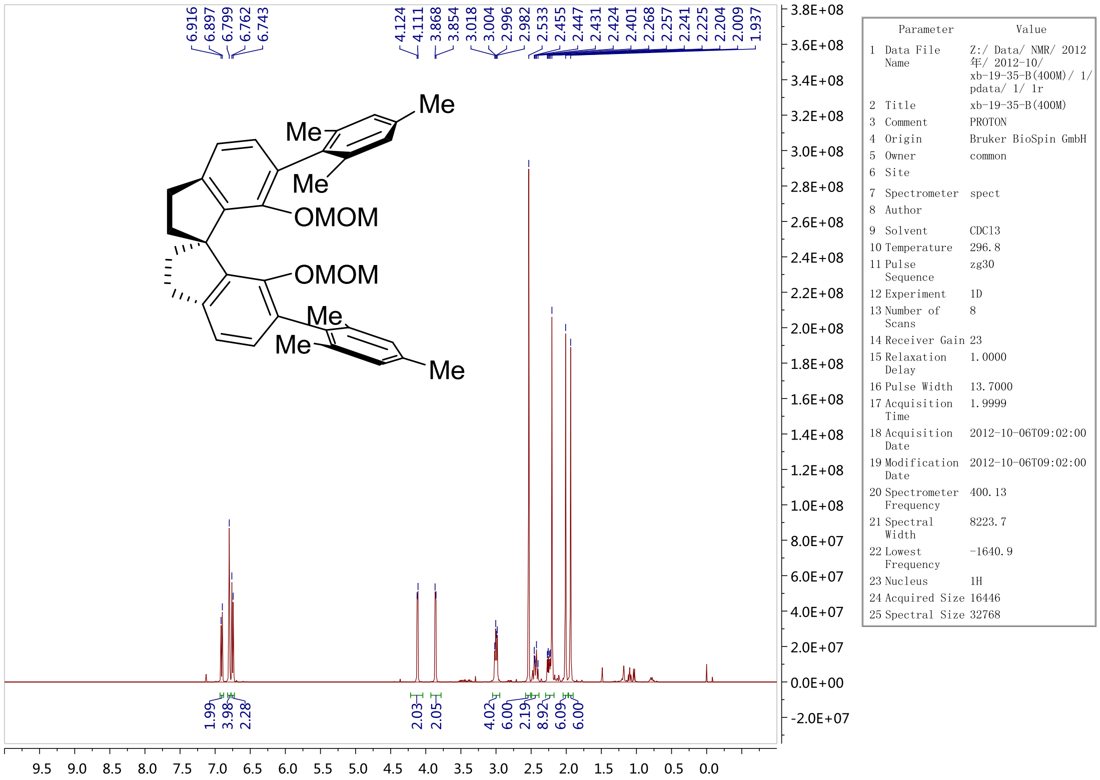

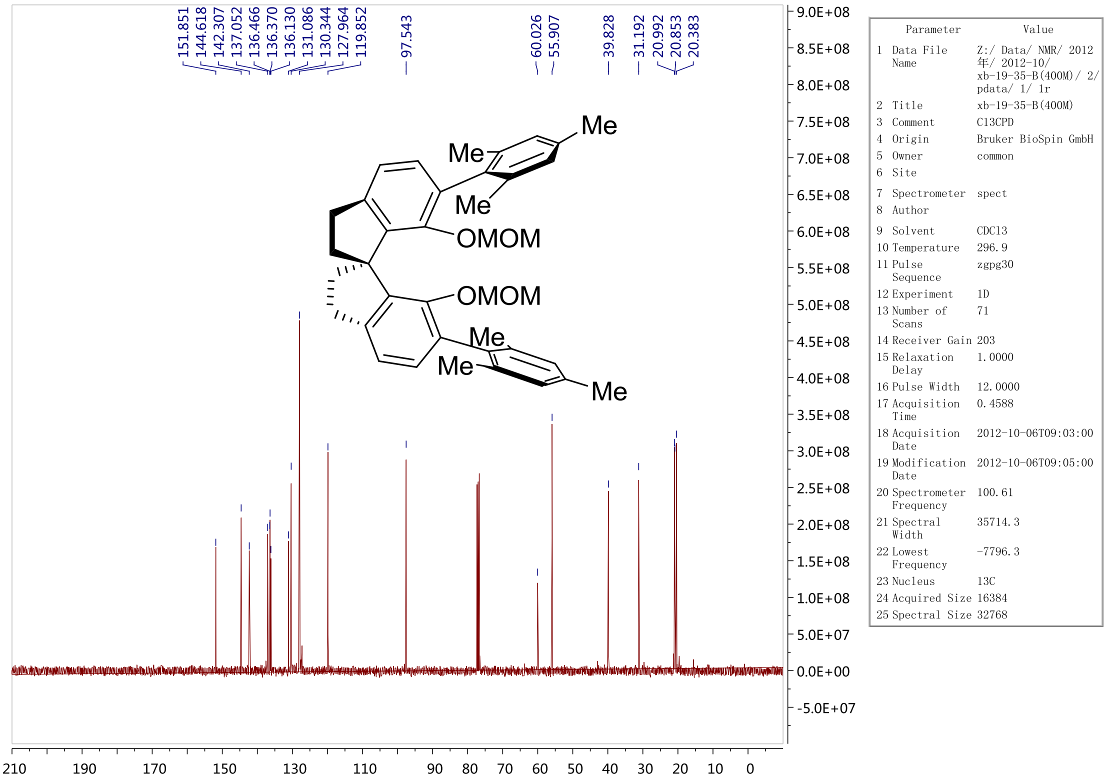

(*R*)-6,6'-dimesityl- 1,1'-spirobiindane-7,7'-diol [(*R*)-9]

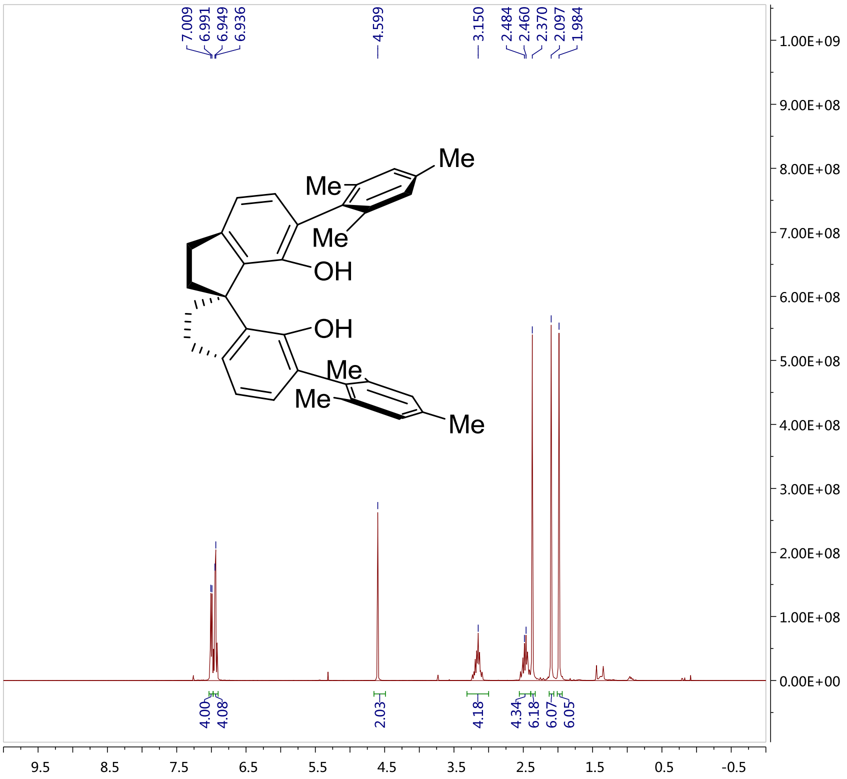

Parameter		Value
1	Data File Name	Z:/ Personal/ 郭军霞/ 磷酸文献/ GJX-10-40-Me-OH/ 1/ pdata/ 1/ 1r
2	Title	xb-19-38
3	Comment	PROTON
4	Origin	Bruker BioSpin GmbH
5	Owner	common
6	Site	
7	Spectrometer	spect
8	Author	
9	Solvent	CDCl3
10	Temperature	291.9
11	Pulse Sequence	zg30
12	Experiment	1D
13	Number of Scans	8
14	Receiver Gain	36
15	Relaxation Delay	1.0000
16	Pulse Width	13.5000
17	Acquisition Time	2.9999
18	Acquisition Date	2012-10-10T08:30:00
19	Modification Date	2012-10-10T08:30:00
20	Spectrometer Frequency	400.13
21	Spectral Width	8223.7
22	Lowest Frequency	-1685.4
23	Nucleus	1H
24	Acquired Size	24670
25	Spectral Size	32768

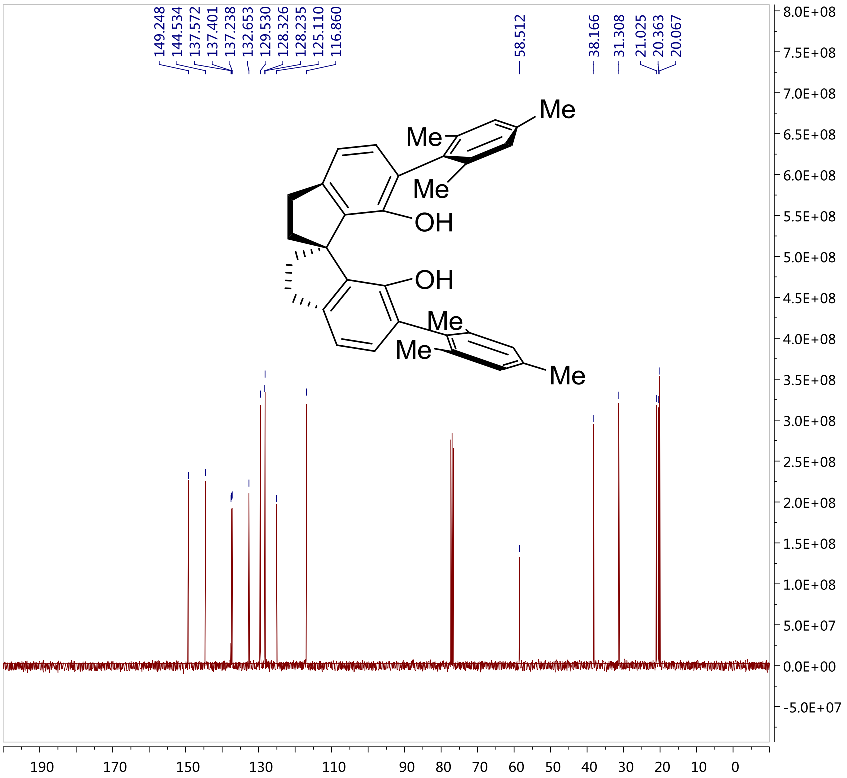

Parameter		Value
1	Data File Name	Z:/ Personal/ 郭军霞/ 磷酸文献/ GJX-10-40-Me-OH/ 2/ pdata/ 1/ 1r
2	Title	xb-19-38
3	Comment	C13CPD
4	Origin	Bruker BioSpin GmbH
5	Owner	common
6	Site	
7	Spectrometer	spect
8	Author	
9	Solvent	CDCl3
10	Temperature	292.3
11	Pulse Sequence	zgpg30
12	Experiment	1D
13	Number of Scans	73
14	Receiver Gain	203
15	Relaxation Delay	1.0000
16	Pulse Width	12.5000
17	Acquisition Time	0.5506
18	Acquisition Date	2012-10-10T08:31:00
19	Modification Date	2012-10-10T08:33:00
20	Spectrometer Frequency	100.61
21	Spectral Width	29761.9
22	Lowest Frequency	-4820.2
23	Nucleus	13C
24	Acquired Size	16384
25	Spectral Size	32768

(*R*)-6,6'-Dimesityl-1,1'-spirobiindanyl-7,7'-diyl-hydrogenphosphate [(*R*)-4g]

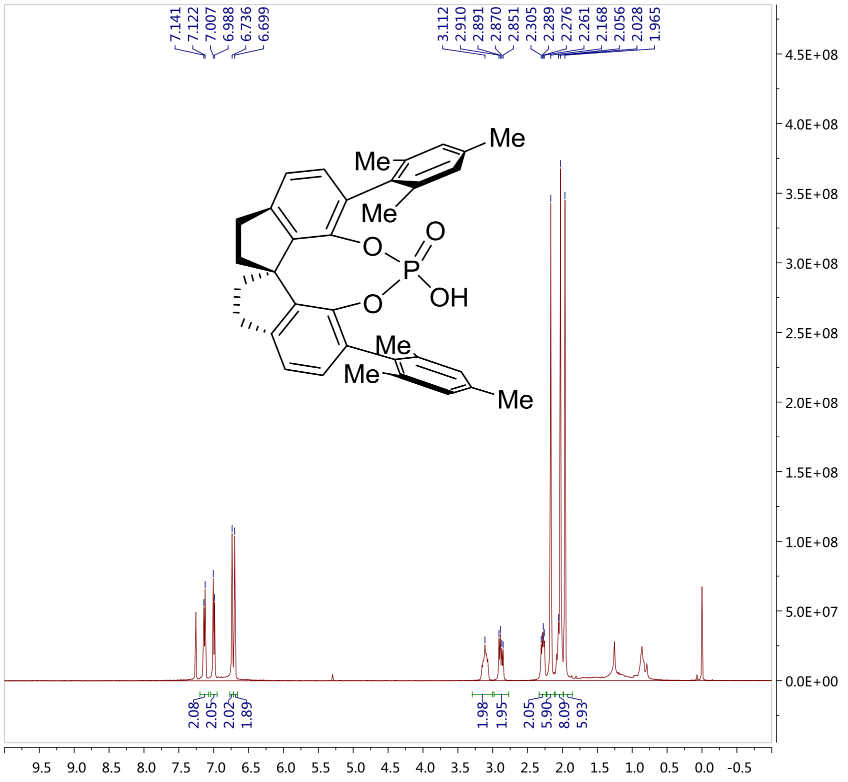

Parameter	Value
1 Data File Name	Z:/ Data/ NMR/ 2014-03/ GJX-20140321-SPA/ 2/ pdata/ 1/ 1r
2 Title	GJX-20140321-SPA
3 Comment	PROTON
4 Origin	Bruker BioSpin GmbH
5 Owner	nmr
6 Site	
7 Spectrometer	spect
8 Author	
9 Solvent	CDCl3
10 Temperature	294.1
11 Pulse Sequence	zg30
12 Experiment	1D
13 Number of Scans	8
14 Receiver Gain	174
15 Relaxation Delay	1.0000
16 Pulse Width	15.0000
17 Acquisition Time	2.4999
18 Acquisition Date	2014-03-21T08:35:20
19 Modification Date	2014-03-21T08:35:00
20 Spectrometer Frequency	400.13
21 Spectral Width	8012.8
22 Lowest Frequency	-1535.4
23 Nucleus	1H
24 Acquired Size	20031
25 Spectral Size	65536

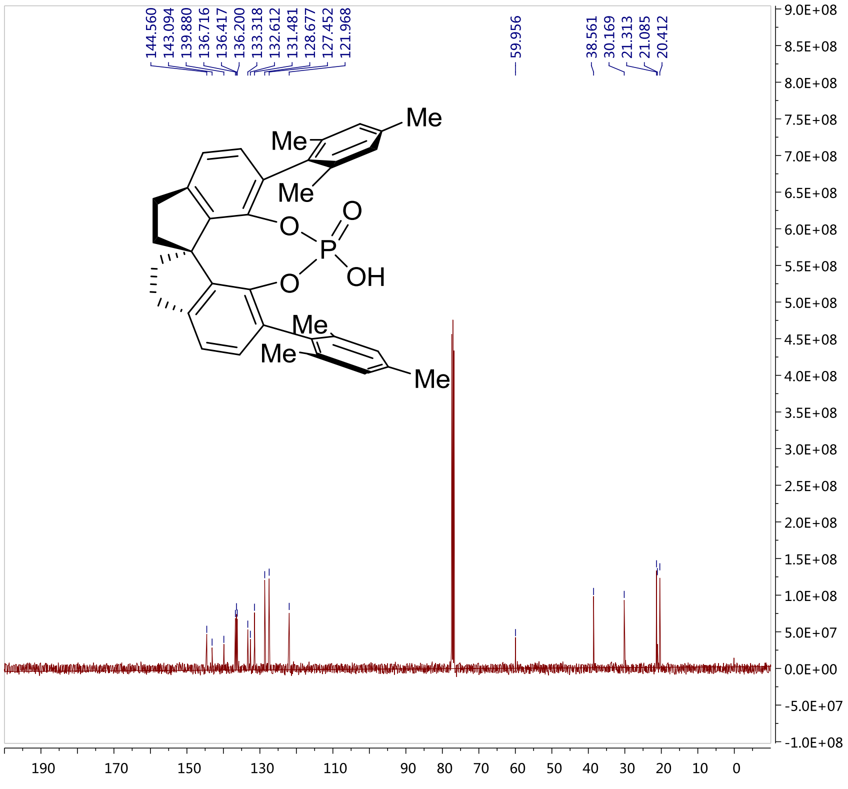

Parameter	Value
1 Data File Name	Z:/ Data/ NMR/ 2014-03/ GJX-20140321-SPA/ 3/ pdata/ 1/ 1r
2 Title	GJX-20140321-SPA
3 Comment	C13CPD
4 Origin	Bruker BioSpin GmbH
5 Owner	nmr
6 Site	
7 Spectrometer	spect
8 Author	
9 Solvent	CDCl3
10 Temperature	295.2
11 Pulse Sequence	zgpg30
12 Experiment	1D
13 Number of Scans	929
14 Receiver Gain	98
15 Relaxation Delay	1.0000
16 Pulse Width	9.8000
17 Acquisition Time	0.4588
18 Acquisition Date	2014-03-21T08:48:40
19 Modification Date	2014-03-21T09:00:00
20 Spectrometer Frequency	100.61
21 Spectral Width	35714.3
22 Lowest Frequency	-7796.8
23 Nucleus	13C
24 Acquired Size	16384
25 Spectral Size	32768

(*E*)-Benzyl 2-diazopent-3-enoate (1a)

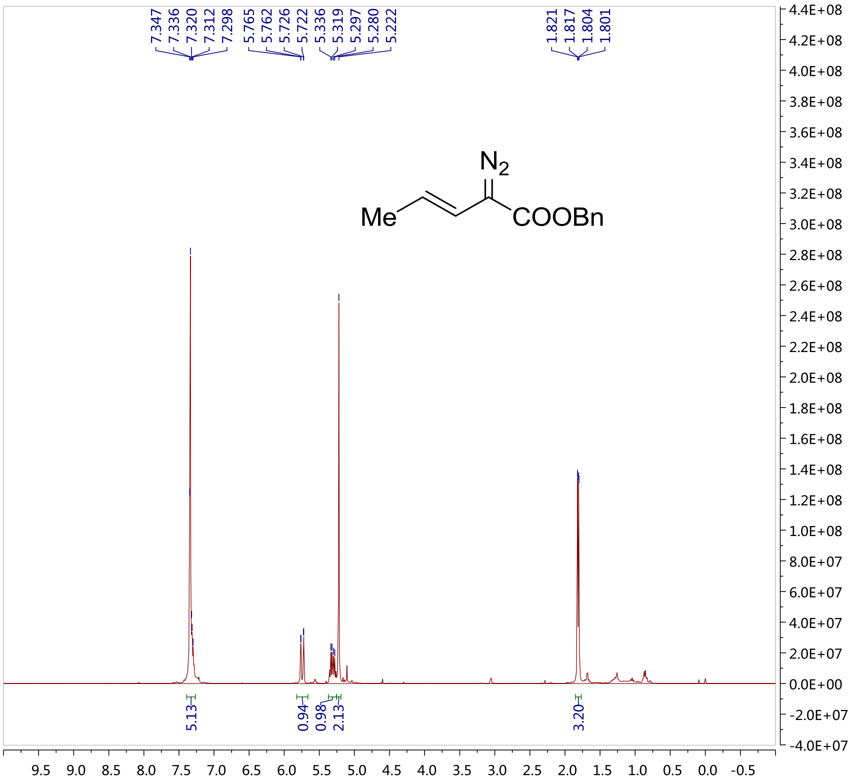

	Parameter	Value
1	Data File Name	Z:/ Data/ NMR/ 2013-1/ GJX-6-24-N2-C/ 1/ pdata/ 1/ 1r
2	Title	GJX-6-24-N2-C
3	Comment	PROTON
4	Origin	Bruker BioSpin GmbH
5	Owner	common
6	Site	
7	Spectrometer	spect
8	Author	
9	Solvent	CDCl3
10	Temperature	290.4
11	Pulse Sequence	zg30
12	Experiment	1D
13	Number of Scans	8
14	Receiver Gain	28
15	Relaxation Delay	1.0000
16	Pulse Width	13.5000
17	Acquisition Time	2.9999
18	Acquisition Date	2013-01-22T21:53:00
19	Modification Date	2013-01-22T21:53:00
20	Spectrometer Frequency	400.13
21	Spectral Width	8223.7
22	Lowest Frequency	-1667.5
23	Nucleus	1H
24	Acquired Size	24670
25	Spectral Size	32768

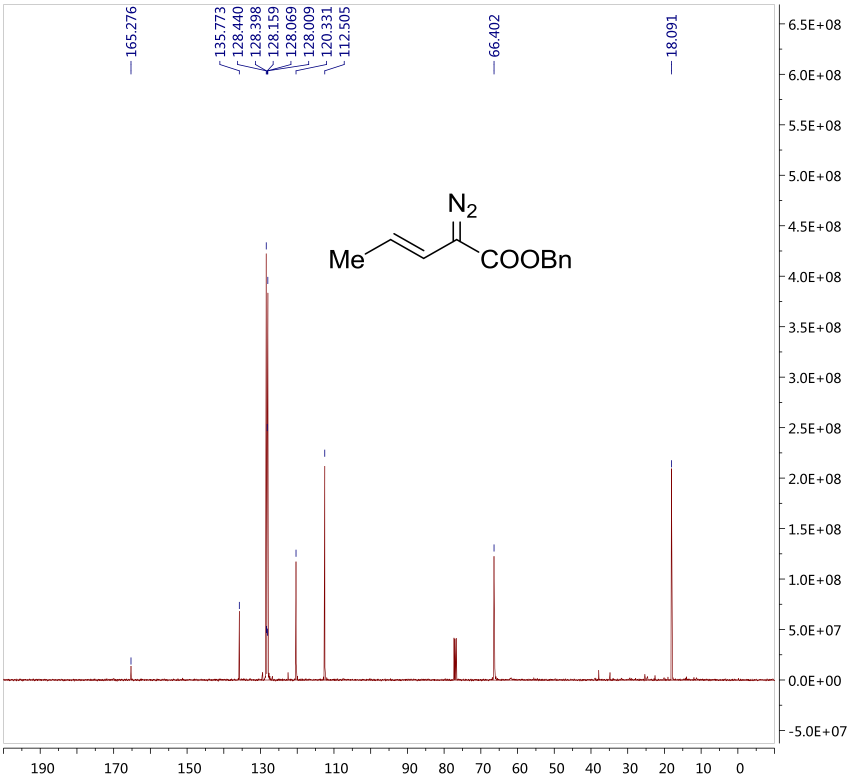

	Parameter	Value
1	Data File Name	Z:/ Data/ NMR/ 2013-1/ GJX-6-24-N2-C/ 2/ pdata/ 1/ 1r
2	Title	GJX-6-24-N2-C
3	Comment	C13CPD
4	Origin	Bruker BioSpin GmbH
5	Owner	common
6	Site	
7	Spectrometer	spect
8	Author	
9	Solvent	CDCl3
10	Temperature	291.6
11	Pulse Sequence	zgpg30
12	Experiment	1D
13	Number of Scans	556
14	Receiver Gain	203
15	Relaxation Delay	1.0000
16	Pulse Width	12.5000
17	Acquisition Time	0.5506
18	Acquisition Date	2013-01-22T22:03:00
19	Modification Date	2013-01-22T22:12:00
20	Spectrometer Frequency	100.61
21	Spectral Width	29761.9
22	Lowest Frequency	-4820.2
23	Nucleus	13C
24	Acquired Size	16384
25	Spectral Size	32768

(*E*)-Benzyl 2-diazohex-3-enoate (1b)

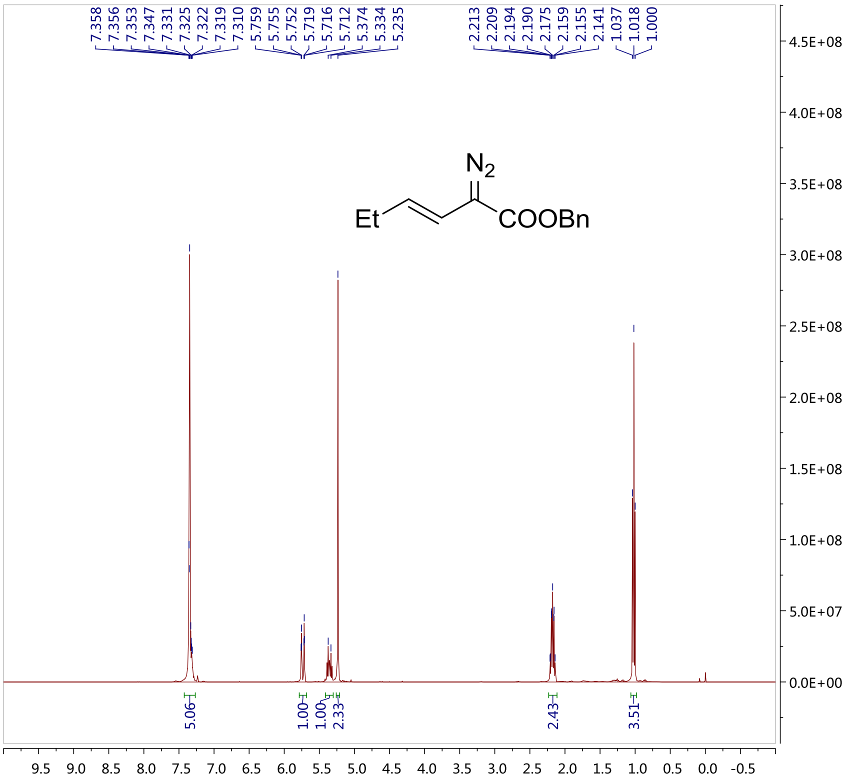

Parameter	Value
1 Data File Name	Z:/ Data/ NMR/ 2013-3/ GJX-6-47-C/ 1/ pdata/ 1/ 1r
2 Title	GJX-6-47-C
3 Comment	PROTON
4 Origin	Bruker BioSpin GmbH
5 Owner	common
6 Site	
7 Spectrometer	spect
8 Author	
9 Solvent	CDCl3
10 Temperature	295.0
11 Pulse Sequence	zg30
12 Experiment	1D
13 Number of Scans	8
14 Receiver Gain	32
15 Relaxation Delay	1.0000
16 Pulse Width	13.7000
17 Acquisition Time	1.9999
18 Acquisition Date	2013-03-07T11:16:00
19 Modification Date	2013-03-07T11:16:00
20 Spectrometer Frequency	400.13
21 Spectral Width	8223.7
22 Lowest Frequency	-1640.9
23 Nucleus	1H
24 Acquired Size	16446
25 Spectral Size	32768

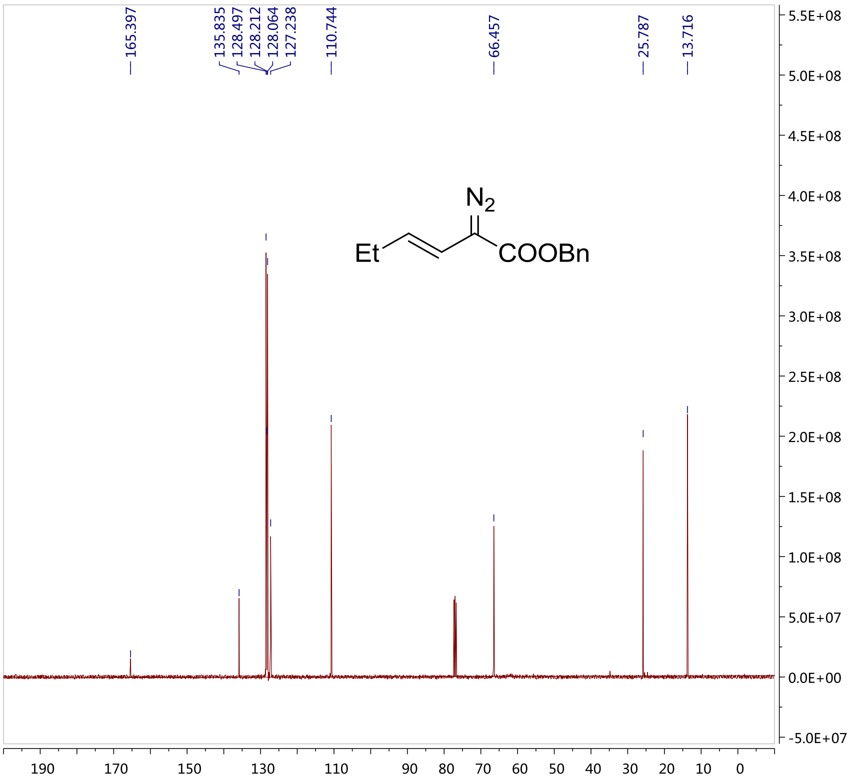

Parameter	Value
1 Data File Name	Z:/ Data/ NMR/ 2013-3/ GJX-6-47-C/ 2/ pdata/ 1/ 1r
2 Title	GJX-6-47-C
3 Comment	C13CPD
4 Origin	Bruker BioSpin GmbH
5 Owner	common
6 Site	
7 Spectrometer	spect
8 Author	
9 Solvent	CDCl3
10 Temperature	295.7
11 Pulse Sequence	zgpg30
12 Experiment	1D
13 Number of Scans	253
14 Receiver Gain	203
15 Relaxation Delay	1.0000
16 Pulse Width	12.0000
17 Acquisition Time	0.4588
18 Acquisition Date	2013-03-07T11:18:00
19 Modification Date	2013-03-07T11:23:00
20 Spectrometer Frequency	100.61
21 Spectral Width	35714.3
22 Lowest Frequency	-7796.3
23 Nucleus	13C
24 Acquired Size	16384
25 Spectral Size	32768

(*E*)-Benzyl 2-diazooct-3-enoate (1c)

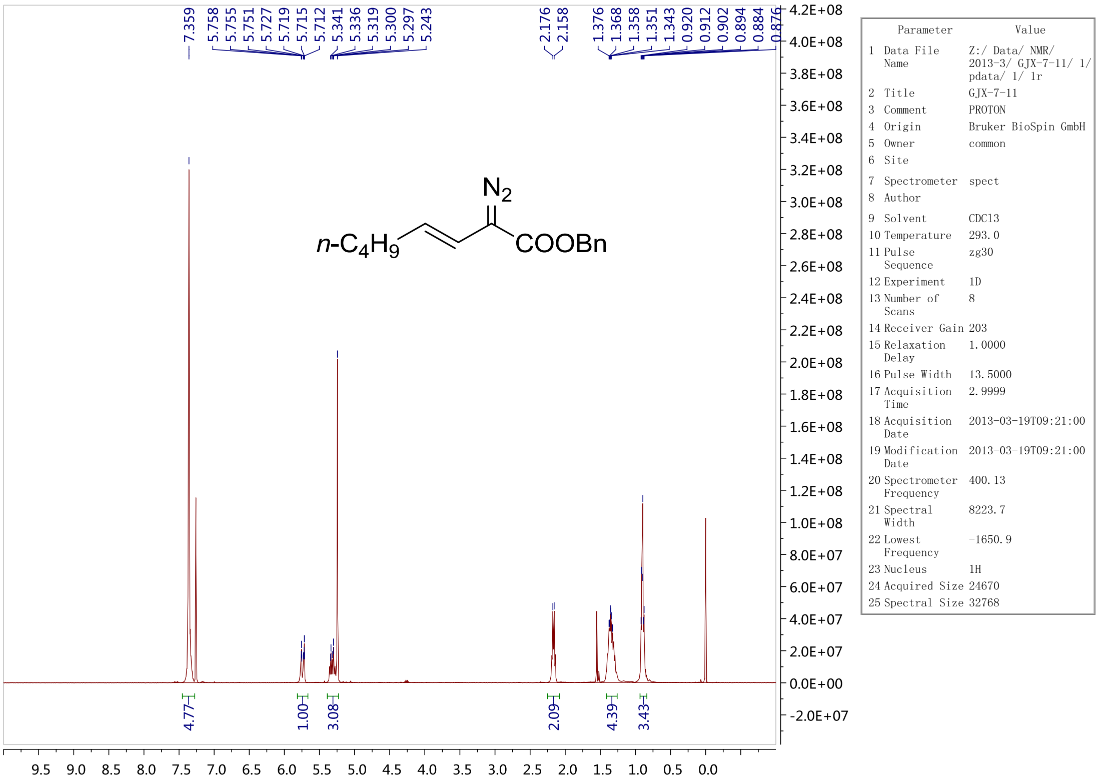

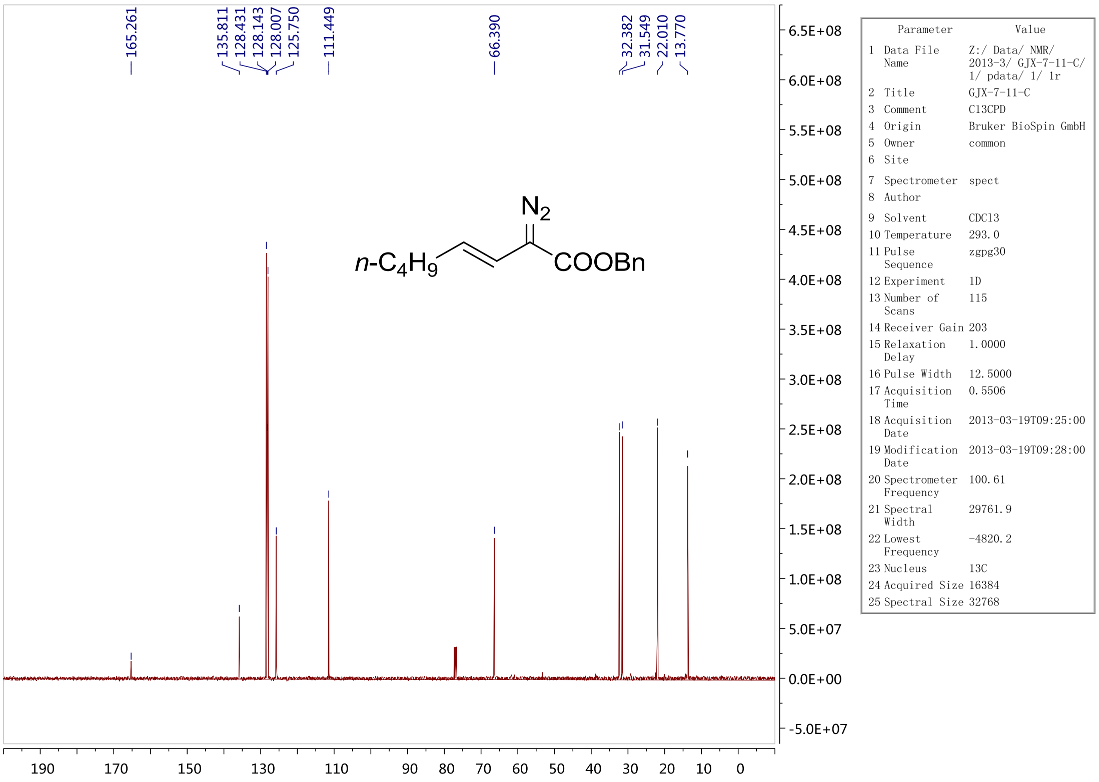

(*E*)-Benzyl 2-diazodec-3-enoate (1d)

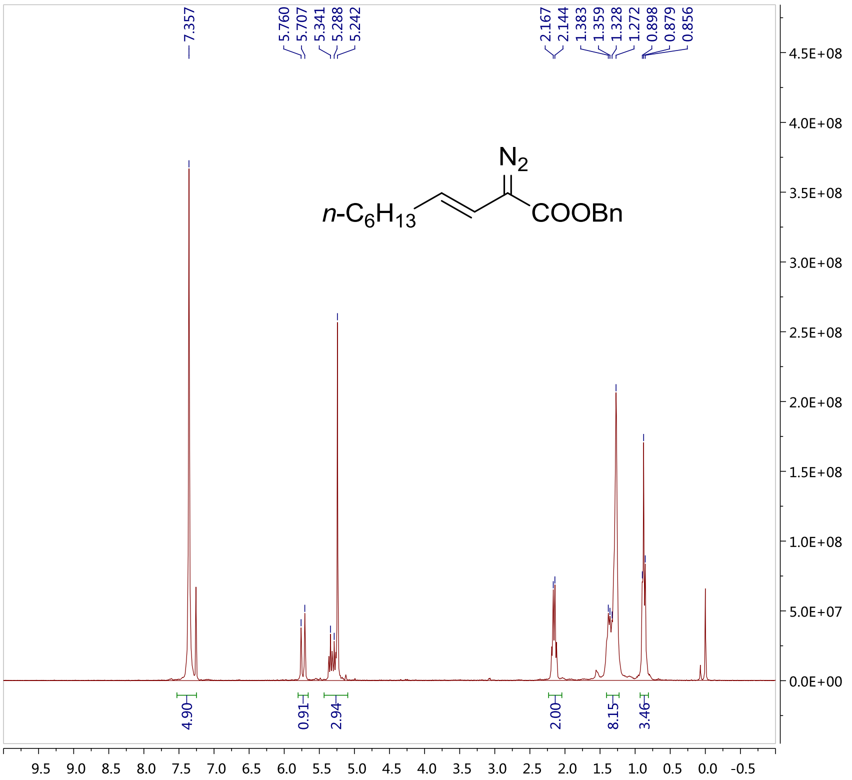

Parameter		Value
1	Data File Name	Z:/ Data/ NMR/ 2013-3/ GJX-6-44/ 1/ pdata/ 1/ 1r
2	Title	GJX-6-44
3	Comment	PROTON
4	Origin	Bruker BioSpin GmbH
5	Owner	common
6	Site	
7	Spectrometer	spect
8	Author	
9	Solvent	CDCl3
10	Temperature	295.5
11	Pulse Sequence	zg30
12	Experiment	1D
13	Number of Scans	8
14	Receiver Gain	645
15	Relaxation Delay	1.0000
16	Pulse Width	12.1000
17	Acquisition Time	2.6543
18	Acquisition Date	2013-03-05T14:24:00
19	Modification Date	2013-03-05T14:24:00
20	Spectrometer Frequency	300.13
21	Spectral Width	6172.8
22	Lowest Frequency	-1239.6
23	Nucleus	1H
24	Acquired Size	16384
25	Spectral Size	32768

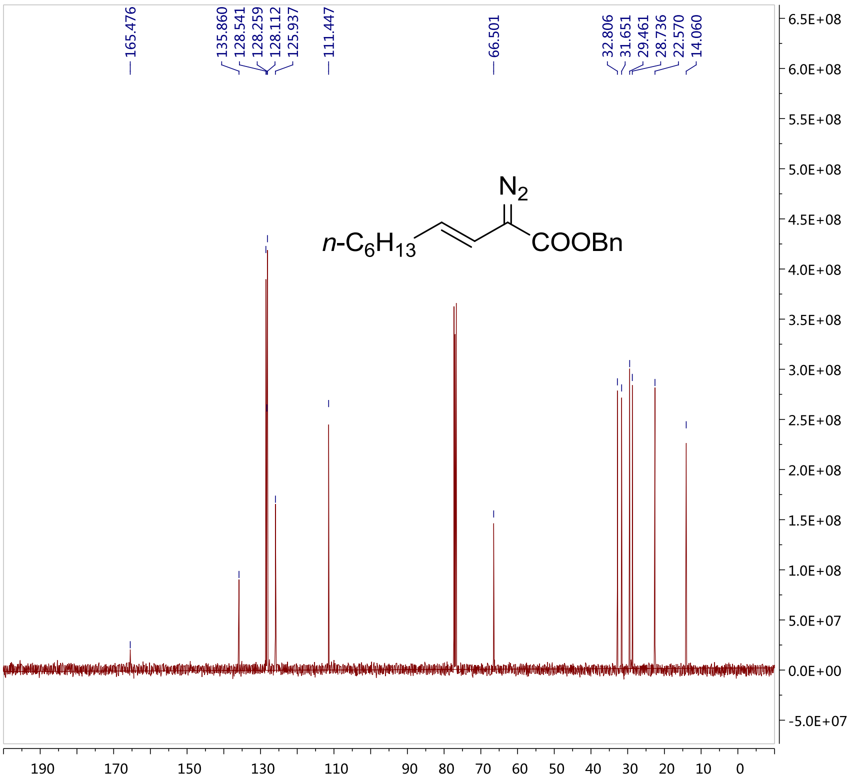

Parameter		Value
1	Data File Name	Z:/ Data/ NMR/ 2013-3/ GJX-6-44-C/ 1/ pdata/ 1/ 1r
2	Title	GJX-6-44-C
3	Comment	C13CPD
4	Origin	Bruker BioSpin GmbH
5	Owner	common
6	Site	
7	Spectrometer	spect
8	Author	
9	Solvent	CDCl3
10	Temperature	295.7
11	Pulse Sequence	zgpg30
12	Experiment	1D
13	Number of Scans	212
14	Receiver Gain	203
15	Relaxation Delay	1.0000
16	Pulse Width	12.0000
17	Acquisition Time	0.4588
18	Acquisition Date	2013-03-05T15:04:00
19	Modification Date	2013-03-05T15:06:00
20	Spectrometer Frequency	100.61
21	Spectral Width	35714.3
22	Lowest Frequency	-7796.3
23	Nucleus	13C
24	Acquired Size	16384
25	Spectral Size	32768

(*E*)-Benzyl 2-diazo-5-methylhex-3-enoate (1e)

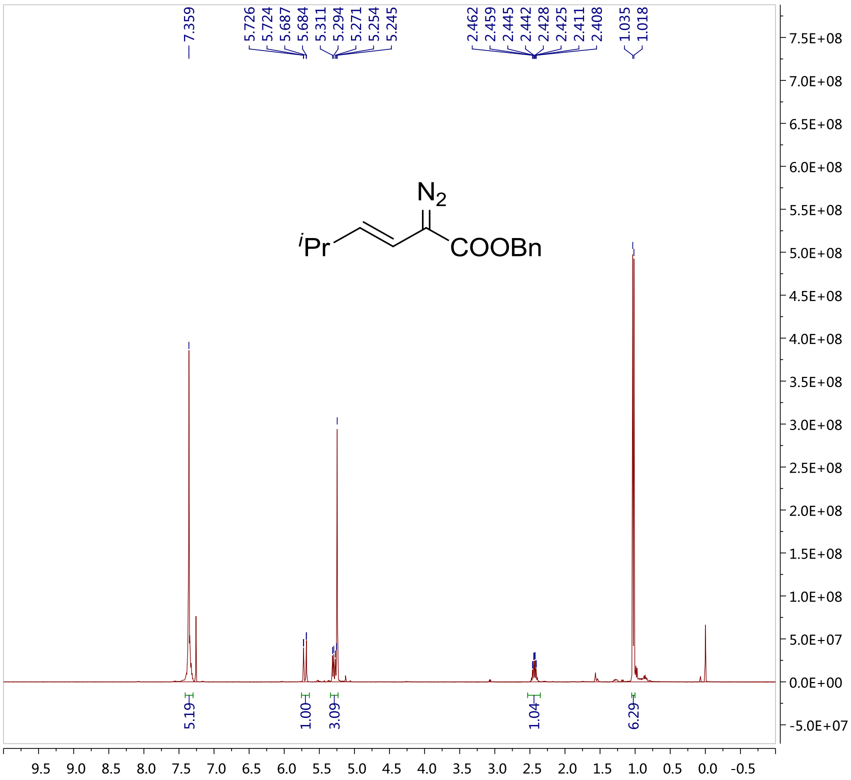

Parameter	Value
1 Data File Name	Z:/ Data/ NMR/ 2013-3/ GJX-7-6-H/ 1/ pdata/ 1/ 1r
2 Title	GJX-7-6-H
3 Comment	PROTON
4 Origin	Bruker BioSpin GmbH
5 Owner	common
6 Site	
7 Spectrometer	spect
8 Author	
9 Solvent	CDCl3
10 Temperature	291.3
11 Pulse Sequence	zg30
12 Experiment	1D
13 Number of Scans	8
14 Receiver Gain	114
15 Relaxation Delay	1.0000
16 Pulse Width	13.5000
17 Acquisition Time	1.9923
18 Acquisition Date	2013-03-15T09:02:00
19 Modification Date	2013-03-15T09:02:00
20 Spectrometer Frequency	400.23
21 Spectral Width	8223.7
22 Lowest Frequency	-1654.1
23 Nucleus	1H
24 Acquired Size	16384
25 Spectral Size	32768

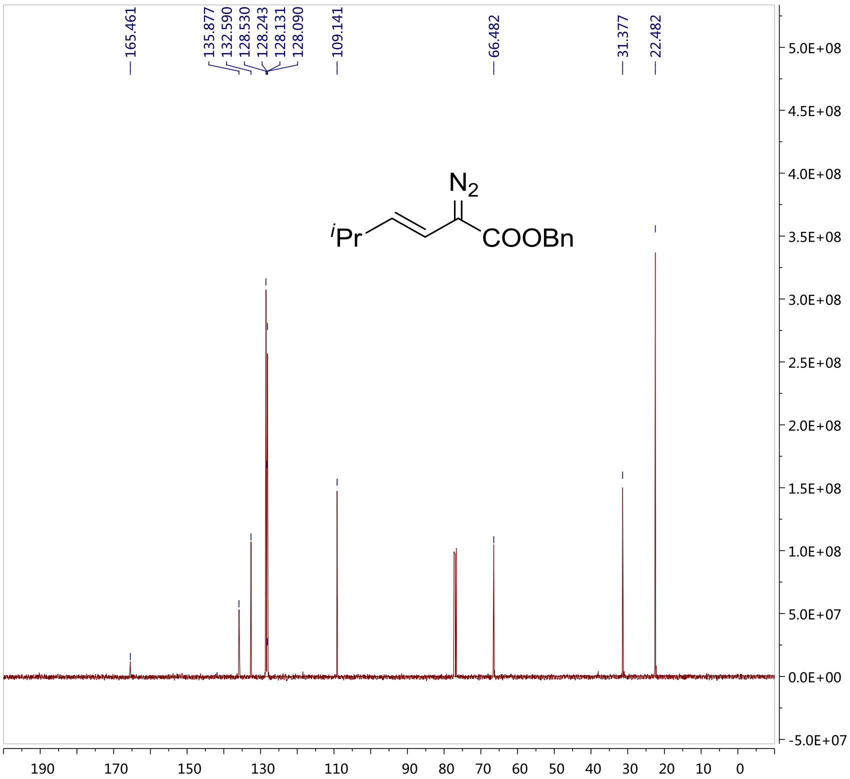

Parameter	Value
1 Data File Name	Z:/ Data/ NMR/ 2013-3/ GJX-7-6-C/ 1/ pdata/ 1/ 1r
2 Title	GJX-7-6-C
3 Comment	C13CPD
4 Origin	Bruker BioSpin GmbH
5 Owner	common
6 Site	
7 Spectrometer	spect
8 Author	
9 Solvent	CDCl3
10 Temperature	292.0
11 Pulse Sequence	zgpg30
12 Experiment	1D
13 Number of Scans	514
14 Receiver Gain	203
15 Relaxation Delay	1.0000
16 Pulse Width	12.0000
17 Acquisition Time	0.5506
18 Acquisition Date	2013-03-15T09:11:00
19 Modification Date	2013-03-15T09:20:00
20 Spectrometer Frequency	100.64
21 Spectral Width	29761.9
22 Lowest Frequency	-4817.6
23 Nucleus	13C
24 Acquired Size	16384
25 Spectral Size	32768

(*E*)-Benzyl 2-diazo-4-phenylbut-3-enoate (1f)

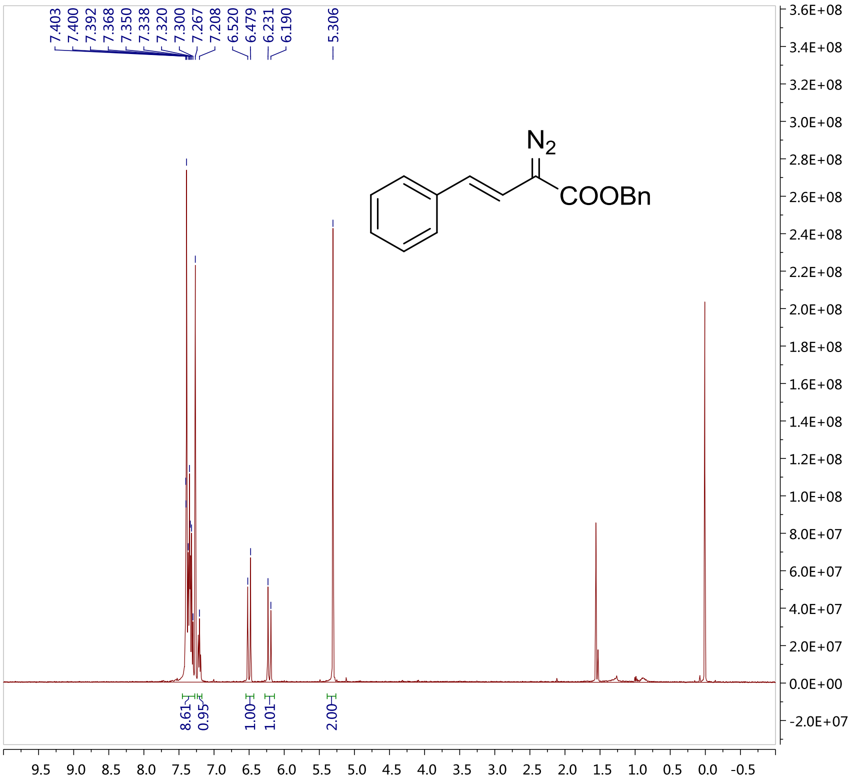

Parameter	Value
1 Data File Name	Z:/ Data/ NMR/ 2013-3/ GJX-7-7-Bn-N2/ 1/ pdata/ 1/ 1r
2 Title	GJX-7-7-Bn-N2
3 Comment	PROTON
4 Origin	Bruker BioSpin GmbH
5 Owner	common
6 Site	
7 Spectrometer	spect
8 Author	
9 Solvent	CDCl3
10 Temperature	294.8
11 Pulse Sequence	zg30
12 Experiment	1D
13 Number of Scans	8
14 Receiver Gain	203
15 Relaxation Delay	1.0000
16 Pulse Width	13.7000
17 Acquisition Time	1.9999
18 Acquisition Date	2013-03-16T11:09:00
19 Modification Date	2013-03-16T11:09:00
20 Spectrometer Frequency	400.13
21 Spectral Width	8223.7
22 Lowest Frequency	-1640.9
23 Nucleus	1H
24 Acquired Size	16446
25 Spectral Size	32768

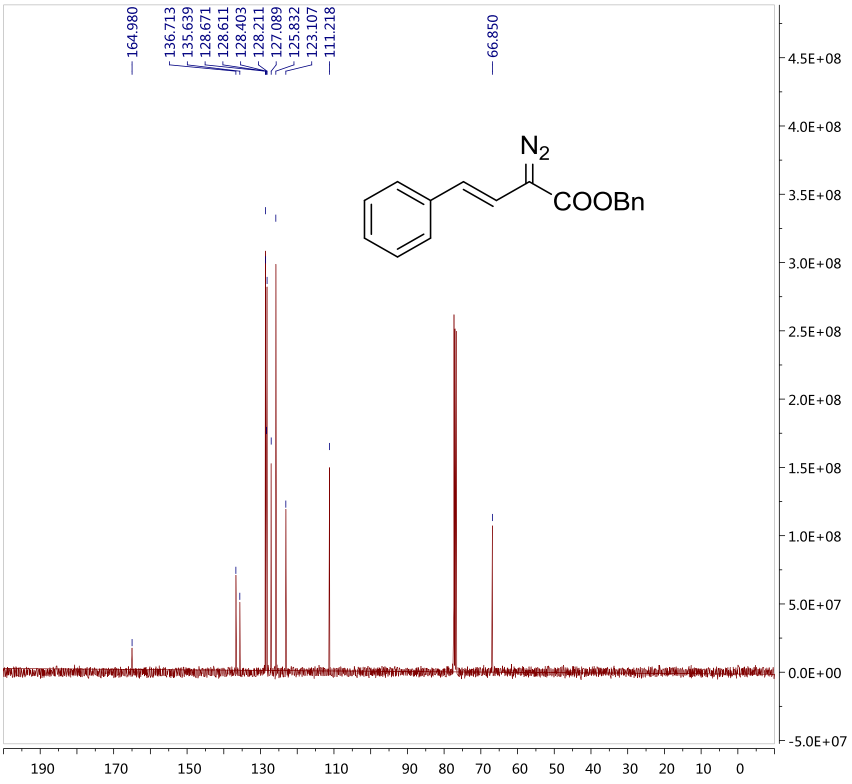

Parameter	Value
1 Data File Name	Z:/ Data/ NMR/ 2013-3/ GJX-7-7-Bn-N2-C/ 1/ pdata/ 1/ 1r
2 Title	GJX-7-7-Bn-N2-C
3 Comment	C13CPD
4 Origin	Bruker BioSpin GmbH
5 Owner	common
6 Site	
7 Spectrometer	spect
8 Author	
9 Solvent	CDCl3
10 Temperature	295.5
11 Pulse Sequence	zgpg30
12 Experiment	1D
13 Number of Scans	330
14 Receiver Gain	203
15 Relaxation Delay	1.0000
16 Pulse Width	12.0000
17 Acquisition Time	0.4588
18 Acquisition Date	2013-03-16T11:13:00
19 Modification Date	2013-03-16T11:21:00
20 Spectrometer Frequency	100.61
21 Spectral Width	35714.3
22 Lowest Frequency	-7796.3
23 Nucleus	13C
24 Acquired Size	16384
25 Spectral Size	32768

(*E*)-Benzyl 2-diazo-4-phenylpent-3-enoate (1g)

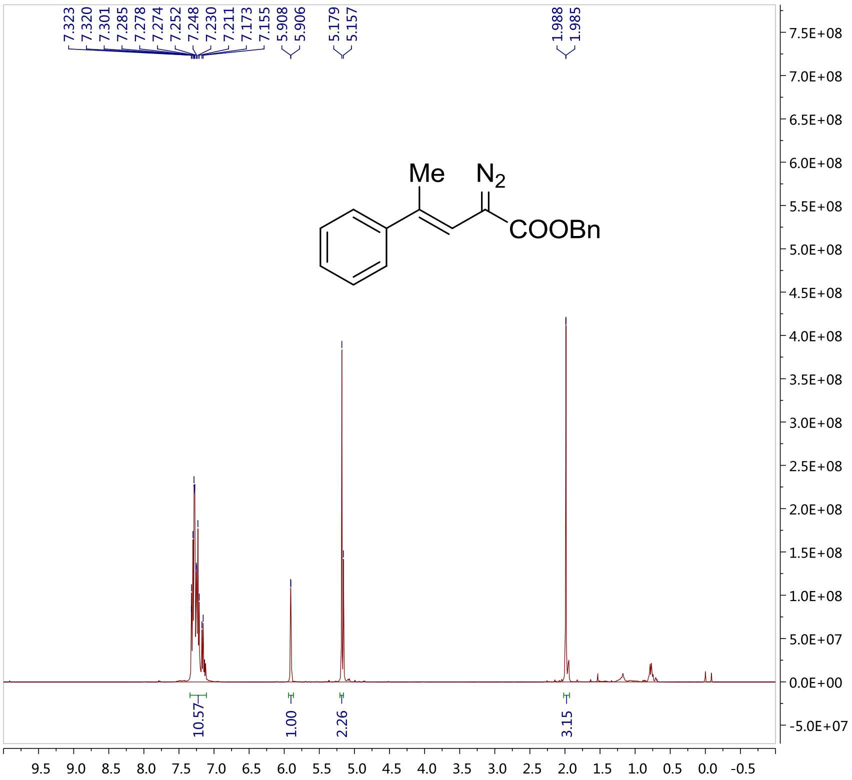

Parameter	Value
1 Data File Name	Z:/ Data/ NMR/ 2013-1/ GJX-6-21-N2/ 1/ pdata/ 1/ 1r
2 Title	GJX-6-21-N2
3 Comment	PROTON
4 Origin	Bruker BioSpin GmbH
5 Owner	common
6 Site	
7 Spectrometer	spect
8 Author	
9 Solvent	CDCl3
10 Temperature	290.2
11 Pulse Sequence	zg30
12 Experiment	1D
13 Number of Scans	8
14 Receiver Gain	40
15 Relaxation Delay	1.0000
16 Pulse Width	13.5000
17 Acquisition Time	2.9999
18 Acquisition Date	2013-01-21T16:33:00
19 Modification Date	2013-01-21T16:33:00
20 Spectrometer Frequency	400.13
21 Spectral Width	8223.7
22 Lowest Frequency	-1706.2
23 Nucleus	1H
24 Acquired Size	24670
25 Spectral Size	32768

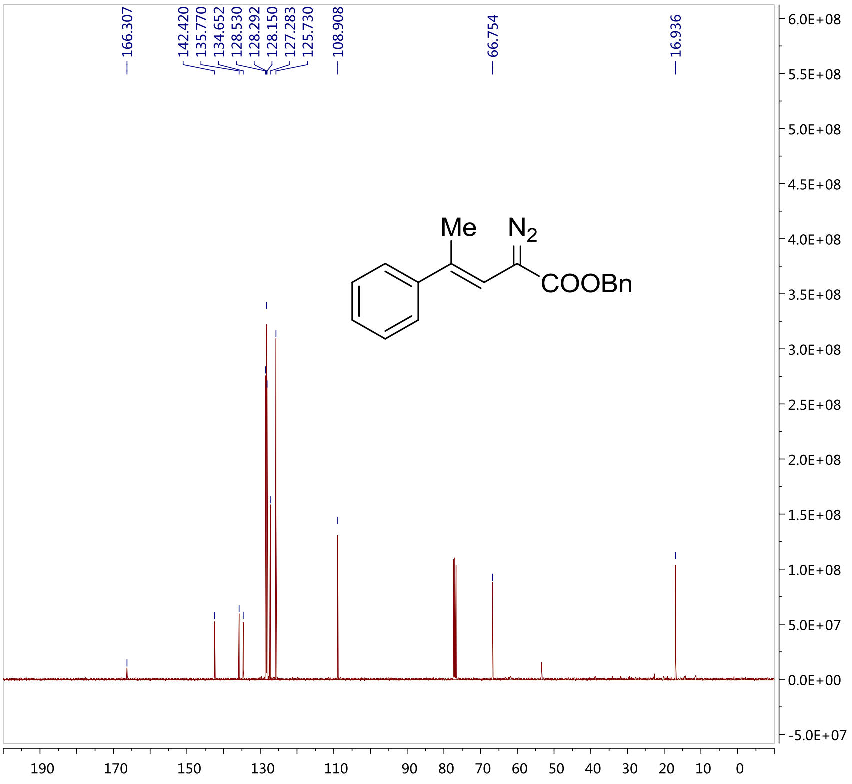

Parameter	Value
1 Data File Name	Z:/ Data/ NMR/ 2013-1/ GJX-6-21-N2/ 2/ pdata/ 1/ 1r
2 Title	GJX-6-21-N2
3 Comment	C13CPD
4 Origin	Bruker BioSpin GmbH
5 Owner	common
6 Site	
7 Spectrometer	spect
8 Author	
9 Solvent	CDCl3
10 Temperature	291.0
11 Pulse Sequence	zgpg30
12 Experiment	1D
13 Number of Scans	739
14 Receiver Gain	203
15 Relaxation Delay	1.0000
16 Pulse Width	12.5000
17 Acquisition Time	0.5506
18 Acquisition Date	2013-01-21T16:36:00
19 Modification Date	2013-01-21T16:54:00
20 Spectrometer Frequency	100.61
21 Spectral Width	29761.9
22 Lowest Frequency	-4820.2
23 Nucleus	13C
24 Acquired Size	16384
25 Spectral Size	32768

(*E*)-Benzyl 4-(4-chlorophenyl)-2-diazopent-3-enoate (1h)

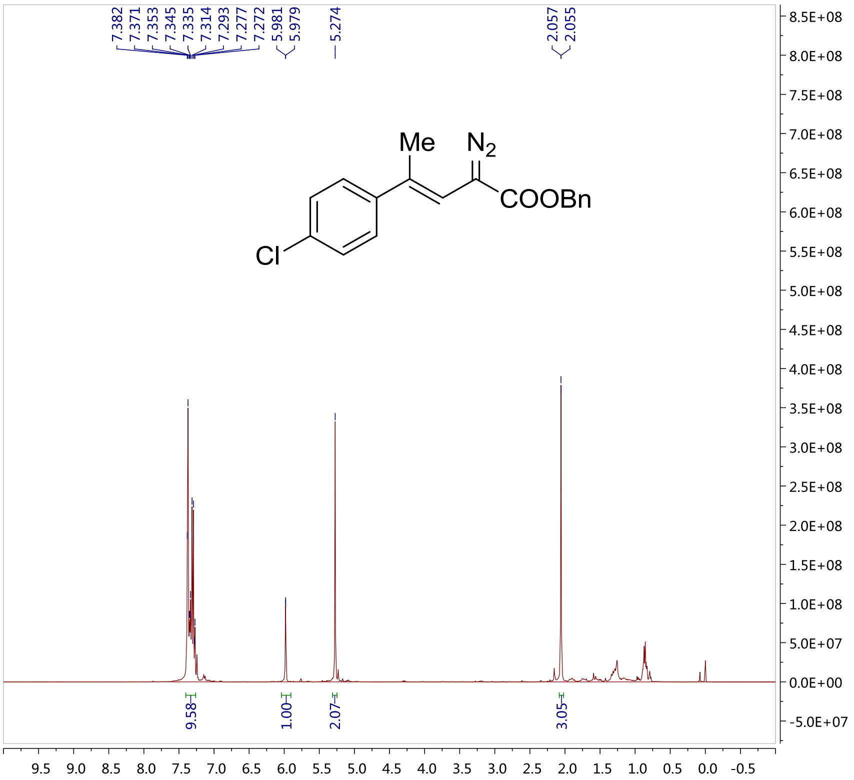

	Parameter	Value
1	Data File Name	Z:/ Data/ NMR/ 2013-4/ GJX-7-42-3/ 1/ pdata/ 1/ 1r
2	Title	GJX-7-42-3
3	Comment	PROTON
4	Origin	Bruker BioSpin GmbH
5	Owner	common
6	Site	
7	Spectrometer	spect
8	Author	
9	Solvent	CDCl3
10	Temperature	294.8
11	Pulse Sequence	zg30
12	Experiment	1D
13	Number of Scans	8
14	Receiver Gain	90
15	Relaxation Delay	1.0000
16	Pulse Width	13.7000
17	Acquisition Time	1.9999
18	Acquisition Date	2013-04-09T15:13:00
19	Modification Date	2013-04-09T15:13:00
20	Spectrometer Frequency	400.13
21	Spectral Width	8223.7
22	Lowest Frequency	-1656.0
23	Nucleus	1H
24	Acquired Size	16446
25	Spectral Size	32768

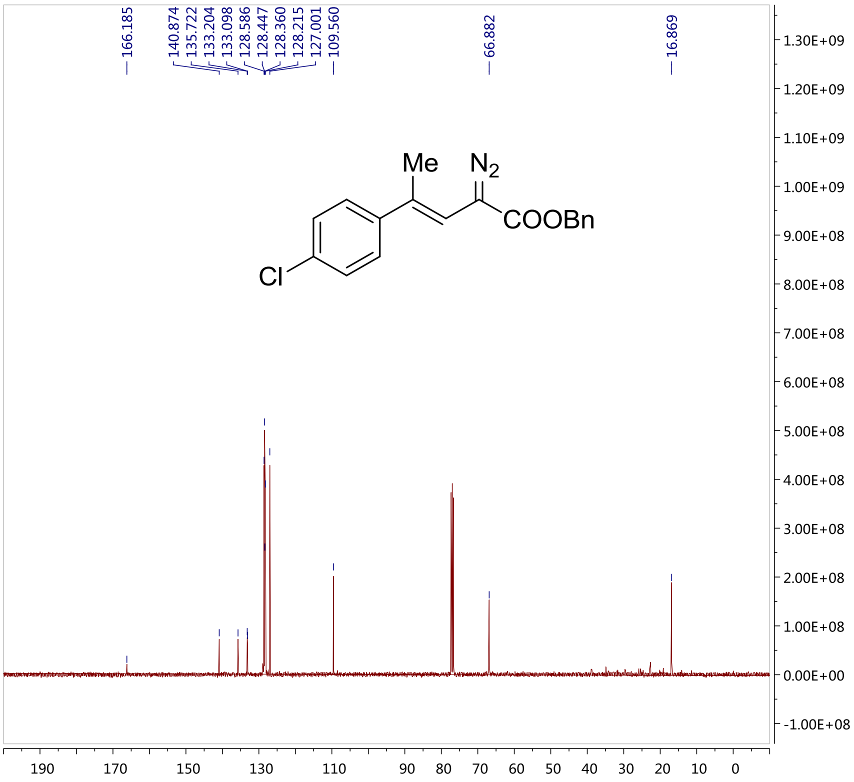

	Parameter	Value
1	Data File Name	Z:/ Data/ NMR/ 2013-4/ GJX-7-42-3/ 2/ pdata/ 1/ 1r
2	Title	GJX-7-42-3
3	Comment	C13CPD
4	Origin	Bruker BioSpin GmbH
5	Owner	common
6	Site	
7	Spectrometer	spect
8	Author	
9	Solvent	CDCl3
10	Temperature	294.9
11	Pulse Sequence	zgpg30
12	Experiment	1D
13	Number of Scans	490
14	Receiver Gain	203
15	Relaxation Delay	1.0000
16	Pulse Width	12.0000
17	Acquisition Time	0.4588
18	Acquisition Date	2013-04-09T15:14:00
19	Modification Date	2013-04-09T15:26:00
20	Spectrometer Frequency	100.61
21	Spectral Width	35714.3
22	Lowest Frequency	-7796.3
23	Nucleus	13C
24	Acquired Size	16384
25	Spectral Size	32768

(*E*)-Benzyl 2-diazo-4-(4-fluorophenyl)pent-3-enoate (1i)

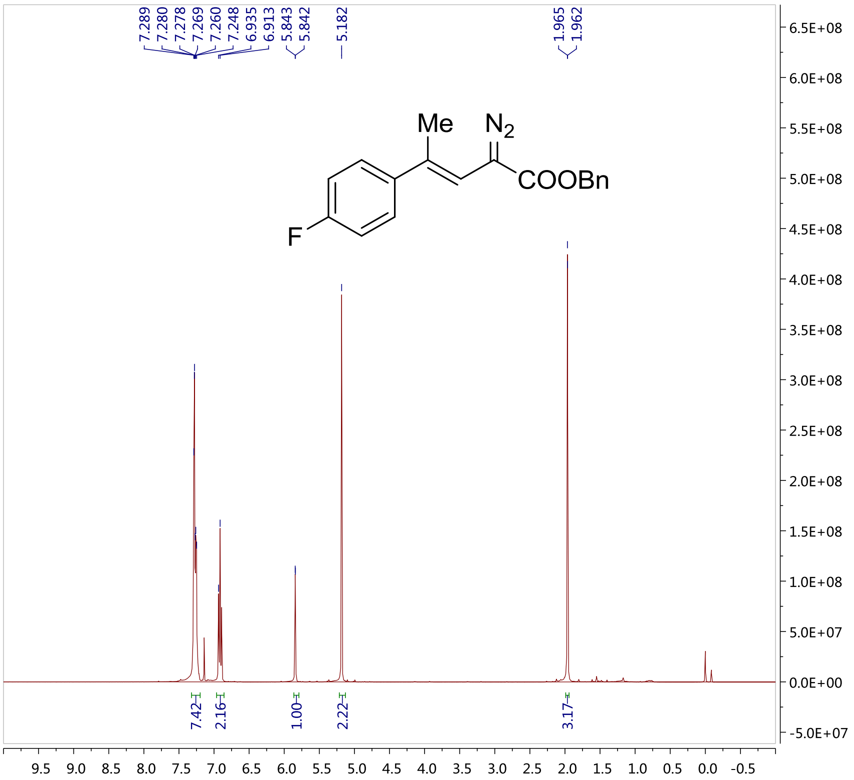

	Parameter	Value
1	Data File Name	Z:/ Data/ NMR/ 2013-4/ GJX-7-47-1/ 1/ pdata/ 1/ 1r
2	Title	GJX-7-47-1
3	Comment	PROTON
4	Origin	Bruker BioSpin GmbH
5	Owner	common
6	Site	
7	Spectrometer	spect
8	Author	
9	Solvent	CDCl3
10	Temperature	291.0
11	Pulse Sequence	zg30
12	Experiment	1D
13	Number of Scans	8
14	Receiver Gain	64
15	Relaxation Delay	1.0000
16	Pulse Width	13.5000
17	Acquisition Time	1.9923
18	Acquisition Date	2013-04-12T10:06:00
19	Modification Date	2013-04-12T10:06:00
20	Spectrometer Frequency	400.23
21	Spectral Width	8223.7
22	Lowest Frequency	-1700.9
23	Nucleus	1H
24	Acquired Size	16384
25	Spectral Size	32768

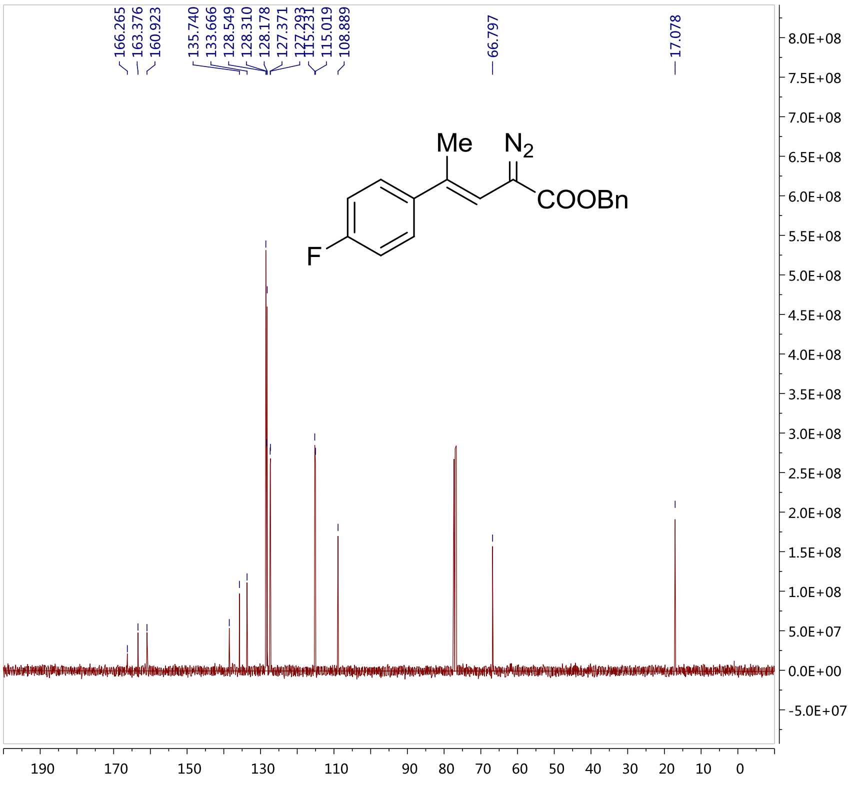

	Parameter	Value
1	Data File Name	Z:/ Data/ NMR/ 2013-4/ GJX-7-47-1/ 2/ pdata/ 1/ 1r
2	Title	GJX-7-47-1
3	Comment	C13CPD
4	Origin	Bruker BioSpin GmbH
5	Owner	common
6	Site	
7	Spectrometer	spect
8	Author	
9	Solvent	CDCl3
10	Temperature	291.0
11	Pulse Sequence	zgpg30
12	Experiment	1D
13	Number of Scans	97
14	Receiver Gain	203
15	Relaxation Delay	1.0000
16	Pulse Width	12.0000
17	Acquisition Time	0.5506
18	Acquisition Date	2013-04-12T10:07:00
19	Modification Date	2013-04-12T10:10:00
20	Spectrometer Frequency	100.64
21	Spectral Width	29761.9
22	Lowest Frequency	-4817.6
23	Nucleus	13C
24	Acquired Size	16384
25	Spectral Size	32768

(*E*)-Benzyl 2-diazo-4-(4-methoxyphenyl)pent-3-enoate (1j)

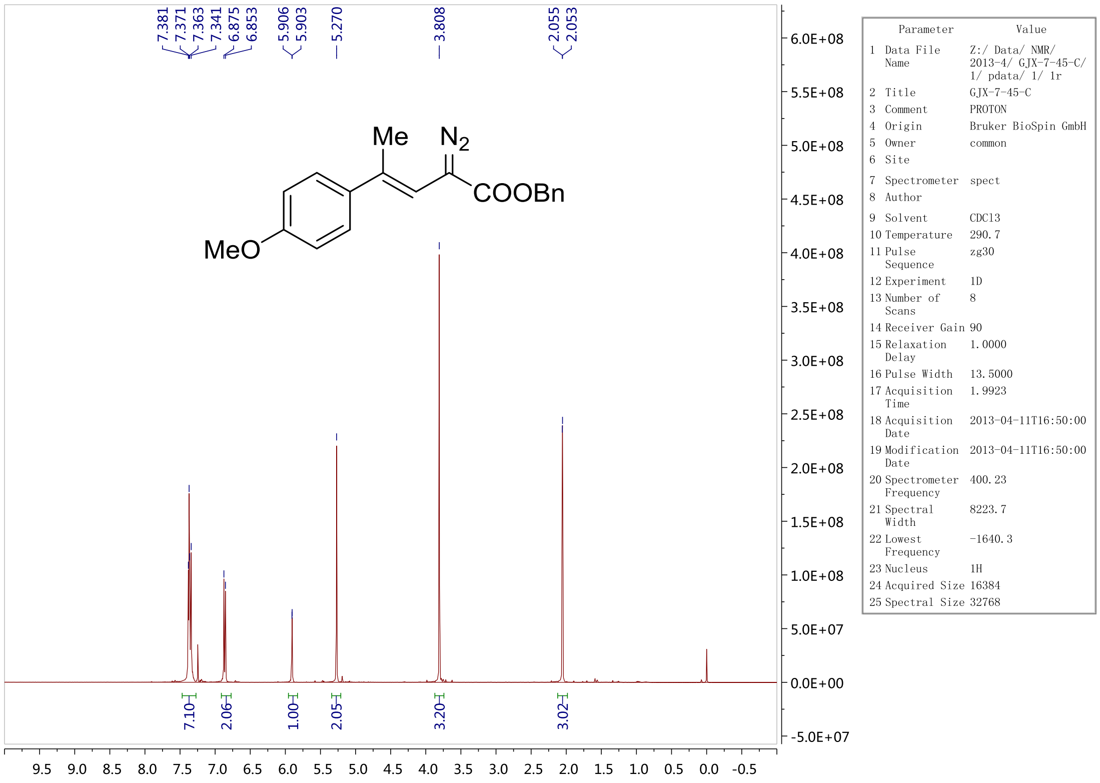

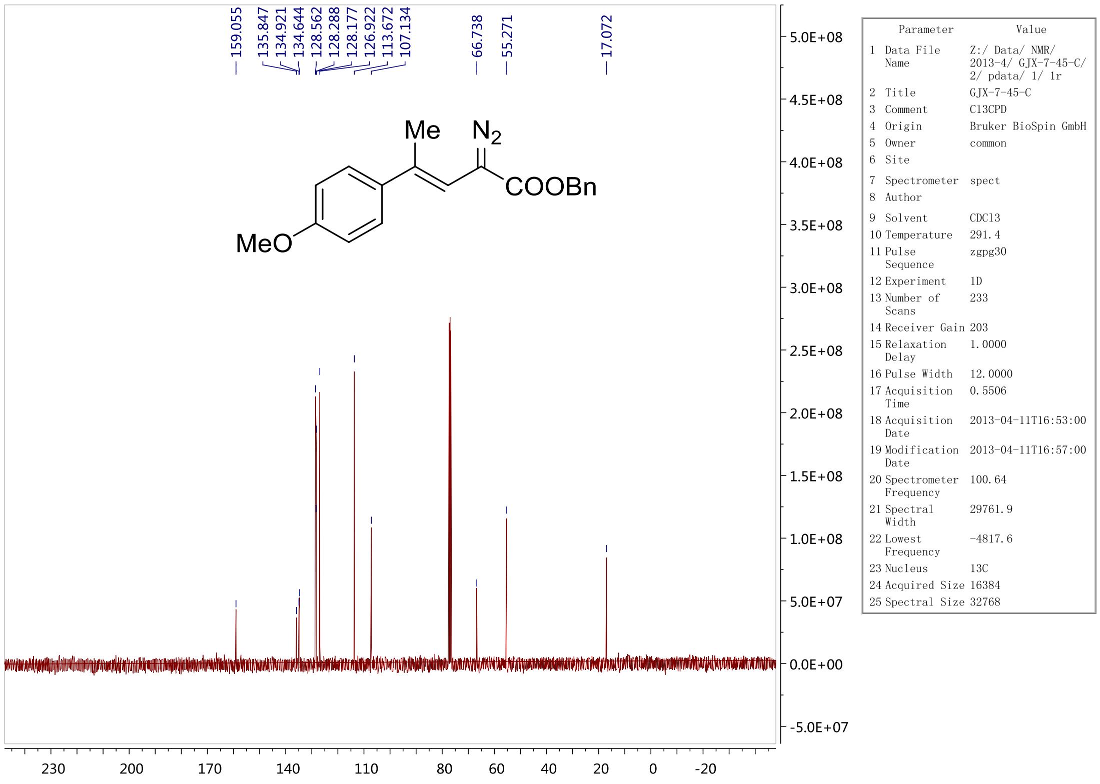

(*E*)-Benzyl 2-diazo-4-(naphthalen-2-yl)pent-3-enoate (1k)

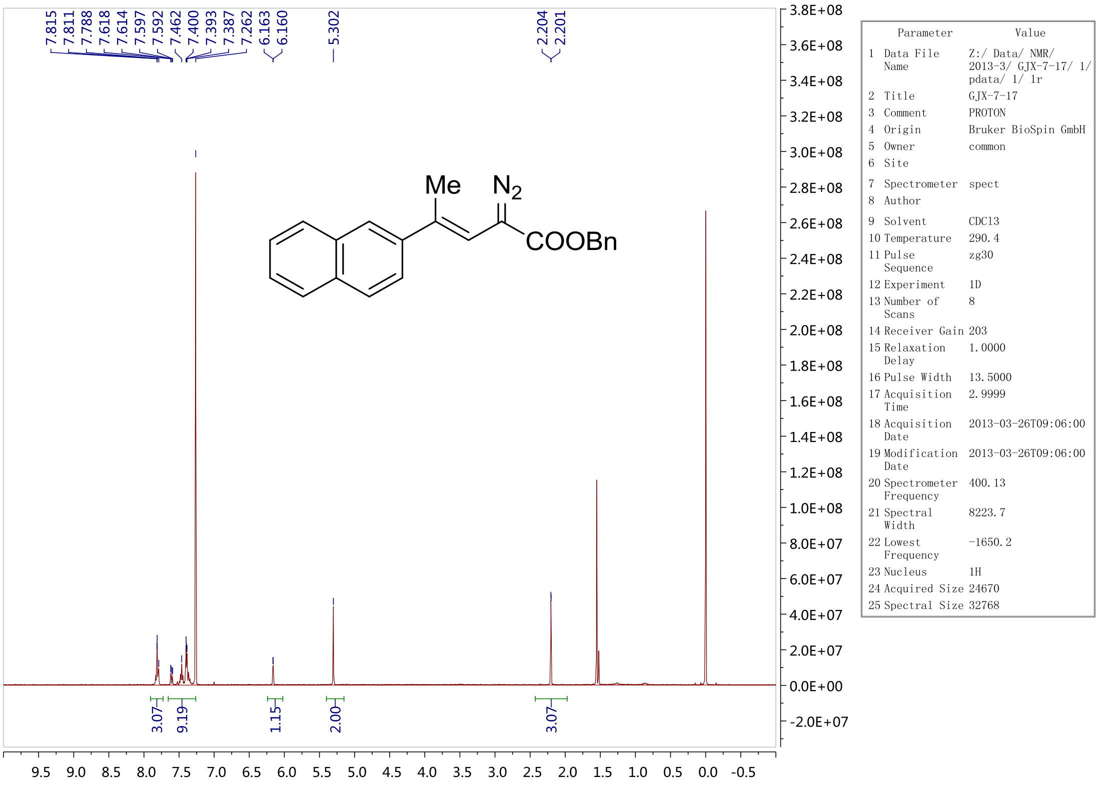

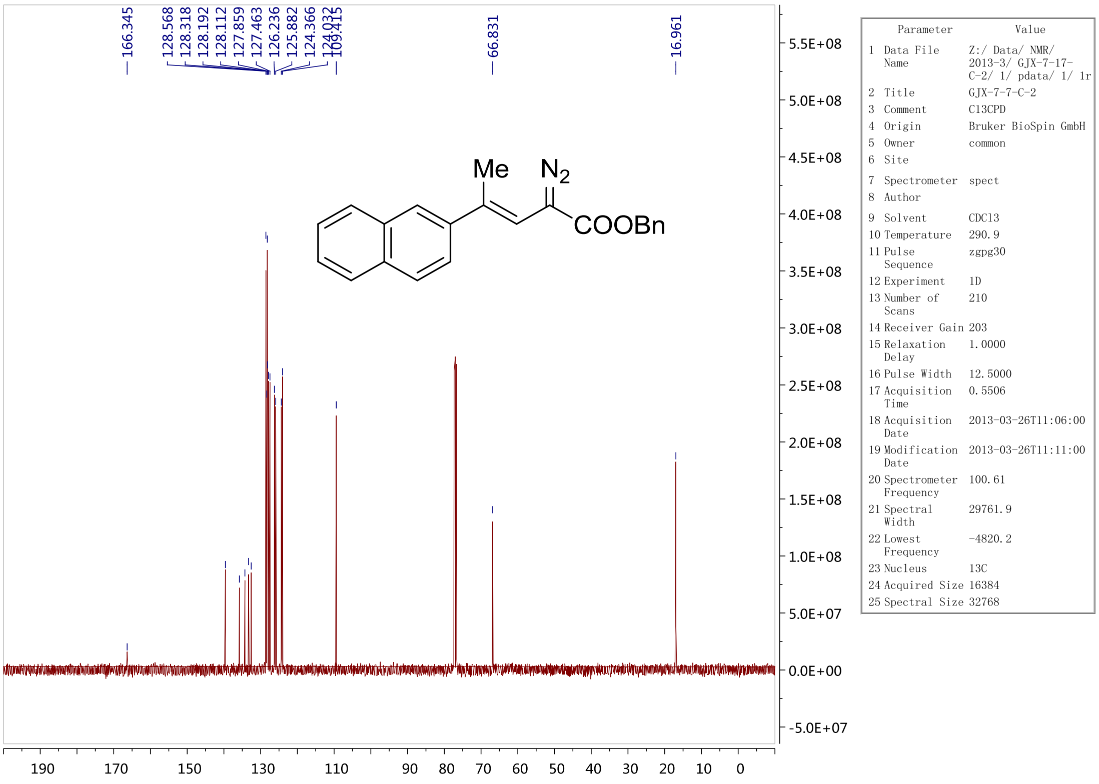

(*E*)-Benzyl 4-(benzo[d][1,3]dioxol-5-yl)-2-diazopent-3-enoate (1l)

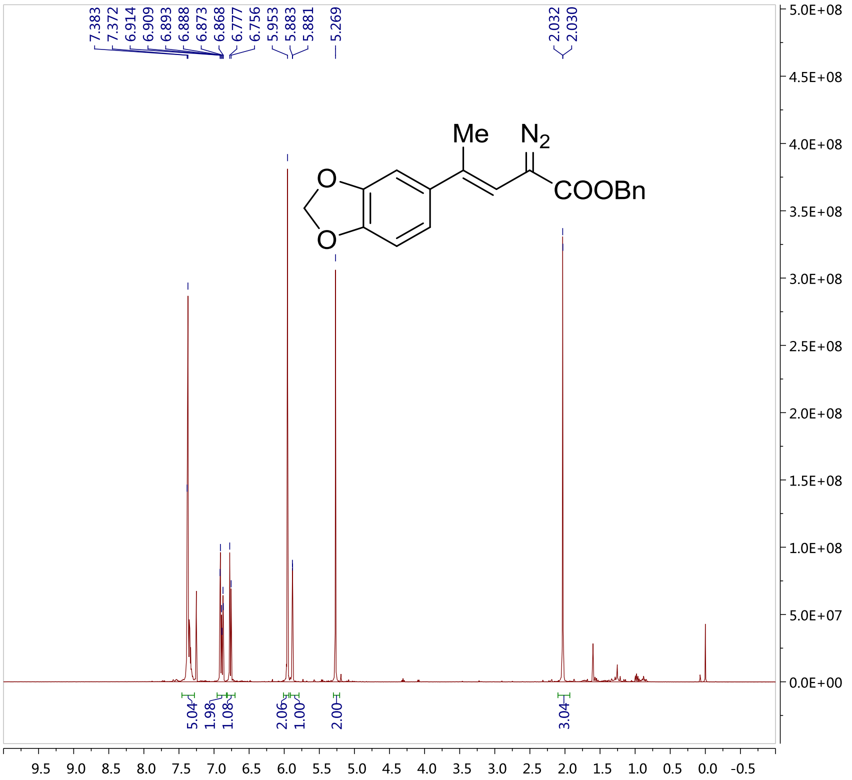

	Parameter	Value
1	Data File Name	Z:/ Data/ NMR/ 2013-4/ GJX-7-50/ 1/ pdata/ 1/ 1r
2	Title	GJX-7-50
3	Comment	PROTON
4	Origin	Bruker BioSpin GmbH
5	Owner	common
6	Site	
7	Spectrometer	spect
8	Author	
9	Solvent	CDCl3
10	Temperature	289. 4
11	Pulse Sequence	zg30
12	Experiment	1D
13	Number of Scans	8
14	Receiver Gain	161
15	Relaxation Delay	1. 0000
16	Pulse Width	13. 5000
17	Acquisition Time	2. 9999
18	Acquisition Date	2013-04-15T15:50:00
19	Modification Date	2013-04-15T15:51:00
20	Spectrometer Frequency	400. 13
21	Spectral Width	8223. 7
22	Lowest Frequency	-1654. 1
23	Nucleus	1H
24	Acquired Size	24670
25	Spectral Size	32768

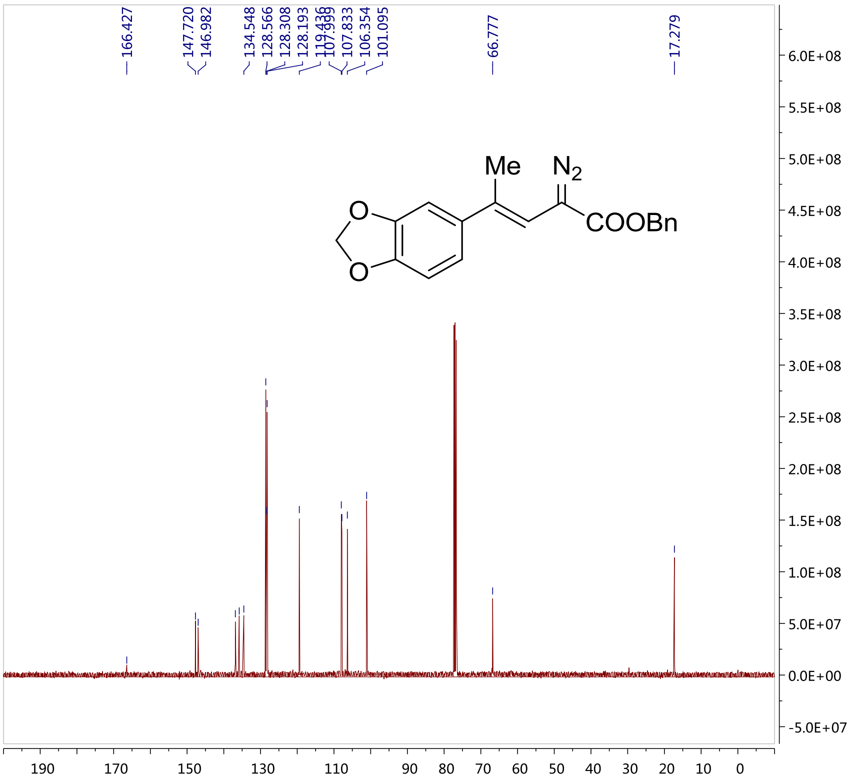

	Parameter	Value
1	Data File Name	Z:/ Data/ NMR/ 2013-4/ GJX-7-50-C/ 1/ pdata/ 1/ 1r
2	Title	GJX-7-50-C
3	Comment	C13CPD
4	Origin	Bruker BioSpin GmbH
5	Owner	common
6	Site	
7	Spectrometer	spect
8	Author	
9	Solvent	CDCl3
10	Temperature	290. 5
11	Pulse Sequence	zgpg30
12	Experiment	1D
13	Number of Scans	1024
14	Receiver Gain	203
15	Relaxation Delay	1. 0000
16	Pulse Width	12. 5000
17	Acquisition Time	0. 5506
18	Acquisition Date	2013-04-15T19:28:00
19	Modification Date	2013-04-15T19:50:00
20	Spectrometer Frequency	100. 61
21	Spectral Width	29761. 9
22	Lowest Frequency	-4820. 2
23	Nucleus	13C
24	Acquired Size	16384
25	Spectral Size	32768

(*E*)-Benzyl 2-diazo-4-(thiophen-2-yl)pent-3-enoate (1m)

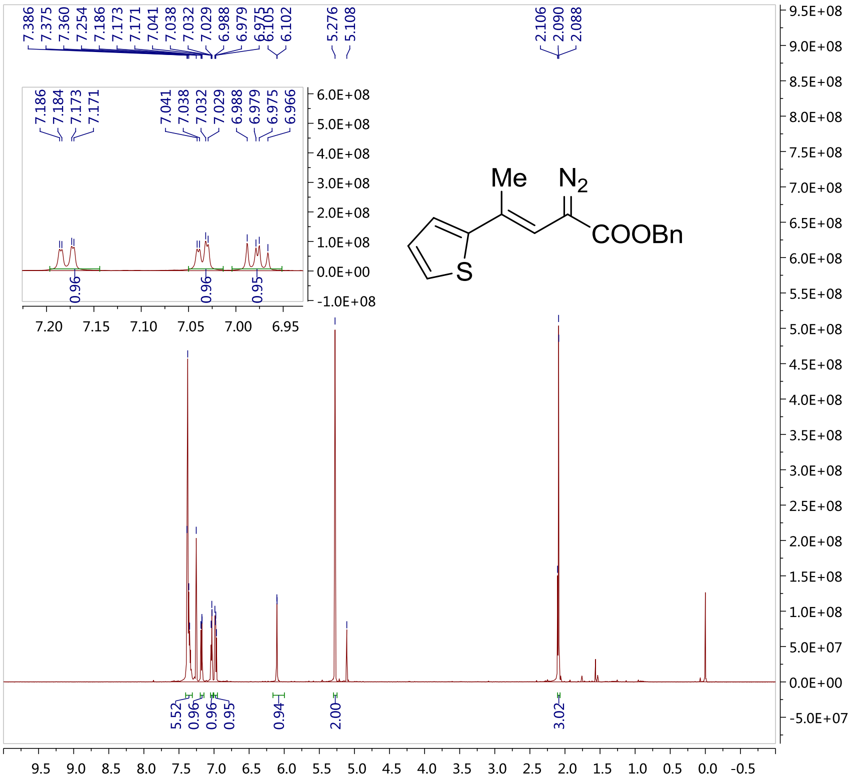

Parameter	Value
1 Data File Name	Z:/ Data/ NMR/ 2013-4/ GJX-8-11-2/ 1/ pdata/ 1/ 1r
2 Title	GJX-8-11-2
3 Comment	PROTON
4 Origin	Bruker BioSpin GmbH
5 Owner	common
6 Site	
7 Spectrometer	spect
8 Author	
9 Solvent	CDCl3
10 Temperature	295.4
11 Pulse Sequence	zg30
12 Experiment	1D
13 Number of Scans	8
14 Receiver Gain	203
15 Relaxation Delay	1.0000
16 Pulse Width	13.7000
17 Acquisition Time	1.9999
18 Acquisition Date	2013-04-27T09:18:00
19 Modification Date	2013-04-27T09:18:00
20 Spectrometer Frequency	400.13
21 Spectral Width	8223.7
22 Lowest Frequency	-1653.2
23 Nucleus	1H
24 Acquired Size	16446
25 Spectral Size	32768

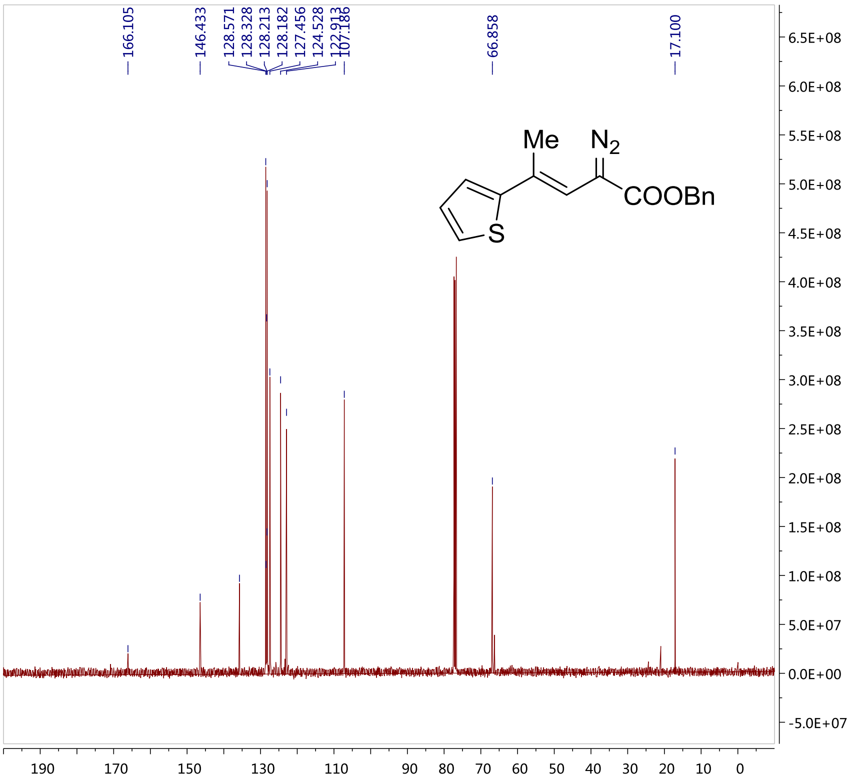

Parameter	Value
1 Data File Name	Z:/ Data/ NMR/ 2013-4/ GJX-8-11-C/ 1/ pdata/ 1/ 1r
2 Title	GJX-8-11-C
3 Comment	C13CPD
4 Origin	Bruker BioSpin GmbH
5 Owner	common
6 Site	
7 Spectrometer	spect
8 Author	
9 Solvent	CDCl3
10 Temperature	295.6
11 Pulse Sequence	zgpg30
12 Experiment	1D
13 Number of Scans	498
14 Receiver Gain	203
15 Relaxation Delay	1.0000
16 Pulse Width	12.0000
17 Acquisition Time	0.4588
18 Acquisition Date	2013-04-27T10:22:00
19 Modification Date	2013-04-27T10:34:00
20 Spectrometer Frequency	100.61
21 Spectral Width	35714.3
22 Lowest Frequency	-7796.3
23 Nucleus	13C
24 Acquired Size	16384
25 Spectral Size	32768

Benzyl 2-diazo-4-methylpent-3-enoate (1n)

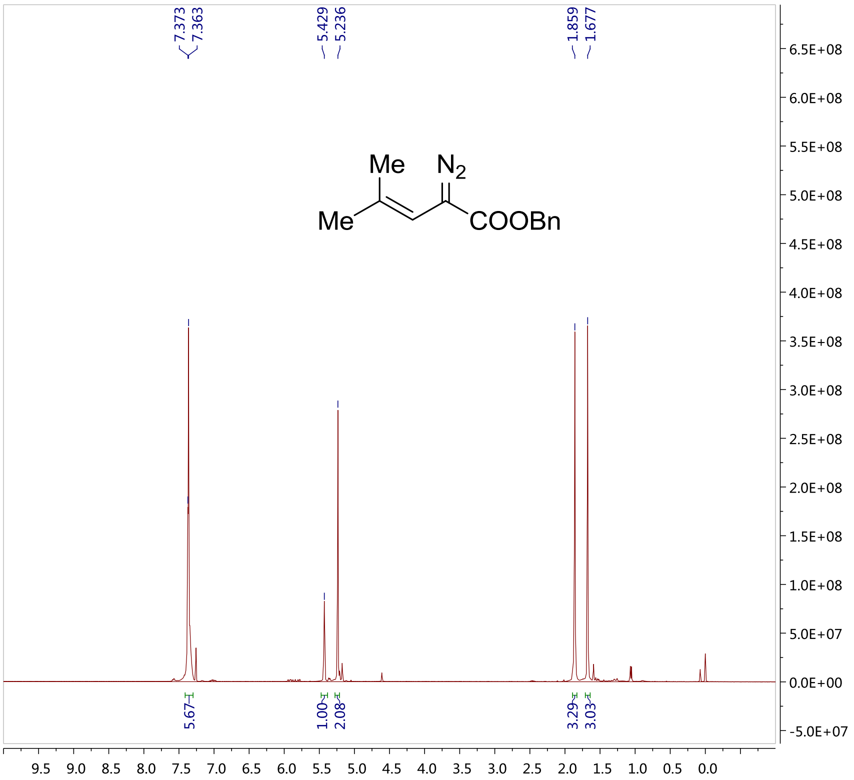

Parameter	Value
1 Data File Name	Z:/ Data/ NMR/ 2013-3/ GJX-7-15-C/ 1/ pdata/ 1/ 1r
2 Title	GJX-7-15-C
3 Comment	PROTON
4 Origin	Bruker BioSpin GmbH
5 Owner	common
6 Site	
7 Spectrometer	spect
8 Author	
9 Solvent	CDCl3
10 Temperature	289.7
11 Pulse Sequence	zg30
12 Experiment	1D
13 Number of Scans	8
14 Receiver Gain	101
15 Relaxation Delay	1.0000
16 Pulse Width	13.5000
17 Acquisition Time	1.9923
18 Acquisition Date	2013-03-29T10:51:00
19 Modification Date	2013-03-29T10:51:00
20 Spectrometer Frequency	400.23
21 Spectral Width	8223.7
22 Lowest Frequency	-1640.3
23 Nucleus	1H
24 Acquired Size	16384
25 Spectral Size	32768

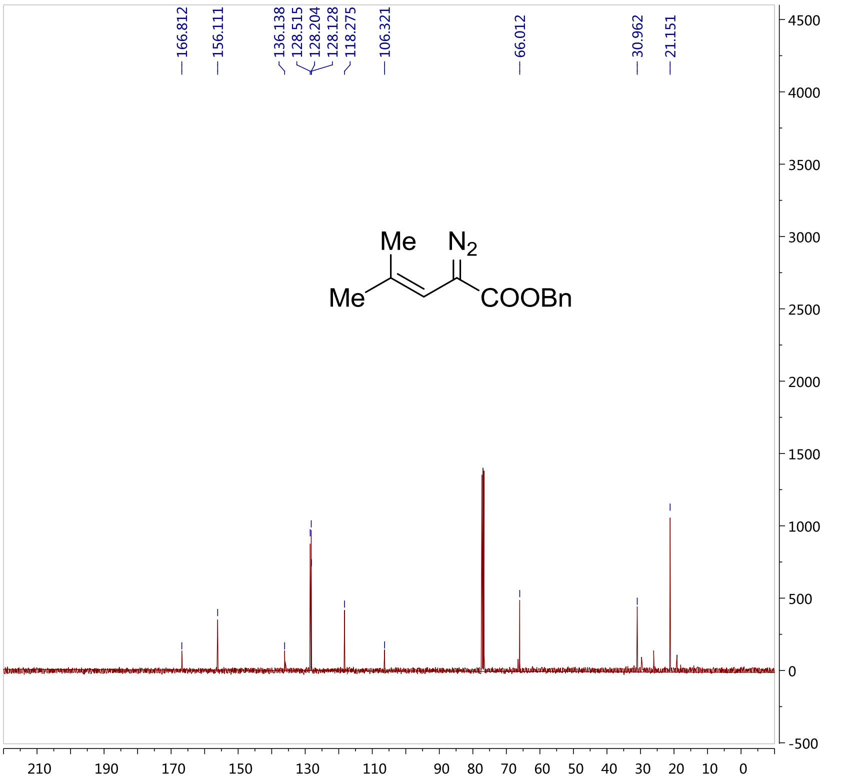

Parameter	Value
1 Data File Name	Z:/ Data/ NMR/ 2013-12/ gjx-20131225/ 2/ fid
2 Title	gjx-20131225
3 Comment	C13CPD
4 Origin	Bruker BioSpin GmbH
5 Owner	common
6 Site	
7 Spectrometer	spect
8 Author	
9 Solvent	CDCl3
10 Temperature	292.5
11 Pulse Sequence	zgpg30
12 Experiment	1D
13 Number of Scans	247
14 Receiver Gain	203
15 Relaxation Delay	1.0000
16 Pulse Width	12.5000
17 Acquisition Time	0.5506
18 Acquisition Date	2013-12-25T10:38:00
19 Modification Date	2013-12-25T10:44:00
20 Spectrometer Frequency	100.61
21 Spectral Width	29761.9
22 Lowest Frequency	-4820.2
23 Nucleus	13C
24 Acquired Size	16384
25 Spectral Size	32768

Benzyl 2-diazobut-3-enoate (1o)

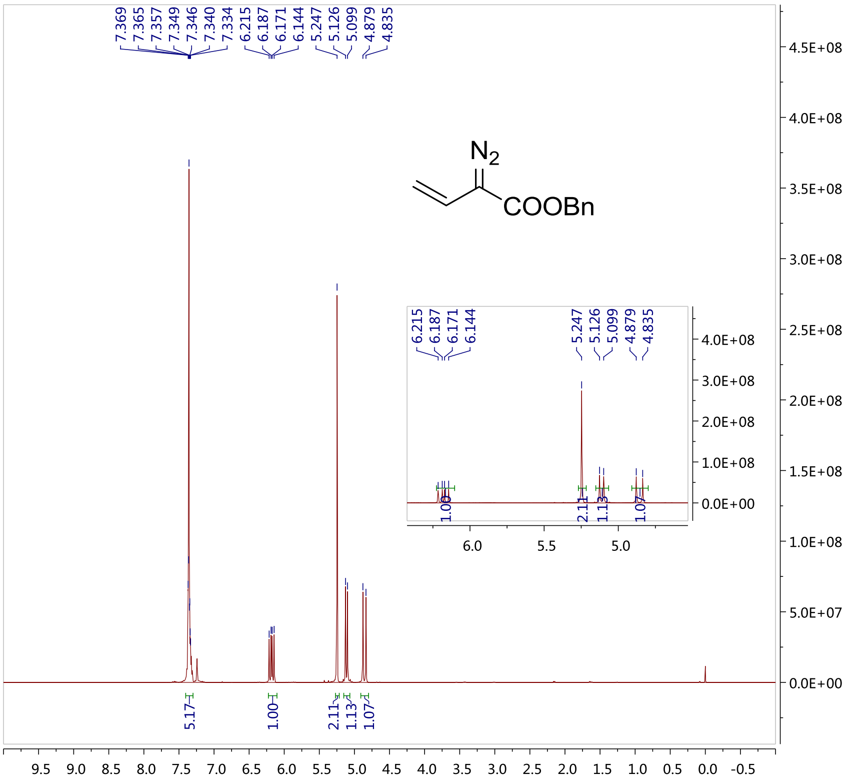

Parameter	Value
1 Data File Name	Z:/ Data/ NMR/ 2013-3/ GJX-7-2-C/ 1/ pdata/ 1/ 1r
2 Title	GJX-7-2-C
3 Comment	PROTON
4 Origin	Bruker BioSpin GmbH
5 Owner	common
6 Site	
7 Spectrometer	spect
8 Author	
9 Solvent	CDCl3
10 Temperature	290.5
11 Pulse Sequence	zg30
12 Experiment	1D
13 Number of Scans	8
14 Receiver Gain	90
15 Relaxation Delay	1.0000
16 Pulse Width	13.5000
17 Acquisition Time	2.9999
18 Acquisition Date	2013-03-22T17:09:00
19 Modification Date	2013-03-22T17:10:00
20 Spectrometer Frequency	400.13
21 Spectral Width	8223.7
22 Lowest Frequency	-1658.2
23 Nucleus	1H
24 Acquired Size	24670
25 Spectral Size	32768

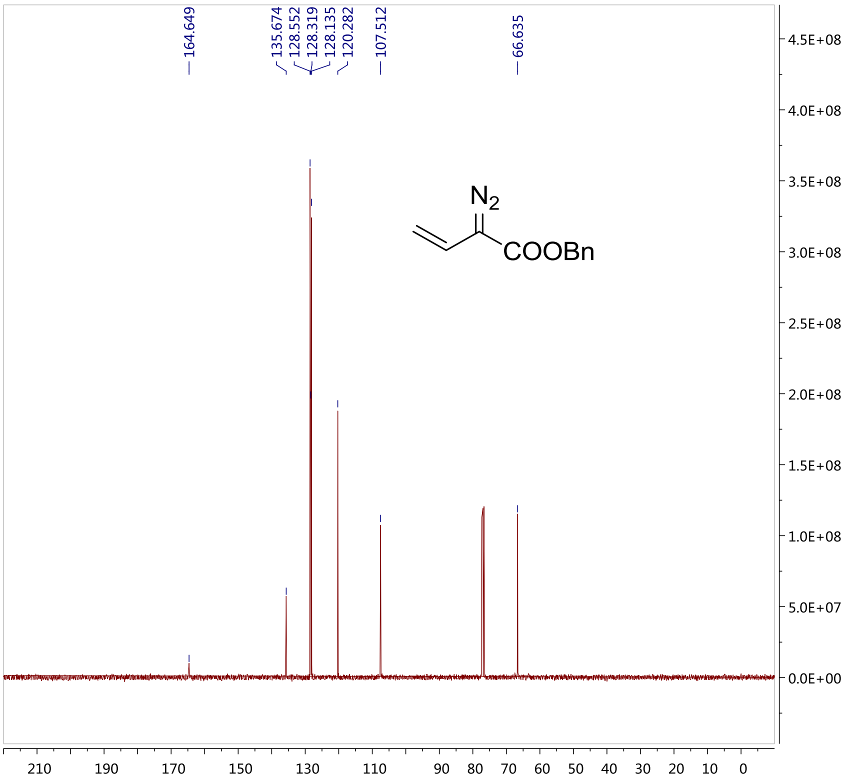

Parameter	Value
1 Data File Name	Z:/ Data/ NMR/ 2013-3/ GJX-7-2-C/ 2/ pdata/ 1/ 1r
2 Title	GJX-7-2-C
3 Comment	C13CPD
4 Origin	Bruker BioSpin GmbH
5 Owner	common
6 Site	
7 Spectrometer	spect
8 Author	
9 Solvent	CDCl3
10 Temperature	290.5
11 Pulse Sequence	zgpg30
12 Experiment	1D
13 Number of Scans	461
14 Receiver Gain	203
15 Relaxation Delay	1.0000
16 Pulse Width	12.5000
17 Acquisition Time	0.5506
18 Acquisition Date	2013-03-22T17:11:00
19 Modification Date	2013-03-22T17:23:00
20 Spectrometer Frequency	100.61
21 Spectral Width	29761.9
22 Lowest Frequency	-4820.2
23 Nucleus	13C
24 Acquired Size	16384
25 Spectral Size	32768

(*E*)-Benzyl 5-(benzyloxy)-2-diazopent-3-enoate (1q)

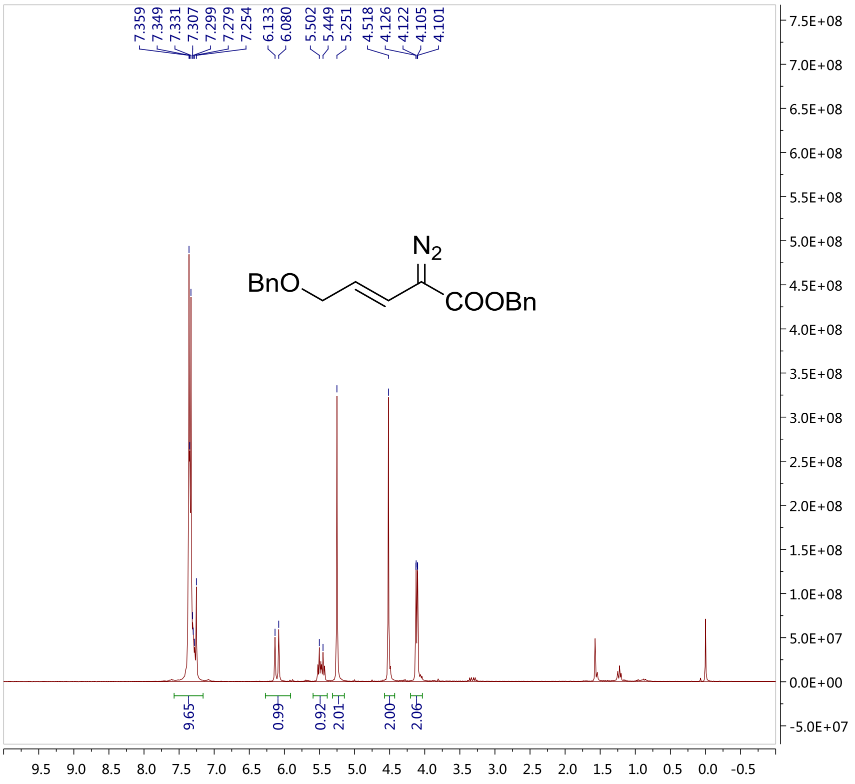

Parameter	Value
1 Data File Name	Z:/ Data/ NMR/ 2013-9/ GJX-9-32-2/ 1/ pdata/ 1/ 1r
2 Title	GJX-9-32-2
3 Comment	PROTON
4 Origin	Bruker BioSpin GmbH
5 Owner	common
6 Site	
7 Spectrometer	spect
8 Author	
9 Solvent	CDCl3
10 Temperature	295.9
11 Pulse Sequence	zg30
12 Experiment	1D
13 Number of Scans	8
14 Receiver Gain	575
15 Relaxation Delay	1.0000
16 Pulse Width	12.1000
17 Acquisition Time	2.6543
18 Acquisition Date	2013-09-03T16:26:00
19 Modification Date	2013-09-03T16:26:00
20 Spectrometer Frequency	300.13
21 Spectral Width	6172.8
22 Lowest Frequency	-1241.3
23 Nucleus	1H
24 Acquired Size	16384
25 Spectral Size	32768

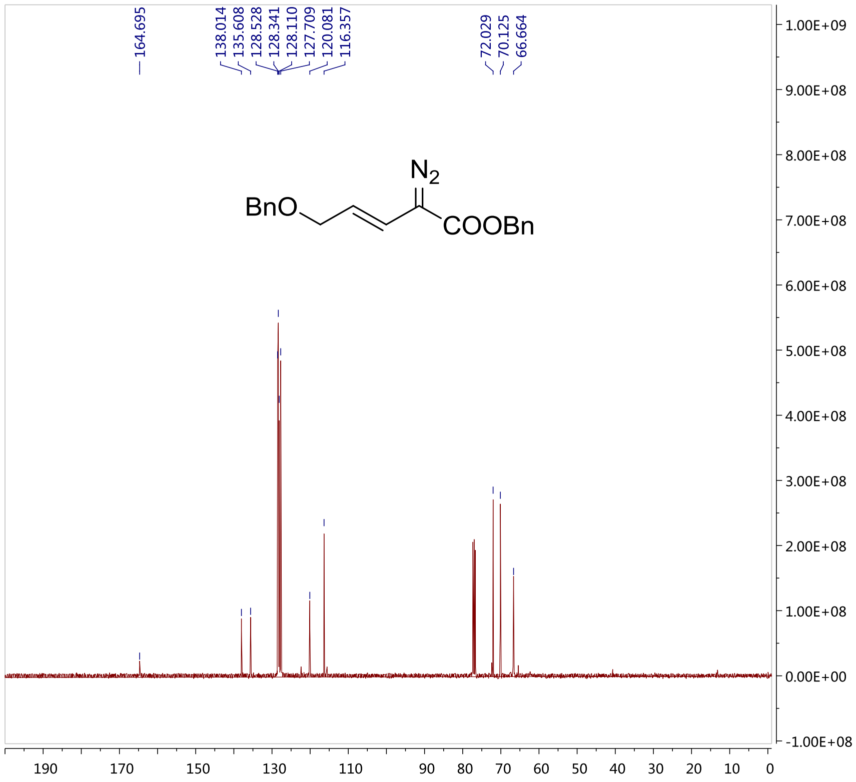

Parameter	Value
1 Data File Name	Z:/ Data/ NMR/ 2013-9/ GJX-9-32-C/ 2/ pdata/ 1/ 1r
2 Title	GJX-9-32-C
3 Comment	C13CPD
4 Origin	Bruker BioSpin GmbH
5 Owner	common
6 Site	
7 Spectrometer	spect
8 Author	
9 Solvent	CDCl3
10 Temperature	291.0
11 Pulse Sequence	zgpg30
12 Experiment	1D
13 Number of Scans	406
14 Receiver Gain	203
15 Relaxation Delay	1.0000
16 Pulse Width	12.5000
17 Acquisition Time	0.5506
18 Acquisition Date	2013-09-04T09:53:00
19 Modification Date	2013-09-04T10:03:00
20 Spectrometer Frequency	100.61
21 Spectral Width	29761.9
22 Lowest Frequency	-4820.2
23 Nucleus	13C
24 Acquired Size	16384
25 Spectral Size	32768

(*E*)-Methyl -2-diazo-4-phenylpent-3-enoate (1r)

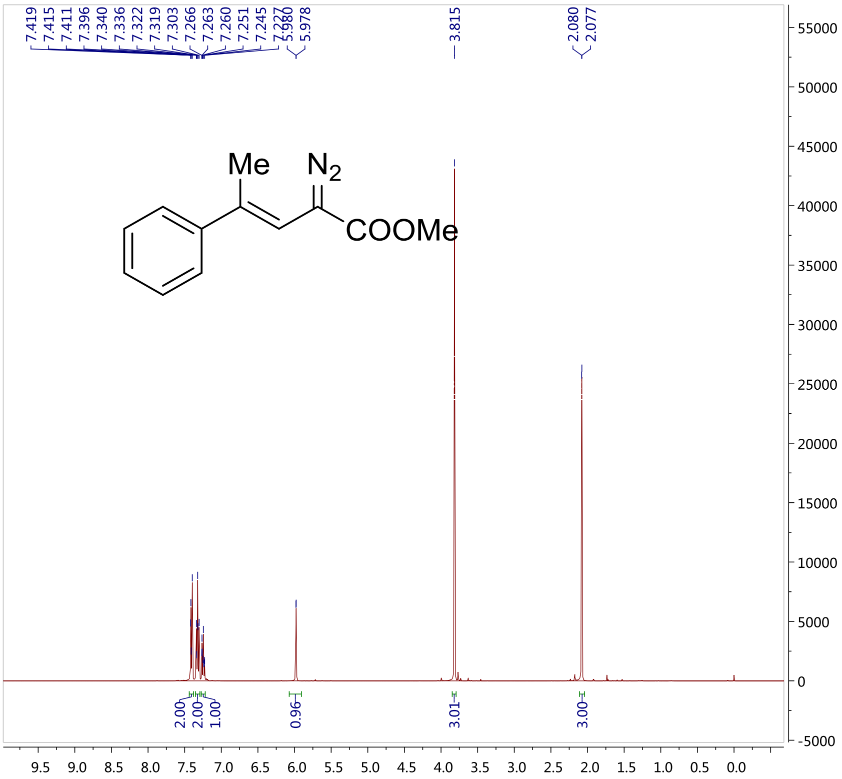

	Parameter	Value
1	Data File Name	Z:/ Data/ NMR/ 2015-04/ zt-3-123-sm/ 1/ fid
2	Title	zt-3-123-sm
3	Comment	PROTON
4	Origin	Bruker BioSpin GmbH
5	Owner	common
6	Site	
7	Spectrometer	spect
8	Author	
9	Solvent	CDCl3
10	Temperature	1155.1
11	Pulse Sequence	zg30
12	Experiment	1D
13	Number of Scans	8
14	Receiver Gain	57
15	Relaxation Delay	1.0000
16	Pulse Width	13.5000
17	Acquisition Time	1.9923
18	Acquisition Date	2015-04-14T11:41:00
19	Modification Date	2015-04-14T11:41:00
20	Spectrometer Frequency	400.23
21	Spectral Width	8223.7
22	Lowest Frequency	-1640.3
23	Nucleus	1H
24	Acquired Size	16384
25	Spectral Size	32768

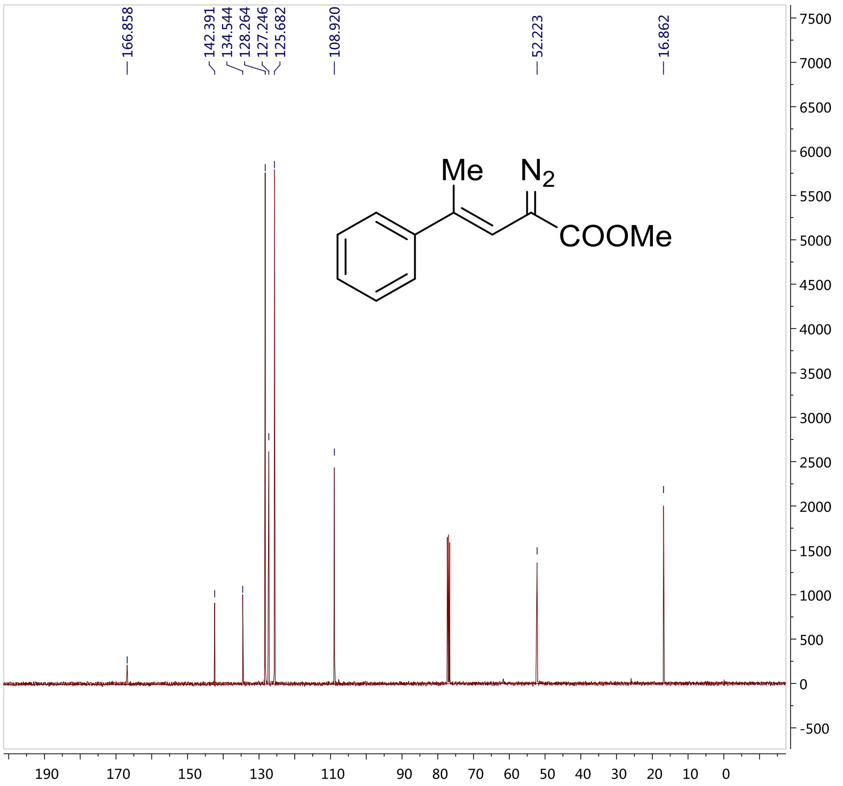

	Parameter	Value
1	Data File Name	Z:/ Data/ NMR/ 2015-04/ zt-3-123-sm/ 2/ fid
2	Title	zt-3-123-sm
3	Comment	C13CPD
4	Origin	Bruker BioSpin GmbH
5	Owner	common
6	Site	
7	Spectrometer	spect
8	Author	
9	Solvent	CDCl3
10	Temperature	1155.2
11	Pulse Sequence	zgpg30
12	Experiment	1D
13	Number of Scans	319
14	Receiver Gain	203
15	Relaxation Delay	1.0000
16	Pulse Width	12.0000
17	Acquisition Time	0.5506
18	Acquisition Date	2015-04-14T11:43:00
19	Modification Date	2015-04-14T11:51:00
20	Spectrometer Frequency	100.64
21	Spectral Width	29761.9
22	Lowest Frequency	-4817.6
23	Nucleus	13C
24	Acquired Size	16384
25	Spectral Size	32768

(*E*)-*tert*-Butyl -2-diazo-4-phenylpent-3-enoate (1s)

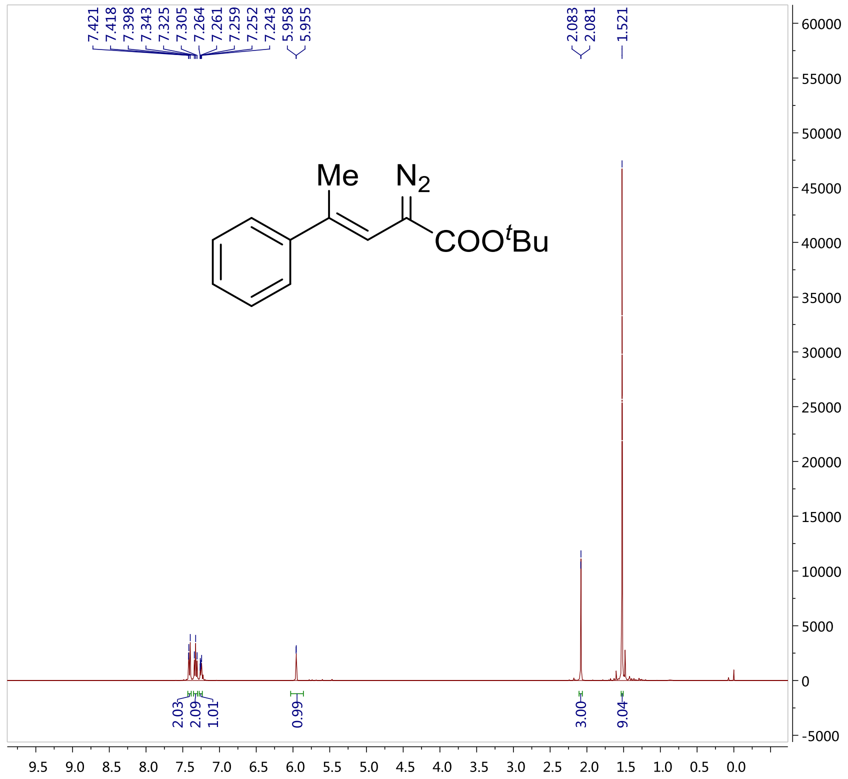

Parameter		Value
1	Data File Name	Z:/ Data/ NMR/ 2015-04/ zt-3-20150429-c/ 1/ fid
2	Title	zt-3-20150429-c
3	Comment	PROTON
4	Origin	Bruker BioSpin GmbH
5	Owner	common
6	Site	
7	Spectrometer	spect
8	Author	
9	Solvent	CDCl3
10	Temperature	1190. 1
11	Pulse Sequence	zg30
12	Experiment	1D
13	Number of Scans	8
14	Receiver Gain	101
15	Relaxation Delay	1. 0000
16	Pulse Width	13. 5000
17	Acquisition Time	1. 9923
18	Acquisition Date	2015-04-29T10:58:00
19	Modification Date	2015-04-29T10:58:00
20	Spectrometer Frequency	400. 23
21	Spectral Width	8223. 7
22	Lowest Frequency	-1640. 3
23	Nucleus	1H
24	Acquired Size	16384
25	Spectral Size	32768

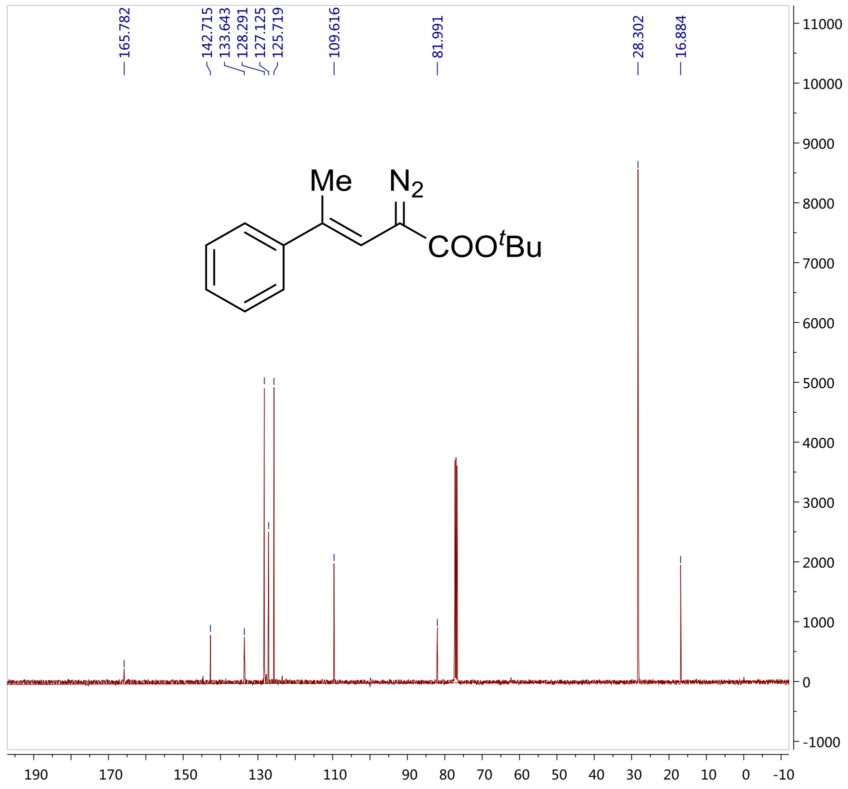

Parameter		Value
1	Data File Name	Z:/ Data/ NMR/ 2015-04/ zt-3-20150429-c/ 2/ fid
2	Title	zt-3-20150429-c
3	Comment	C13CPD
4	Origin	Bruker BioSpin GmbH
5	Owner	common
6	Site	
7	Spectrometer	spect
8	Author	
9	Solvent	CDCl3
10	Temperature	1192. 9
11	Pulse Sequence	zgpg30
12	Experiment	1D
13	Number of Scans	1024
14	Receiver Gain	203
15	Relaxation Delay	1. 0000
16	Pulse Width	12. 0000
17	Acquisition Time	0. 5506
18	Acquisition Date	2015-04-29T10:59:00
19	Modification Date	2015-04-29T11:26:00
20	Spectrometer Frequency	100. 64
21	Spectral Width	29761. 9
22	Lowest Frequency	-4817. 6
23	Nucleus	13C
24	Acquired Size	16384
25	Spectral Size	32768

(*E*)-Benzyl 2-(*tert*-butoxycarbonylamino)pent-3-enoate (2a)

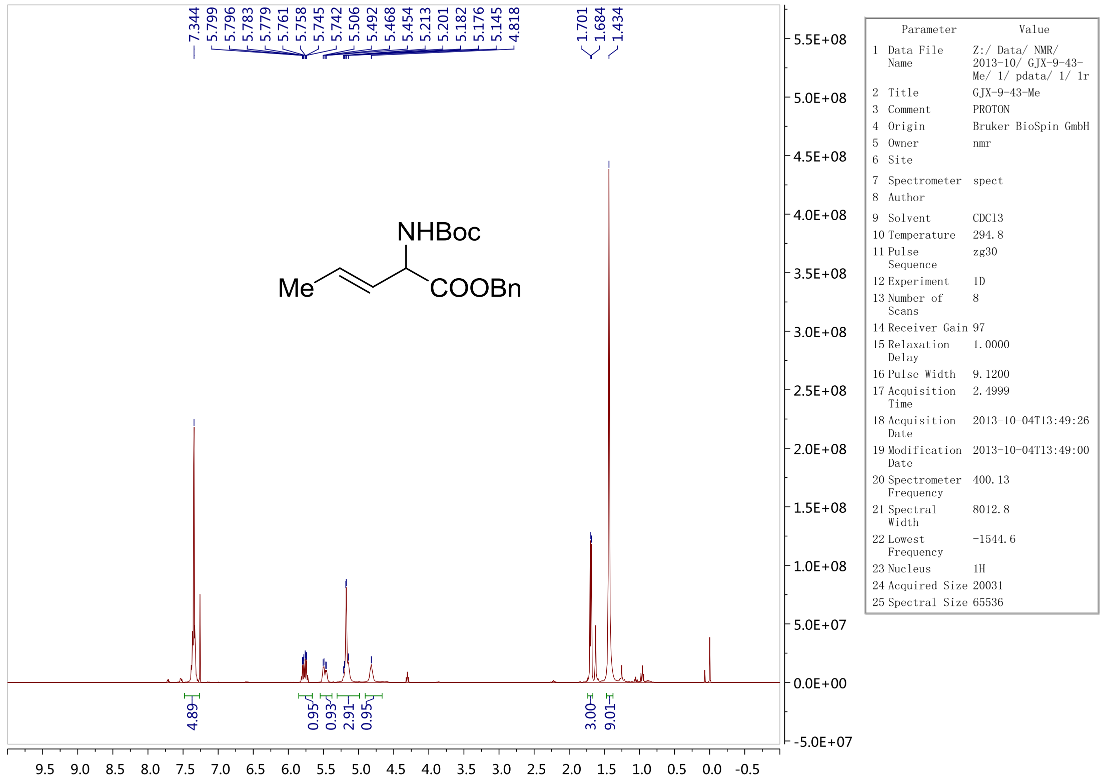

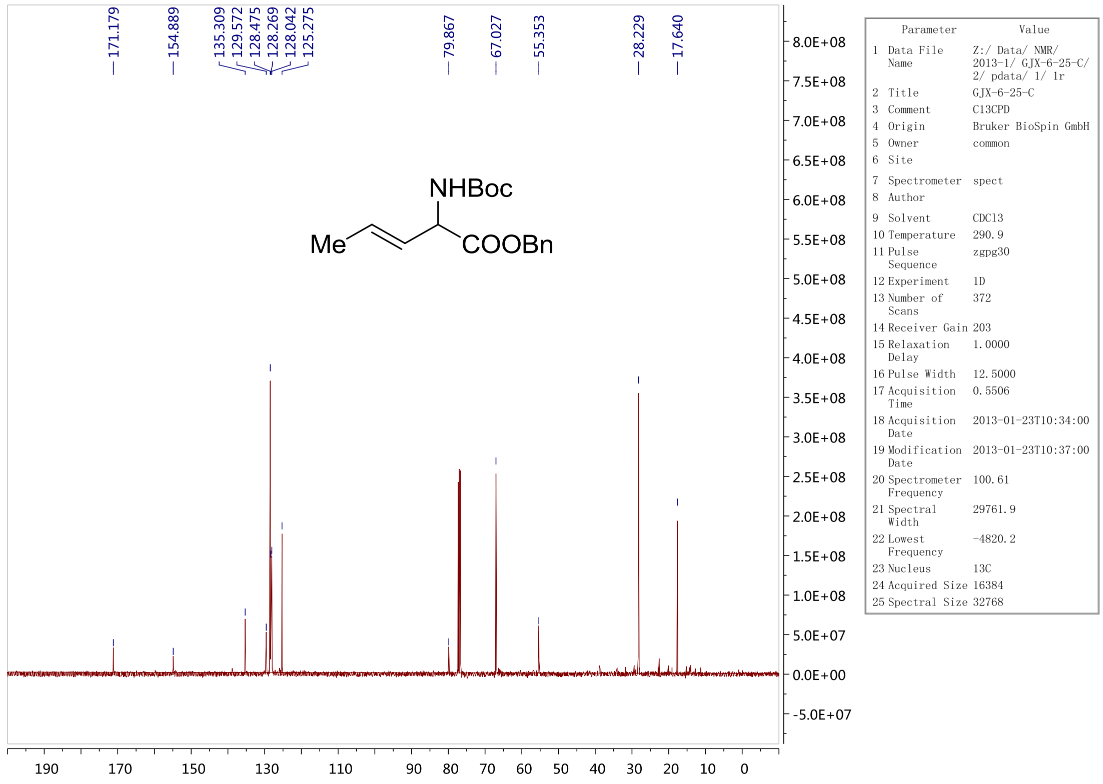

(*E*)-Benzyl 2-(*tert*-butoxycarbonylamino)hex-3-enoate (2b)

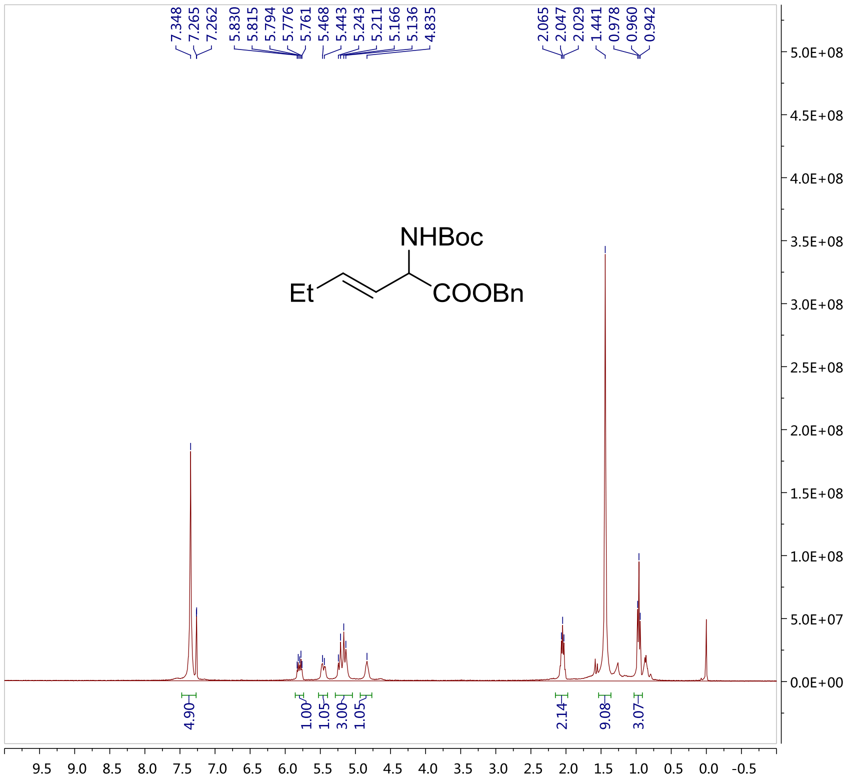

Parameter	Value
1 Data File Name	Z:/ Data/ NMR/ 2013-3/ GJX-6-48-H/ 1/ pdata/ 1/ 1r
2 Title	GJX-6-48-H
3 Comment	PROTON
4 Origin	Bruker BioSpin GmbH
5 Owner	common
6 Site	
7 Spectrometer	spect
8 Author	
9 Solvent	CDCl3
10 Temperature	290. 1
11 Pulse Sequence	zg30
12 Experiment	1D
13 Number of Scans	8
14 Receiver Gain	128
15 Relaxation Delay	1. 0000
16 Pulse Width	13. 5000
17 Acquisition Time	1. 9923
18 Acquisition Date	2013-03-07T14:50:00
19 Modification Date	2013-03-07T14:50:00
20 Spectrometer Frequency	400. 23
21 Spectral Width	8223. 7
22 Lowest Frequency	-1640. 3
23 Nucleus	1H
24 Acquired Size	16384
25 Spectral Size	32768

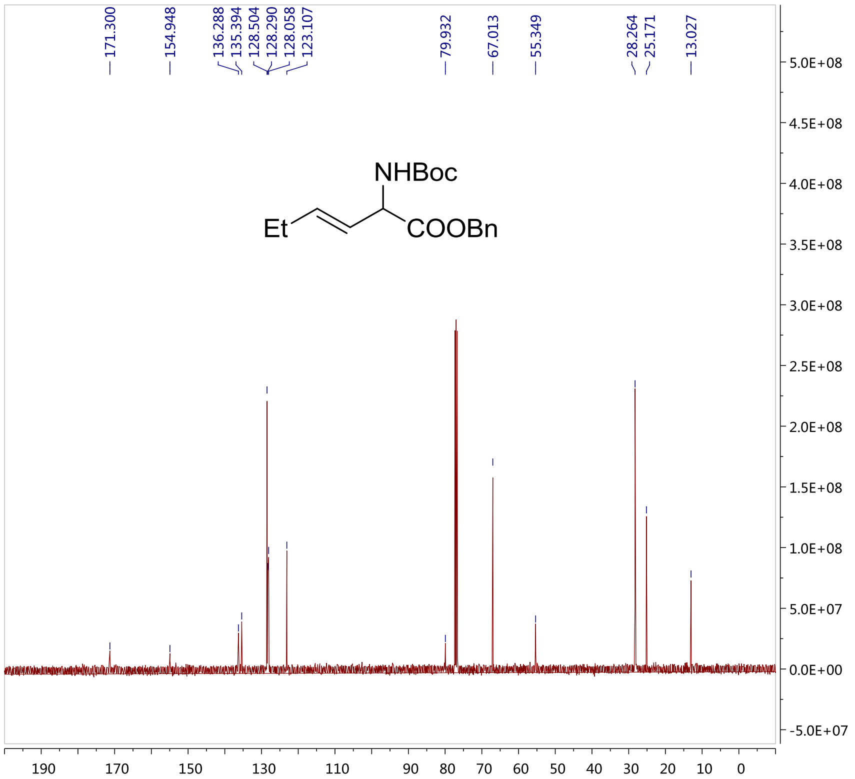

Parameter	Value
1 Data File Name	Z:/ Data/ NMR/ 2013-3/ GJX-6-48-C/ 1/ pdata/ 1/ 1r
2 Title	GJX-6-48-C
3 Comment	C13CPD
4 Origin	Bruker BioSpin GmbH
5 Owner	common
6 Site	
7 Spectrometer	spect
8 Author	
9 Solvent	CDCl3
10 Temperature	291. 1
11 Pulse Sequence	zgpg30
12 Experiment	1D
13 Number of Scans	492
14 Receiver Gain	203
15 Relaxation Delay	1. 0000
16 Pulse Width	12. 0000
17 Acquisition Time	0. 5506
18 Acquisition Date	2013-03-07T15:03:00
19 Modification Date	2013-03-07T15:09:00
20 Spectrometer Frequency	100. 64
21 Spectral Width	29761. 9
22 Lowest Frequency	-4817. 6
23 Nucleus	13C
24 Acquired Size	16384
25 Spectral Size	32768

(*E*)-Benzyl 2-(*tert*-butoxycarbonylamino)oct-3-enoate (2c)

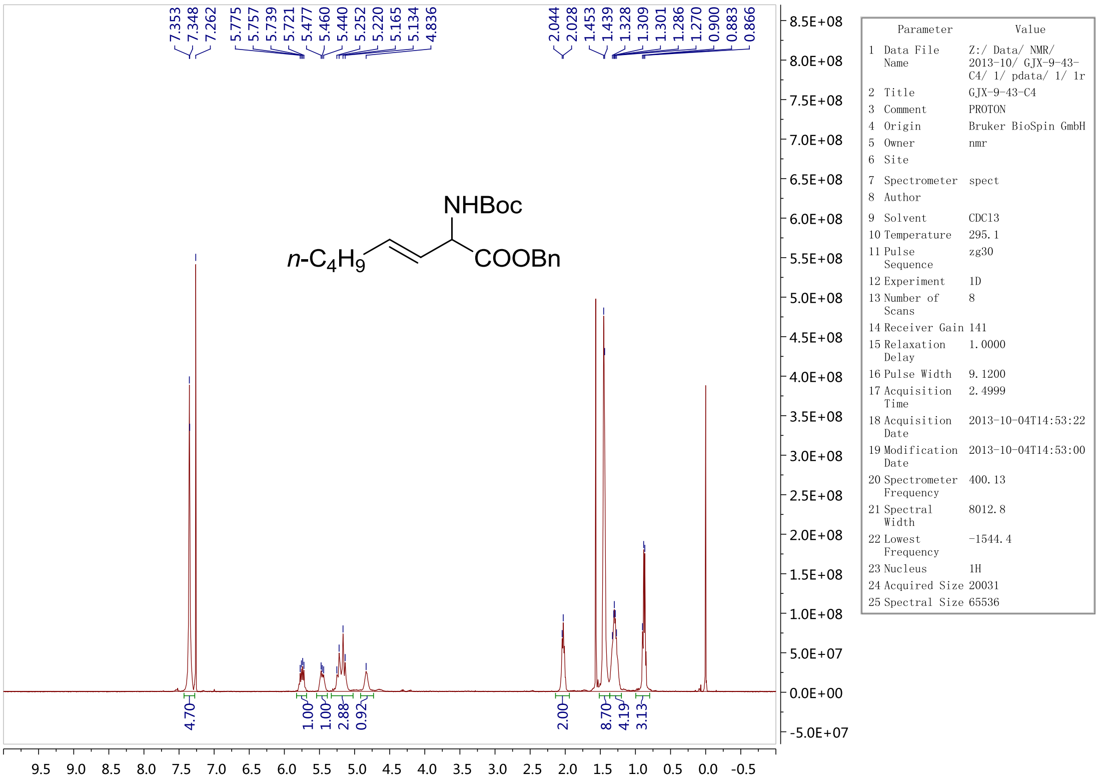

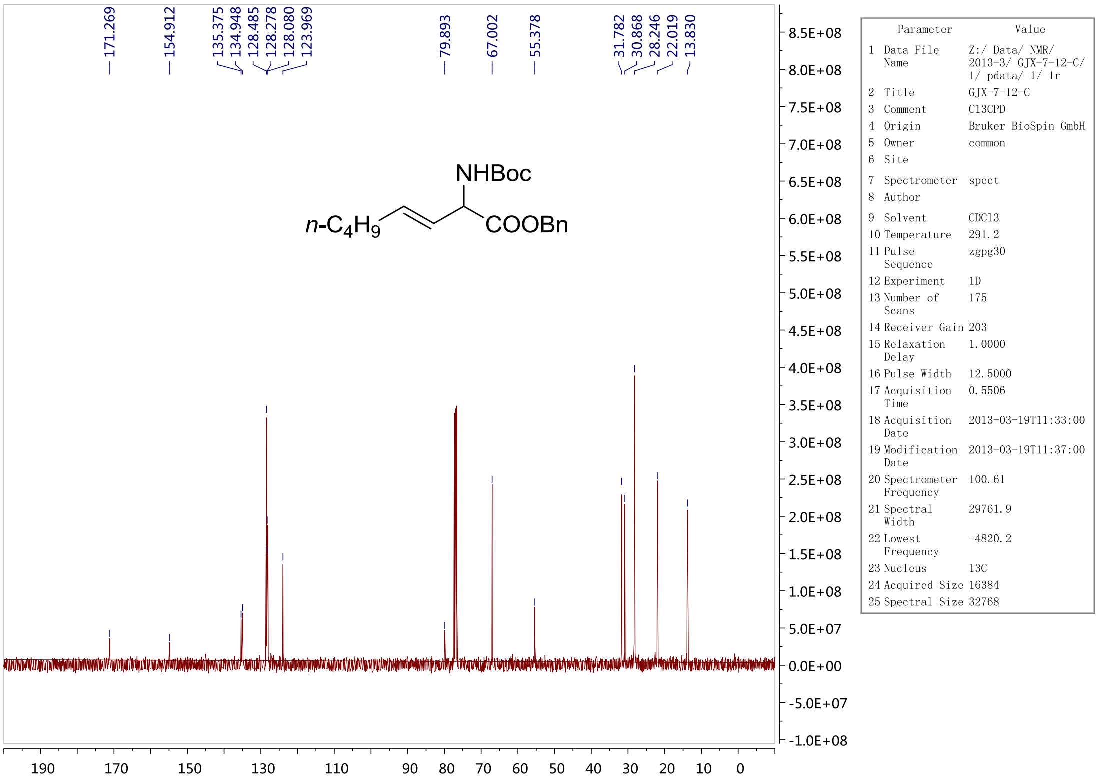

(*E*)-Benzyl 2-(*tert*-butoxycarbonylamino)dec-3-enoate (2d)

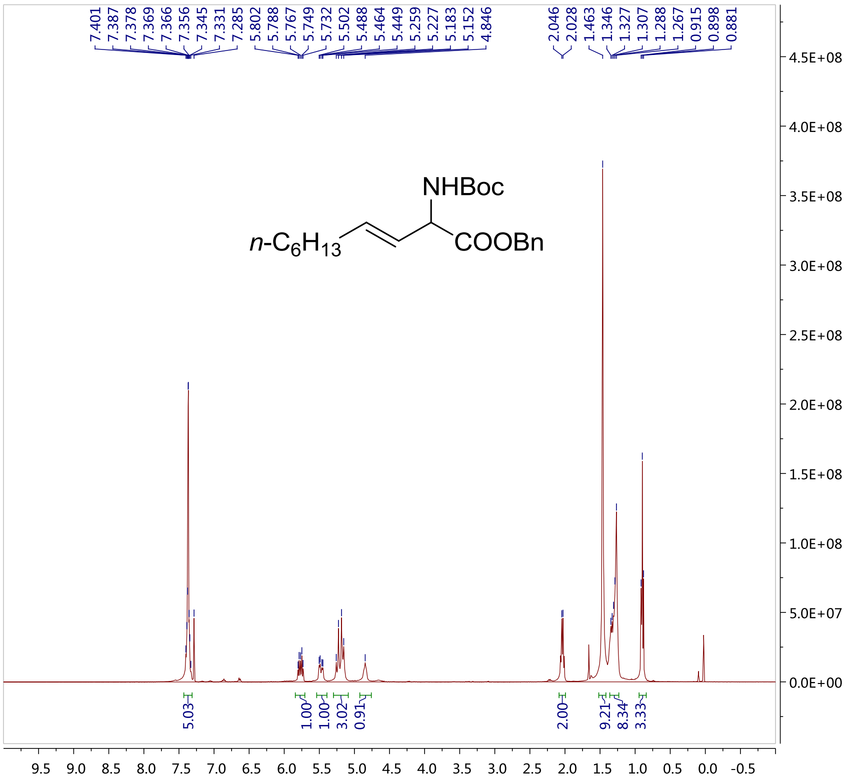

Parameter	Value
1 Data File Name	Z:/ Data/ NMR/ 2013-3/ GJX-6-45-H/ 1/ pdata/ 1/ 1r
2 Title	GJX-6-45-H
3 Comment	PROTON
4 Origin	Bruker BioSpin GmbH
5 Owner	common
6 Site	
7 Spectrometer	spect
8 Author	
9 Solvent	CDCl3
10 Temperature	294.9
11 Pulse Sequence	zg30
12 Experiment	1D
13 Number of Scans	8
14 Receiver Gain	90
15 Relaxation Delay	1.0000
16 Pulse Width	13.7000
17 Acquisition Time	1.9999
18 Acquisition Date	2013-03-05T18:32:00
19 Modification Date	2013-03-05T18:32:00
20 Spectrometer Frequency	400.13
21 Spectral Width	8223.7
22 Lowest Frequency	-1640.9
23 Nucleus	1H
24 Acquired Size	16446
25 Spectral Size	32768

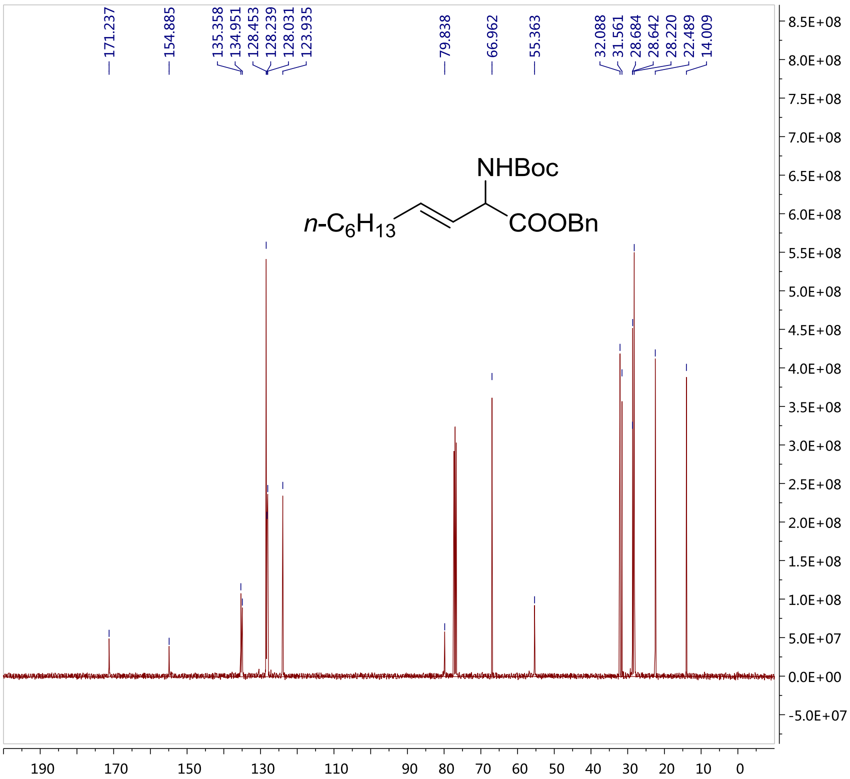

Parameter	Value
1 Data File Name	Z:/ Data/ NMR/ 2013-3/ GJX-6-45-C/ 1/ pdata/ 1/ 1r
2 Title	GJX-6-45-C
3 Comment	C13CPD
4 Origin	Bruker BioSpin GmbH
5 Owner	common
6 Site	
7 Spectrometer	spect
8 Author	
9 Solvent	CDCl3
10 Temperature	295.5
11 Pulse Sequence	zgpg30
12 Experiment	1D
13 Number of Scans	346
14 Receiver Gain	203
15 Relaxation Delay	1.0000
16 Pulse Width	12.0000
17 Acquisition Time	0.4588
18 Acquisition Date	2013-03-05T18:37:00
19 Modification Date	2013-03-05T18:44:00
20 Spectrometer Frequency	100.61
21 Spectral Width	35714.3
22 Lowest Frequency	-7796.3
23 Nucleus	13C
24 Acquired Size	16384
25 Spectral Size	32768

(*E*)-Benzyl 2-(*tert*-butoxycarbonylamino)-5-methylhex-3-enoate (2e)

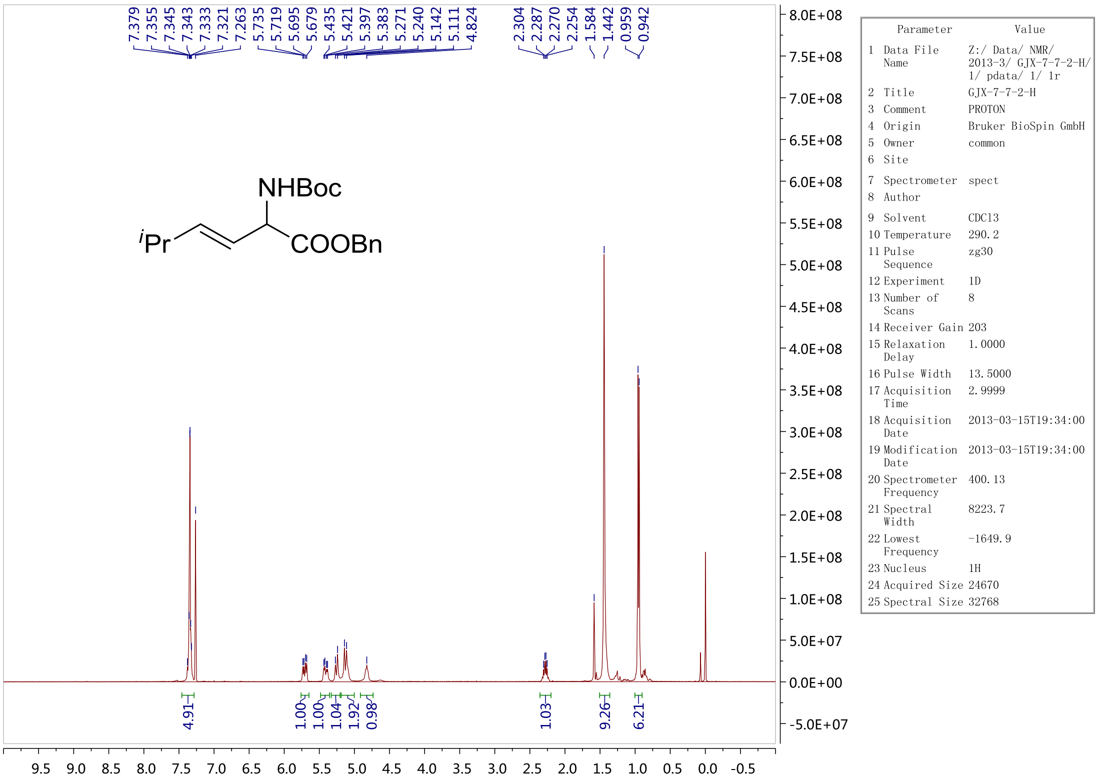

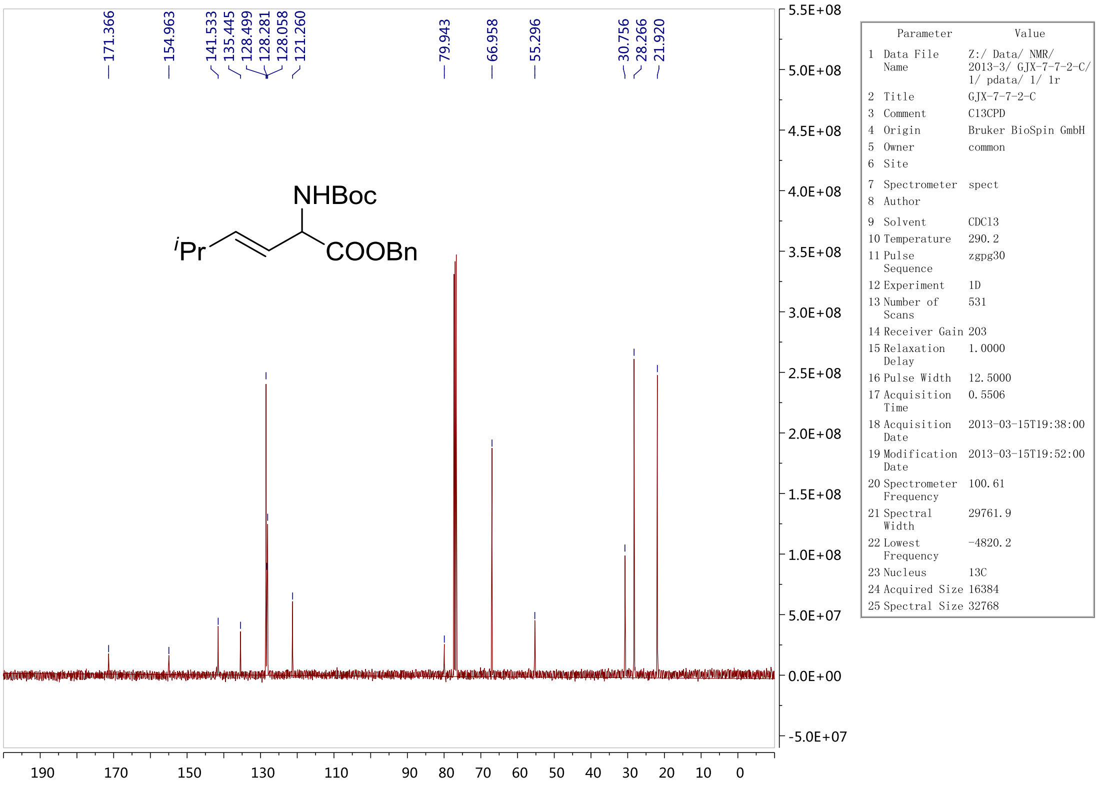

(*E*)-benzyl 2-(*tert*-butoxycarbonylamino)-4-phenylbut-3-enoate (2f)

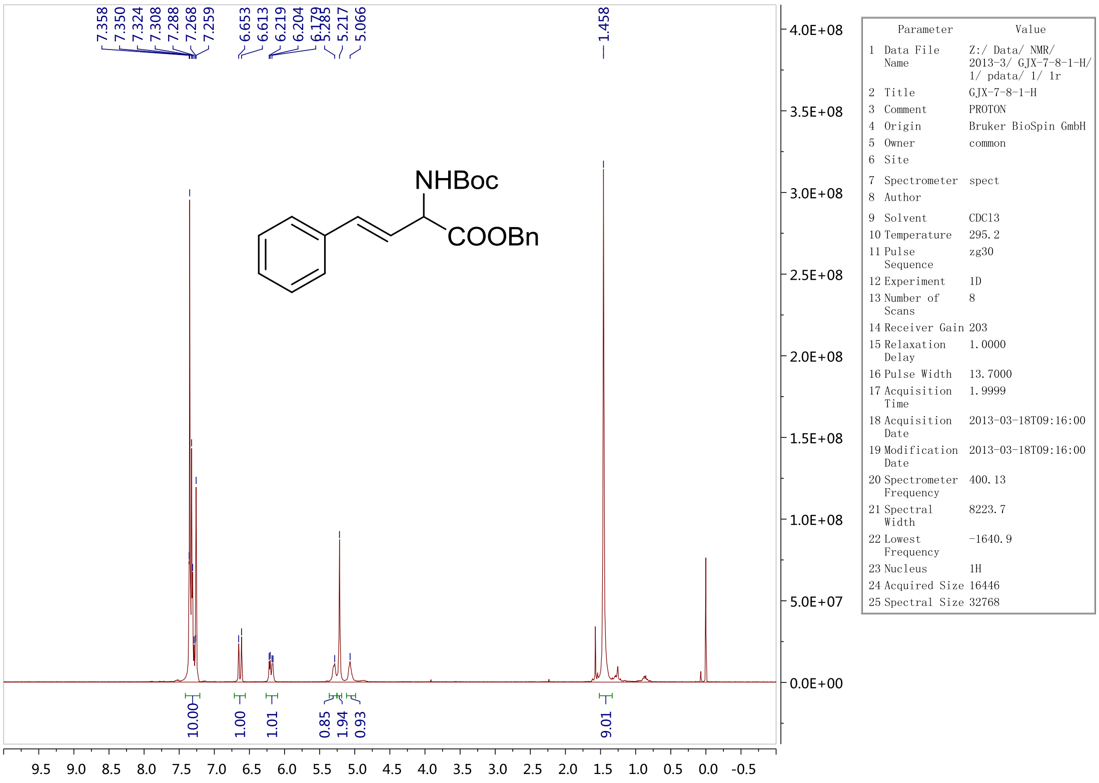

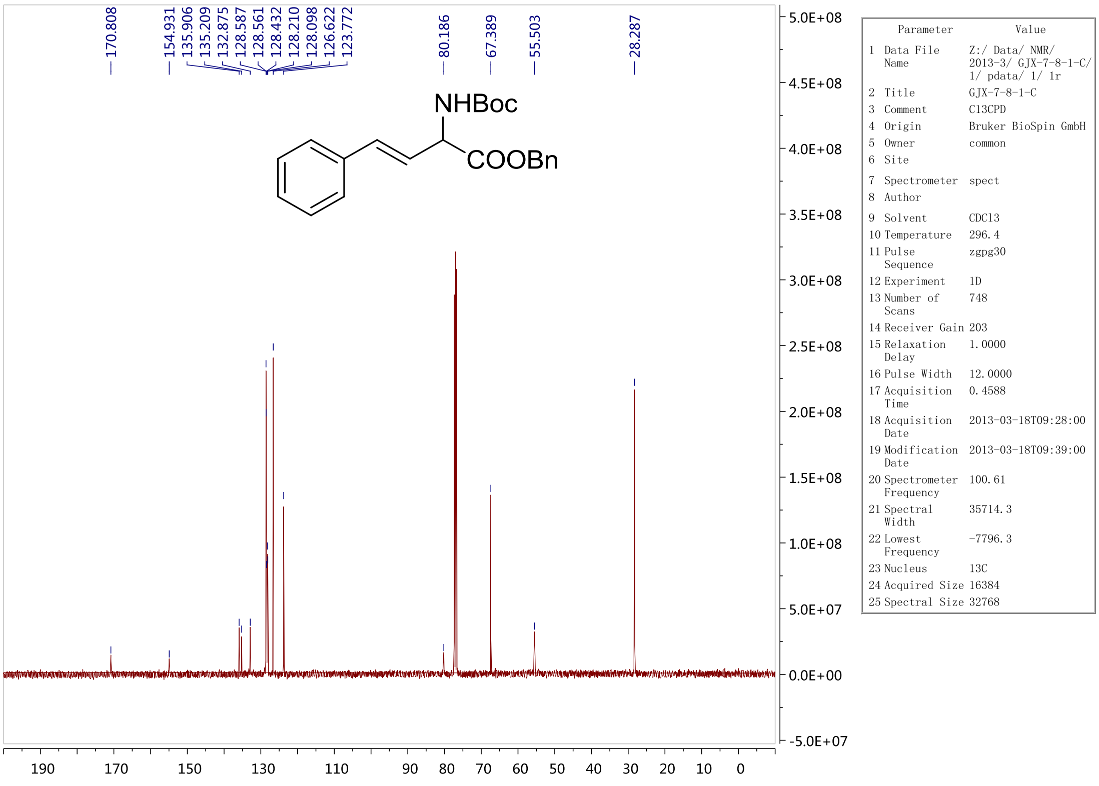

(*E*)-Benzyl 2-(*tert*-butoxycarbonylamino)-4-phenylpent-3-enoate (2g)

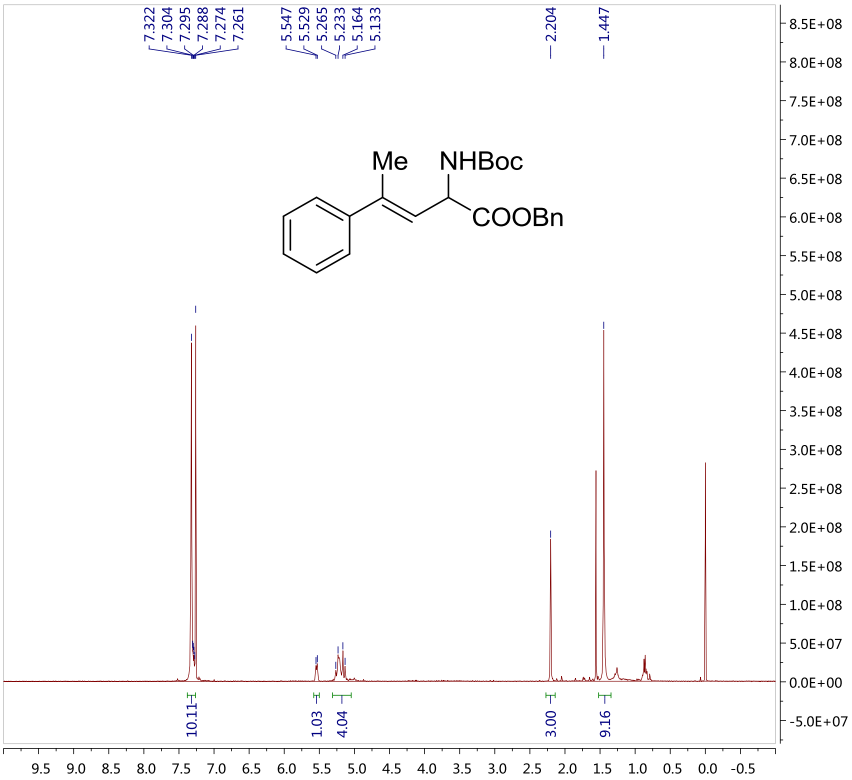

Parameter	Value
1 Data File Name	Z:/ Data/ NMR/ 2013-1/ GJX-6-22-2/ 1/ pdata/ 1/ 1r
2 Title	GJX-6-22-2
3 Comment	PROTON
4 Origin	Bruker BioSpin GmbH
5 Owner	common
6 Site	
7 Spectrometer	spect
8 Author	
9 Solvent	CDCl3
10 Temperature	290.6
11 Pulse Sequence	zg30
12 Experiment	1D
13 Number of Scans	8
14 Receiver Gain	203
15 Relaxation Delay	1.0000
16 Pulse Width	13.5000
17 Acquisition Time	2.9999
18 Acquisition Date	2013-01-21T21:02:00
19 Modification Date	2013-01-21T21:02:00
20 Spectrometer Frequency	400.13
21 Spectral Width	8223.7
22 Lowest Frequency	-1650.5
23 Nucleus	1H
24 Acquired Size	24670
25 Spectral Size	32768

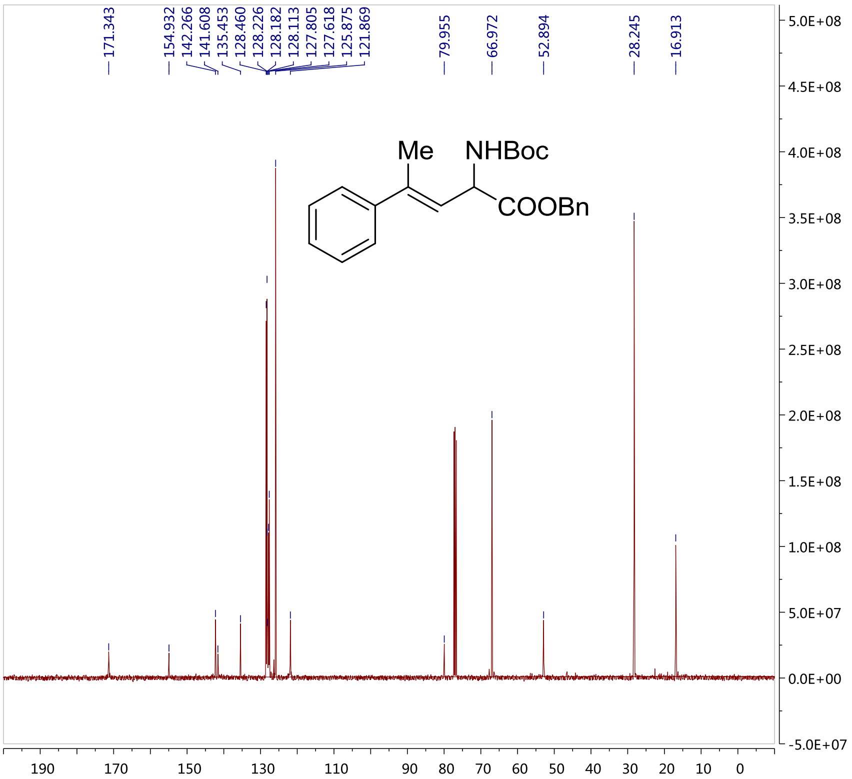

Parameter	Value
1 Data File Name	Z:/ Data/ NMR/ 2013-1/ GJX-6-22-C/ 1/ pdata/ 1/ 1r
2 Title	GJX-6-22-C
3 Comment	C13CPD
4 Origin	Bruker BioSpin GmbH
5 Owner	common
6 Site	
7 Spectrometer	spect
8 Author	
9 Solvent	CDCl3
10 Temperature	292.0
11 Pulse Sequence	zgpg30
12 Experiment	1D
13 Number of Scans	552
14 Receiver Gain	203
15 Relaxation Delay	1.0000
16 Pulse Width	12.5000
17 Acquisition Time	0.5506
18 Acquisition Date	2013-01-21T21:27:00
19 Modification Date	2013-01-21T21:27:00
20 Spectrometer Frequency	100.61
21 Spectral Width	29761.9
22 Lowest Frequency	-4820.2
23 Nucleus	13C
24 Acquired Size	16384
25 Spectral Size	32768

(*E*)-Benzyl 2-(*tert*-butoxycarbonylamino)-4-(4-chlorophenyl)pent-3-enoate (2h)

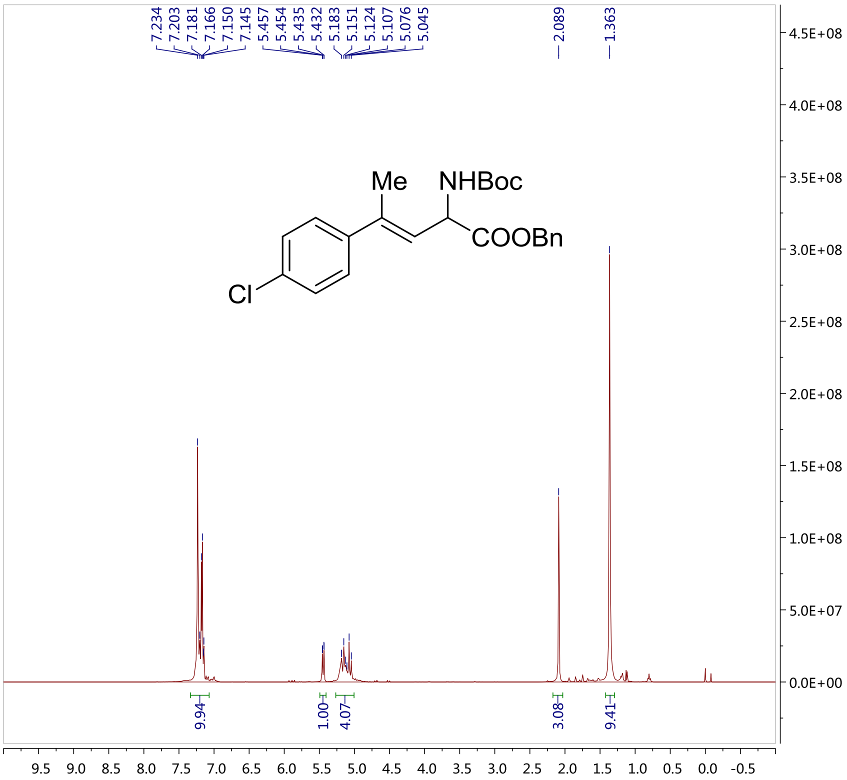

	Parameter	Value
1	Data File Name	Z:/ Data/ NMR/ 2013-4/ GJX-7-43/ 1/ pdata/ 1/ 1r
2	Title	GJX-7-43
3	Comment	PROTON
4	Origin	Bruker BioSpin GmbH
5	Owner	common
6	Site	
7	Spectrometer	spect
8	Author	
9	Solvent	CDCl3
10	Temperature	295.1
11	Pulse Sequence	zg30
12	Experiment	1D
13	Number of Scans	8
14	Receiver Gain	51
15	Relaxation Delay	1.0000
16	Pulse Width	13.7000
17	Acquisition Time	1.9999
18	Acquisition Date	2013-04-09T15:34:00
19	Modification Date	2013-04-09T15:35:00
20	Spectrometer Frequency	400.13
21	Spectral Width	8223.7
22	Lowest Frequency	-1686.1
23	Nucleus	1H
24	Acquired Size	16446
25	Spectral Size	32768

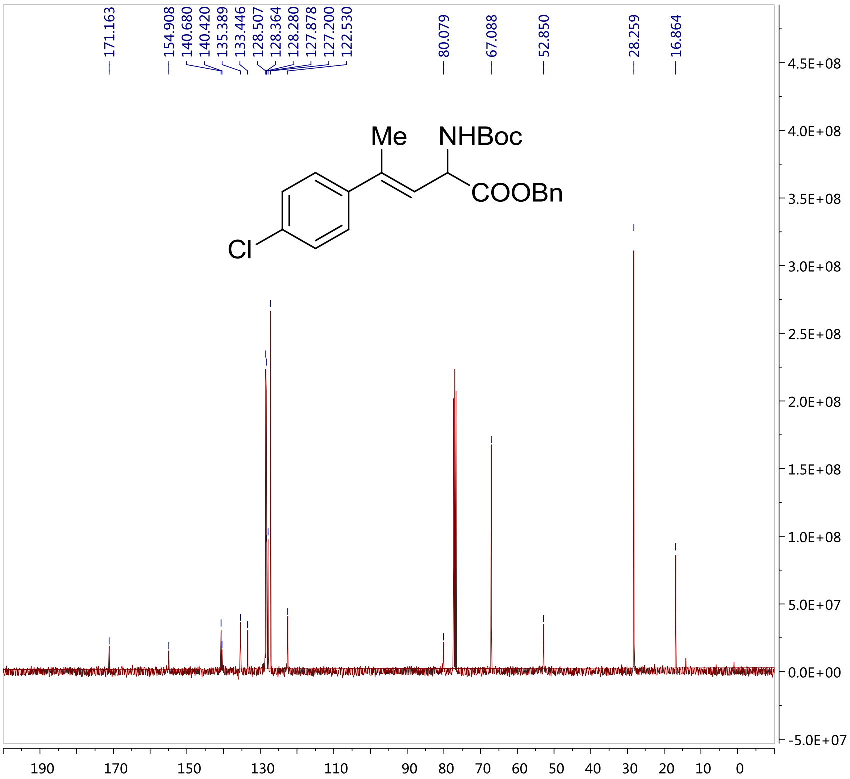

	Parameter	Value
1	Data File Name	Z:/ Data/ NMR/ 2013-4/ GJX-7-43/ 2/ pdata/ 1/ 1r
2	Title	GJX-7-43
3	Comment	C13CPD
4	Origin	Bruker BioSpin GmbH
5	Owner	common
6	Site	
7	Spectrometer	spect
8	Author	
9	Solvent	CDCl3
10	Temperature	296.0
11	Pulse Sequence	zgpg30
12	Experiment	1D
13	Number of Scans	237
14	Receiver Gain	203
15	Relaxation Delay	1.0000
16	Pulse Width	12.0000
17	Acquisition Time	0.4588
18	Acquisition Date	2013-04-09T15:40:00
19	Modification Date	2013-04-09T15:42:00
20	Spectrometer Frequency	100.61
21	Spectral Width	35714.3
22	Lowest Frequency	-7796.3
23	Nucleus	13C
24	Acquired Size	16384
25	Spectral Size	32768

(*E*)-Benzyl 2-(*tert*-butoxycarbonylamino)-4-(4-fluorophenyl)pent-3-enoate (2i)

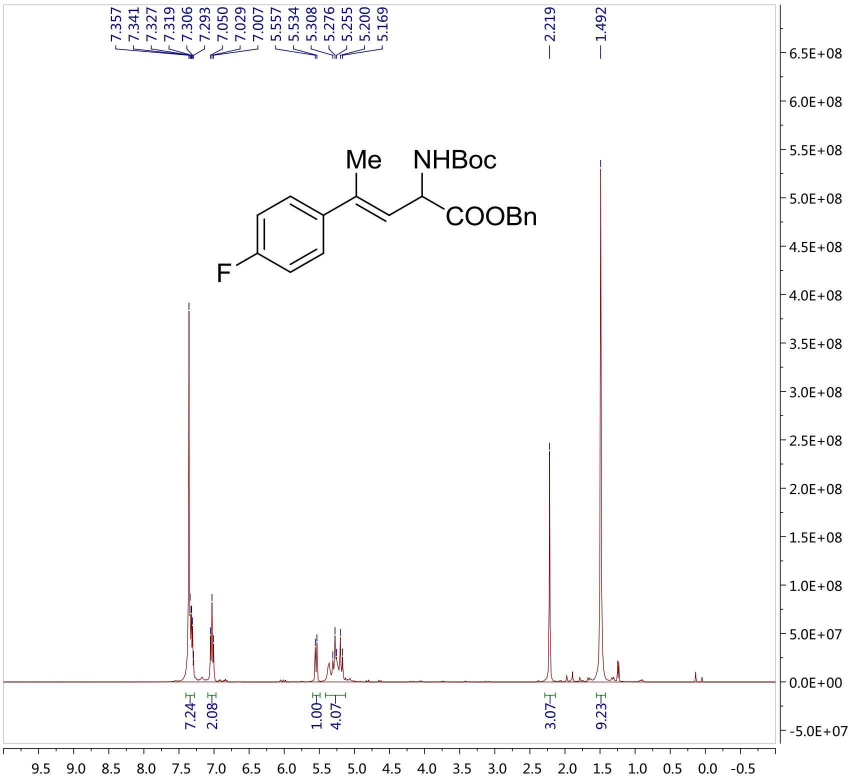

	Parameter	Value
1	Data File Name	Z:/ Data/ NMR/ 2013-4/ GJX-7-48/ 1/ pdata/ 1/ 1r
2	Title	GJX-7-48
3	Comment	PROTON
4	Origin	Bruker BioSpin GmbH
5	Owner	common
6	Site	
7	Spectrometer	spect
8	Author	
9	Solvent	CDCl3
10	Temperature	290.7
11	Pulse Sequence	zg30
12	Experiment	1D
13	Number of Scans	8
14	Receiver Gain	45
15	Relaxation Delay	1.0000
16	Pulse Width	13.5000
17	Acquisition Time	1.9923
18	Acquisition Date	2013-04-12T15:08:00
19	Modification Date	2013-04-12T15:08:00
20	Spectrometer Frequency	400.23
21	Spectral Width	8223.7
22	Lowest Frequency	-1640.3
23	Nucleus	1H
24	Acquired Size	16384
25	Spectral Size	32768

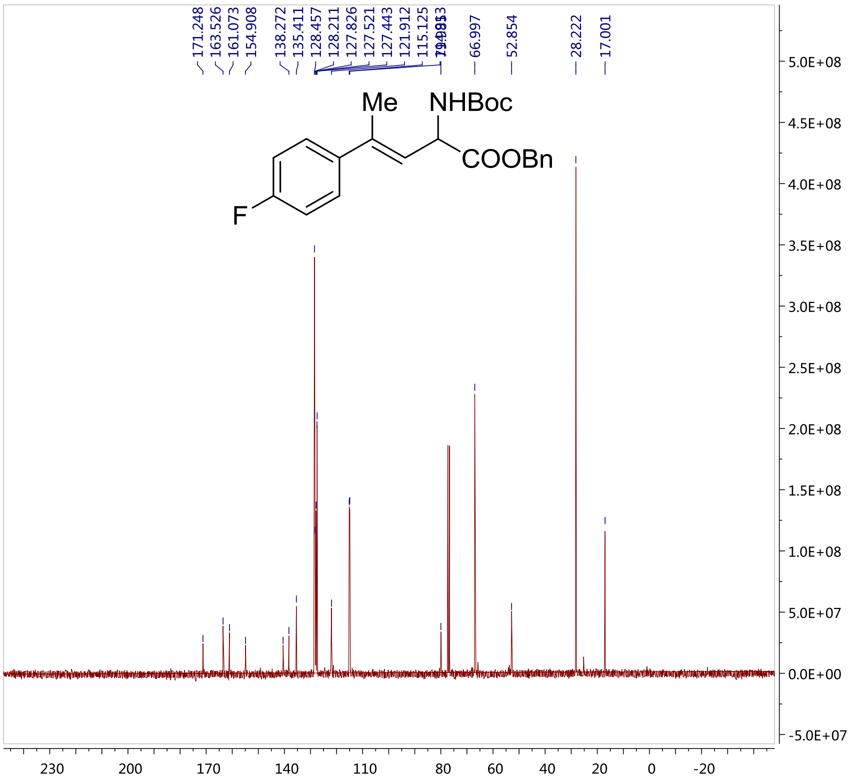

	Parameter	Value
1	Data File Name	Z:/ Data/ NMR/ 2013-4/ GJX-7-48/ 2/ pdata/ 1/ 1r
2	Title	GJX-7-48
3	Comment	C13CPD
4	Origin	Bruker BioSpin GmbH
5	Owner	common
6	Site	
7	Spectrometer	spect
8	Author	
9	Solvent	CDCl3
10	Temperature	291.7
11	Pulse Sequence	zgpg30
12	Experiment	1D
13	Number of Scans	320
14	Receiver Gain	203
15	Relaxation Delay	1.0000
16	Pulse Width	12.0000
17	Acquisition Time	0.5506
18	Acquisition Date	2013-04-12T15:15:00
19	Modification Date	2013-04-12T15:17:00
20	Spectrometer Frequency	100.64
21	Spectral Width	29761.9
22	Lowest Frequency	-4817.6
23	Nucleus	13C
24	Acquired Size	16384
25	Spectral Size	32768

(*E*)-Benzyl 2-(*tert*-butoxycarbonylamino)-4-(4-methoxyphenyl)pent-3-enoate (2j)

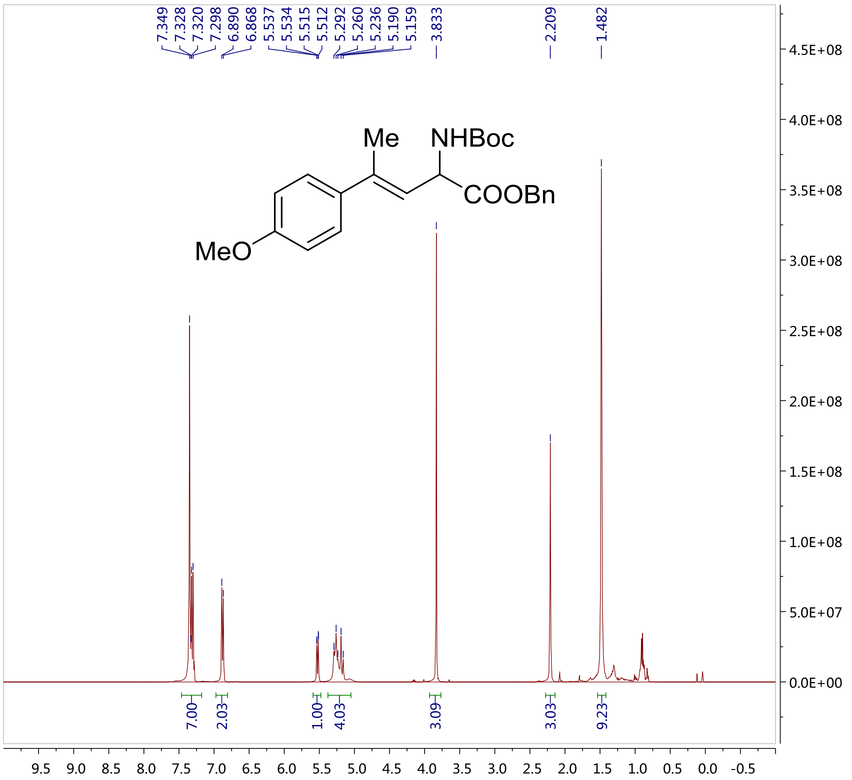

	Parameter	Value
1	Data File Name	Z:/ Data/ NMR/ 2013-4/ GJX-7-46/ 1/ pdata/ 1/ 1r
2	Title	GJX-7-46
3	Comment	PROTON
4	Origin	Bruker BioSpin GmbH
5	Owner	common
6	Site	
7	Spectrometer	spect
8	Author	
9	Solvent	CDCl3
10	Temperature	294.8
11	Pulse Sequence	zg30
12	Experiment	1D
13	Number of Scans	8
14	Receiver Gain	28
15	Relaxation Delay	1.0000
16	Pulse Width	13.7000
17	Acquisition Time	1.9999
18	Acquisition Date	2013-04-11T19:33:00
19	Modification Date	2013-04-11T19:33:00
20	Spectrometer Frequency	400.13
21	Spectral Width	8223.7
22	Lowest Frequency	-1640.9
23	Nucleus	1H
24	Acquired Size	16446
25	Spectral Size	32768

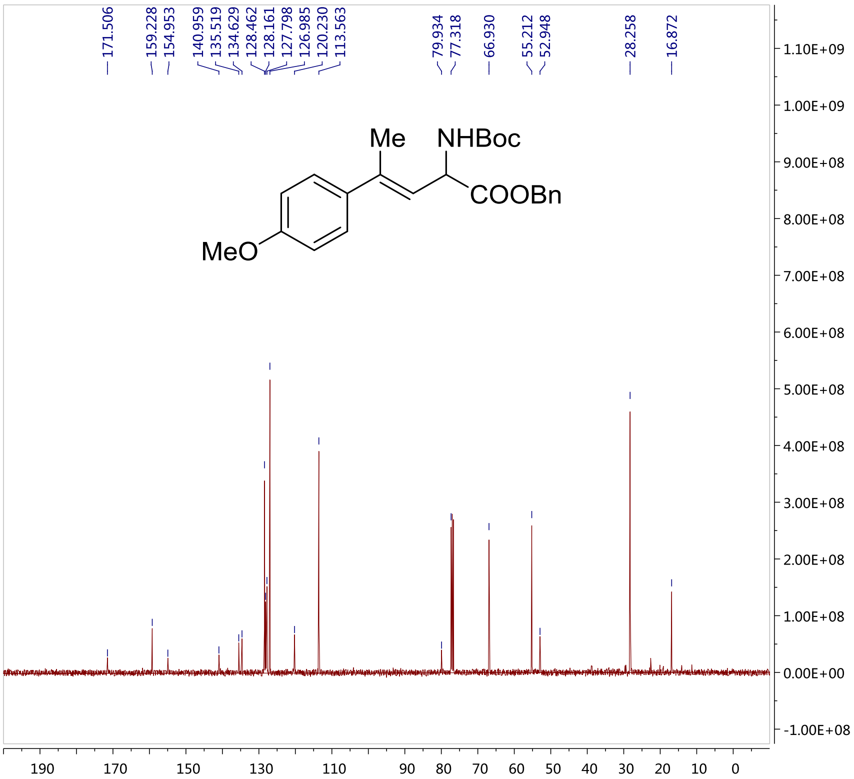

	Parameter	Value
1	Data File Name	Z:/ Data/ NMR/ 2013-4/ GJX-7-46/ 2/ pdata/ 1/ 1r
2	Title	GJX-7-46
3	Comment	C13CPD
4	Origin	Bruker BioSpin GmbH
5	Owner	common
6	Site	
7	Spectrometer	spect
8	Author	
9	Solvent	CDCl3
10	Temperature	295.9
11	Pulse Sequence	zgpg30
12	Experiment	1D
13	Number of Scans	179
14	Receiver Gain	203
15	Relaxation Delay	1.0000
16	Pulse Width	12.0000
17	Acquisition Time	0.4588
18	Acquisition Date	2013-04-11T19:38:00
19	Modification Date	2013-04-11T19:38:00
20	Spectrometer Frequency	100.61
21	Spectral Width	35714.3
22	Lowest Frequency	-7796.3
23	Nucleus	13C
24	Acquired Size	16384
25	Spectral Size	32768

(*E*)-Benzyl 2-(*tert*-butoxycarbonylamino)-4-(naphthalen-2-yl)pent-3-enoate (2k)

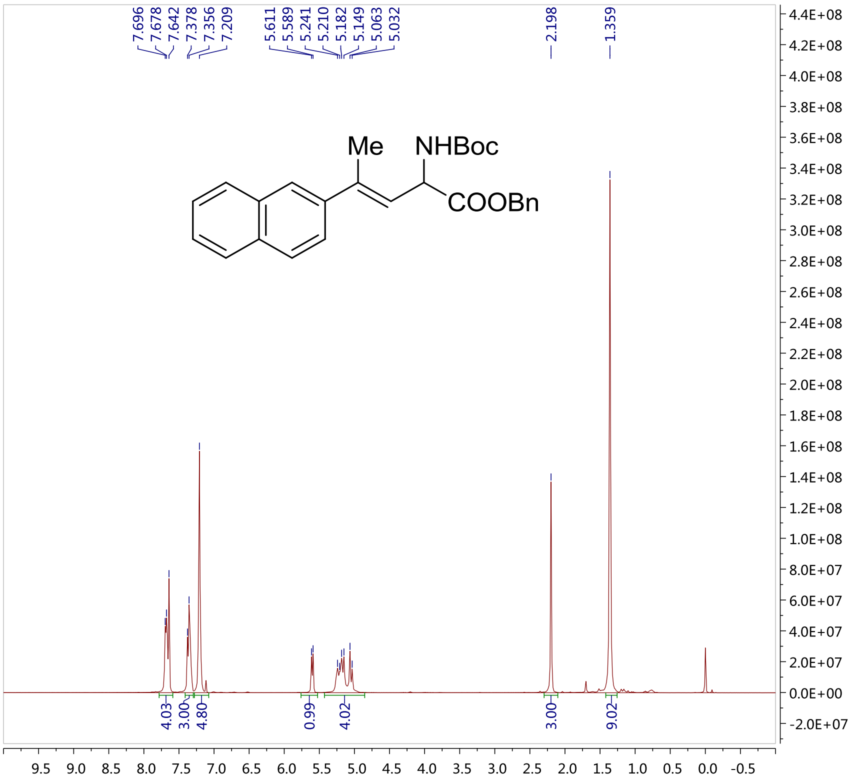

Parameter	Value
1 Data File Name	Z:/ Data/ NMR/ 2013-10/ GJX-9-43-naph/ 1/ pdata/ 1/ 1r
2 Title	GJX-9-43-naph
3 Comment	PROTON
4 Origin	Bruker BioSpin GmbH
5 Owner	nmr
6 Site	
7 Spectrometer	spect
8 Author	
9 Solvent	CDCl3
10 Temperature	295.8
11 Pulse Sequence	zg30
12 Experiment	1D
13 Number of Scans	8
14 Receiver Gain	23
15 Relaxation Delay	1.0000
16 Pulse Width	13.7000
17 Acquisition Time	1.9999
18 Acquisition Date	2013-10-04T13:51:00
19 Modification Date	2013-10-04T13:52:00
20 Spectrometer Frequency	400.13
21 Spectral Width	8223.7
22 Lowest Frequency	-1709.1
23 Nucleus	1H
24 Acquired Size	16446
25 Spectral Size	32768

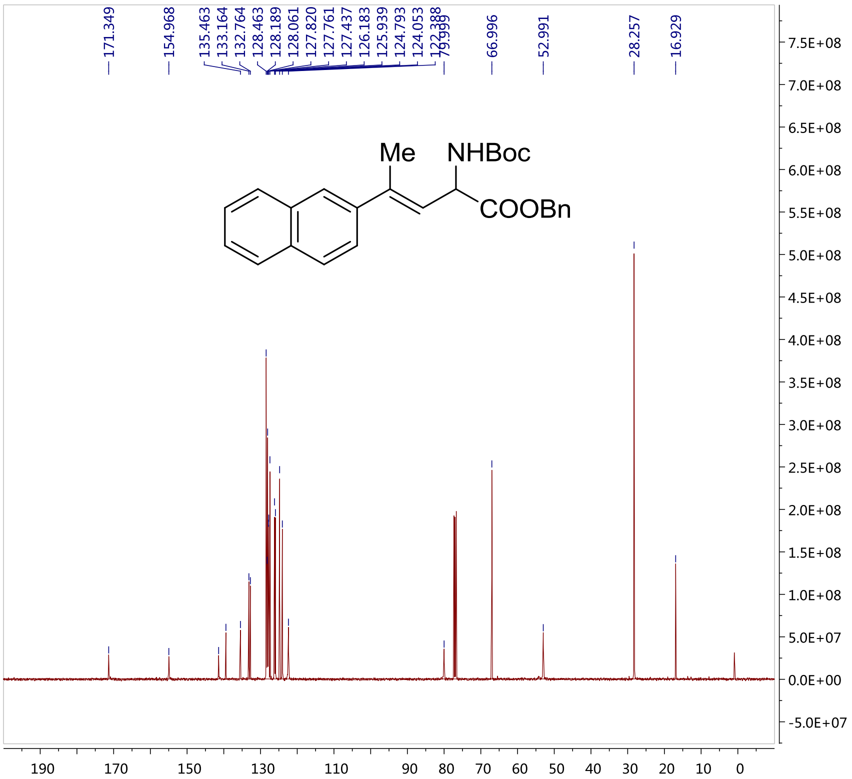

Parameter	Value
1 Data File Name	Z:/ Data/ NMR/ 2013-10/ GJX-9-43-naph/ 2/ pdata/ 1/ 1r
2 Title	GJX-9-43-naph
3 Comment	C13CPD
4 Origin	Bruker BioSpin GmbH
5 Owner	nmr
6 Site	
7 Spectrometer	spect
8 Author	
9 Solvent	CDCl3
10 Temperature	297.1
11 Pulse Sequence	zgpg30
12 Experiment	1D
13 Number of Scans	1024
14 Receiver Gain	203
15 Relaxation Delay	1.0000
16 Pulse Width	12.0000
17 Acquisition Time	0.4588
18 Acquisition Date	2013-10-04T14:19:00
19 Modification Date	2013-10-04T14:19:00
20 Spectrometer Frequency	100.61
21 Spectral Width	35714.3
22 Lowest Frequency	-7725.9
23 Nucleus	13C
24 Acquired Size	16384
25 Spectral Size	32768

(*E*)-Benzyl-4-(benzo[d][1,3]dioxol-5-yl)-2-(*tert*-butoxycarbonylamino)pent-3-enoate (2l)

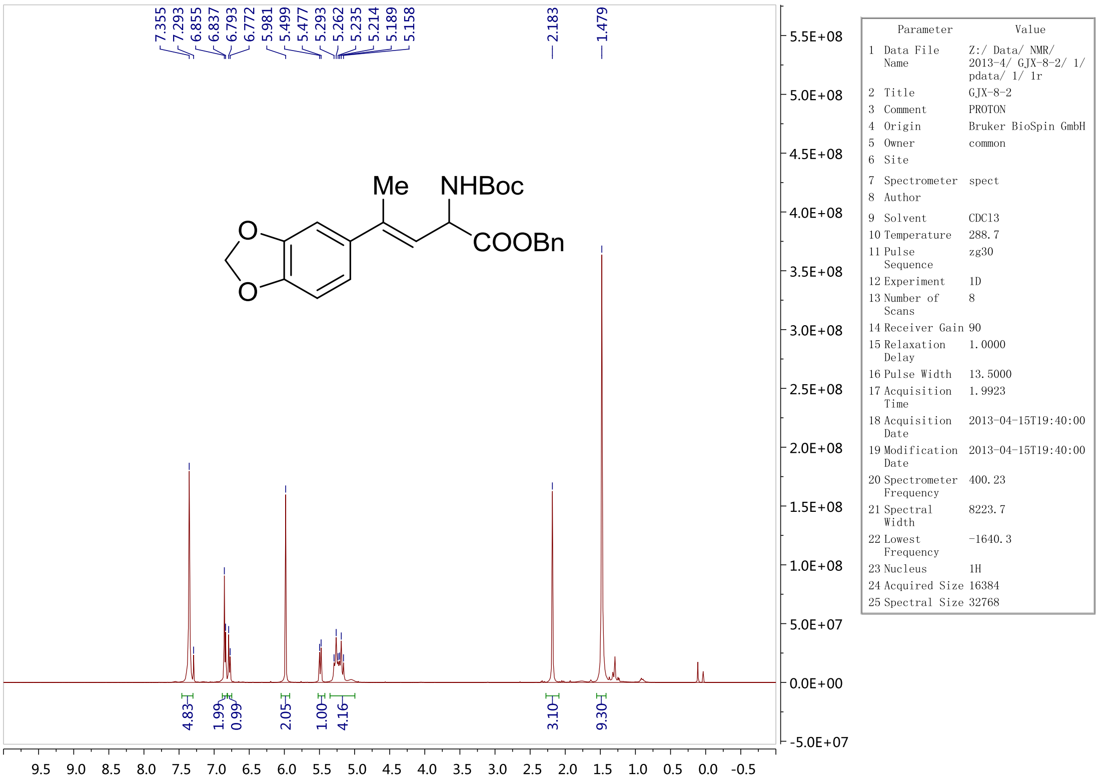

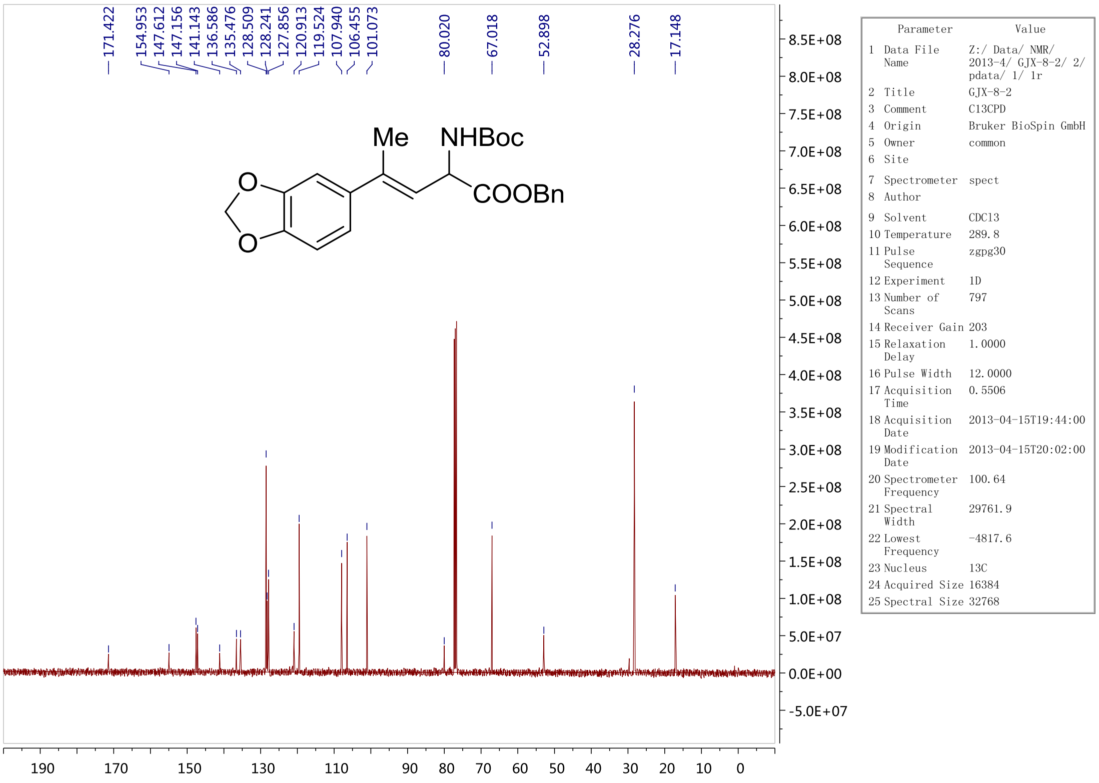

(*E*)-Benzyl 2-(*tert*-butoxycarbonylamino)-4-(thiophen-2-yl)pent-3-enoate (2m)

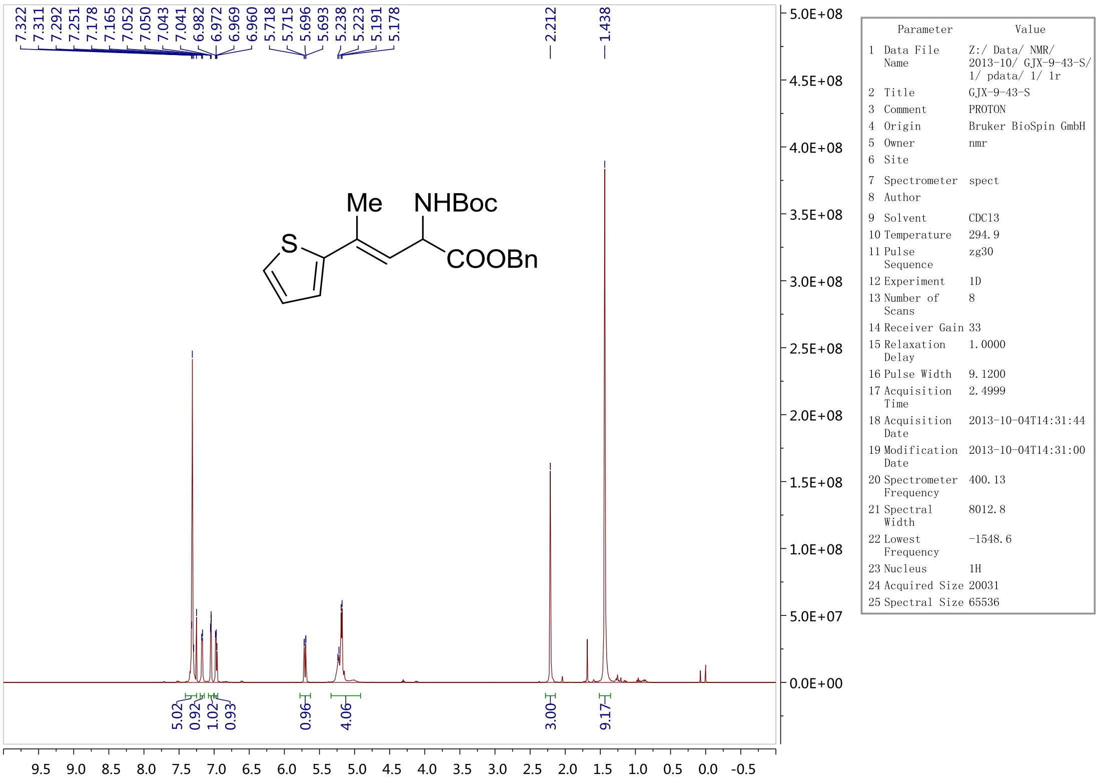

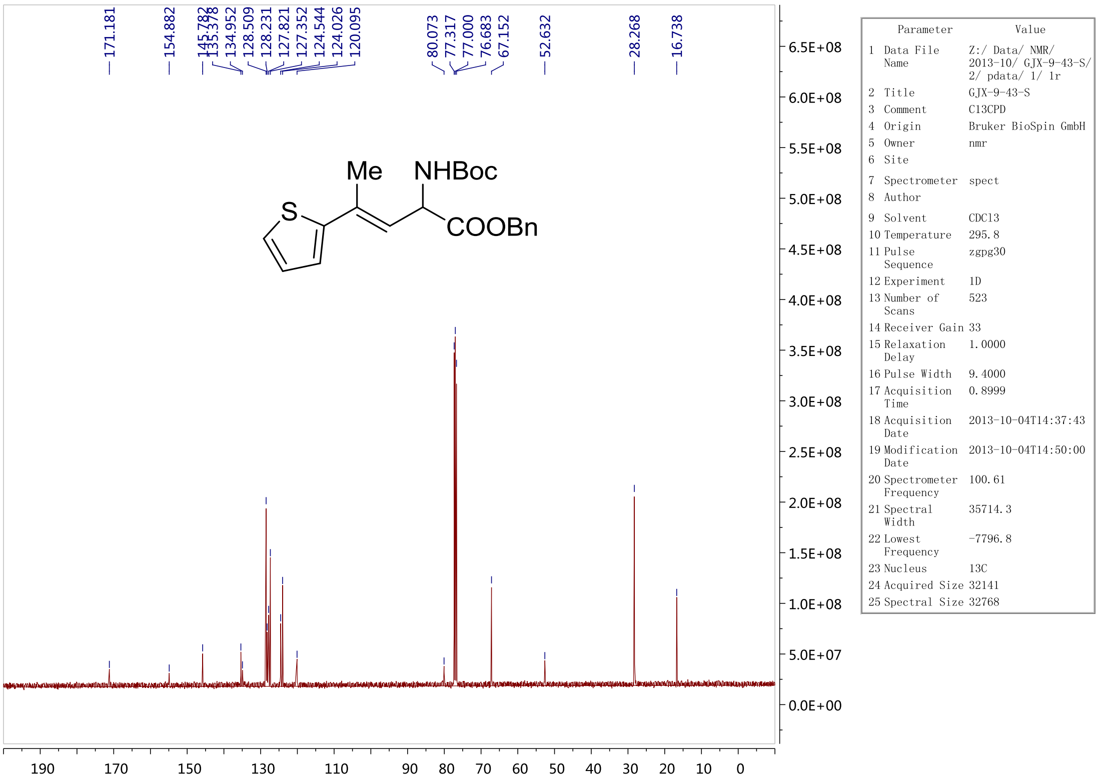

Benzyl 2-(*tert*-butoxycarbonylamino)-4-methylpent-3-enoate (2n)

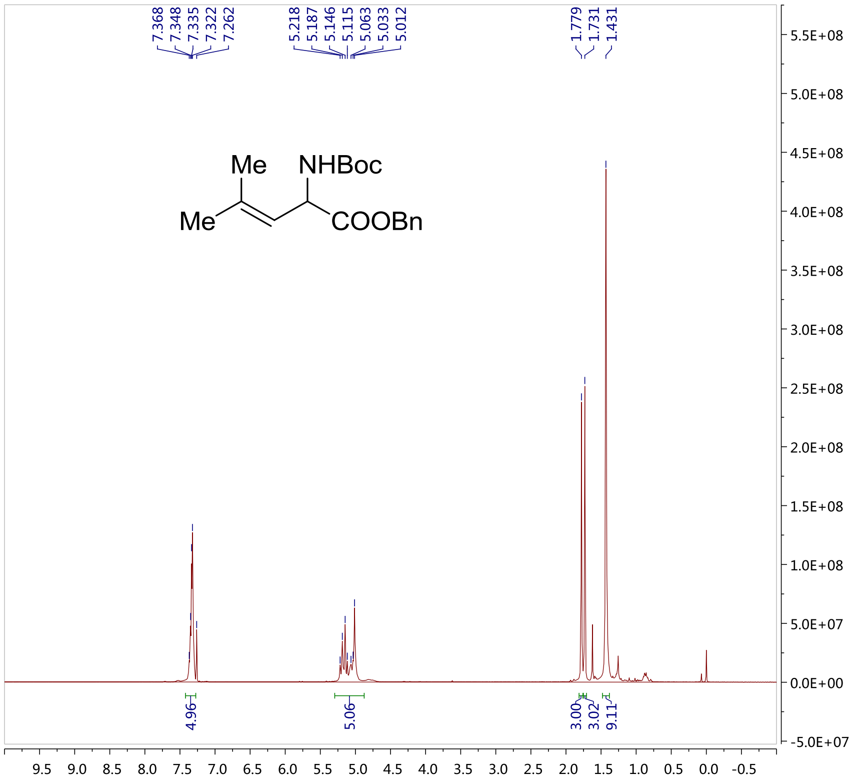

	Parameter	Value
1	Data File Name	Z:/ Data/ NMR/ 2013-4/ GJX-7-24-CH/ 1/ pdata/ 1/ 1r
2	Title	GJX-7-24-CH
3	Comment	PROTON
4	Origin	Bruker BioSpin GmbH
5	Owner	common
6	Site	
7	Spectrometer	spect
8	Author	
9	Solvent	CDCl3
10	Temperature	291.0
11	Pulse Sequence	zg30
12	Experiment	1D
13	Number of Scans	8
14	Receiver Gain	114
15	Relaxation Delay	1.0000
16	Pulse Width	13.5000
17	Acquisition Time	1.9923
18	Acquisition Date	2013-04-01T10:47:00
19	Modification Date	2013-04-01T10:47:00
20	Spectrometer Frequency	400.23
21	Spectral Width	8223.7
22	Lowest Frequency	-1640.3
23	Nucleus	1H
24	Acquired Size	16384
25	Spectral Size	32768

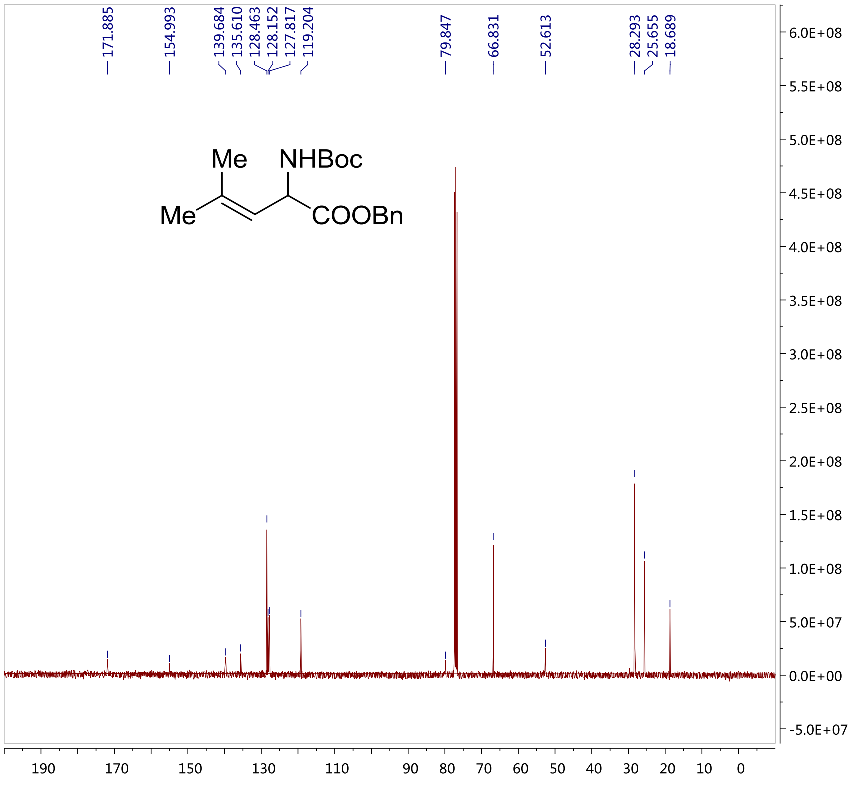

	Parameter	Value
1	Data File Name	Z:/ Data/ NMR/ 2013-4/ GJX-7-24-DEPT/ 1/ pdata/ 1/ 1r
2	Title	GJX-7-24-DEPT
3	Comment	C13CPD
4	Origin	Bruker BioSpin GmbH
5	Owner	common
6	Site	
7	Spectrometer	spect
8	Author	
9	Solvent	CDCl3
10	Temperature	295.4
11	Pulse Sequence	zgpg30
12	Experiment	1D
13	Number of Scans	1024
14	Receiver Gain	203
15	Relaxation Delay	1.0000
16	Pulse Width	12.0000
17	Acquisition Time	0.4588
18	Acquisition Date	2013-04-01T14:14:00
19	Modification Date	2013-04-01T14:31:00
20	Spectrometer Frequency	100.61
21	Spectral Width	35714.3
22	Lowest Frequency	-7796.3
23	Nucleus	13C
24	Acquired Size	16384
25	Spectral Size	32768

Benzyl 2-(*tert*-butoxycarbonylamino)but-3-enoate (2o)

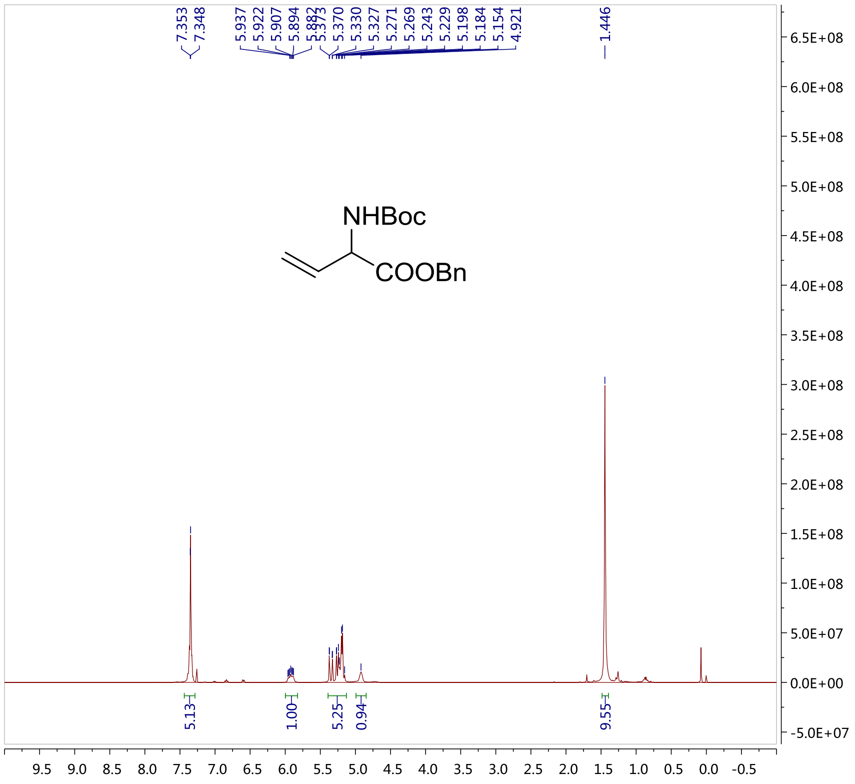

	Parameter	Value
1	Data File Name	Z:/ Data/ NMR/ 2013-3/ GJX-7-3-C/ 1/ pdata/ 1/ 1r
2	Title	GJX-7-3-C
3	Comment	PROTON
4	Origin	Bruker BioSpin GmbH
5	Owner	common
6	Site	
7	Spectrometer	spect
8	Author	
9	Solvent	CDCl3
10	Temperature	294.8
11	Pulse Sequence	zg30
12	Experiment	1D
13	Number of Scans	8
14	Receiver Gain	64
15	Relaxation Delay	1.0000
16	Pulse Width	13.7000
17	Acquisition Time	1.9999
18	Acquisition Date	2013-03-22T19:04:00
19	Modification Date	2013-03-22T19:04:00
20	Spectrometer Frequency	400.13
21	Spectral Width	8223.7
22	Lowest Frequency	-1640.9
23	Nucleus	1H
24	Acquired Size	16446
25	Spectral Size	32768

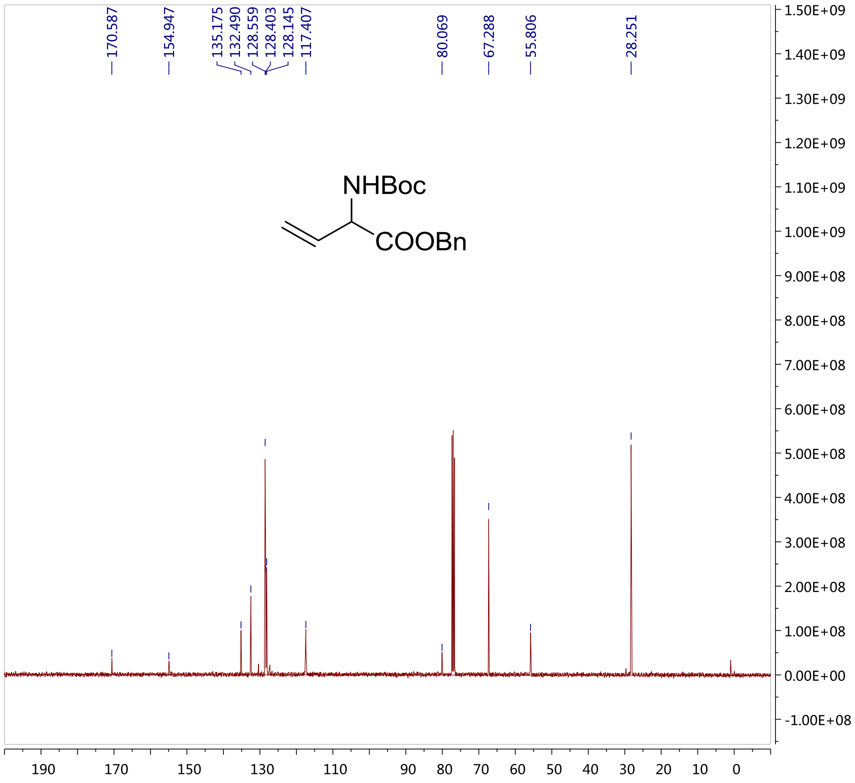

	Parameter	Value
1	Data File Name	Z:/ Data/ NMR/ 2013-3/ GJX-7-3-C/ 2/ pdata/ 1/ 1r
2	Title	GJX-7-3-C
3	Comment	C13CPD
4	Origin	Bruker BioSpin GmbH
5	Owner	common
6	Site	
7	Spectrometer	spect
8	Author	
9	Solvent	CDCl3
10	Temperature	296.0
11	Pulse Sequence	zgpg30
12	Experiment	1D
13	Number of Scans	583
14	Receiver Gain	203
15	Relaxation Delay	1.0000
16	Pulse Width	12.0000
17	Acquisition Time	0.4588
18	Acquisition Date	2013-03-22T19:19:00
19	Modification Date	2013-03-22T19:19:00
20	Spectrometer Frequency	100.61
21	Spectral Width	35714.3
22	Lowest Frequency	-7901.2
23	Nucleus	13C
24	Acquired Size	16384
25	Spectral Size	32768

Ethyl 2-(*tert*-butoxycarbonylamino)-2-cyclohexenylacetate (2p)

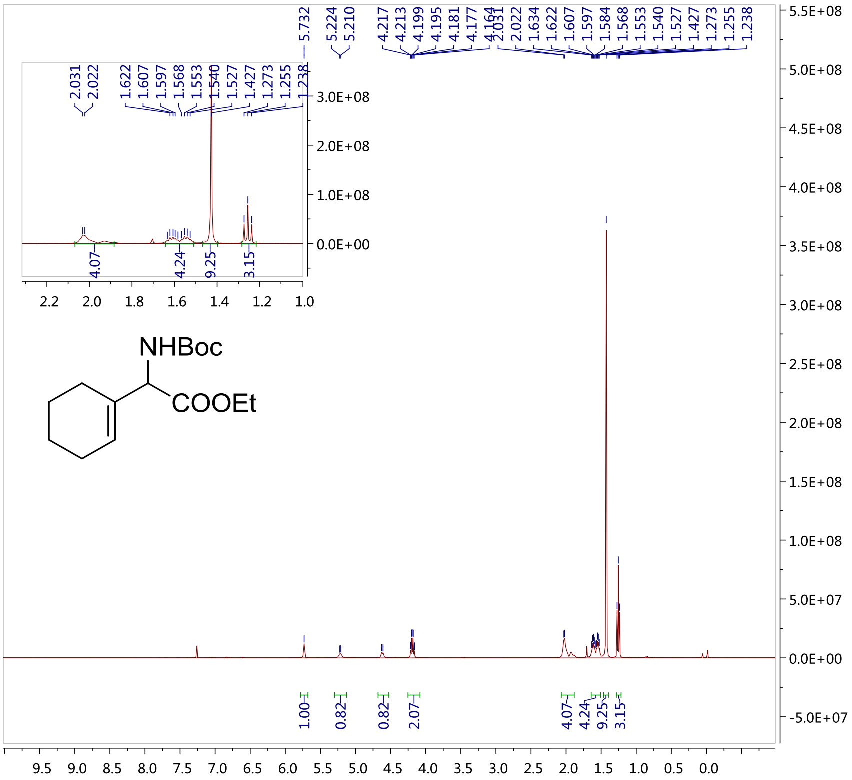

Parameter	Value
1 Data File Name	Z:/ Data/ NMR/ 2013-4/ GJX-7-34/ 1/ pdata/ 1/ 1r
2 Title	GJX-7-34
3 Comment	PROTON
4 Origin	Bruker BioSpin GmbH
5 Owner	common
6 Site	
7 Spectrometer	spect
8 Author	
9 Solvent	CDCl3
10 Temperature	295.0
11 Pulse Sequence	zg30
12 Experiment	1D
13 Number of Scans	8
14 Receiver Gain	72
15 Relaxation Delay	1.0000
16 Pulse Width	13.7000
17 Acquisition Time	1.9999
18 Acquisition Date	2013-04-05T15:18:00
19 Modification Date	2013-04-05T15:18:00
20 Spectrometer Frequency	400.13
21 Spectral Width	8223.7
22 Lowest Frequency	-1644.6
23 Nucleus	1H
24 Acquired Size	16446
25 Spectral Size	32768

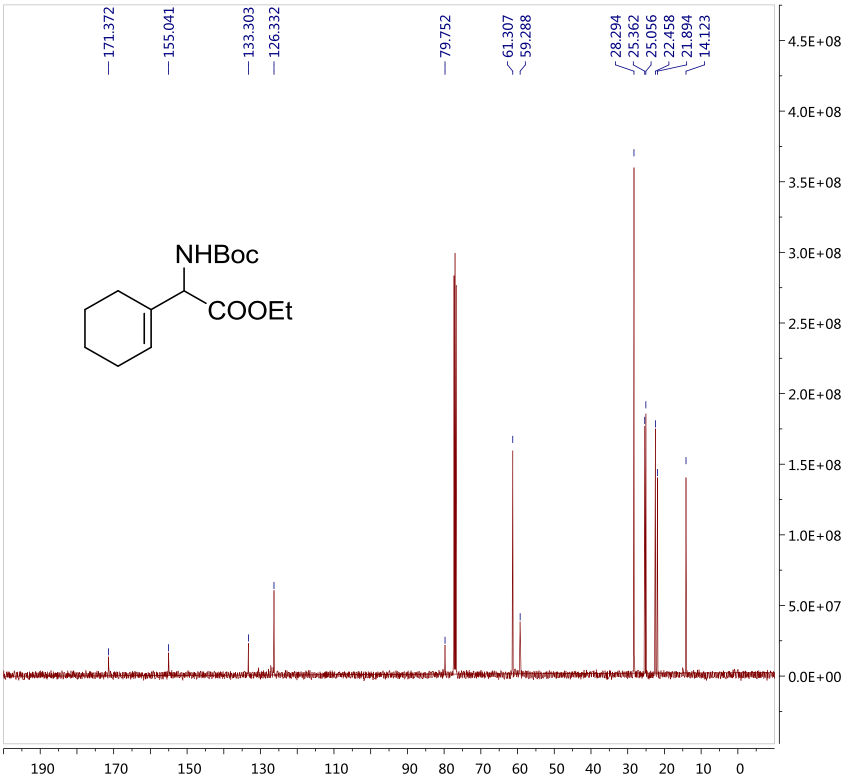

Parameter	Value
1 Data File Name	Z:/ Data/ NMR/ 2013-4/ GJX-7-34/ 2/ pdata/ 1/ 1r
2 Title	GJX-7-34
3 Comment	C13CPD
4 Origin	Bruker BioSpin GmbH
5 Owner	common
6 Site	
7 Spectrometer	spect
8 Author	
9 Solvent	CDCl3
10 Temperature	296.2
11 Pulse Sequence	zgpg30
12 Experiment	1D
13 Number of Scans	686
14 Receiver Gain	203
15 Relaxation Delay	1.0000
16 Pulse Width	12.0000
17 Acquisition Time	0.4588
18 Acquisition Date	2013-04-05T15:31:00
19 Modification Date	2013-04-05T15:36:00
20 Spectrometer Frequency	100.61
21 Spectral Width	35714.3
22 Lowest Frequency	-7796.3
23 Nucleus	13C
24 Acquired Size	16384
25 Spectral Size	32768

(*E*)-Benzyl-5-(benzyloxy)-2-(*tert*-butoxycarbonylamino)pent-3-enoate (2q)

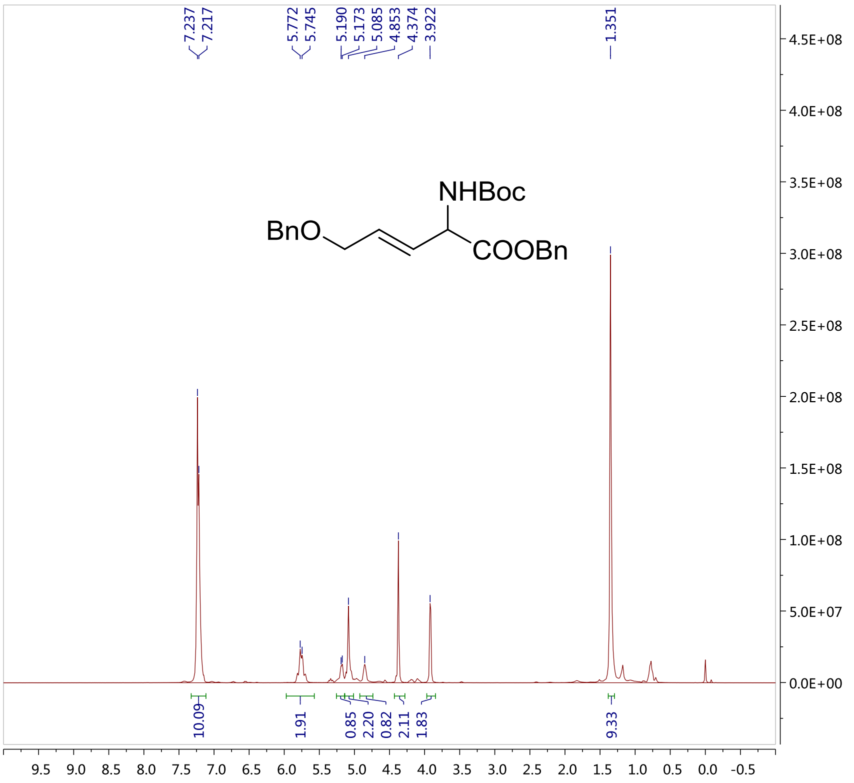

Parameter	Value
1 Data File Name	Z:/ Data/ NMR/ 2013-6/ GJX-8-50-C/ 1/ pdata/ 1/ 1r
2 Title	GJX-8-50-C
3 Comment	PROTON
4 Origin	Bruker BioSpin GmbH
5 Owner	nmr
6 Site	
7 Spectrometer	spect
8 Author	
9 Solvent	CDCl3
10 Temperature	295.5
11 Pulse Sequence	zg30
12 Experiment	1D
13 Number of Scans	8
14 Receiver Gain	23
15 Relaxation Delay	1.0000
16 Pulse Width	13.7000
17 Acquisition Time	1.9999
18 Acquisition Date	2013-06-20T14:02:00
19 Modification Date	2013-06-20T14:02:00
20 Spectrometer Frequency	400.13
21 Spectral Width	8223.7
22 Lowest Frequency	-1696.0
23 Nucleus	1H
24 Acquired Size	16446
25 Spectral Size	32768

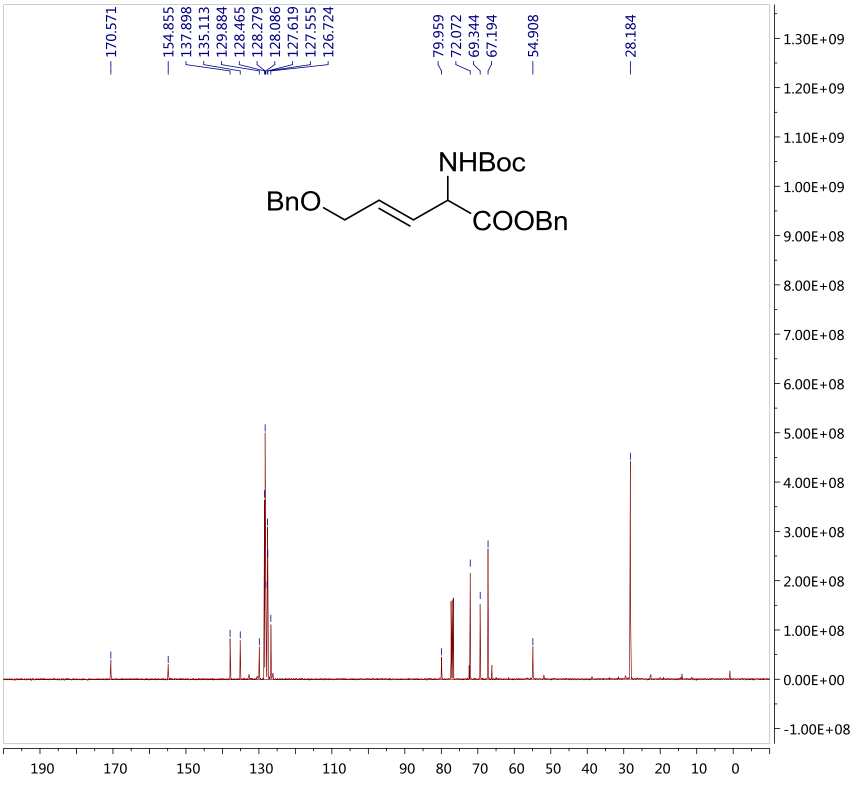

Parameter	Value
1 Data File Name	Z:/ Data/ NMR/ 2013-6/ GJX-8-50-C/ 2/ pdata/ 1/ 1r
2 Title	GJX-8-50-C
3 Comment	C13CPD
4 Origin	Bruker BioSpin GmbH
5 Owner	nmr
6 Site	
7 Spectrometer	spect
8 Author	
9 Solvent	CDCl3
10 Temperature	295.5
11 Pulse Sequence	zgpg30
12 Experiment	1D
13 Number of Scans	920
14 Receiver Gain	203
15 Relaxation Delay	1.0000
16 Pulse Width	12.0000
17 Acquisition Time	0.4588
18 Acquisition Date	2013-06-20T14:03:00
19 Modification Date	2013-06-20T14:26:00
20 Spectrometer Frequency	100.61
21 Spectral Width	35714.3
22 Lowest Frequency	-7905.3
23 Nucleus	13C
24 Acquired Size	16384
25 Spectral Size	32768

(*E*)-Methyl 2-(*tert*-butoxycarbonylamino)-4-phenylpent-3-enoate (2r)

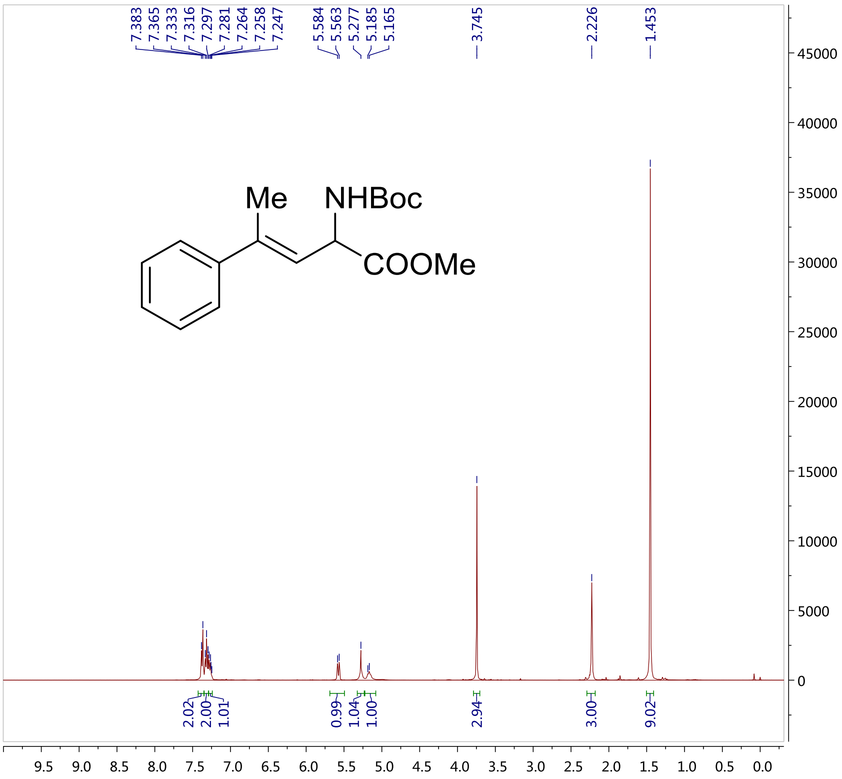

Parameter	Value
1 Data File Name	Z:/ Data/ NMR/ 2015-04/ zt-3-125-2/ 1/ fid
2 Title	zt-3-125-2
3 Comment	PROTON
4 Origin	Bruker BioSpin GmbH
5 Owner	common
6 Site	
7 Spectrometer	spect
8 Author	
9 Solvent	CDCl3
10 Temperature	1130.3
11 Pulse Sequence	zg30
12 Experiment	1D
13 Number of Scans	8
14 Receiver Gain	51
15 Relaxation Delay	1.0000
16 Pulse Width	13.5000
17 Acquisition Time	1.9923
18 Acquisition Date	2015-04-14T21:40:00
19 Modification Date	2015-04-14T21:41:00
20 Spectrometer Frequency	400.23
21 Spectral Width	8223.7
22 Lowest Frequency	-1640.3
23 Nucleus	1H
24 Acquired Size	16384
25 Spectral Size	32768

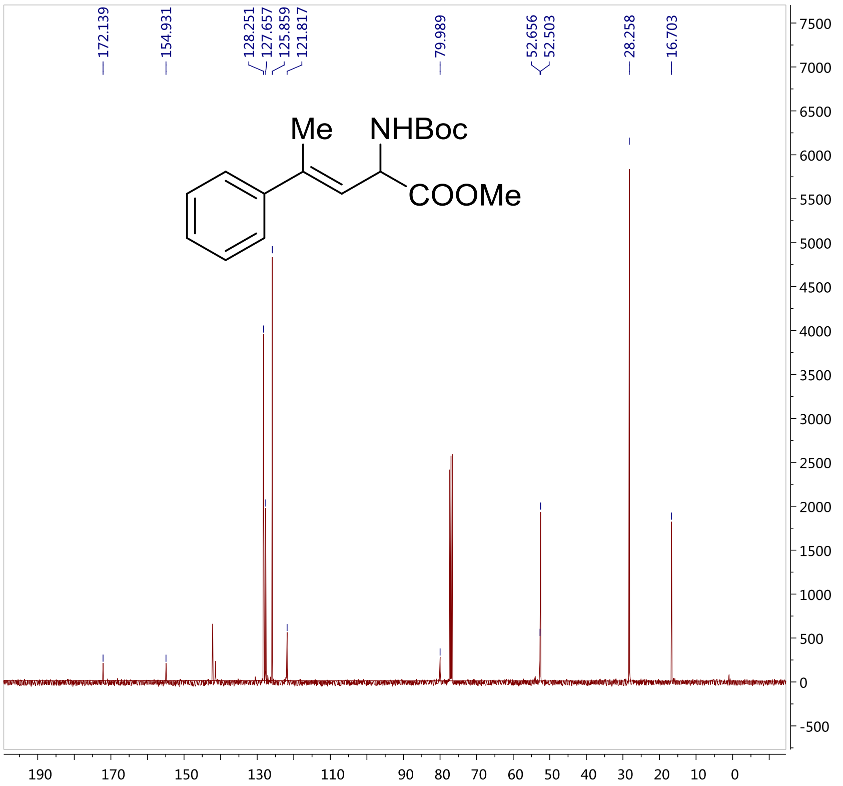

Parameter	Value
1 Data File Name	Z:/ Data/ NMR/ 2015-04/ ZT-3-125-opt-C13/ 1/ fid
2 Title	ZT-3-125-opt-C13
3 Comment	C13CPD
4 Origin	Bruker BioSpin GmbH
5 Owner	common
6 Site	
7 Spectrometer	spect
8 Author	
9 Solvent	CDCl3
10 Temperature	1144.1
11 Pulse Sequence	zgpg30
12 Experiment	1D
13 Number of Scans	685
14 Receiver Gain	203
15 Relaxation Delay	1.0000
16 Pulse Width	12.0000
17 Acquisition Time	0.5506
18 Acquisition Date	2015-04-15T09:58:00
19 Modification Date	2015-04-15T09:59:00
20 Spectrometer Frequency	100.64
21 Spectral Width	29761.9
22 Lowest Frequency	-4923.7
23 Nucleus	13C
24 Acquired Size	16384
25 Spectral Size	32768

(*E*)-*tert*-Butyl 2-(*tert*-butoxycarbonylamino)-4-phenylpent-3-enoate (2s)

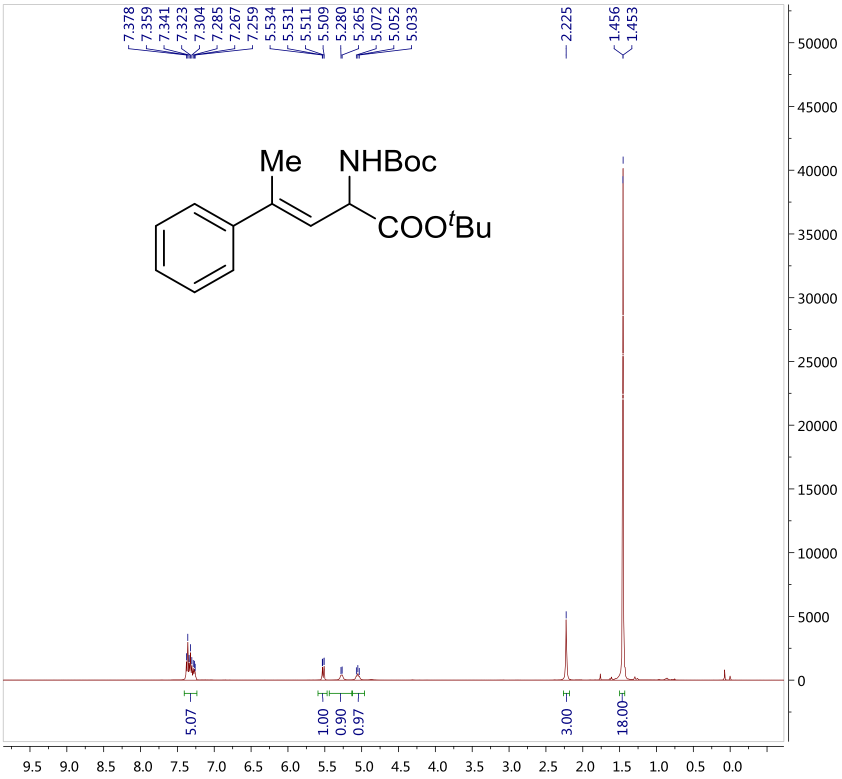

Parameter		Value
1	Data File Name	Z:/ Data/ NMR/ 2015-04/ zt-3-121-2/ 1/ fid
2	Title	zt-2-121-2
3	Comment	PROTON
4	Origin	Bruker BioSpin GmbH
5	Owner	common
6	Site	
7	Spectrometer	spect
8	Author	
9	Solvent	CDCl3
10	Temperature	1148.7
11	Pulse Sequence	zg30
12	Experiment	1D
13	Number of Scans	8
14	Receiver Gain	64
15	Relaxation Delay	1.0000
16	Pulse Width	13.5000
17	Acquisition Time	1.9923
18	Acquisition Date	2015-04-13T19:35:00
19	Modification Date	2015-04-13T19:35:00
20	Spectrometer Frequency	400.23
21	Spectral Width	8223.7
22	Lowest Frequency	-1640.3
23	Nucleus	1H
24	Acquired Size	16384
25	Spectral Size	32768

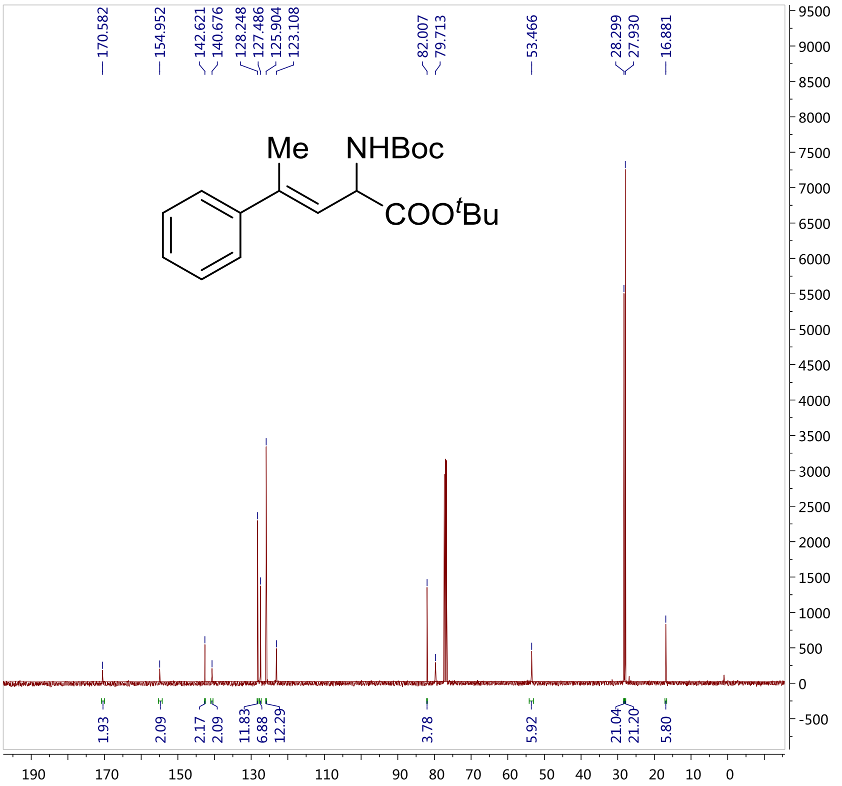

Parameter		Value
1	Data File Name	Z:/ Data/ NMR/ 2015-04/ zt-3-121-2/ 2/ fid
2	Title	zt-2-121-2
3	Comment	C13CPD
4	Origin	Bruker BioSpin GmbH
5	Owner	common
6	Site	
7	Spectrometer	spect
8	Author	
9	Solvent	CDCl3
10	Temperature	1146.5
11	Pulse Sequence	zgpg30
12	Experiment	1D
13	Number of Scans	673
14	Receiver Gain	203
15	Relaxation Delay	1.0000
16	Pulse Width	12.0000
17	Acquisition Time	0.5506
18	Acquisition Date	2015-04-13T19:39:00
19	Modification Date	2015-04-13T19:55:00
20	Spectrometer Frequency	100.64
21	Spectral Width	29761.9
22	Lowest Frequency	-4923.7
23	Nucleus	13C
24	Acquired Size	16384
25	Spectral Size	32768

8. HPLC Charts of N−H Insertion Products

(*E*)-Benzyl 2-(*tert*-butoxycarbonylamino)pent-3-enoate (2a)

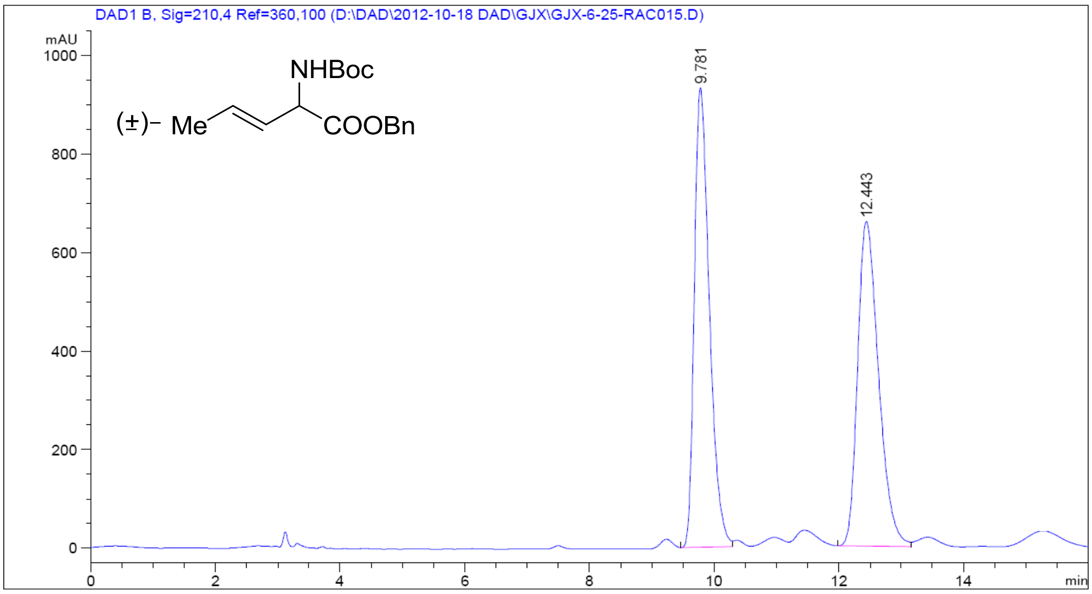

Peak #	RetTime [min]	Type	Width [min]	Area [mAU*s]	Height [mAU]	Area %
1	9.781	BV	0.2577	1.56464e4	932.71869	50.3876
2	12.443	BV	0.3586	1.54057e4	659.22626	49.6124

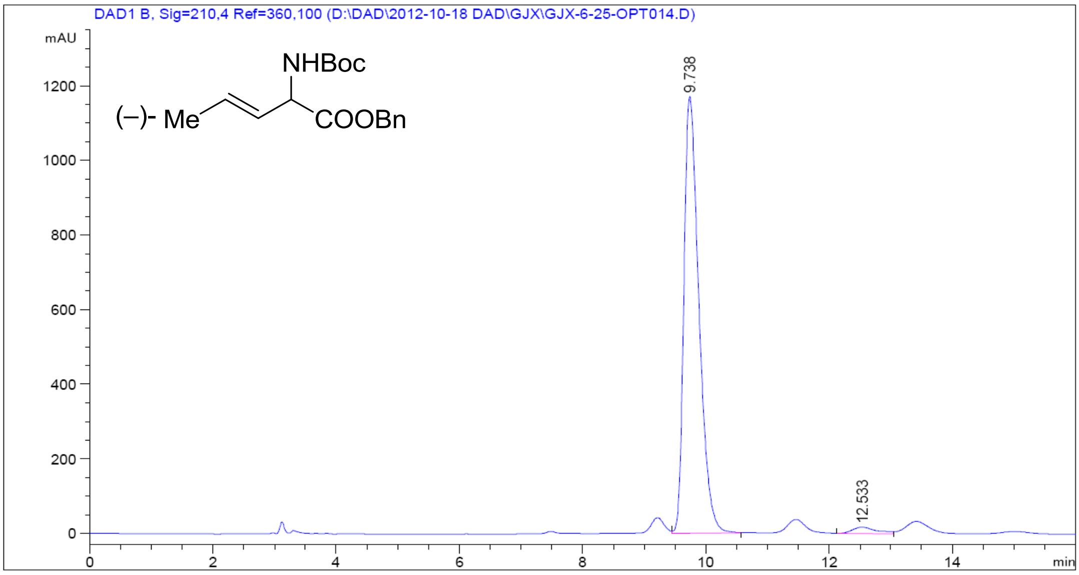

Peak #	RetTime [min]	Type	Width [min]	Area [mAU*s]	Height [mAU]	Area %
1	9.738	VV	0.2594	1.98060e4	1170.64514	97.7395
2	12.533	BV	0.3971	458.07730	17.06765	2.2605

(*E*)-Benzyl 2-(*tert*-butoxycarbonylamino)hex-3-enoate (2b)

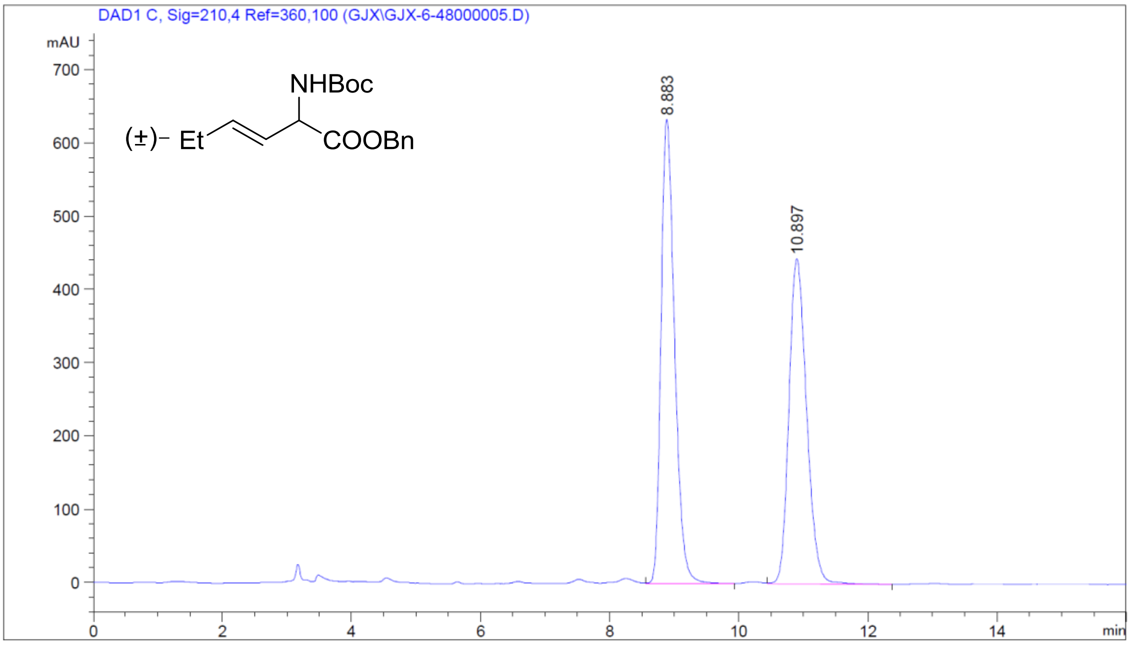

Peak #	RetTime [min]	Type	Width [min]	Area [mAU*s]	Height [mAU]	Area %
1	8.883	VB	0.2260	9226.93848	633.27240	52.2464
2	10.897	VB	0.2978	8433.47949	443.69116	47.7536

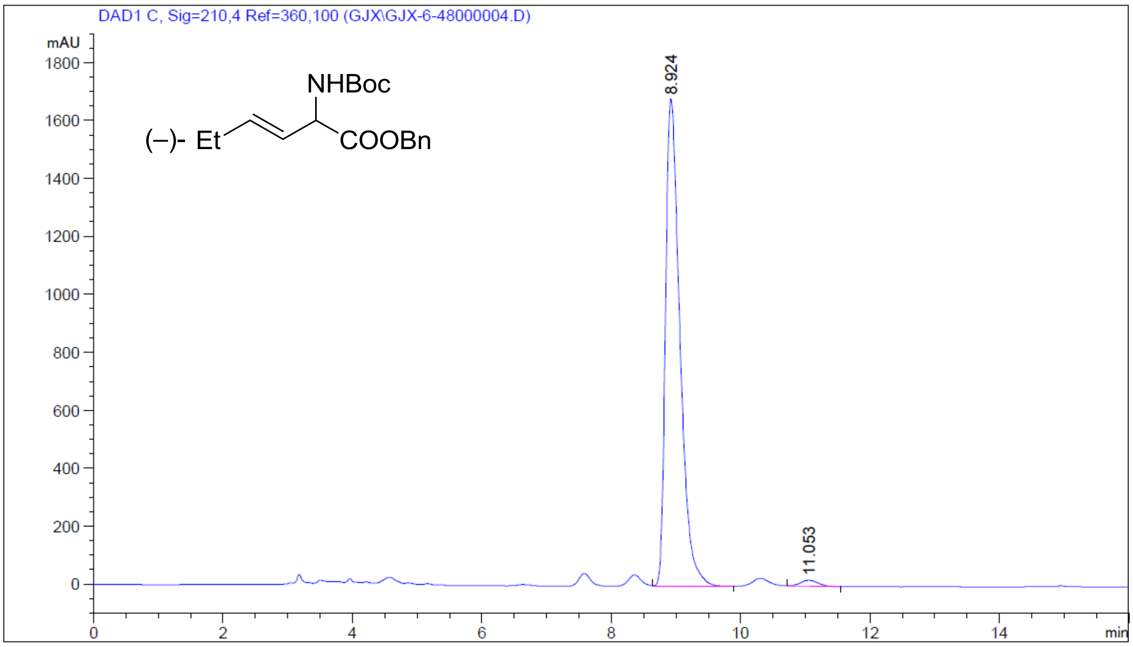

Peak #	RetTime [min]	Type	Width [min]	Area [mAU*s]	Height [mAU]	Area %
1	8.924	VB	0.2406	2.59757e4	1677.67651	98.4670
2	11.053	VB	0.2967	404.39600	21.37522	1.5330

(*E*)-Benzyl 2-(*tert*-butoxycarbonylamino)oct-3-enoate (2c)

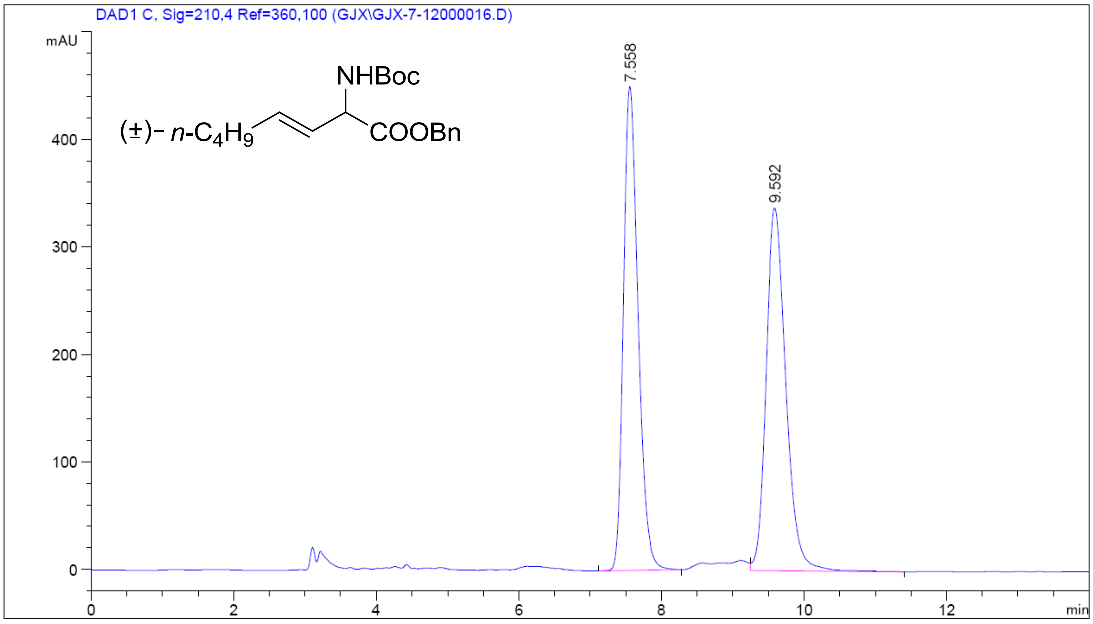

Peak #	RetTime [min]	Type	Width [min]	Area [mAU*s]	Height [mAU]	Area %
1	7.558	BB	0.2168	6356.18359	449.92758	49.4425
2	9.592	VB	0.2948	6499.52539	337.25769	50.5575

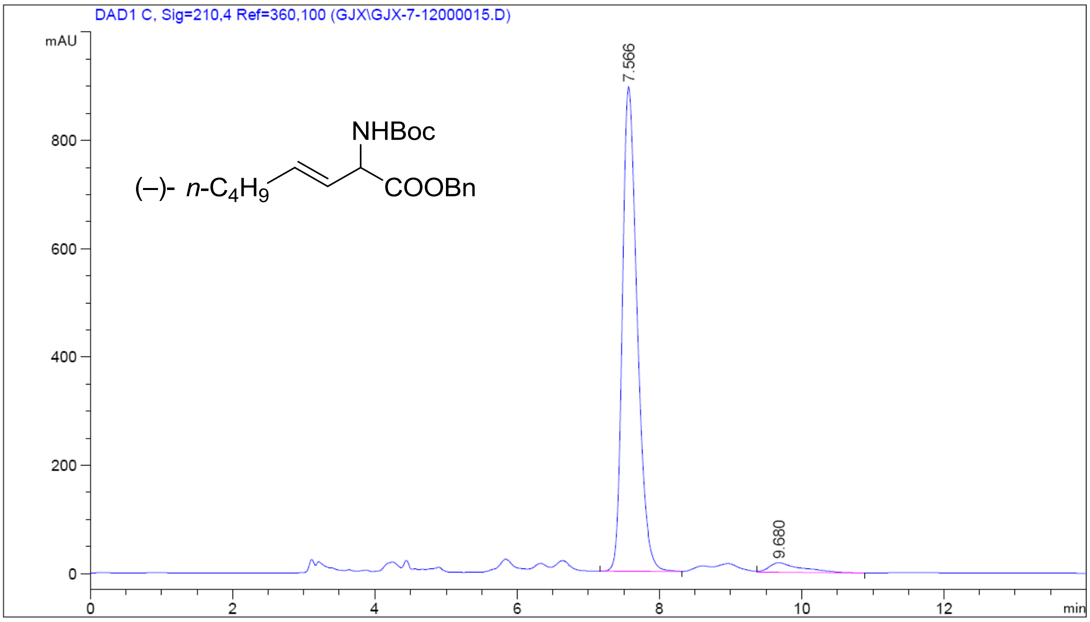

Peak #	RetTime [min]	Type	Width [min]	Area [mAU*s]	Height [mAU]	Area %
1	7.566	BB	0.2193	1.28330e4	894.71368	95.8479
2	9.680	VB	0.4392	555.92456	17.56835	4.1521

(*E*)-Benzyl 2-(*tert*-butoxycarbonylamino)dec-3-enoate (2d)

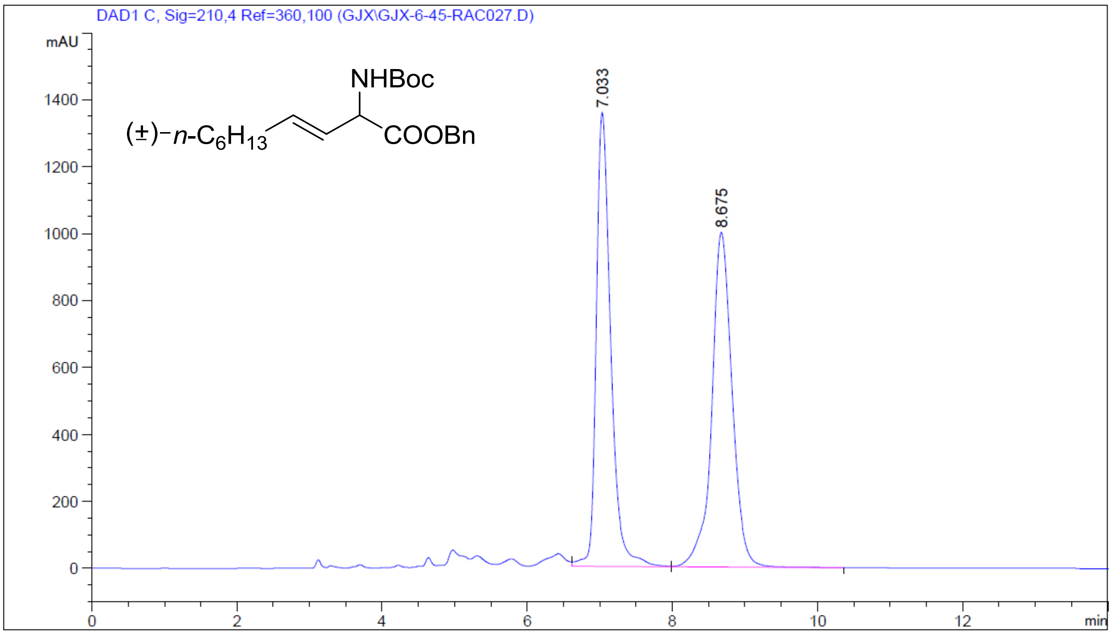

Peak #	RetTime [min]	Type	Width [min]	Area [mAU*s]	Height [mAU]	Area %
1	7.033	VV	0.2087	1.86826e4	1356.54114	49.0208
2	8.675	VB	0.2927	1.94290e4	999.96375	50.9792

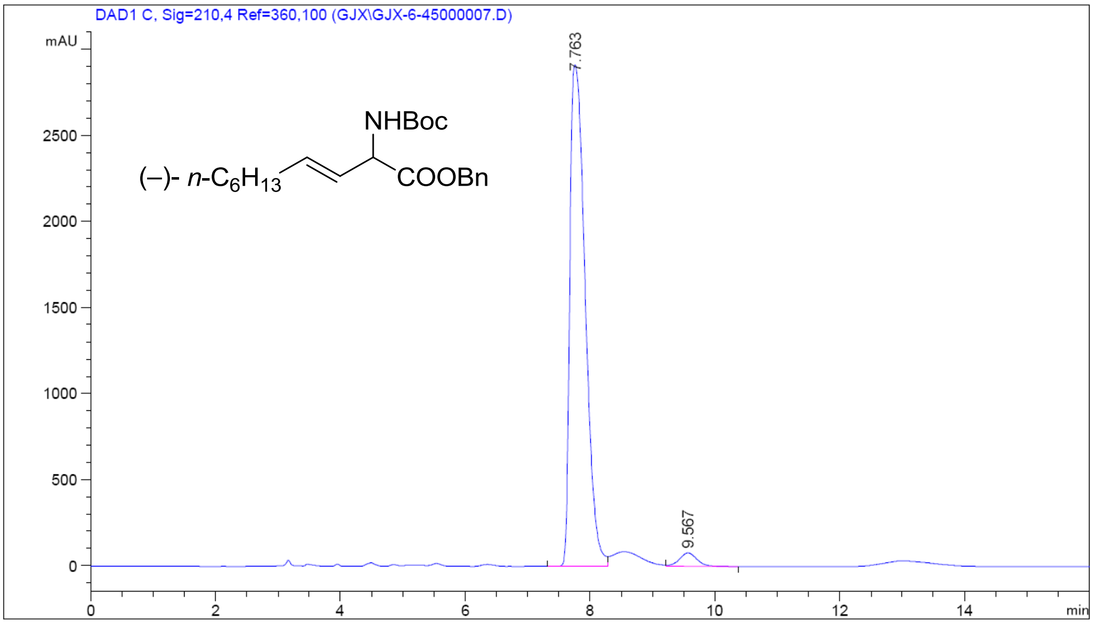

Peak #	RetTime [min]	Type	Width [min]	Area [mAU*s]	Height [mAU]	Area %
1	7.763	BV	0.2712	4.96454e4	2908.18677	96.9512
2	9.567	VB	0.3076	1561.20471	78.65253	3.0488

(*E*)-Benzyl 2-(*tert*-butoxycarbonylamino)-5-methylhex-3-enoate (2e)

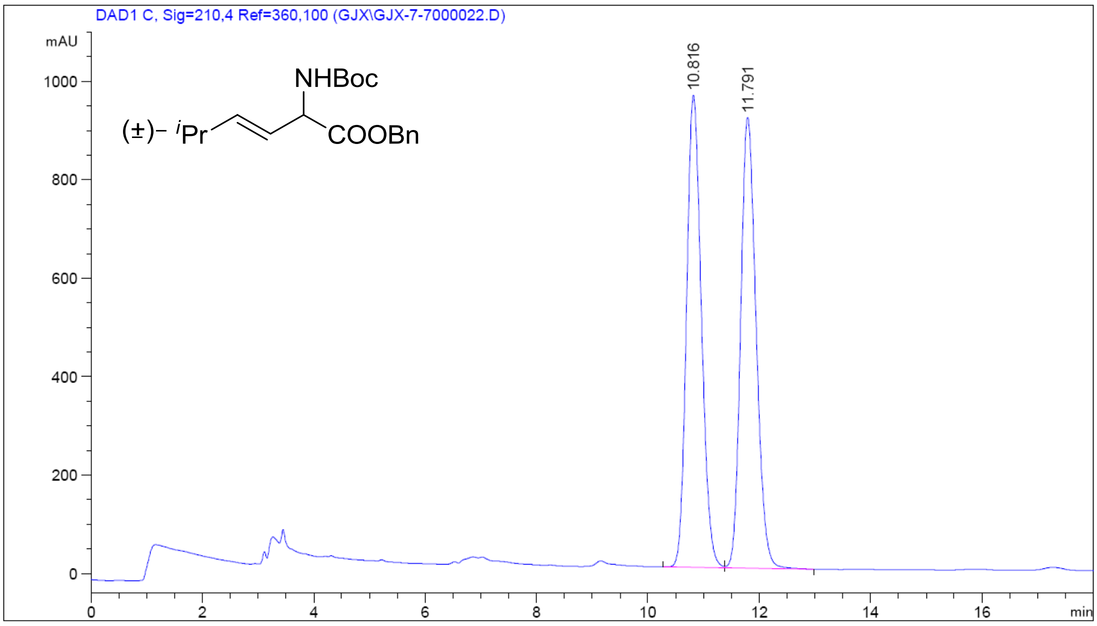

Peak #	RetTime [min]	Type	Width [min]	Area [mAU*s]	Height [mAU]	Area %
1	10.816	BV	0.2857	1.77537e4	960.30084	50.1417
2	11.791	VB	0.2987	1.76534e4	916.38843	49.8583

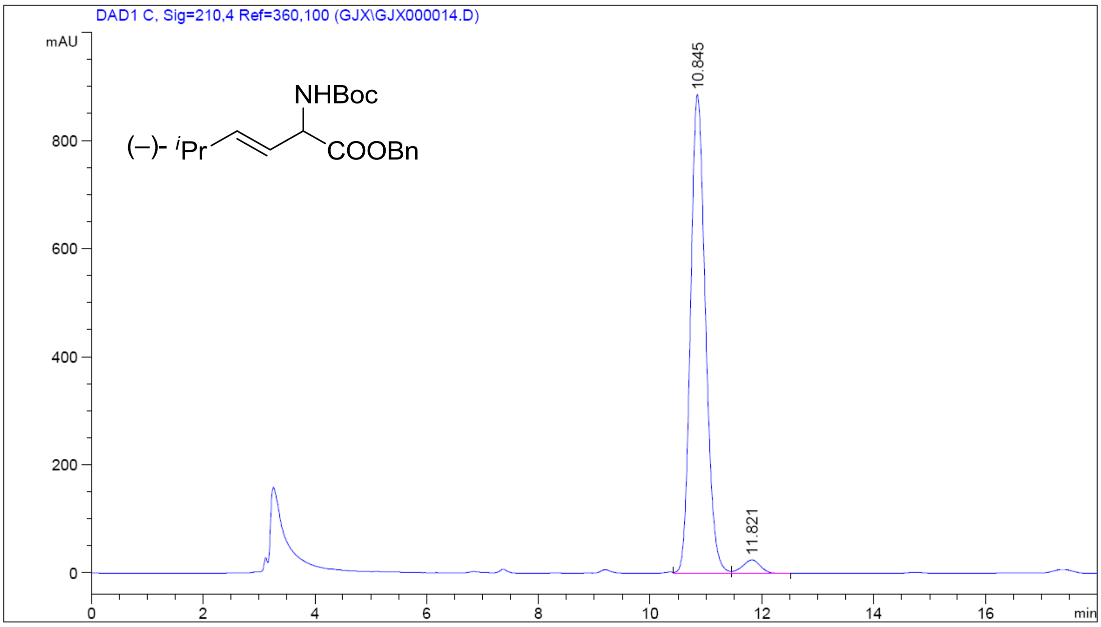

Peak #	RetTime [min]	Type	Width [min]	Area [mAU*s]	Height [mAU]	Area %
1	10.845	VV	0.2886	1.64495e4	886.06555	96.7305
2	11.821	VB	0.3369	555.98602	25.05516	3.2695

(*E*)-Benzyl 2-(*tert*-butoxycarbonylamino)-4-phenylbut-3-enoate (2f)

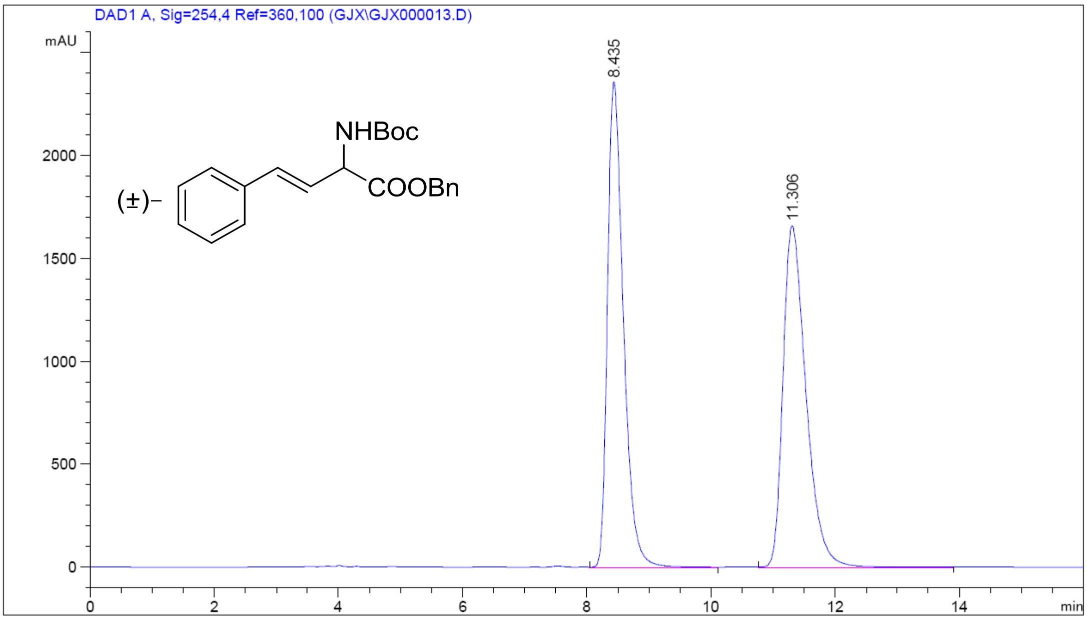

Peak #	RetTime [min]	Type	Width [min]	Area [mAU*s]	Height [mAU]	Area %
1	8.435	VB	0.2714	4.19177e4	2357.96997	49.5928
2	11.306	VV	0.3938	4.26060e4	1657.90076	50.4072

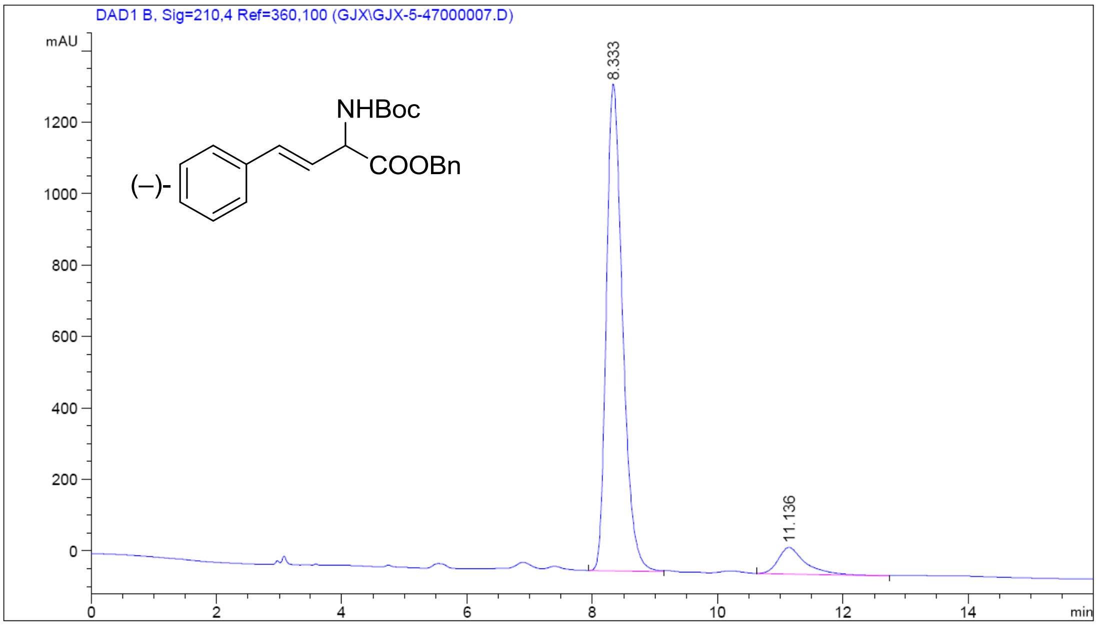

Peak #	RetTime [min]	Type	Width [min]	Area [mAU*s]	Height [mAU]	Area %
1	8.333	BV	0.2737	2.44789e4	1361.86194	91.4836
2	11.136	VV	0.4434	2278.78320	74.79577	8.5164

(*E*)-Benzyl 2-(*tert*-butoxycarbonylamino)-4-phenylpent-3-enoate (2g)

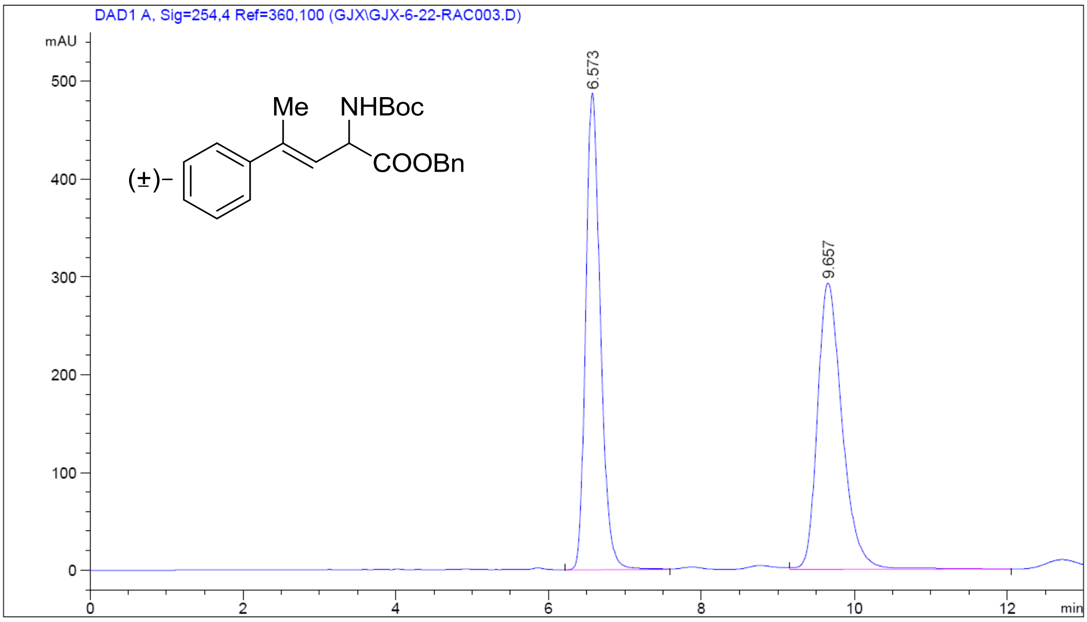

Peak #	RetTime [min]	Type	Width [min]	Area [mAU*s]	Height [mAU]	Area %
1	6.573	BV	0.2058	6512.42480	487.55612	49.5285
2	9.657	VB	0.3428	6586.52197	292.44870	50.0920

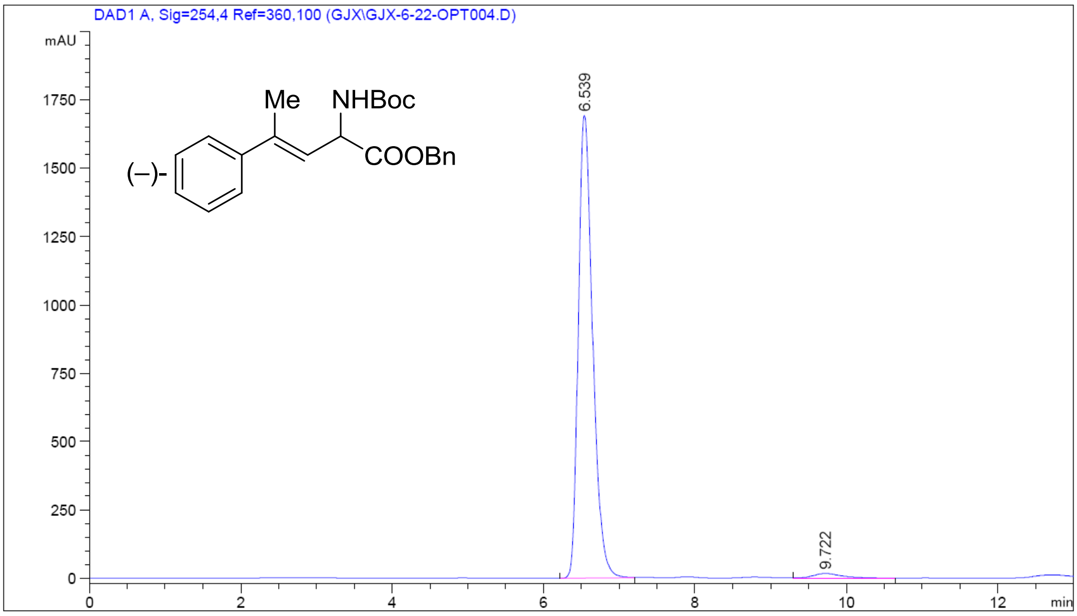

Peak #	RetTime [min]	Type	Width [min]	Area [mAU*s]	Height [mAU]	Area %
1	6.539	BV	0.2014	2.22544e4	1692.94983	98.0202
2	9.722	VB	0.3962	449.48950	16.90284	1.9798

(*E*)-Benzyl 2-(*tert*-butoxycarbonylamino)-4-(4-chlorophenyl)pent-3-enoate (2h)

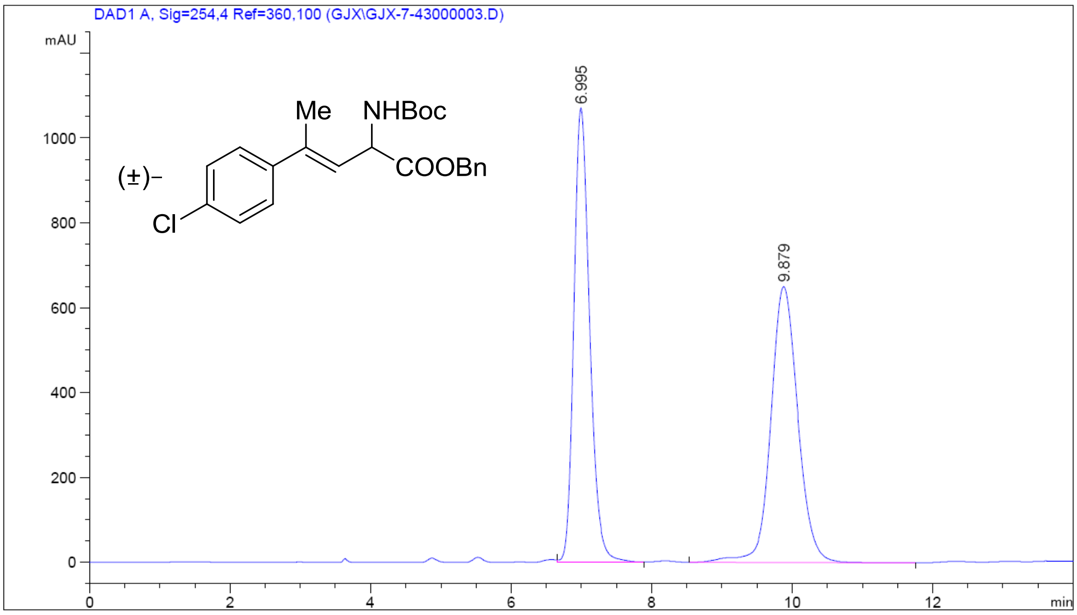

Peak #	RetTime [min]	Type	Width [min]	Area [mAU*s]	Height [mAU]	Area %
1	6.995	VV	0.2370	1.66028e4	1069.98157	48.8165
2	9.879	BB	0.4123	1.74078e4	650.22107	51.1835

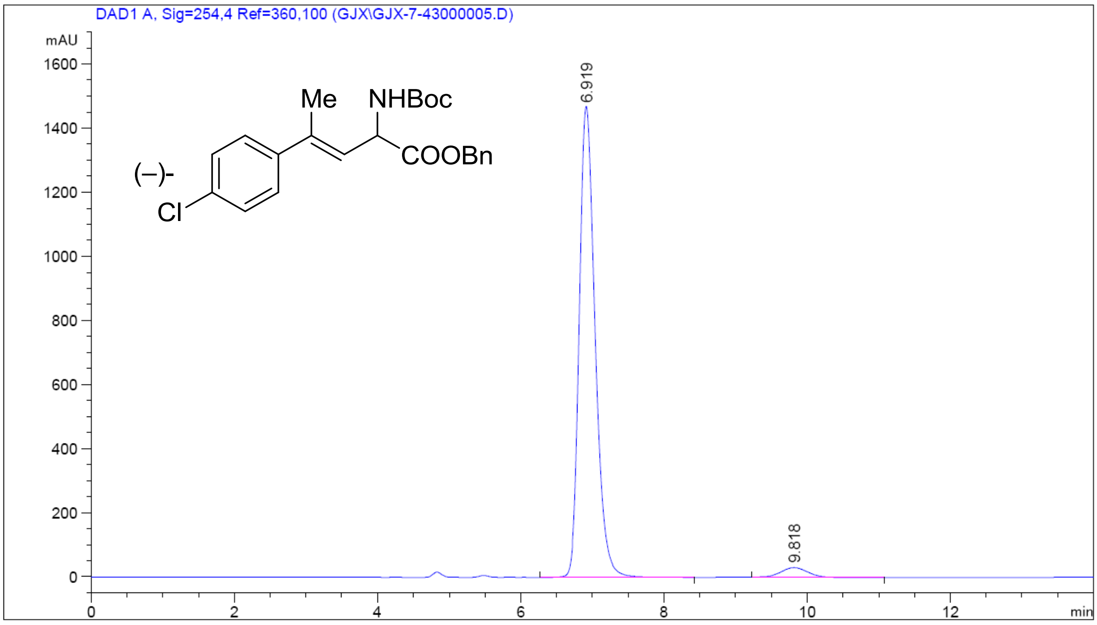

Peak #	RetTime [min]	Type	Width [min]	Area [mAU*s]	Height [mAU]	Area %
1	6.919	BB	0.2340	2.24221e4	1469.71277	96.4133
2	9.818	VB	0.4160	834.13776	30.79394	3.5867

(*E*)-Benzyl 2-(*tert*-butoxycarbonylamino)-4-(4-fluorophenyl)pent-3-enoate (2i)

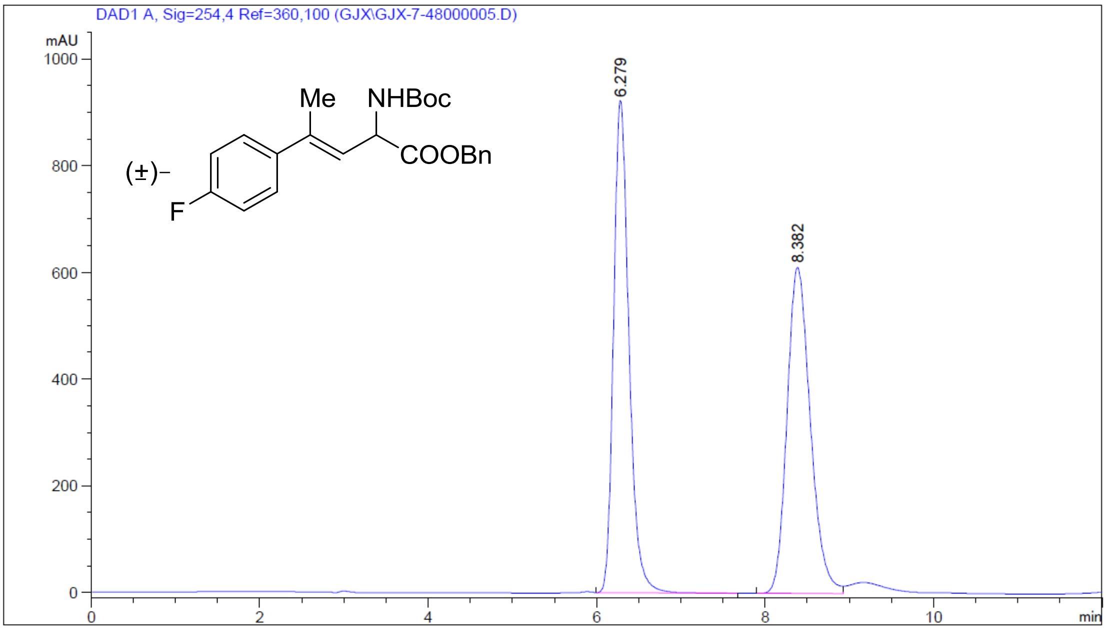

Peak #	RetTime [min]	Type	Width [min]	Area [mAU*s]	Height [mAU]	Area %
1	6.279	VB	0.1963	1.17195e4	922.18781	49.9090
2	8.382	BV	0.2967	1.17623e4	610.57947	50.0910

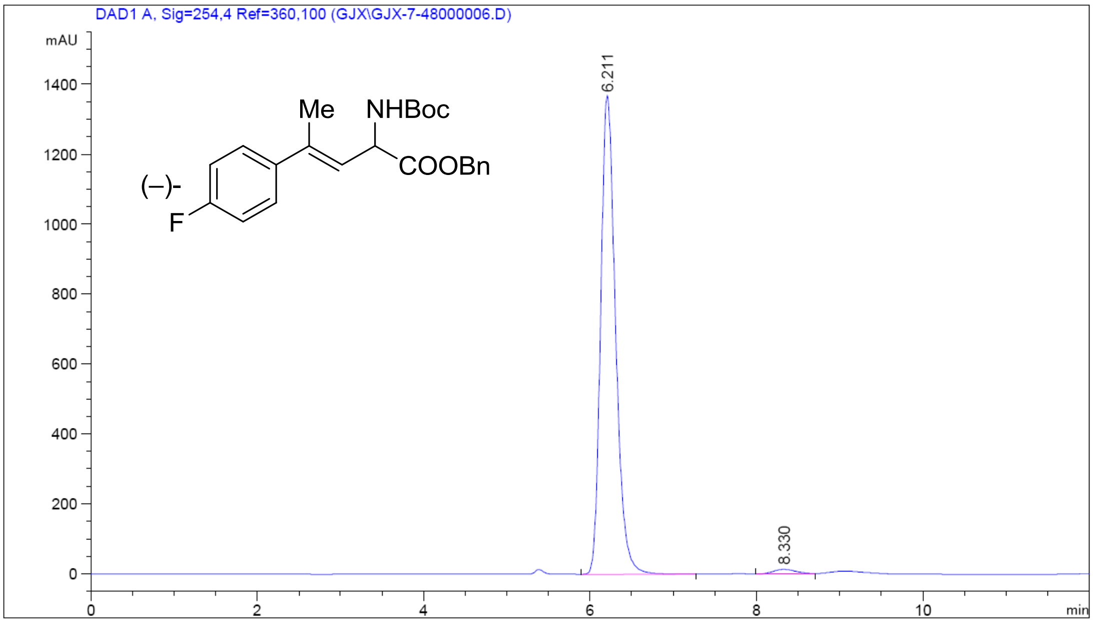

Peak #	RetTime [min]	Type	Width [min]	Area [mAU*s]	Height [mAU]	Area %
1	6.211	BV	0.1930	1.70057e4	1368.41260	98.4122
2	8.330	BV	0.3093	274.36639	13.72074	1.5878

(*E*)-Benzyl 2-(*tert*-butoxycarbonylamino)-4-(4-methoxyphenyl)pent-3-enoate (2j)

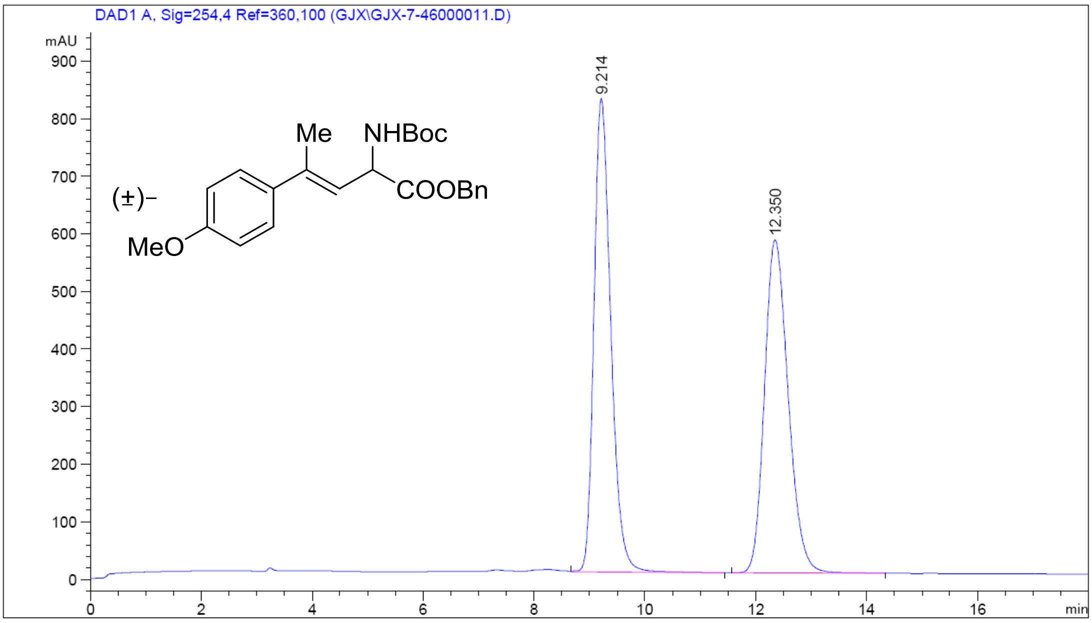

Peak #	RetTime [min]	Type	Width [min]	Area [mAU*s]	Height [mAU]	Area %
1	9.214	VB	0.3222	1.73453e4	821.97418	50.2109
2	12.350	BB	0.4589	1.71996e4	578.36987	49.7891

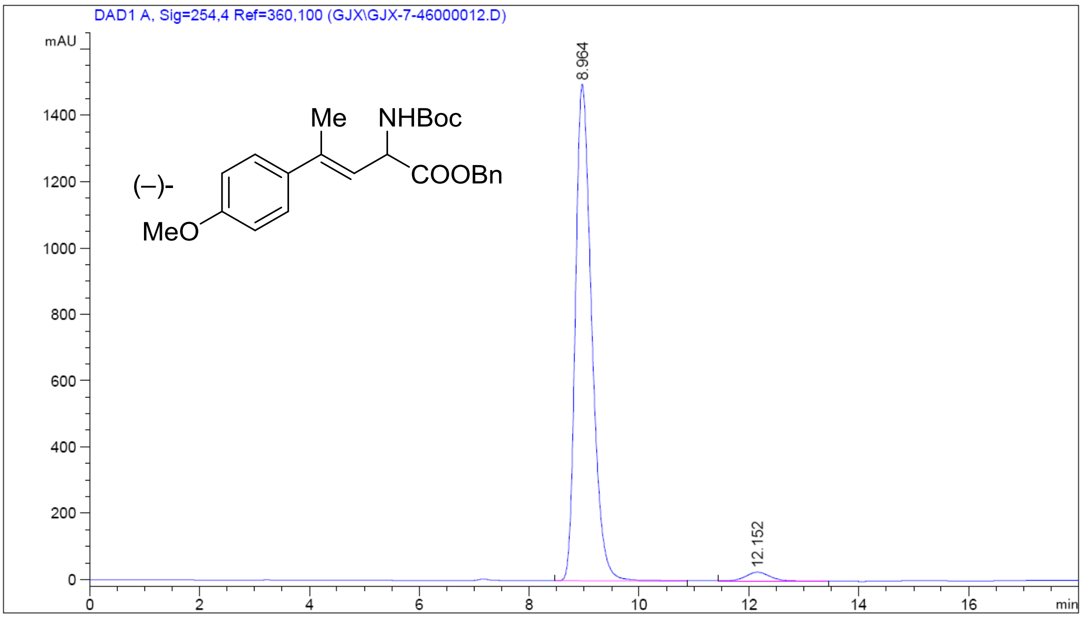

Peak #	RetTime [min]	Type	Width [min]	Area [mAU*s]	Height [mAU]	Area %
1	8.964	BV	0.3157	3.07674e4	1497.23352	97.1728
2	12.152	VV	0.5032	895.16010	26.97139	2.8272

(*E*)-Benzyl 2-(*tert*-butoxycarbonylamino)-4-(naphthalen-2-yl)pent-3-enoate (2k)

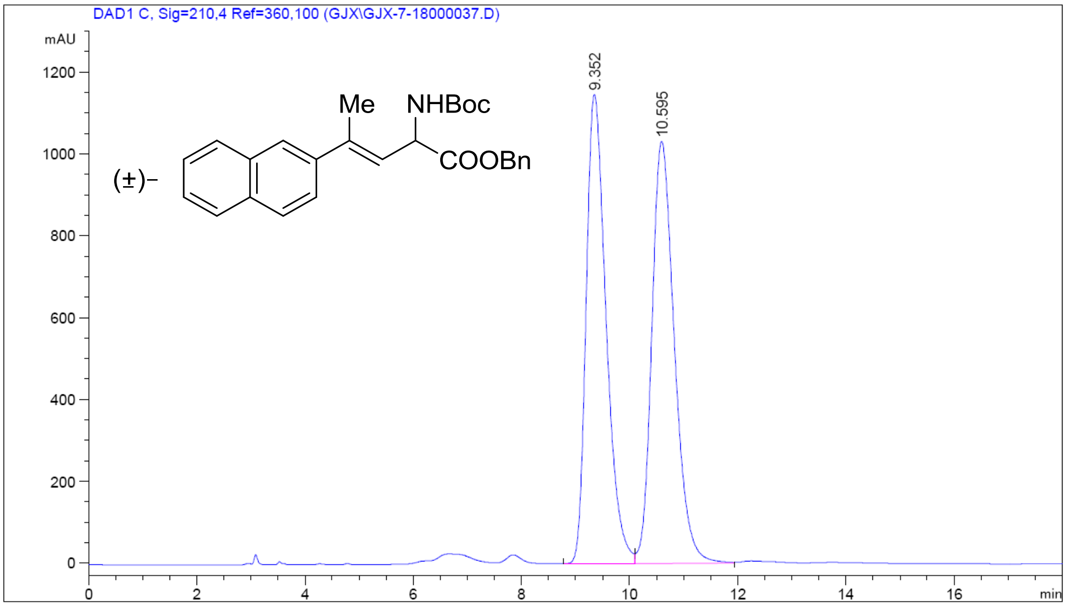

Peak #	RetTime [min]	Type	Width [min]	Area [mAU*s]	Height [mAU]	Area %
1	9.352	BV	0.3919	2.92637e4	1145.95276	49.4687
2	10.595	VV	0.4463	2.98923e4	1030.86682	50.5313

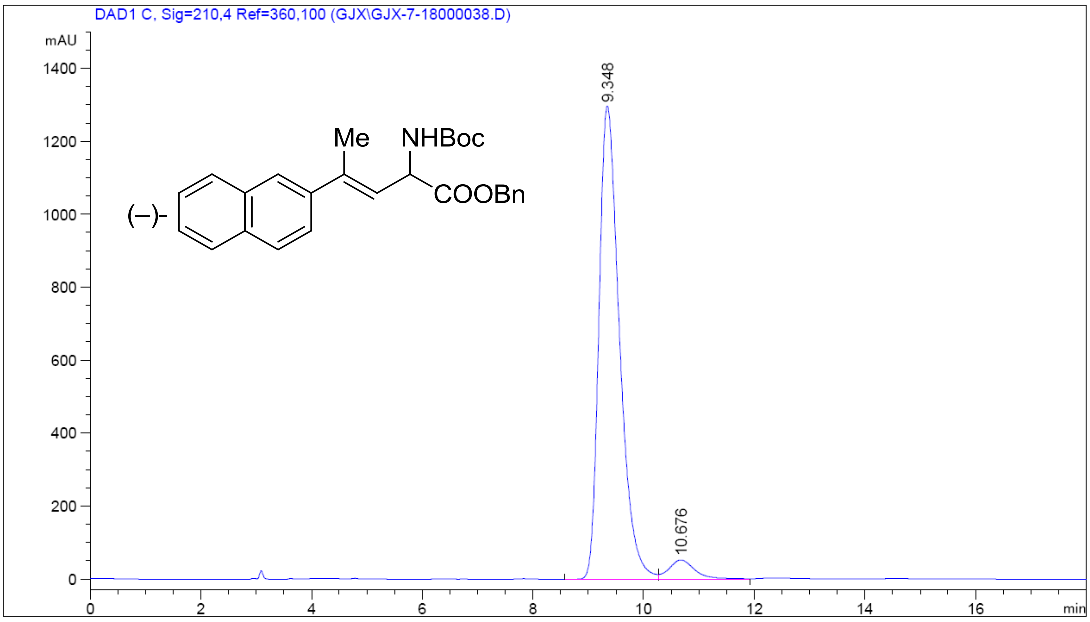

Peak #	RetTime [min]	Type	Width [min]	Area [mAU*s]	Height [mAU]	Area %
1	9.348	BV	0.3944	3.34380e4	1298.58557	94.9661
2	10.676	VV	0.5010	1772.46387	52.88394	5.0339

(*E*)-Benzyl-4-(benzo[d][1,3]dioxol-5-yl)-2-(*tert*-butoxycarbonylamino)pent-3-enoate (2l)

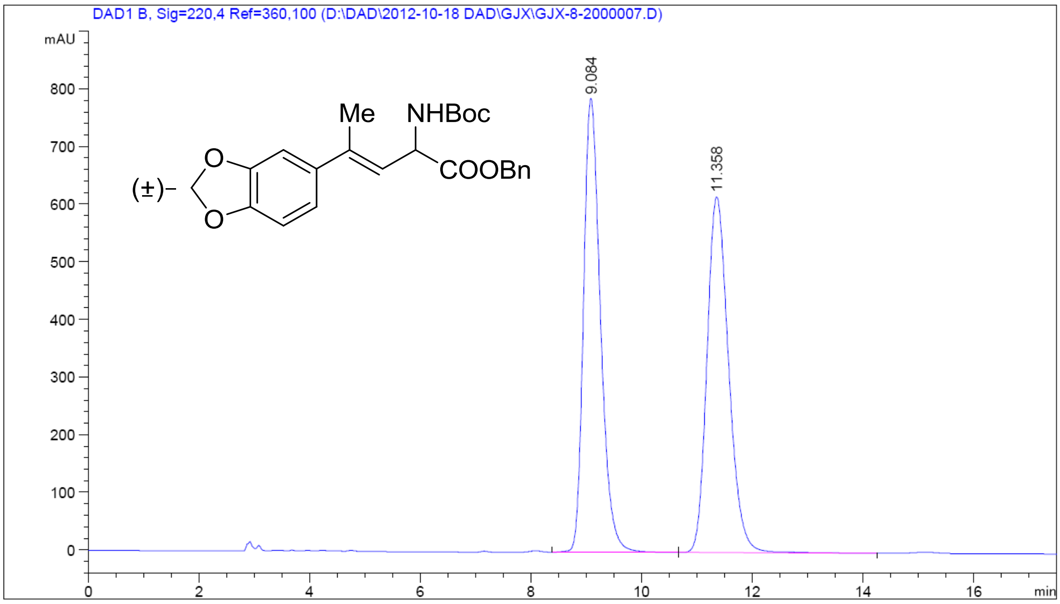

Peak #	RetTime [min]	Type	Width [min]	Area [mAU*s]	Height [mAU]	Area %
1	9.084	VB	0.3280	1.67378e4	787.32544	49.9552
2	11.358	BV	0.4210	1.67678e4	617.05975	50.0448

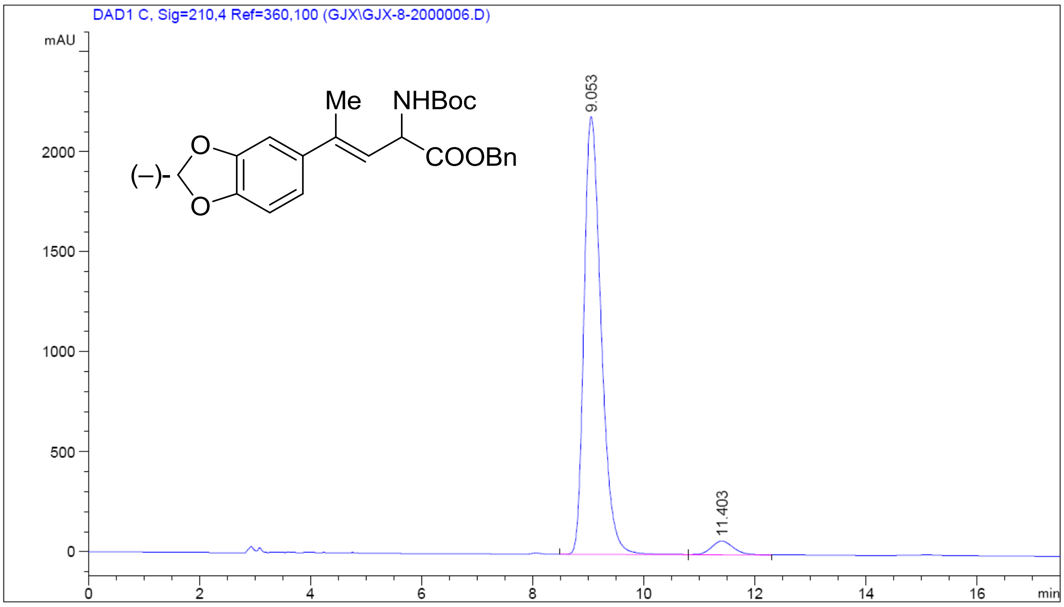

Peak #	RetTime [min]	Type	Width [min]	Area [mAU*s]	Height [mAU]	Area %
1	9.053	BV	0.3343	4.69577e4	2188.98853	96.0242
2	11.403	VV	0.4323	1944.25269	69.08539	3.9758

(*E*)-Benzyl 2-(*tert*-butoxycarbonylamino)-4-(thiophen-2-yl)pent-3-enoate (2m)

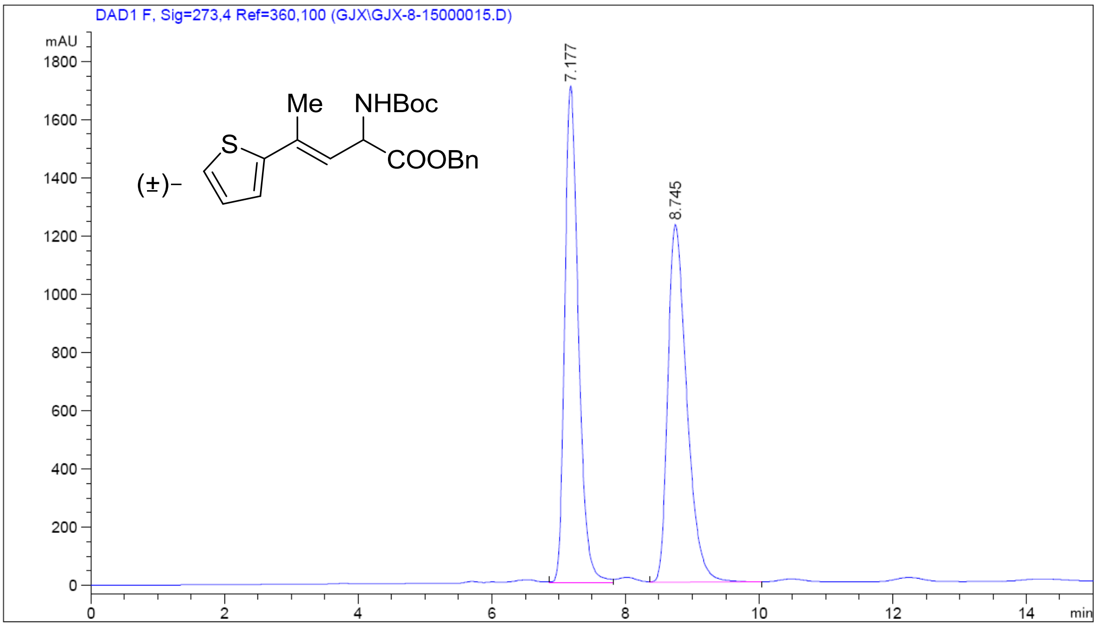

Peak #	RetTime [min]	Type	Width [min]	Area [mAU*s]	Height [mAU]	Area %
1	7.177	VV	0.2178	2.42105e4	1703.89233	49.9931
2	8.745	VB	0.3020	2.42173e4	1228.32629	50.0069

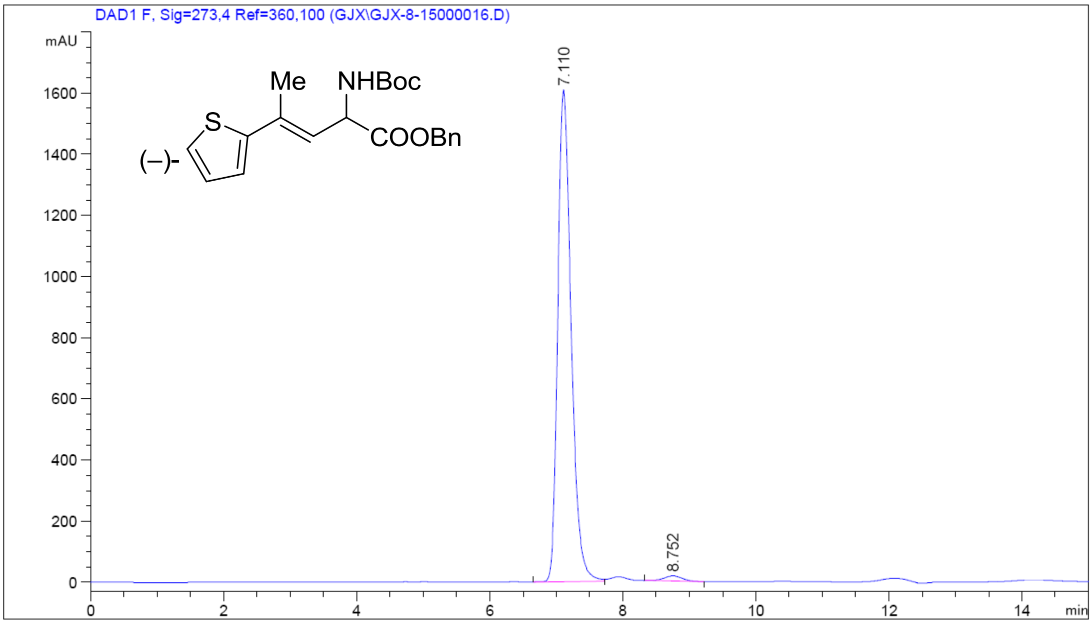

Peak #	RetTime [min]	Type	Width [min]	Area [mAU*s]	Height [mAU]	Area %
1	7.110	BV	0.2149	2.24545e4	1608.19812	98.5756
2	8.752	BB	0.2977	324.45999	16.76851	1.4244

Benzyl 2-(*tert*-butoxycarbonylamino)-4-methylpent-3-enoate (2n)

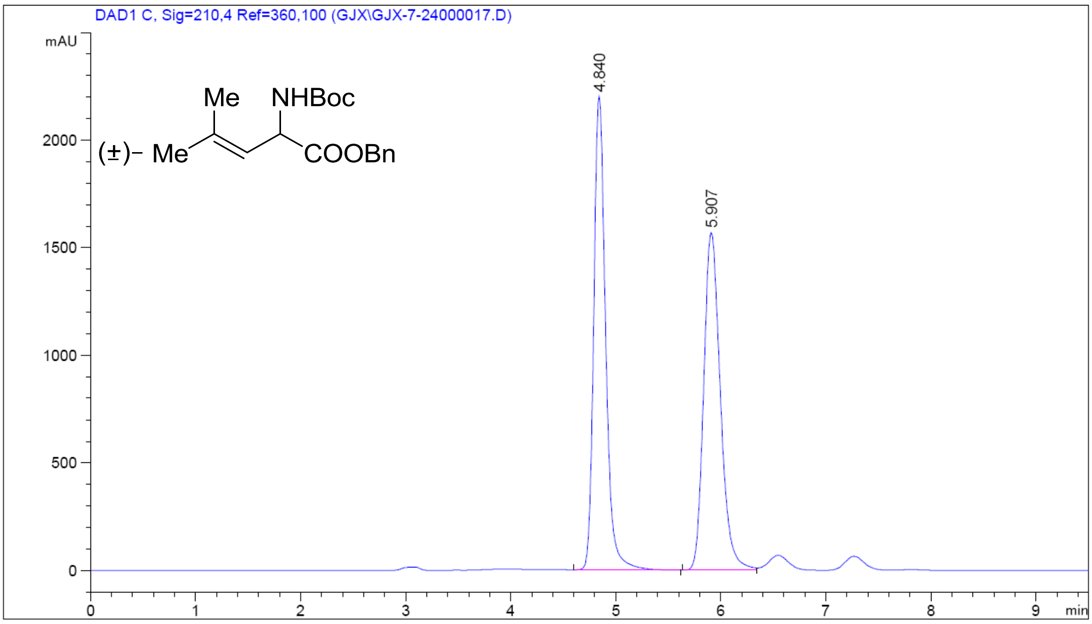

Peak #	RetTime [min]	Type	Width [min]	Area [mAU*s]	Height [mAU]	Area %
1	4.840	BB	0.1236	1.72699e4	2200.69092	49.9537
2	5.907	BV	0.1686	1.73019e4	1569.05139	50.0463

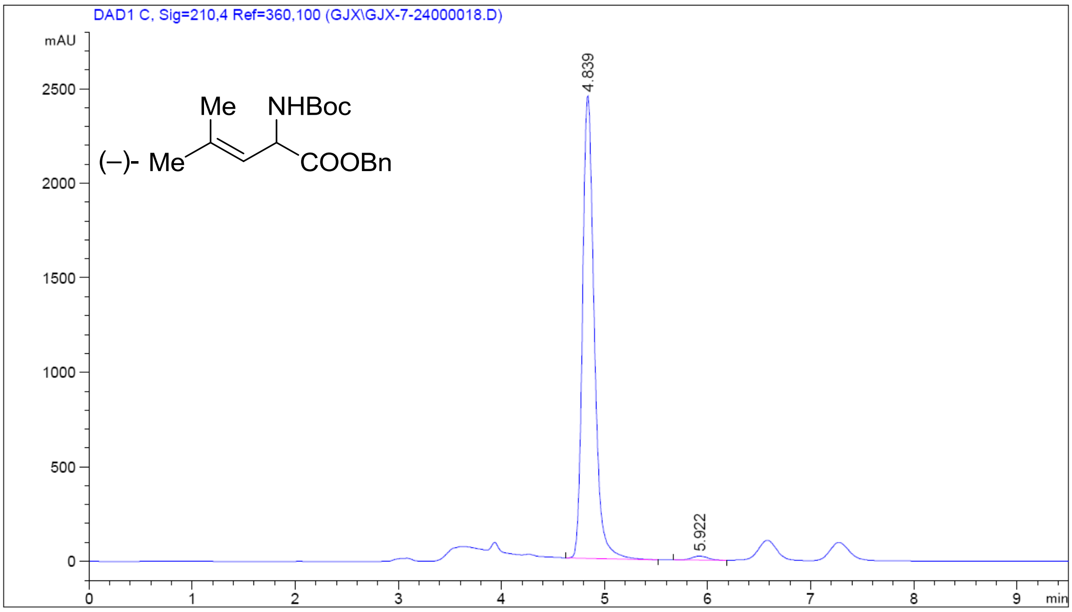

Peak #	RetTime [min]	Type	Width [min]	Area [mAU*s]	Height [mAU]	Area %
1	4.839	BB	0.1239	1.92541e4	2444.36353	98.8164
2	5.922	BB	0.1648	230.61382	21.55677	1.1836

Benzyl 2-(*tert*-butoxycarbonylamino)but-3-enoate (2o)

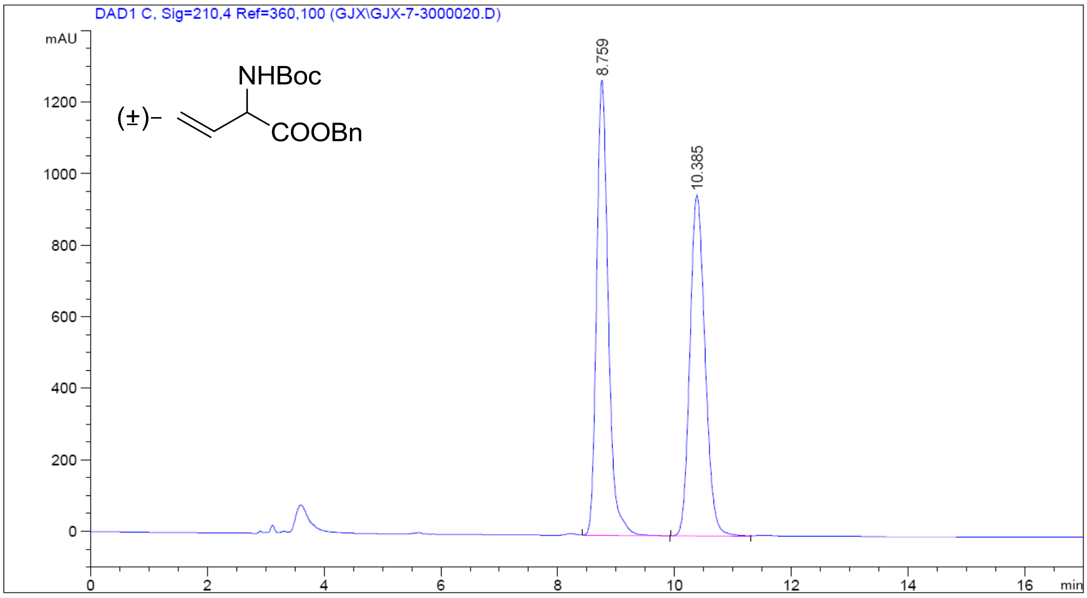

Peak #	RetTime [min]	Type	Width [min]	Area [mAU*s]	Height [mAU]	Area %
1	8.759	VB	0.2136	1.76488e4	1273.85620	50.6494
2	10.385	BV	0.2806	1.71962e4	952.67725	49.3506

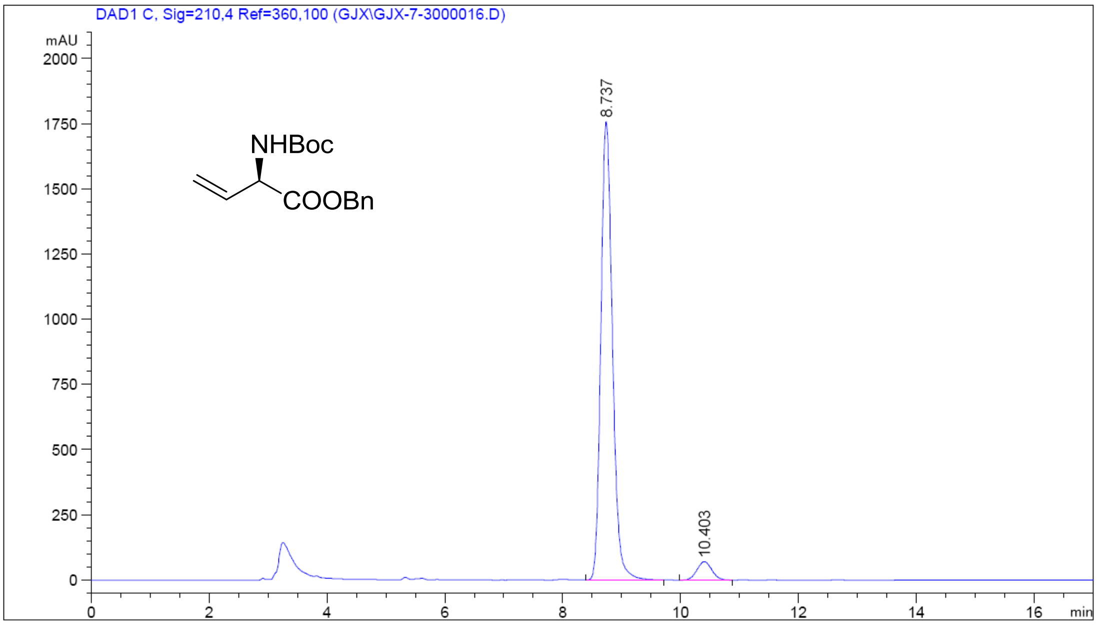

Peak #	RetTime [min]	Type	Width [min]	Area [mAU*s]	Height [mAU]	Area %
1	8.737	BB	0.2109	2.39160e4	1756.37842	95.0736
2	10.403	BB	0.2758	1239.23535	70.24461	4.9264

Ethyl 2-(*tert*-butoxycarbonylamino)-2-cyclohexenylacetate (2p)

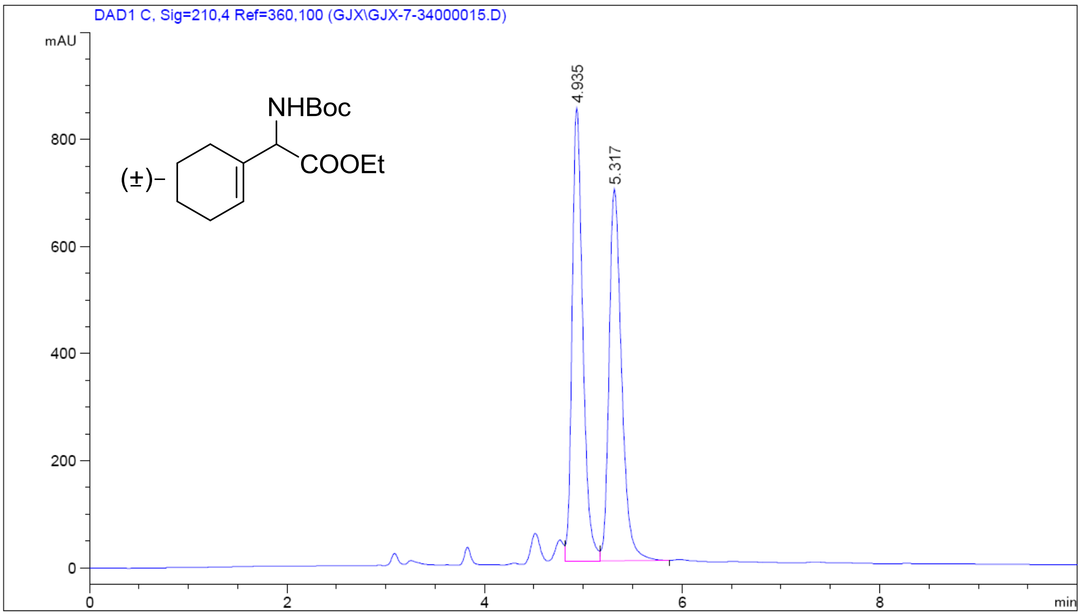

Peak #	RetTime [min]	Type	Width [min]	Area [mAU*s]	Height [mAU]	Area %
1	4.935	VV	0.1133	6330.91016	846.17407	50.8157
2	5.317	VB	0.1349	6127.64893	694.57050	49.1843

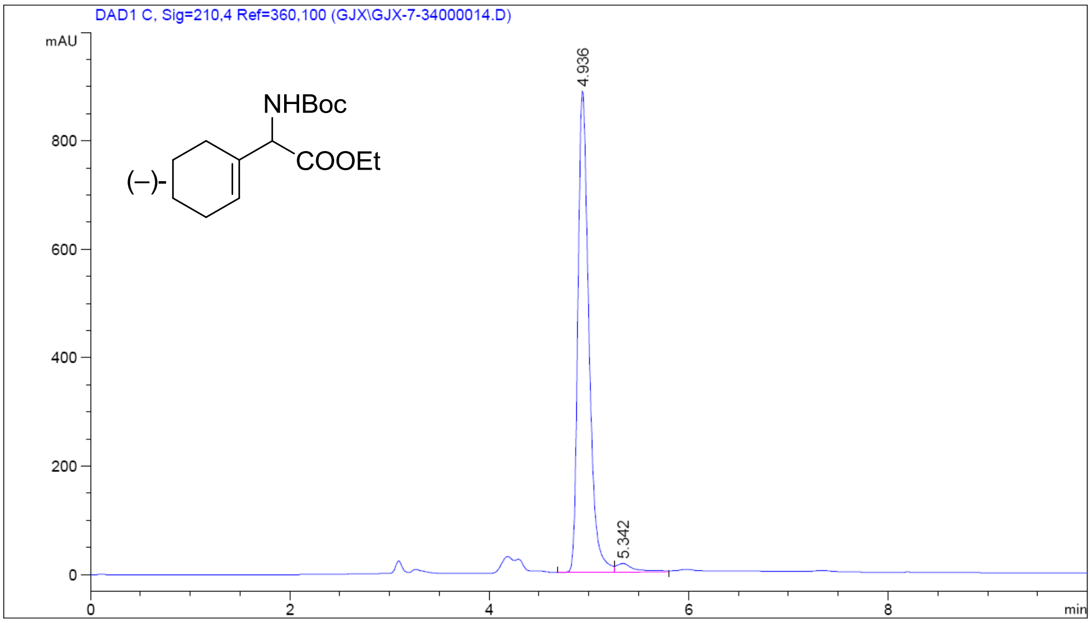

Peak #	RetTime [min]	Type	Width [min]	Area [mAU*s]	Height [mAU]	Area %
1	4.936	BV	0.1172	6788.60840	888.00256	96.8737
2	5.342	VV	0.1879	219.07800	16.18241	3.1263

(*E*)-Benzyl 5-(benzyloxy)-2-(*tert*-butoxycarbonylamino)pent-3-enoate (2q)

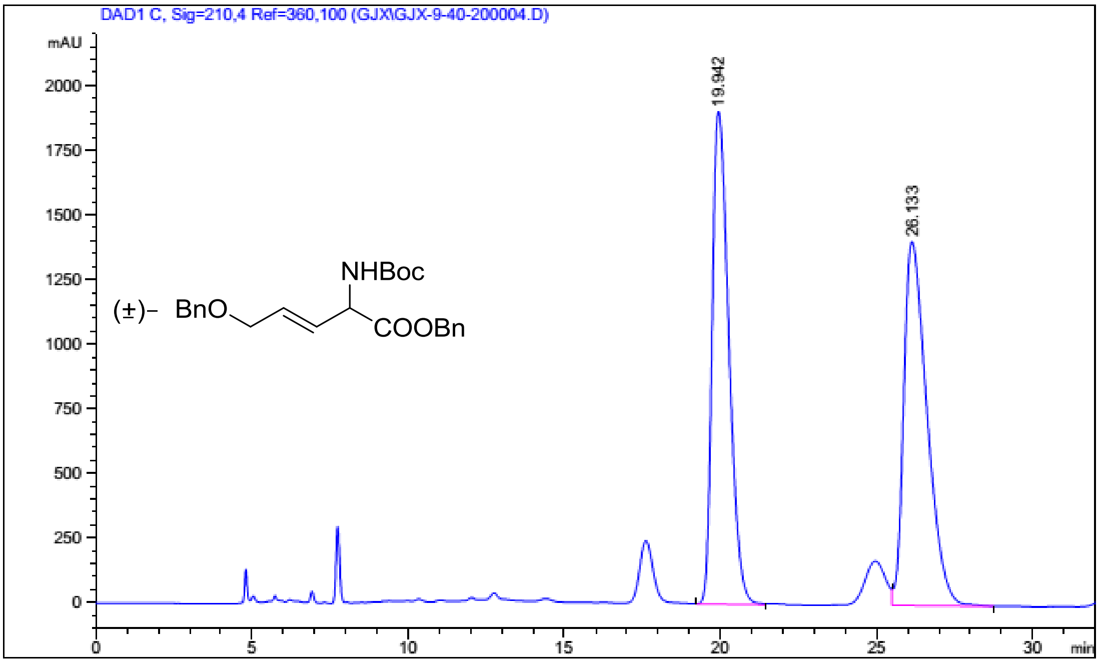

Peak #	RetTime [min]	Type	Width [min]	Area [mAU*s]	Height [mAU]	Area %
1	19.942	VV	0.5789	7.10786e4	1906.59277	49.4628
2	26.133	VB	0.7699	7.26225e4	1407.57996	50.5372

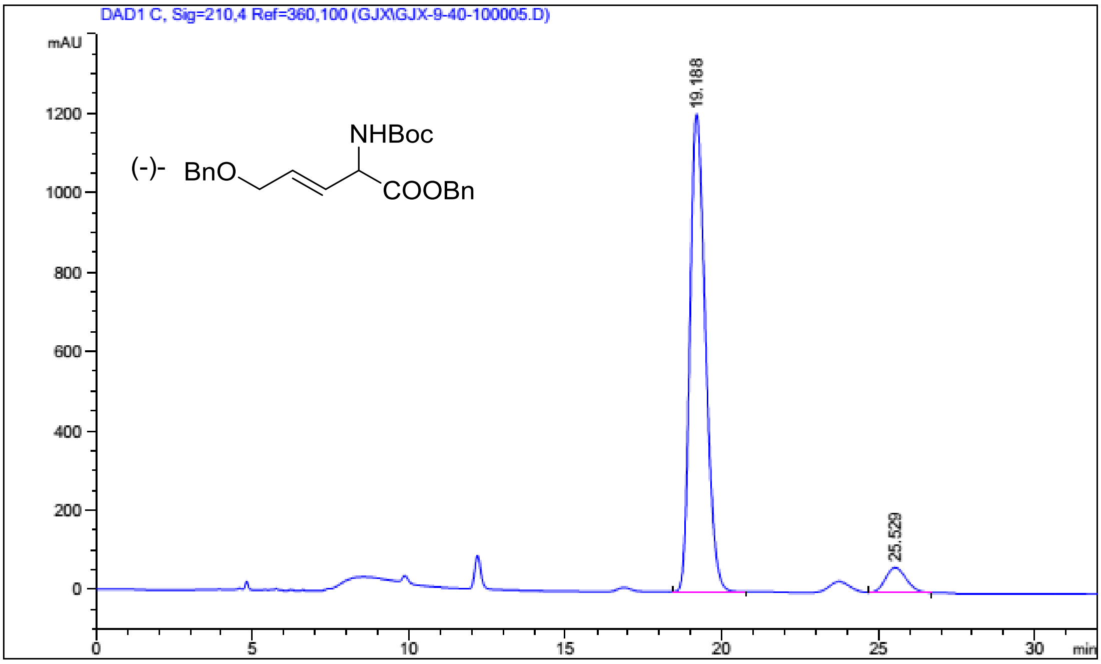

Peak #	RetTime [min]	Type	Width [min]	Area [mAU*s]	Height [mAU]	Area %
1	19.188	BB	0.5264	4.15265e4	1204.09241	93.3830
2	25.529	VB	0.5573	2942.53101	63.89362	6.6170

(*E*)-Methyl 2-(*tert*-butoxycarbonylamino)-4-phenylpent-3-enoate (2r)

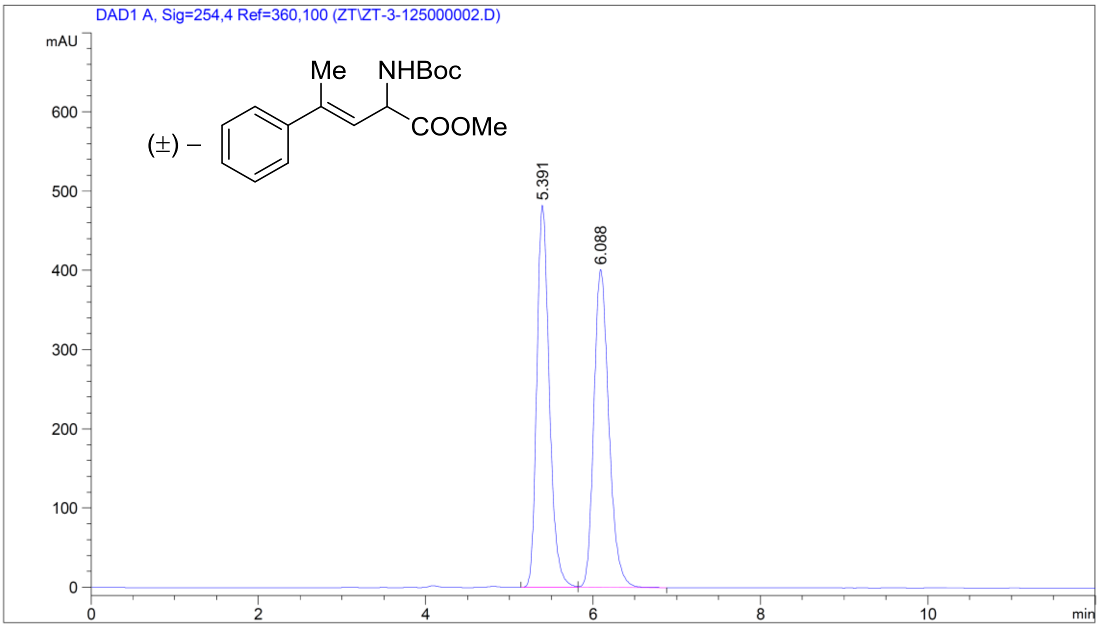

Peak #	RetTime [min]	Type	Width [min]	Area [mAU*s]	Height [mAU]	Area %
1	5.391	BV	0.1593	5015.86279	482.43726	50.0634
2	6.088	VB	0.1914	5003.14941	401.43359	49.9366

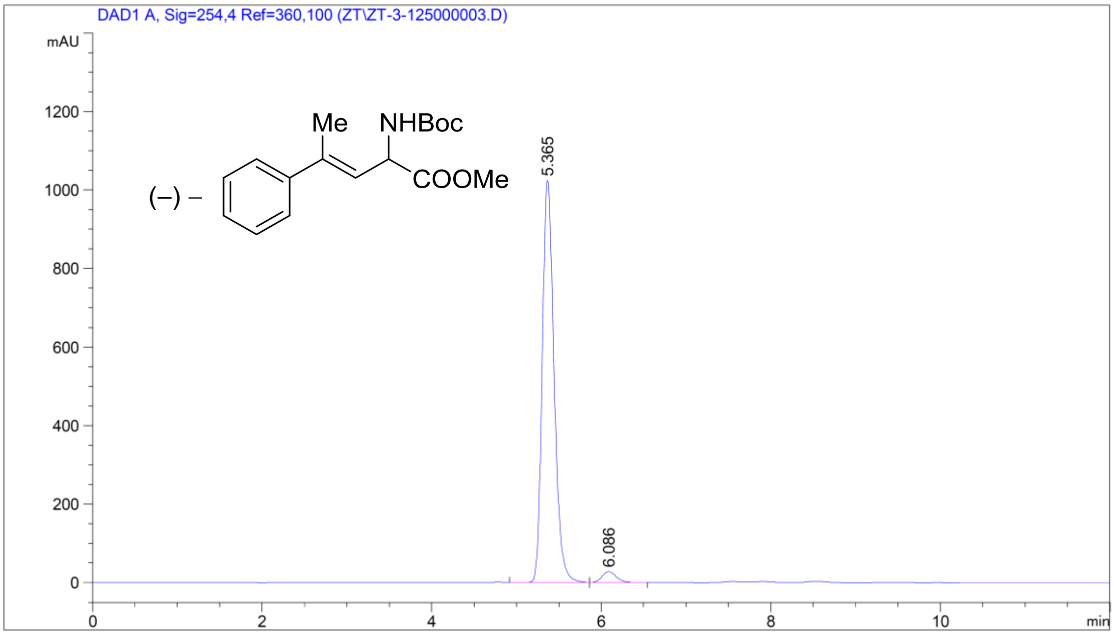

Peak #	RetTime [min]	Type	Width [min]	Area [mAU*s]	Height [mAU]	Area %
1	5.365	BV	0.1509	9904.03809	1023.81274	96.7442
2	6.086	VB	0.1819	333.31058	28.20852	3.2558

(*E*)-*tert*-Butyl 2-(*tert*-butoxycarbonylamino)-4-phenylpent-3-enoate (2s)

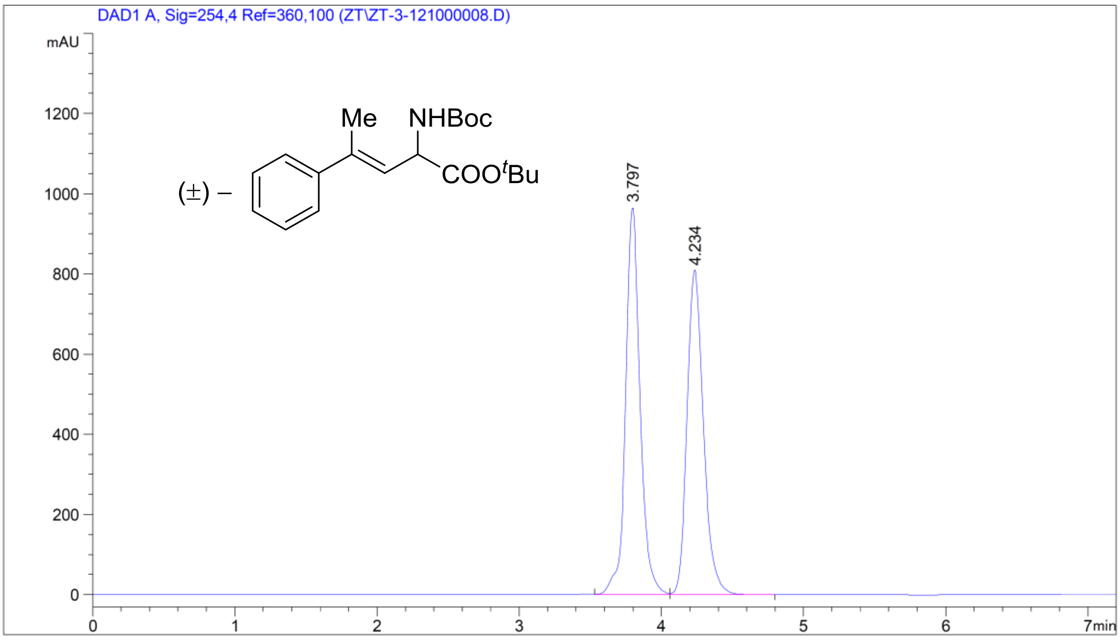

Peak #	RetTime [min]	Type	Width [min]	Area [mAU*s]	Height [mAU]	Area %
1	3.797	VV	0.1066	6844.79883	967.06610	51.2909
2	4.234	VB	0.1237	6500.25586	809.63422	48.7091

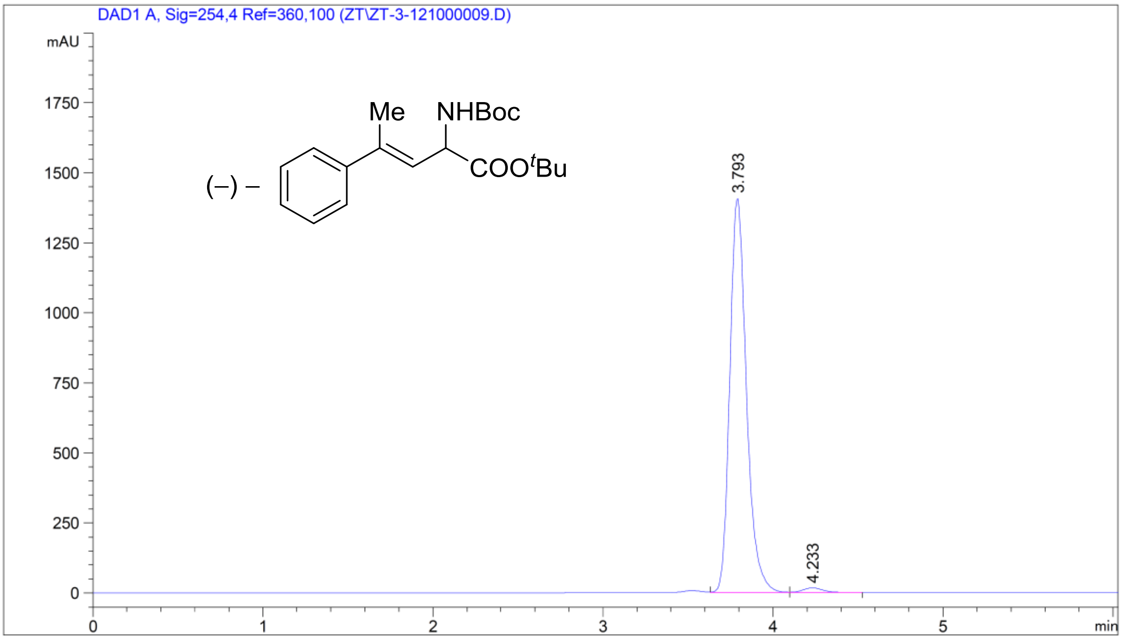

Peak #	RetTime [min]	Type	Width [min]	Area [mAU*s]	Height [mAU]	Area %
1	3.793	VV	0.1032	9567.33008	1409.60132	98.5523
2	4.233	VB	0.1238	140.54015	17.11239	1.4477

9. References

1 W. L. F. Armarego and C. L. L. Chai, *Purification of Laboratory Chemicals-Six Edition*; Elsevier Inc., London, 2009.

2 B. Xu, S.-F. Zhu, X.-L. Xie, J.-J. Shen and Q.-L. Zhou, *Angew. Chem. Int. Ed.*, 2011, **50**, 11483.

3 (a) P. Bulugahapitiya, Y. Landais, L. Parra-Rapado, D. Planchenault and V. Weber, *J. Org. Chem.*, 1997, **62**, 1630; (b) M. P. Doyle, M. Yan, W. H. Hu and L. S. Gronenberg, *J. Am. Chem. Soc.*, 2003, **125**, 4692; (c) B. D. Schwartz, J. R. Denton, Y. Lian, H. M. L. Davies and C. M. Williams, *J. Am. Chem. Soc.*, 2009, **131**, 8329.

4 Y. Yang, S.-F. Zhu, H.-F. Duan, C.-Y. Zhou, L.-X. Wang and Q.-L. Zhou, *J. Am. Chem. Soc.*, 2007, **129**, 2248.

5 K. O. Hallinan, D. H. G. Crout and W. Errington, *J. Chem. Soc., Perkin Trans.1*, 1994, 3537.

6 P. A. Lander and L. S. Hegedus, *J. Am. Chem. Soc.*, 1994, **116**, 8126.
